# Supplementary material for: SurgiCal Obesity Treatment Study (SCOTS): a prospective, observational cohort study on health and socioeconomic burden in treatment-seeking individuals with severe obesity in Scotland, UK
Source: BMJ Open. 2021 Aug 26;11(8):e046441. doi: 10.1136/bmjopen-2020-046441 (PMC8395268; doi:10.1136/bmjopen-2020-046441)
Supplement: Supplementary data [file bmjopen-2020-046441supp001.pdf]

Supplementary Table 1. STROBE Statement completed checklist (from von Elm *et al.* [1]).

SECTION/TOPIC	ITEM No.	STROBE Recommendation	OUR PAPER
**Title and abstract**	1	(a) Indicate the study's design with a commonly used term in the title or the abstract	(a) See title
		(b) Provide in the abstract an informative and balanced summary of what was done and what was found	(b) See abstract
**Introduction**			
Background / rationale	2	Explain the scientific background and rationale for the investigation being reported	Paragraphs 1-3 provide background and paragraph 4 explains rationale
Objectives	3	State specific objectives, including any prespecified hypotheses	Paragraph 4
**Methods**			
Study design	4	Present key elements of study design early in the paper	Paragraph 3/'Study Design' section
Setting	5	Describe the setting, locations, and relevant dates, including periods of recruitment, exposure, follow-up, and data collection	Paragraphs 4-6/'Participant and Centre Eligibility', 'Recruitment Procedures and Consent' and 'Data Collection' sections
Participants	6	(a) Give the eligibility criteria, and the sources and methods of selection of participants. Describe methods of follow-up	(a) Paragraphs 4-6/'Participant and Centre Eligibility', 'Recruitment Procedures and Consent' and 'Data Collection' sections
		(b) For matched studies, give matching criteria and number of exposed and unexposed	(b) Not applicable
Variables	7	Clearly define all outcomes, exposures, predictors, potential confounders, and effect modifiers. Give diagnostic criteria, if applicable	Paragraphs 7-9/'Data Collection' section
Data sources / measurement	8	For each variable of interest, give sources of data and details of methods of assessment (measurement). Describe comparability of assessment methods if there is more than one group	Paragraphs 7 and 8/'Data Collection' section
Bias	9	Describe any efforts to address potential sources of bias	Not applicable
Study size	10	Explain how the study size was arrived at	Paragraph 4/'Patient and Centre Eligibility' section, paragraph 1 of 'Results' section

Quantitative variables	11	Explain how quantitative variables were handled in the analyses. If applicable, describe which groupings were chosen and why	'Statistical Analyses' section
Statistical methods	12	(a) Describe all statistical methods, including those used to control for confounding	(a) 'Statistical Analyses' section
		(b) Describe any methods used to examine subgroups and interactions	(b) Not applicable
		(c) Explain how missing data were addressed	(c) 'Statistical Analyses' section
		(d) If applicable, explain how loss to follow-up was addressed	(d) Not applicable
		(e) Describe any sensitivity analyses	(e) Not applicable
**Results**			
Participants	13	(a) Report numbers of individuals at each stage of study— e.g. numbers potentially eligible, examined for eligibility, confirmed eligible, included in the study, completing follow-up, and analysed	(a) Paragraph 1 and Figure 1
		(b) Give reasons for non-participation at each stage	(b) Paragraph 1 and Figure 1
		(c) Consider use of flow diagram	(c) Figure 1
Descriptive data	14	(a) Give characteristics of study participants (e.g. demographic, clinical, social) and information on exposures and potential confounders	(a) Paragraphs 2-5/'Characteristics of recruited and analysed sample', 'Comorbidities', "Health and obesity-related quality of life' and physical activity' sections, Table 1 and Table 2
		(b) Indicate number of participants with missing data for each variable of interest	(b) Paragraphs 2-7, Table 1, Table 2, Supplementary Table 2 and Supplementary Table 3
		(c) Summarise follow-up time (e.g. average and total amount)	(c) Not applicable
Outcome data	15	Report numbers of outcome events or summary measures over time	Table 2, Supplementary Table 2 and Supplementary Table 3
Main results	16	(a) Give unadjusted estimates and, if applicable, confounder-adjusted estimates and their precision (e.g. 95% confidence interval). Make clear which confounders were adjusted for and why they were included	(a) Table 3 and Supplementary Table 4
		(b) Report category boundaries when continuous variables were categorised	(b) Supplementary Table 4

		(c) If relevant, consider translating estimates of relative risk into absolute risk for a meaningful time period	(c) Not applicable
Other analyses	17	Report other analyses done e.g. analyses of subgroups and interactions, and	Not applicable
**Discussion**			
Key results	18	Summarise key results with reference to study objectives	Paragraph 1
Limitations	19	Discuss limitations of the study, taking into account sources of potential bias or imprecision. Discuss both direction and magnitude of any potential bias	Paragraph 5
Interpretation	20	Give a cautious overall interpretation of results considering objectives, limitations, multiplicity of analyses, results from similar studies, and other relevant evidence	'Conclusion'
Generalisability	21	Discuss the generalisability (external validity) of the study results	Paragraph 5
**Other**			
Funding	22	Give the source of funding and the role of the funders for the present study and, if applicable, for the original study on which the present article is based	'Funding' section

Reference

1) von EE, Altman DG, Egger M, Pocock SJ, Gotzsche PC, Vandenbroucke JP. The Strengthening the Reporting of Observational Studies in Epidemiology (STROBE) statement: guidelines for reporting observational studies. *Lancet* 2007; **370**(9596): 1453-1457
