# Supplementary material for: SurgiCal Obesity Treatment Study (SCOTS): a prospective, observational cohort study on health and socioeconomic burden in treatment-seeking individuals with severe obesity in Scotland, UK
Source: BMJ Open. 2021 Aug 26;11(8):e046441. doi: 10.1136/bmjopen-2020-046441 (PMC8395268; doi:10.1136/bmjopen-2020-046441)
Supplement: Supplementary data [file bmjopen-2020-046441supp002.pdf]

Glasgow Clinical Trials Unit

Surgical Obesity Treatment Study

SCOTS

Running title: **Surgical Obesity Treatment Study**

Protocol Version: **1.6**

Date: 22/11/2017

REC Reference Number: **13/WS/0005**

ISRCTN/Clinical trial.gov: **47072588**

Sponsor's Protocol Number: **GN21CA196**

Sponsor: **NHS Greater Glasgow & Clyde**

Funder: **National Institute for Health Research Health Technology Assessment Programme Ref 10/42/02**

**Date**	**Protocol version**	**Amendment number & description**
14/12/2012	1.0	Original
15/04/2013	1.1	Amendment 1: Minor changes to the format and presentation of the protocol
11/07/2013	1.2	Amendment 2: Correction of typographical errors in the protocol
30/06/2014	1.3	Amendment 3: Change to recruitment process for private hospitals involved in the study. Private hospitals will now become participant identification sites (and any NHS sites which in future are unable to support local recruitment), with participants being recruited and

		consented by a research team member (usually a research nurse).
03/06/2015	1.4	Amendment 4: Introduction of follow-up letter for patients recruited over the telephone by research nurses at the clinical research facility if completed consent form is not received within two weeks.
07/02/2017	1.5	Amendment 5; Revised study plan (including timetable & outputs). Patient recruitment stopping at a lower number and earlier than originally planned due to unforeseen recruitment issues. Amendment to clinical objectives and outcomes to reflect smaller sample size and shorter follow-up period. Reduction of post-operative questionnaire frequency to 2 and 3 years post-operatively only. Introduction of modest voucher incentives for PROMS completion.
22/11/2017	1.6	Amendment 6; Removal of annual blood test reminder. This procedure is completed as part of the routine clinical care by the patient's GP and therefore, it is not necessary for a reminder to be sent in addition for the SCOTS study.

This study will be performed according to the Research Governance Framework for Health and Community Care (Second edition, 2006) and WORLD MEDICAL ASSOCIATION DECLARATION OF HELSINKI Ethical Principles for Medical Research Involving Human Subjects 1964 (as amended).

Glasgow Clinical Trials Unit

CONTACTS

Chief Investigator

Dr Jennifer Logue MBChB MRCP MD FRCPath
CSO Clinician Scientist
Clinical Senior Lecturer and Honorary Consultant in Metabolic Medicine
Institute of Cardiovascular and Medical Sciences University of Glasgow
BHF Glasgow Cardiovascular Research Centre University of Glasgow
126 University Place, Room 208
University of Glasgow
Glasgow
G12 8TA

Tel: 0141 330 2569
Fax: 0141 330 6955
E-mail: Jennifer.Logue@Glasgow.ac.uk

Co-investigator

Professor Naveed Sattar
Professor of Metabolic Medicine
Cardiovascular and Medical Sciences
BHF Glasgow Cardiovascular Research Centre
RC214 Level C2
126 University Place, Room 208
University of Glasgow
Glasgow
G12 8TA
Tel: 0141 330 3419
Fax: 0141 330 6972
E-mail: Naveed.Sattar@glasgow.ac.uk

Dr Julie Bruce
Principal Research Fellow
Division of Health Sciences,
Warwick Medical School,
The University of Warwick,
Coventry,
CV4 7AL
Tel: 024 7615 1128
E-mail: julie.bruce@warwick.ac.uk

Mr Duff Bruce
Bariatric Surgeon
Ward 33
Aberdeen Royal Infirmary
Foresterhill
Aberdeen
AB25 2ZN
Tel: 01224 551275
Fax: 01224 443212
E-mail: duff.bruce@nhs.net

Glasgow Clinical Trials Unit

Dr Robert Lindsay
Reader Diabetes and Endocrinology
Cardiovascular and Medical Sciences
RC337 Level C3
BHF Glasgow Cardiovascular Research Centre
126 University Place, Room 208
University of Glasgow
Glasgow
G12 8TA
Tel: 0141 211 2502
Fax: 0141 330 6997
E-mail: Robert.Lindsay@glasgow.ac.uk

Professor Ian Ford
Professor of Biostatistics
R1122B Level 11
Robertson Centre for Biostatistics
Boyd Orr Building
Glasgow
G12 8QQ
Tel: 0141 330 4048
Fax: 0141 330 5094
E-mail: Ian.Ford@glasgow.ac.uk

Professor Andrew Briggs
Chair in Health Economics
(Health Economics and Health Technology Assessment) HEHTA
Institute of Health & Wellbeing
1 Lilybank Gardens
Glasgow
G12 8RZ
Tel: 0141 330 5017
E-mail: Andrew.Briggs@glasgow.ac.uk

Professor Mike Lean
Professor in Human Nutrition
R408 Level 4
Human Nutrition
Glasgow Royal Infirmary
84 Castle Street Glasgow
G40SF
Tel: 0141 211 4686
Fax: 0141 211 4844
E-mail: Mike.Lean@glasgow.ac.uk

Jane Munro
Research Lead
Cardiovascular and Medical Sciences
BHF Glasgow Cardiovascular Research Centre University of Glasgow
126 University Place, Room 208
University of Glasgow
Glasgow

Glasgow Clinical Trials Unit

G12 8TA
Tel: 0141 330 1670
E-mail: Jane.Munro@glasgow.ac.uk

Project Research Assistant

Mrs. Joanne O'Donnell
SCOTS Research Assistant
Cardiovascular and Medical Sciences
BHF Glasgow Cardiovascular Research Centre University of Glasgow
126 University Place, Room 208
University of Glasgow
Glasgow
G12 8TA
Tel: 0141 330 1670
Fax: 0141 330 5094
E-mail: Joanne.O'Donnell@glasgow.ac.uk

Study Statistician

Professor Ian Ford
Professor of Biostatistics
R1122B Level 11
Robertson Centre for Biostatistics
Boyd Orr Building
Glasgow
G12 8QQ
Tel: 0141 330 4048
Fax: 0141 330 5094
E-mail: Ian.Ford@glasgow.ac.uk

Data Centre

Alex McConnachie
Assistant Director of Biostatistics (Robertson Centre)
R1122E Level 11
Robertson Centre for Biostatistics
Boyd Orr Building
Glasgow
G12 8QQ
Tel: 01413304744
Fax: 0141 330 5094
E-mail: Alex.McConnachie@glasgow.ac.uk

Chair of the Study Steering Committee

Mr Richard Welbourn
Bariatric Surgeon
Musgrove Park Hospital
Taunton
TA1 5DA
Tel: 01823 343 565
Fax: 01823 353560
E-mail: richardwelbourn@gmail.com

Glasgow Clinical Trials Unit

Sponsor - NHS Greater Glasgow and Clyde R&D

Sponsor's representative

Dr Maureen Travers
Research Co-ordinator
NHS Greater Glasgow & Clyde
Research and Development Management Office
Tennent Institute
38 Church Street
Western Infirmary
Glasgow G11 6NT
Tel: 0141 211 6389
E-mail: maureen.travers@ggc.scot.nhs.uk

Funding Body

National Institute for Health Research (NIHR)

Funding Body Representative

Mrs Lesley Dodd
Programme Manager
NIHR Co-ordinating Centre for Health Technology Assessment (NETSCC, HTA)
University of Southampton
Alpha House
Enterprise Road
Southampton Science Park
Chilworth
Southampton
SO16 7NS
Tel: 02380 597558
Fax: 02380 595639
E-mail: L.Dodd@southampton.ac.uk

Mackenzie RM, *et al. BMJ Open* 2022; 11:e046441. doi: 10.1136/bmjopen-2020-046441

Glasgow Clinical Trials Unit

PROTOCOL APPROVAL

Surgical Obesity Treatment Study (SCOTS)

Chief Investigator

Dr Jennifer Logue
MBChB MRCP MD FRCPath CSO Clinician Scientist
Clinical Senior Lecturer and Honorary Consultant in Metabolic Medicine
Institute of Cardiovascular and Medical Sciences BHF
Glasgow Cardiovascular Research Centre
University of Glasgow
126 University Place, Room 208
University of Glasgow
Glasgow
G12 8QQ

Signature: 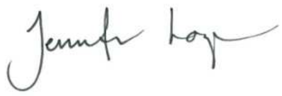

Date: Tuesday, 03 August 2021

Sponsor's representative

Dr Maureen Travers
Research Co-ordinator
NHS Greater Glasgow & Clyde Research and Development Management Office
Tennent Institute
38 Church Street
Western Infirmary
Glasgow
G11 6NT

Signature:

Date:

TABLE OF CONTENTS

CONTACTS 3

PROTOCOL APPROVAL 7

TABLE OF CONTENTS 8

ABBREVIATIONS 11

STUDY SYNOPSIS 14

GLOSSARY OF TERMS 16

STUDY FLOW CHART 19

SCHEDULE OF ASSESSMENTS 20

1 INTRODUCTION 23

1.1 Background 23

1.2 Rationale 23

1.2.1 Definition of bariatric surgery 24

1.2.2 Clinical Outcomes of weight loss surgery 24

1.3 Study hypothesis 25

2 STUDY OBJECTIVES 25

2.1 Primary Objective 25

2.2 Secondary Objectives 25

2.3 Primary Outcome 26

2.4 Secondary Outcomes 26

3 STUDY DESIGN 27

3.1 Overview of study design 27

3.1.1 Part 1 Record Linkage 29

3.1.2 Part 2 Patient Reported Outcomes (PROMS) Questionnaire 29

3.1.3 Part 3: Registration for a database of bariatric patients for future research 29

3.1.4 Service user involvement 30

3.2 Study Population 30

3.3 Reference populations 30
3.4 Inclusion criteria 31
3.5 Exclusion criteria 31
3.6 Identification of participants and consent 31
3.7 Withdrawal of subjects 32
4 STUDY PROCEDURES 33
4.1 Duration of participation 36
4.2 Study schedule 37
4.2.1 Start of Study - Surgical team registration 37
4.2.2 Visit 1 Routine care invitation visit 38
4.2.3 Visit 2 Recruitment visit 38
4.2.4 SCOTS study team contact patient 42
4.2.5 Visit 3 Weight loss operation 42
4.2.6 Visit 4 -Post operative follow up 42
4.2.7 Visit 5 - Re-operation (If applicable) 43
5 STATISTICS AND DATA ANALYSIS 44
5.1 Statistical Analysis Plan 44
5.2 Software for statistical analysis 44
5.3 Sample size 44
5.4 Management and delivery 46
6 STUDY CLOSURE 47
7 Data Handling 47
7.1.1 Surgeon registration data 47
7.1.2 Patient recruitment data 47
7.1.3 Surgical data collection 48
7.1.4 Patient Reported Outcomes 48
7.1.5 Routine clinical information 48

7.1.6 Re-operation ... 48
7.1.7 Record Retention ... 49
7.1.8 Access to data ... 49
8 SCOTS STUDY MANAGEMENT ... 50
8.1 Study Sponsor ... 50
8.2 SCOTS Study Team ... 50
8.3 SCOTS Principal investigators ... 50
8.4 Routine management of study: SCOTS Management Group (SMG) ... 50
8.5 SCOTS Steering Committee (SSC) ... 51
9 STUDY MONITORING/AUDITING ... 52
10 PROTOCOL AMENDMENTS ... 52
11 ETHICAL CONSIDERATIONS ... 52
11.1 Ethical conduct of the study ... 52
11.2 Informed consent ... 52
12 INSURANCE AND INDEMNITY ... 53
13 FUNDING ... 53
14 ANNUAL REPORTS ... 53
15 DISSEMINATION OF FINDINGS ... 54
16 REFERENCES ... 55
17 Appendix E: Summary of Patient Reported Outcome Questions ... 59

ABBREVIATIONS

BHF GCRC	*British Heart Foundation, Glasgow Cardiovascular Research Centre*
BMI	*Body Mass Index*
BP	*Blood Pressure*
BPD-DS	*BilioPancreatic Diversion with Duodenal Switch*
CHD	*Coronary Heart Disease*
CHI	*Community Health Index*
CHF	*Congestive Heart Failure*
CI	*Chief Investigator*
CKD	*Chronic Kidney Disease*
CNORIS	*Clinical Negligence and Other Risks Indemnity Scheme*
CRF	*Case Report Form*
CSO	*Chief Scientist Office*
CTU	*Clinical Trials Unit*
CVD	*Cardiovascular Disease*
DM	*Diabetes Mellitus*
DM(E)C	*Data Monitoring (Ethics) Committee*
DOH	*Department of Health*
DS	*Duodenal Switch*
e-CRF	*Electronic Case Report Form*
EQ-5D	*EuroQol -5D*
GAD-7	*Generalised Anxiety Disorder – 7*
GCP	*Good Clinical Practice*
GCP	*Good Clinical Practice*
GCTU	*Glasgow Clinical Trials Unit*
GP	*General Practitioner*

Glasgow Clinical Trials Unit

GRDS	*Gastric Reduction Duodenal Switch*
GROS	*General Register Office for Scotland*
HbA1c	*Glycated Haemoglobin A1c*
HDL	*High Density Lipoprotein*
HR-QL	*Health Related- Quality of Life*
HTA	*Health Technology Assessment*
ICMS	*Institute of Cardiovascular and Medicinal Sciences*
IPAQ	*International Physical Activity Questionnaire*
IPSS	*International Prostate Symptom Score*
IRAS	*Integrated Research Application System*
ISD	*Information and Services Division (NHS)*
ISRCTN	*International Standard Randomised Controlled Trial Number*
IWQOL-Lite	*Impact of Weight on Quality of Life Lite Questionnaire*
LOT	*Life orientation Test*
MG	*Management Group*
MGM	*Management Group Meeting*
MI	*Myocardial Infarction*
NBSR	*National Bariatric Surgery Register*
NHS	*National Health Service*
NICE	*National Institute for Health and Clinical Excellence*
NIHR	*National Institute for Health Research*
NIHR CSP	*National Institute for Health Research Coordinated System for Gaining NHS Permission*
PHQ-9	*Patient Health Questionnaire - 9*
PI	*Principal Investigator*
PROMS	*Patient Reported Outcome Measures*
QALY	*Quality Adjusted Life Year*

Mackenzie RM, *et al. BMJ Open* 2022; 11:e046441. doi: 10.1136/bmjopen-2020-046441

Glasgow Clinical Trials Unit

QOL	*Quality Of Life*
R&D	*Research and Development*
RCB	*Robertson Centre for Biostatistics*
REC	*Research Ethics Committee*
SCOTS	*SurgiCal Obesity Treatment Study*
SCR	*Scottish Cancer Registry*
SCI-DC	*Scottish Care Information – Diabetes Collaboration*
SDRN	*Scottish Diabetes Research Network*
SDRN EG	*Scottish Diabetes Research Network Epidemiology Group*
SF-12	*Short Form -12*
SIGN	*Scottish Intercollegiate Guidelines Network*
SMG	*SCOTS Management Group*
SMR	*Scottish Morbidity Record*
SOP	*Standard Operating Procedure*
SSC	*SCOTS Steering Committee*
SSI	*Surgical Site Infection*

Mackenzie RM, *et al. BMJ Open* 2022; 11:e046441. doi: 10.1136/bmjopen-2020-046441

Glasgow Clinical Trials Unit

STUDY SYNOPSIS

Title of Study:	**Surgical Obesity Treatment Study**; SCOTS
Study Centre:	University of Glasgow
Duration of Study:	03/01/2012 – 30/06/2026
Primary Objective:	The SurgiCal Obesity Treatment Study (SCOTS) will establish the clinical outcomes and adverse events of different bariatric surgical procedures (taking into consideration the different care pathways), their impact on quality of life and nutritional status, and the effect on co-morbidities in a cohort of 450 patients.
Secondary Objectives:	1. The physical and mental health and social burden of severe obesity.<br>2. Incidence of acute and chronic postoperative complications. Acute complications, defined as up to three months post-surgery, will include surgical site infection; chronic complications will include revisional surgery, plastic surgery and chronic pain, for different bariatric surgical procedures<br>3. The effect of surgical experience and the pre and post-operative care pathway on complication rates and weight loss, for different bariatric surgical procedures.<br>4. The effect of the pre-surgical pathway and criteria on bariatric surgery patient selection.<br>5. Change in health related quality of life, anxiety and depression over time pre- and post-operatively for a mean of 3 years from date of bariatric surgery.<br>6. The weight status pre and post-operatively for 3 years after bariatric surgery.<br>7. The glycaemic control, lipids, blood pressure, medication prescription, and rate of diabetes complications (microalbuminuria and renal disease, and retinopathy) in those that have pre-existing diabetes or develop diabetes for 3 years follow up since bariatric surgery.<br>8. Changes in socio-economic factors (employment, benefit receipt, sick leave, and health care use) for 3 years since bariatric surgery.
Primary Endpoint:	Change in weight from pre-bariatric surgery weight

Glasgow Clinical Trials Unit

Rationale:	The efficacy of bariatric surgery for large scale, long term weight loss is well established. However, many questions remain over the medium term benefits of that weight loss for health, particularly when accounting for the potential complications of surgery.
Methodology:	Observational cohort study/ registry
Sample Size:	450
Screening:	All new bariatric surgery patients
Registration/Randomisation:	Not applicable
Main Inclusion Criteria:	• Patients 16 years or over undergoing their first bariatric surgery in NHS hospitals or private practice in Scotland.<br>• Capacity to consent<br>• Resident in Scotland<br>• Written informed consent
Main Exclusion Criteria:	• Does not meet inclusion criteria<br>• Patients who have had previous weight loss surgery (at time of potential recruitment are undergoing a repeat procedure)<br>• No written informed consent<br>• Patients under 16 years<br>• Patients who do not have capacity to consent for themselves<br>• Weight loss surgery performed at a site outside Scotland<br>• Patients who live outside Scotland<br>• Patients who do not have strong enough English language skills to understand the patient information leaflet without assistance will be excluded from Part 2 of the study.
Product, Dose, Modes of Administration:	Web based or paper questionnaire to be completed<br>1 month pre-surgery<br>2 years post surgery<br>3 years post surgery
Duration of Treatment:	Observational study so protocol will be applied until a participant has been in study for 3 years or until December 2020 whichever comes first. If funding available record linkage will continue throughout patient’s life.
Statistical Analysis:	Detailed statistical analysis plans will be developed by the Robertson Centre for Biostatistics to address each of the objectives described above.

Mackenzie RM, *et al. BMJ Open* 2022; 11:e046441. doi: 10.1136/bmjopen-2020-046441

GLOSSARY OF TERMS

Bariatric Surgical Procedures: *A bariatric surgical procedure is defined as a surgical intervention which has the primary purpose of large scale weight loss in a patient who is obese. Bariatric Surgical procedures eligible for inclusion in SCOTS are:* ***duodenal switch, gastric banding, gastric bypass*** *and* ***sleeve gastrectomy****. The inclusion of emerging techniques in this definition will be decided by the steering committee.*

Biliopancreatic diversion with duodenal switch (BPD-DS): *see* ***Duodenal Switch****:*

Body mass index (BMI) *is a simple index of weight-for-height that is commonly used to classify* ***overweight*** *and* ***obesity*** *in adults. It is defined as a person's weight in kilograms divided by the square of his height in meters (kg/m^2). BMI provides the most useful population-level measure of overweight and obesity as it is the same for both sexes and for all ages of adults. However, it should be considered a rough guide because it may not correspond to the same degree of fatness in different individuals.*

Co-morbidity: *is either the presence of one or more disorders (or diseases) in addition to a primary disease or disorder, or the effect of such additional disorders or diseases.*

Duodenal Switch: *(DS) is a weight loss surgery procedure that is composed of a restrictive and a malbsorbative aspect. The restrictive portion of the surgery involves removing approximately 70% of the stomach along the greater curvature. The malabsorptive portion of the surgery reroutes a lengthy portion of the small intestine, creating two separate pathways and one common channel. Duodenal switch (DS) is also known as biliopancreatic diversion with duodenal switch (BPD-DS) or gastric reduction duodenal switch (GRDS).*

Endoluminal barrier: *is a liner that fits inside a section of the intestine, creating a barrier between the intestinal wall and the food you eat. It helps patients to lose weight by preventing food from coming into contact with the intestinal wall thus delaying digestion, which will alter the activation of hormonal signals that originate in the intestine.*

Gastric balloon: *An inflatable device implanted in the stomach as an adjunct to therapy of morbid obesity. For the purposes of the SCOTS study a balloon treatment is not considered to be bariatric surgery. Use of a balloon before or after surgery will be recorded as a pre-operative treatment but a balloon alone would not meet the criteria for inclusion in SCOTS.*

Gastric band: *adjustable gastric banding surgery, is a restrictive weight loss surgery that works by decreasing the amount of food that is able to be consumed before feeling full. During gastric banding surgery, a firm silicone band with an inner inflatable ring is placed around the stomach to slow the passage of food from the upper portion of the stomach to the lower portion*

Gastric bypass: *a surgical bypass operation that typically involves reducing the size of the stomach and reconnecting the smaller stomach to bypass the first portion of the small*

intestine so as to restrict food intake and reduce caloric absorption in cases of severe obesity. This includes Gastric bypass, Roux en-Y (proximal), Gastric bypass, Roux en-Y (distal) and Mini-gastric bypass" (MGB)

Gastric reduction duodenal switch (GRDS): see duodenal switch

Glycaemic control*: is a medical term referring to the typical levels of blood sugar (glucose) in a person with diabetes mellitus. Much evidence suggests that many of the long-term complications of diabetes, especially the microvascular complications, result from many years of hyperglycemia (elevated levels of glucose in the blood).*

Hemoglobin A1c (HbA1c): *Blood sugar levels fluctuate throughout the day and glucose records are imperfect indicators of these changes, the percentage of haemoglobin which is glycosylated is used as a proxy measure of long-term glycemic control in research trials and clinical care of people with diabetes. This test, the hemoglobin A1c or glycosylated hemoglobin reflects average glucoses over the preceding 2–3 months. From 1 October 2011 the way in which HbA1c results are expressed in the UK has changed. Results are now reported in the IFCC reference method of mmol/L, rather than the DCCT units as a percentage. The equivalent of the current HbA1c targets of 6.5% and 7.5% are therefore 48mmol/mol and 58mmol/mol in the new units for diabetic patients, with the non-diabetic reference range of 4.0% to 6.0% being 20mmol/L to 42mmol/L.*

HDL cholesterol: *High density lipoprotein cholesterol. Lipoproteins, which are combinations of fats (lipids) and proteins, are the form in which lipids are transported in the blood. HDLs transport cholesterol from the tissues of the body to the liver, so the cholesterol can be eliminated in the bile. HDL cholesterol is therefore considered the 'good' cholesterol: The higher the HDL cholesterol level, the lower the risk of coronary artery disease. The average man has an HDL cholesterol level of 40 to 50 mg/dL. In the average woman, HDL levels range from 50 to 60 mg/dL.*

Health Technology Assessment*: is a multidisciplinary process that summarises information about the medical, social, economic and ethical issues related to the use of a health technology in a systematic, transparent, unbiased, robust manner. Its aim is to inform the formulation of safe, effective, health policies that are patient focused and seek to achieve best value.*

Hypertriglycerideamia*: denotes high (''hyper-'') blood levels (''-aemia'') of triglycerides, the most abundant fatty molecule in most organisms.*

Laparoscopic gastric banding*: Lap Band surgery, or adjustable gastric banding surgery, is a restrictive weight loss surgery that works by decreasing the amount of food that is able to be consumed before feeling full. During gastric banding surgery, a firm silicone band with an inner inflatable ring is placed around the stomach to slow the passage of food from the upper portion of the stomach to the lower portion. Most surgeries are performed laparoscopically, which allows the surgeon to work using long instruments placed in the body via incisions a few centimetres long.*

Mackenzie RM, *et al. BMJ Open* 2022; 11:e046441. doi: 10.1136/bmjopen-2020-046441

***Laparoscopy**: is a type of surgical procedure that allows a surgeon to access the inside of the abdomen and pelvis without having to make large incisions in the skin. It is also known as keyhole surgery. Laparoscopy is minimally invasive. This is made possible with an instrument called a laparoscope.*

***Morbidity**: Morbidity is an incidence of ill health.*

***Mortality**: is incidence of death in a population.*

***NHS patient**: A patient who receives free healthcare through the National Health Service*

***Nutritional Status**: The state of a person's health in terms of the nutrients stored in his or her body.*

***Obesity**: Obesity is a term used to describe somebody who is very overweight with a high degree of body fat. There are a number of ways a person's weight can be assessed. The most widely used method is the body mass index.*

***Overweight**: a BMI of between 25 and 29, would be considered overweight*

***Obese**: a BMI of 30 or above would be considered obese*

***Private sector**: healthcare and medicine provided by entities other than the government and paid for by the patient or a private insurance company.*

***Prospective cohort study**: a study designed to determine the relationship between a condition and a characteristic shared by some members of a group. The population selected is healthy at the beginning of the study. Members share a particular characteristic. The study follows the population groups over a long period, noting the rate at which a condition occurs in the population.*

***Revisional surgery**: a bariatric surgical procedure on a patient who has already had bariatric surgery*

***Roux-en-Y gastric bypass:** a small part of the stomach is used to create a new stomach pouch, roughly the size of an egg. The smaller stomach is connected directly to the middle portion of the small intestine (jejunum), bypassing the rest of the stomach and the upper portion of the small intestine (duodenum).*

***Sleeve gastrectomy**: is a surgical weight-loss procedure in which the stomach is reduced to about 25% of its original size, by surgical removal of a large portion of the stomach, following the major curve. The open edges are then attached together (often with surgical staples) to form a sleeve or tube with a banana shape. The procedure permanently reduces the size of the stomach. The procedure is performed laparoscopically and is not reversible.*

Mackenzie RM, *et al. BMJ Open* 2022; 11:e046441. doi: 10.1136/bmjopen-2020-046441

Glasgow Clinical Trials Unit

STUDY FLOW CHART

FIGURE 0-1 THIS STUDY FLOW CHART SUMMARISES THE PATIENT JOURNEY TRHOUGHOUT THE STUDY

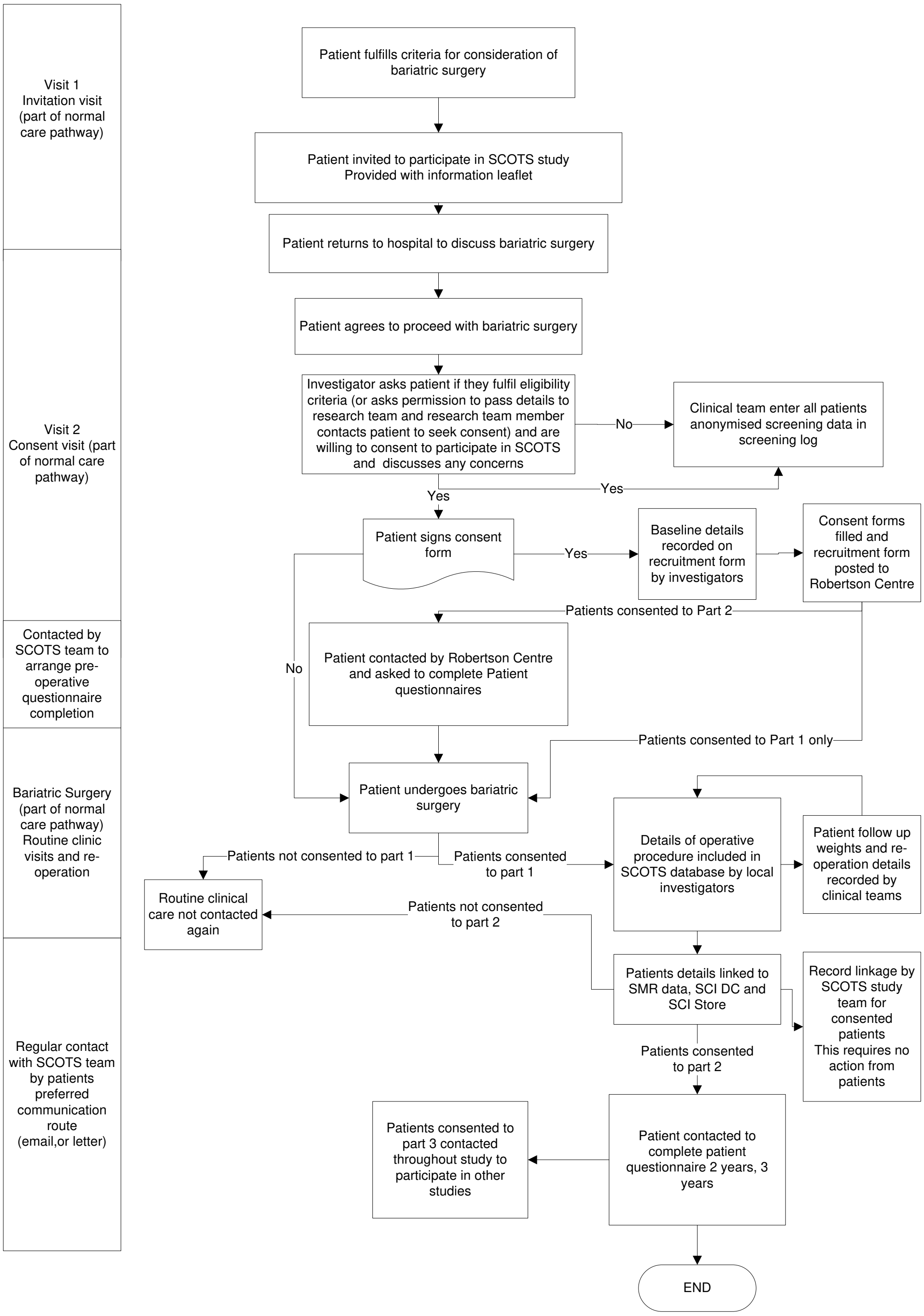

SCHEDULE OF ASSESSMENTS

TABLE 0-1: THIS SCHEDULE SHOWS EACH EVENT THAT INVOLVES THE PATIENT AND STUDY TEAM.

	Pre-surgery					Post surgery SCOTS Questionnaires	
Study Procedure	Pre-surgery Visit 1 1 year to six weeks before surgery	Pre-surgery Visit 2 1 year to six weeks before surgery	Pre-surgery Contact1	Pre-surgery Contact2	Surgery T0	2yr	3yr
Patients agree to proceed to bariatric surgery with clinical team	√						
Review Inclusion/Exclusion Criteria	√[a]						
Patients provided with patient information sheet	√						

Glasgow Clinical Trials Unit

Patient data entered onto screening log for SCOTS		√					
Patient return for pre-surgery clinic		√					
Clinical team ask permission from patients to pass details to research team who will contact patient (if not recruiting in local site)		(√)					
Obtain Informed Consent to participate in SCOTS study		√					
Patient baseline and contact details entered on registration form and sent to Robertson Centre for Biostatistics		√					
Consent form passed filed in Site File, copy given to patient and copy sent to RCB		√					
Contacted by SCOTS team to ask to complete pre-surgery questionnaire			√				

Glasgow Clinical Trials Unit

Patient completes pre-surgery questionnaire				√			
Patient undergoes bariatric surgery					√		
Clinical teams record operative details for patient post surgery					√		
Clinical teams record weight and re-operation details						√	√
Patients contacted to remind them to complete post surgery questionnaire						√	√
Part 3 consented patients may be invited to participate in other studies	Ongoing throughout study						
Record linkage to SCI-Store, SCI – DC, SMR (ISD), GRO throughout study	Ongoing throughout study, no interaction with patient unless to clarify if admission was a complication of bariatric surgery.						

1 INTRODUCTION

1.1 Background

SCOTS is a prospective cohort study commissioned by the Department of Health (DoH) National Institute of Health Research (NIHR) which will follow bariatric surgery patients for 3 years after their surgery. The NIHR commissioning brief for this study stated: “Obesity is a growing problem in the United Kingdom, with a growing number of people among the morbidly obese. Concomitantly, there is an increase in requests for bariatric surgery but insufficient evidence of long term effectiveness and safety of these procedures. There are existing registries of bariatric surgery, but these suffer from problems of collected data, completeness of follow- up, or data availability for secondary analysis. There is a need for a long term study of bariatric surgery, so that the outcomes and complications of different procedures, their impact on quality of life and nutritional status, and the effect on co-morbidities can be monitored in both the short and the long term.” A recent Health Technology Assessment (HTA) (1) concluded that “Bariatric surgery is a more clinically effective intervention for treating obesity compared to non-surgical options” however they qualified this with “Uncertainties remain over the long-term outcomes of bariatric surgical interventions, particularly their impact on weight-related health problems and patient quality of life. Further studies will be required”. This study was awarded NIHR HTA funding to address some of the uncertainties around the clinical effectiveness of bariatric surgery in the longer term. The original plan included a 10 year follow up period, however, due to unforeseen recruitment issues (reductions in both NHS & private surgical numbers) the study & statistical plans were reviewed. Following discussions with the NIHR it was agreed that many objectives could still be met from the number of patients currently recruited over a shorter period of follow up (3 years). The protocol has been updated to reflect these changes. Clearly some patients will have completed the 1 month, 6 month and 1 year post-operative questionnaire already. Where appropriate, these data will be used to supplement the year 2 and 3 questionnaires and the health record data in order to deliver the study objectives.

1.2 Rationale

The efficacy of bariatric surgery for large scale, long term weight loss is well established (2). However, many questions remain over the medium term benefits of that weight loss for health, particularly when accounting for the potential complications of surgery. Given that the proportions of the population who are obese is increasingly rapidly, further information is required as to who benefits most from bariatric surgery.

1.2.1 Definition of bariatric surgery

A bariatric surgical procedure is defined as a surgical intervention which has the primary purpose of large scale weight loss in a patient who is obese. Currently that includes gastric bypass, sleeve gastrectomy and laparoscopic gastric banding. The inclusion of emerging techniques in this definition will be decided by the SCOTS Steering Committee (SCC).

1.2.2 Clinical Outcomes of weight loss surgery

There are some pre-existing data on clinical outcomes with 10 year mortality rates being the best known. Overall mortality was found to be 29-40% lower in patients receiving bariatric surgery compared to Body Mass Index (BMI)-matched subjects not receiving surgery (3;4). Although impressive, these figures are from studies which initially recruited in the 1980s and early 1990s and further work is required to update the mortality rate in the current population, now that primary prevention measures for cardiovascular disease, such as statins and ACE-inhibitors, have lowered the risk of premature mortality in at risk subjects, including those who are obese or have diabetes.

Diabetes improvement post bariatric surgery has also been of interest. Dixon et al conducted a randomised controlled trial of laparoscopic gastric banding in patients with newly diagnosed type 2 diabetes resulted in a 73% remission rate after 2 years (5). Similar results have been shown for gastric bypass using meta-analysis of heterogeneous, mainly retrospective, cohort studies (6). Whether remission is maintained beyond the first few years post surgery is unknown. However, the main outcomes of interest are the longer term effects on the development of diabetes complications such as retinopathy, nephropathy and cardiovascular disease and those are not established.

The effect of bariatric surgery on cardiovascular outcomes has not been ascertained. It is known that HDL cholesterol is less likely to be low and hypertriglycerideamia is less common after surgery (7), as expected by a procedure which will reduce intra-abdominal adiposity and thus insulin resistance, but a reduction in cardiovascular events has not been shown. Hypertension, which would be presumed to decrease, in fact shows no difference 8 years post surgery, once the initial antihypertensive effects of acute weight loss in the first few years post-surgery are accounted for (8). Many have used cardiovascular risk factors measured during the acute weight loss phase, applied a population based cardiovascular risk score and stated a reduction in cardiovascular risk; this is of course a flawed use of such a score based on temporarily reduced cardiovascular risk factors. So, while the general population and the majority of physicians may assume that weight loss with bariatric surgery reduces the risk of a cardiovascular event, this is currently unknown.

What is established is that health related quality of life (HR-QoL) is increased compared to pre-operative levels (9). When patients who decide to proceed to surgery are compared to those who opt for non-surgical treatment only, the baseline HR-QoL is far lower in those

who select surgery, showing at least a perception of reduced quality of life in these individuals (10). To what extent the improvements seen in HR-QoL correlate with weight loss, complications and clinical outcomes is not known.

The complication rates of bariatric surgery are poorly reported in the literature, leading to polarised views on their true incidence. Mortality rates in the first year post surgery have been reported as being similar to those of BMI-matched controls (11); however, the same results may not be found today due to the decreased mortality rates as a result of primary preventative. There is little report of specific wound complications and re-operation rates, with a general belief that the low rates reported by the few studies releasing this data would not be replicated in routine practice.

1.3 Study hypothesis

This study is a registry of bariatric surgery and is therefore the study design is based on objectives but not hypotheses.

2 STUDY OBJECTIVES

SCOTS is a prospective observational cohort study of 450 patients undergoing bariatric surgery in Scotland which will be used to assess medium term outcomes and complications of bariatric surgery. This is in order to identify risks and complications of bariatric surgery and develop an evidence base to support clinical teams in building a patient centred, safe, effective and efficient bariatric surgery service.

2.1 Primary Objective

The SurgiCal Obesity Treatment Study (SCOTS) will establish the clinical outcomes and adverse events of different bariatric surgical procedures (taking in to consideration different care pathways), their impact on quality of life and nutritional status, and the effect on co-morbidities in a cohort of 450 patients.

2.2 Secondary Objectives

To establish in a cohort of obese patients who are undergoing bariatric surgery:

1. The physical and mental health and social burden of severe obesity.
2. Incidence of acute and chronic postoperative complications. Acute complications, defined as up to three months post-surgery, will include surgical site infection; chronic complications will include revisional surgery, plastic surgery and chronic pain, for different bariatric surgical procedures.

3. The effect of surgical experience and the pre and post-operative care pathway on complication rates and weight loss, for different bariatric surgical procedures.
4. The effect of the pre-surgical pathway and criteria on bariatric surgery patient selection. Change in health related quality of life, anxiety and depression over time pre- and post-operatively for a mean of 3 years from date of bariatric surgery.
5. The weight status pre and post-operatively for 3 years after bariatric surgery.
6. The glycaemic control, lipids, blood pressure, medication prescription, and rate of diabetes complications (microalbuminuria and renal disease, and retinopathy) in those that have pre-existing diabetes or develop diabetes for 3 years follow up since bariatric surgery.
7. Changes in socio-economic factors (employment, benefit receipt, sick leave, and health care use) for 3 years since bariatric surgery.

All the above objectives are due to be achieved by December 2020 (Start Jan 2012, 2 years development, 3 years recruitment finishing March 2017, mean 3 years post-operative follow-up finishing December 2020).

2.3 Primary Outcome

Change in weight from pre-bariatric surgery weight.

2.4 Secondary Outcomes

1. Incidence of physical and psychological comorbidity in a population seeking bariatric surgery

In the 3 years following bariatric surgery:

2. Change in weight/BMI from pre-bariatric surgery weight
3. Rate of incident type 2 diabetes
4. Change in incidence of depression and anxiety compared to baseline level pre-operatively
5. Incidence of complications immediately post-operatively and the need for readmission for revisional procedures
6. Change in health-related quality of life compared to baseline level pre-operatively
7. Change in glycaemic control, cardiovascular risk factors, CKD, retinopathy and medications prescribed in patients with diabetes compared to equally obese patient with diabetes who did not have bariatric surgery
8. Cost of the procedure and follow-up

Outcomes, including their source and frequency of measurement, are summarised in table 1.

3 STUDY DESIGN

3.1 Overview of study design

This 3 year prospective cohort study will collect data on the medium term outcomes of bariatric surgery in a minimum of 400 individuals. The study will be performed according to the Research Governance Framework for Health and Community Care (Second edition, 2006) (12). The information required to meet the objectives will be collected by a combination of sources including: bariatric surgery specific information entered by clinical teams; data linkage to routine clinical data and patient reported outcome measure questionnaires (PROMS). The outcomes and the data source are described in detail in Table 3-1.

TABLE 3-1: PRIMARY AND SECONDARY OBJECTIVES OF SCOTS DESCRIBING SOURCES OF OUTCOME DATA

	Outcome	Data source	Frequency
1	Incidence of physical and psychological comorbidity in a population seeking bariatric surgery.	SCI-DC Scottish National dataset, eDRIS at National Service Scotland, Patient reported questionnaires.	Continuous throughout study
2.1	Change in weight/BMI from pre-bariatric surgery	Clinical teams	At recruitment, annual check up
2.2	Rate of incidence of type 2 diabetes	SCI-DC Scottish National dataset	Continuous throughout study
2.3	Change in incidence of depression and anxiety compared to baseline levels pre-operatively	eDRIS at National Service Scotland, Patient reported questionnaires	Pre-operatively, ,2,3 years post operatively
2.4	Incidence of complications immediately post-operatively and the need for revisional procedures	Patient reported questionnaires, surgical teams	Pre-operatively, 2, 3 years post operatively, at time of re-operation.
2.5	Change in health related quality of life compared to baseline	Patient reported questionnaires	Pre-operatively, 2,3years post operatively
2.6	Change in glycaemic control, cardiovascular risk factors, chronic kidney disease (CKD), retinopathy, medications in patients with	SCI-DC Scottish National dataset	Pre-operatively, 2, 3 years post operatively, at time of re-operation.

	diabetes compared with non-obese cohort		
2.7	Cost of the procedure and follow-up (to inform cost effectiveness analysis)	Clinical teams, e-prescribing record linkage, patient reported outcomes	At time of patients operation, continuous linkage throughout the study

This protocol describes the objectives of the study, the sources of data collection for each objective, and the input required from patients and clinical teams throughout the study. A data collection portal will be developed for the study with secure logins for clinical teams and participating patients. A detailed user manual will be developed for the operation of this system will be produced by the Robertson Centre for Biostatistics - University of Glasgow.

A SCOTS web based portal will be developed which will present information on the study to the public and other researchers. The portal will contain secure logins to the Robertson Centre databases which will allow clinical teams to enter data. Clinical teams will be asked to register for a user ID and password. These will be issued once the team have undergone the necessary training for their delegated role. Everyone who has a user ID and login will be a member of the SCOTS investigators group.

The SCOTS portal will include an area which will allow patients who have agreed to complete the patient questionnaires to login securely and complete the patient reported outcome questionnaires. If patients prefer to complete the questionnaires on paper the data will be entered by staff at the Robertson Centre into the database. Patients will be kept up to date with progress of the study via updates on the study website. Patients will be sent reminders through their preferred route of communication to let them know when they are due to complete a questionnaire.

Patient identifiable information will be stored separately from the clinical outcome data. The information will be linked using a database code. Within the Robertson Centre individuals who can access patient information to send reminder letters will not have access to any clinical information. Any clinical information to be analysed will have all patient contact details remove. Production of any reports will follow the NHS Information and Statistics division (ISD) Statistical Disclosure Control protocol (13) to ensure that patient confidentially is maintained, (this is a requirement of the record linkage arrangement with ISD).

SCOTS has three distinct parts. All adult patients who are having weight loss surgery in Scotland will be invited to participate in three parts of the study. These parts are described in detail below.

3.1.1 Part 1 Record Linkage

The study will collect data by record linkage to patients' clinical outcomes. Information will be recorded by the clinical team on the operation they have had. They will be followed using their medical records until at least December 2020 and if funding is available for the rest of their life. This will include all hospital admissions, blood test results, diagnostic test results, diabetes complications, medications they are prescribed, pregnancy, psychiatry and mortality. This part of the study will observe patients care. The study will not alter their planned care in any way. No additional tests or treatments will be given to patients who consent to be part of the study. (This will fulfil the primary objective and objectives 1, 2, 3 and 4). If patients do not consent to participate in this part of the study they will not be asked to participate in Part 2 or Part 3.

Each record linkage requires approvals to access data including a favourable ethical opinion. These will be sought by the SCOTS Project team. A separate procedure for each data linkage will be developed by the Robertson Centre for Biostatistics. Patient identifiable information is required to identify study participants. The information required is patient CHI number, family name, given name, patient date of birth, gender, postcode, consent data and unique study identifier.

3.1.2 Part 2 Patient Reported Outcomes (PROMS) Questionnaire

We will ask patients if they are willing to complete a questionnaire which will include questions on quality of life, overall health and fitness, diet, and anxiety and depression, healthcare utilisation and ability to work. They will also be asked about problems they may have experienced since their surgery such as pain and surgical site infection post discharge.). Where patients are not completing PROMS and there is no reliable clinical weight record, we will write after 3 years requesting completion of a weight questionnaire (simply asking their current weight). The patients will be offered a voucher based incentive of £30 for completing both the year 2 and 3 questionnaires (high street voucher), and £10 for the year 3 weight follow-up questionnaire (for those no longer in clinical follow-up/not completing PROMS)

3.1.3 Part 3: Registration for a database of bariatric patients for future research

Patients who have agreed to Parts 1 and 2 will be asked if they are willing to be contacted about possibly taking part in other studies. This will not be an obligation to take part, just an expression of interest in being invited to take part. This will create a database of patients who have had bariatric surgery and are willing to consider participation in future research. Ethical opinion will be sought for the development of these study protocols.

Mackenzie RM, *et al. BMJ Open* 2022; 11:e046441. doi: 10.1136/bmjopen-2020-046441

3.1.4 Service user involvement

SCOTS will uphold the principles embodied by NIHR INVOLVE in relation to user involvement in research, to provide training and support, negotiated roles, time and equitable access for service users involved in this study. SCOTS recruited a patient focus group made of service users who have undergone different procedures both in NHS and private healthcare.

The SCOTS patient focus group have commented on the design of the study, in particular by providing advice on the acceptability of the patient reported outcome questionnaires, the burden to patients and on material and resources that will be given to potential participants. Future sessions are planned to discuss patient recruitment and retention strategies. SCOTS have included a service user on the SCOTS Steering Committee.

3.2 Study Population

All patients due to have a bariatric surgical procedure in Scotland will be invited to participate - both NHS and private sector.

A bariatric surgical procedure is defined as a surgical intervention which has the primary purpose of large scale weight loss in a patient who is obese. Currently those procedures include: duodenal switch; gastric banding; gastric bypass and sleeve gastrectomy. The inclusion of emerging techniques in this definition will be decided by the steering committee.

3.3 Reference populations

Within this protocol the objectives will be met by comparing before and after surgery outcomes for the SCOTS cohort. Additional funding shall be sought to develop protocols to allow comparisons with the following groups for a final Health Technology Assessment report.

1. SCOTS will compare outcomes with the IT based non-surgical weight management database in Greater Glasgow and Clyde (treatment seeking population with current data on 10,000 patients and 3,000 new referrals per year) which has prospective consent for research allowing data linkage for morbidity and mortality along with adjustment for smoking, deprivation, BMI, age and sex.

2. Standardised mortality ratios are available for a BMI matched cohort from the Scottish Health Survey.

3. The Scottish diabetes database allows an anonymous control group matched for key demographics to be created with prospective follow up of diabetes care and complications, compared to those with diabetes who had bariatric surgery.

3.4 Inclusion criteria

- Patients aged 16 years or over undergoing their first bariatric surgery in NHS hospitals or private practice in Scotland.
- Capacity to consent
- Resident in Scotland
- Written informed consent

3.5 Exclusion criteria

- Does not meet inclusion criteria
- Patients who have had previous weight loss surgery (at time of potential recruitment are undergoing a repeat procedure)
- No written informed consent
- Patients under 16 years
- Patients who do not have capacity to consent for themselves
- Weight loss surgery performed at a site outside Scotland
- Patients who live outside Scotland
- Patients who do not have strong enough English language skills to understand the patient information leaflet without assistance will be excluded from Part 2 of the study, the patient questionnaires. If they require a translator to understand the information they may be able to participate in Part 1 of the study which requires no further interaction from the patient. This decision will be made by the clinical teams caring for patients.

3.6 Identification of participants and consent

Patients will be prospectively identified by clinical teams as they are referred for surgery. Patients will be recruited from both NHS and private bariatric surgical practice in Scotland. They will either be recruited by a member of the surgical team prior to undergoing a bariatric surgical procedure, including any significant pre-operative weight loss (such as a very low energy diet for liver shrinkage or use of an intra-gastric balloon) which is considered part of the surgical pathway, or the surgical team will ask the patient’s permission to pass over their name and contact details to the SCOTS research team who will then contact the patient directly to give further information and obtain fully informed consent.

Depending on local patient pathways patient information sheets will be provided to patients at a clinic visits or group session. This will be provided at least 24 hours before the patient will be asked to consent. In the case of those patients who are to be recruited by the research team (identified by surgical team), a patient information sheet will be provided when the permission is sought to hand over contact details to the research team. Patients who meet the eligibility criteria will be identified by their local clinical care teams. All patients undergoing bariatric surgery will be recorded on the screening log at their next appointment. This will allow patients to consider their participation and ask any additional questions they may have about the study face to face. An independent contact is provided on the patient information leaflet who patients can contact to discuss participation in research studies.

They will be asked to give fully informed consent for clinical data linkage (Part 1) and to be contacted by Glasgow University by post, email or phone about to completing the Patient questionnaires (Part 2) and for permission to contact them about future studies (Part 3). Patients who require a translator will only be asked to participate in Part 1. Only patients who agree to Part 1 will be eligible for Part 2 or Part 3.

3.7 Withdrawal of subjects

Participants have the right to withdraw from the study at any point for any reason. The SCOTS web site and patient information sheets will provide contact details for patients if they wish to withdraw from the study. If patients have asked to complete the forms by paper, contact details will be included within each paper questionnaire. The SCOTS team will provide patients with a form to complete, if they are contacted with a request to withdraw from the study.

If the SCOTS team are contacted by a patient who no longer wishes to participate in the study, they will be contact by the SCOTS team at University of Glasgow and asked to specify which following options they would like to apply to their data:

A. (For those within Part 1) I no longer wish to continue the study but I am content to allow my existing data to remain within the study for analysis.

B. (For those consented to Part 1 and 2). I am content for my records to be linked but I no longer wish to complete the patient questionnaires.

C. (For those consented to Part 1, 2 and 3). I am content for my records to be linked but I no longer wish to complete the patient questionnaires or be contacted regarding future studies.

D. For any patient I would like to completely remove my all data from the study and wish not to be contacted again by the study.

Mackenzie RM, *et al. BMJ Open* 2022; 11:e046441. doi: 10.1136/bmjopen-2020-046441

When a request is received from a patient to withdraw from the SCOTS study, a standardised form will be sent to the patient by their preferred communication route. The form will be completed by the patient including the patients name date of birth and address, and the on receipt data manager will flag the patient as having withdrawn from the study. The patient will be asked if they would like to be informed of the findings of the study even though they will no longer be participating in the study.

4 STUDY PROCEDURES

The study design involves local clinical teams identifying and potentially recruiting participants (some participants will be recruited by the SCOTS research team after identification by the local clinical team). Patients who consent to participate will have recruitment data collected by their clinical care teams or the SCOTS research team. Following their operation patients will have their weight loss surgery details entered into the database. The clinical teams then use the system to include follow up weights and re-operations. For the remainder of the study, patients who have consented to part 2 of the study will be asked to complete questionnaires and their health outcomes will be recorded by record linkage to routine data sets. A detailed breakdown of patient contacts with the study is described in Table 4-1 below.

TABLE 4-1: SCOTS PATIENT VISIT SCHEDULE: DESCRIBES EACH INTERATION WITH PATIENTS IF THEY CONSENT TO PART 1 AND 2 OF THE STUDY.

Patient contact	Time	Routine care	SCOTS	Part 1	Part 2	Part 3
1	1 year to 6 weeks before surgery	Agree bariatric surgery	Patients given/sent Invitation to participate letter and patient information sheet (and asked if they are to be contacted by SCOTS research team for further information and informed consent if no local recruitment)	1	1	
2	1 year to 6 weeks before surgery	Patient pre-surgical clinic visit, dietician, psychologist if local recruitment; phone call from SCOTS research team member if research team recruitment	Patients asked if they are willing to consent to participate in SCOTS, Patient signs consent forms, clinical team and patient complete contact details and baseline height and weight.	1	1	
3	At least one month before surgery		Patient contacted by SCOTS team to complete pre-operative questionnaire	1	1	
4	Date of surgery	Patient has weight loss operation	Clinical team enters details of weight loss operation to SCOTS portal	1	1	

5	Patient admitted to hospital	Identified by record linkage	If patient hospital admission is possibly related to bariatric surgery, patient will be contacted by SCOTS Team.	1	1	
7	At routine follow up visits	Patients attend routine clinical visits	Clinical teams enter weight and re-operation details		1	
8	Two year post surgery	Patients have routine annual blood test and results are made available via SCI-store.	Patients complete two year post surgical questionnaire		1	
9	Three years post surgery	Patients have routine annual blood test and results are made available via SCI-store.	Patients complete three year post surgical questionnaire		1	
10	Any point after recruitment		Possibly contacted to participate in future studies			1
11	Three years onward	Patient continues to have routine annual blood test every year	Patient contacted to thank them for completing the PROMS. Record linkage continues. Patients will be informed about future SCOTS publications.		1	
Total				5	9	1

Figure **4-1** shows what happens at each point within the study. This includes background record linkage and patient contacts.

FIGURE 4-1: BASELINE – PRIOR TO OPERATIVE WEIGHT LOSS (INCLUDING PRE LIVER-PREP DIET OR BALLOON PHASE)

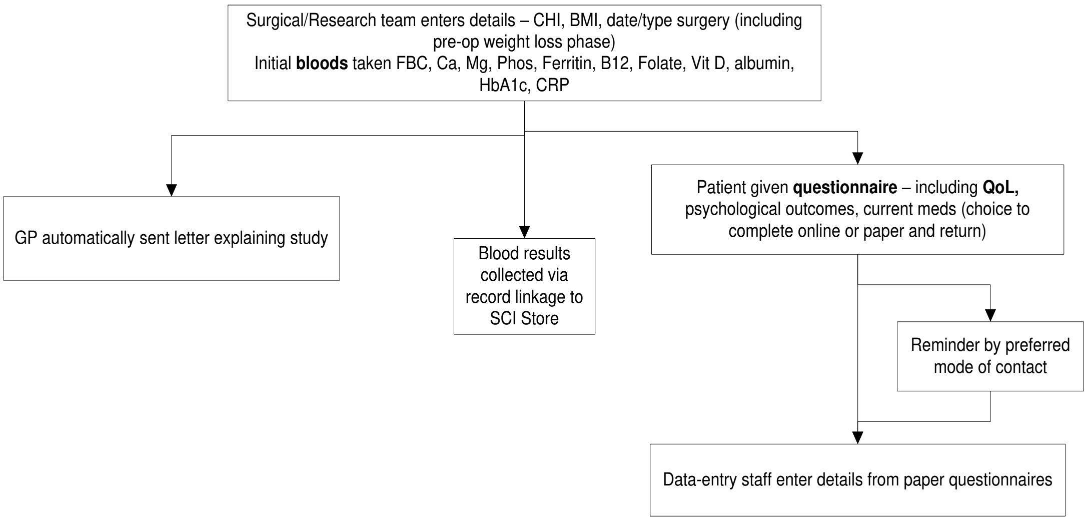

FIGURE 4-2: 2 AND 3 YEARS POST DEFINITIVE SURGERY

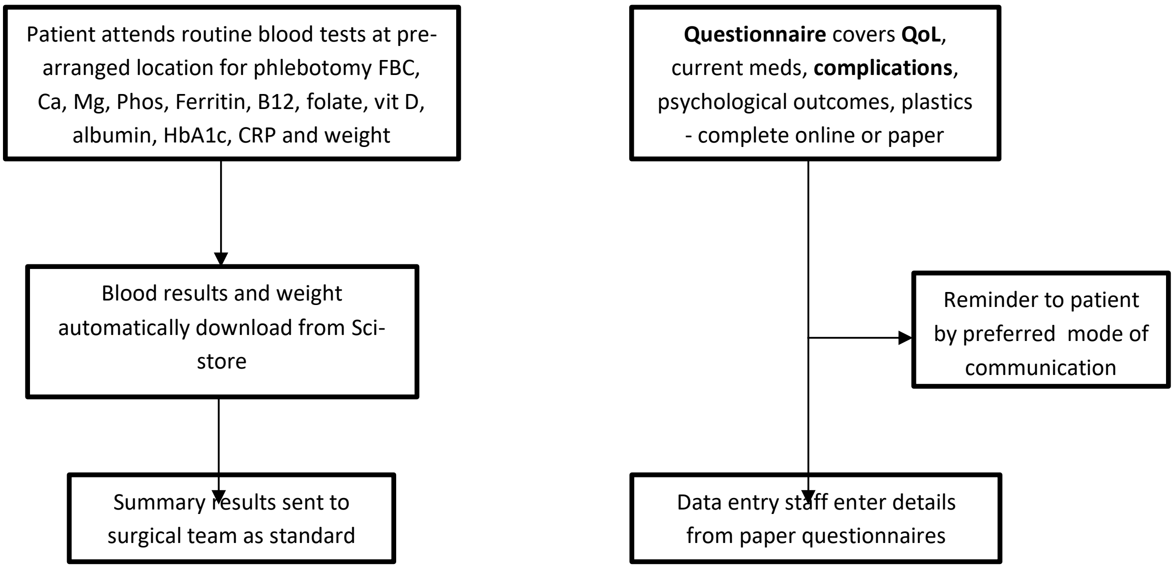

FIGURE 4-3: PART 3 IF NEW BARIATRIC SURGERY STUDY BEGINS

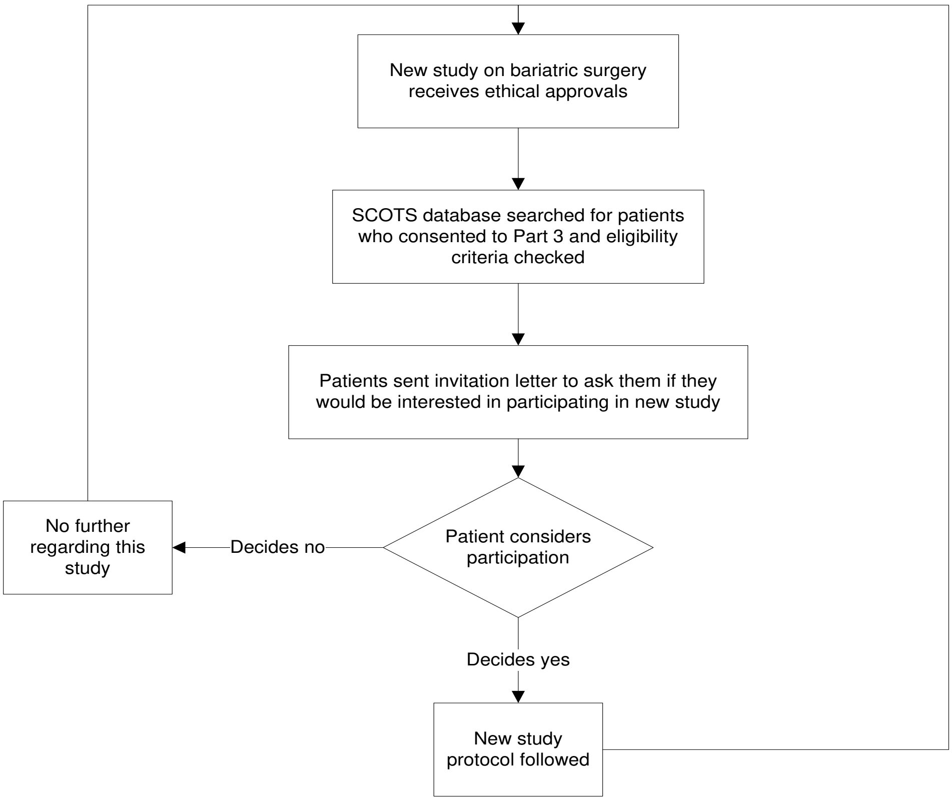

4.1 Duration of participation

Part 1: if funding is available, record linkage, follow up of patients will continue throughout their life. The end of a patient's life will be shown by linkage to the GRO data set. This will be used to flag deceased patients.

Part 2: patient reported outcome measures (PROMS) questionnaires will be collected for a maximum of 3 years. Collection of PROMS will stop after December 2020.

Part 3: Patient contact details will be kept by the study team until the end of the patient's life. The end of a patient's life will be shown by linkage to the GRO data set. This will be used to flag deceased patients and they will be removed from the database in Part 3 to ensure patients families are not contacted after their death. Patient will only be contacted for future studies if record linkage is available to show that they are still living.

Patients will be able to withdraw from the study at any point. Data collected will be retained unless explicitly requested to be removed from the study by the patient.

4.2 Study schedule

This section provides a brief overview of the procedures for investigators at each point within the study. SCOTS will integrate into routine care pathways for bariatric surgery as much as possible. No additional clinic visits will be required. There are five points within the patient journey that the clinical/research team are required to undertake any tasks. These points are described in detail in the following sections.

4.2.1 Start of Study - Surgical team registration

Each site will be defined before the start of the study and login and passwords will be created for each principal investigator and their clinical teams.

A training plan will be developed for clinical teams. Clinical teams will be provided with an overview of the study provided with a training package which will be developed electronically, to be completed at their convenience, electronic guidance will be integrated into the data collection tool to assist them. A user manual will be created which will explain the data which is required for each field and define each data field.

An electronic form will be used to collect surgeon specific data the first time they log onto the bariatric data collections system. This form will be developed by the study Data Centre at the Robertson Centre for Biostatistics, University of Glasgow and access to the form will be restricted, with only authorised site-specific personnel able to make entries or amendments to their data. A manual will be developed to assist investigators with use of the electronic data collection system. It is the investigator's responsibility to ensure completion and to review and approve all data captured. Surgeons will be asked to outline which locations they work in, which procedures they use, and the details of these procedures. They will also be asked information on their surgical experience including date when they qualified, number of procedures the previously year, total number of procedures undertaken in the last five years.

The aim of this task is to ensure that surgeons' data is entered at the start of the study and patient records can be pre-populated to ensure that data can be collected as efficiently as possible.

Checklist:	
Surgeon registers on SCOTS system	
Surgeon enters names and email addresses of clinical team who will be part of SCOTS investigators team	
Investigators have the opportunity to view SCOTS training materials	
SCOTS resource pack is available at the site	

Mackenzie RM, *et al. BMJ Open* 2022; 11:e046441. doi: 10.1136/bmjopen-2020-046441

4.2.2 Visit 1 Routine care invitation visit

Patient pathways vary throughout Scotland, invitation and introduction should take place at either an individual's clinic visit or at a group session discussing weight loss surgery. The aim of this visit is to ensure that patients who are planning weight loss surgery are aware of the SCOTS study, why it is being undertaken and what would be required from them.

4.2.2.1 Invitation visit protocol (in sites with local team recruitment)

1. Patient attends routine clinic to discuss bariatric surgery with clinical team.
2. Patient provided with an overview of SCOTS
3. Patient provided with opportunity to ask initial questions
4. Patient provided with invitation to participate in SCOTS and patient information leaflet (pre-prepared pack).

Checklist:	
Patients provided with overview of SCOTS	
Patient provided with SCOTS information pack	

4.2.2.2 Invitation visit protocol (in sites with SCOTS research team recruitment)

5. Patient attends routine clinic to discuss bariatric surgery with clinical team.
6. Patient provided with a summary overview of SCOTS
7. Patient provided with invitation to participate in SCOTS and patient information leaflet (pre-prepared pack).
8. Patient asked permission for surgical team to pass on contact details to SCOTS research team
9. Participant Identification form completed and returned to research team

Checklist:	
Patients provided with a summary overview of SCOTS	
Patient provided with SCOTS information pack	
Permission sought and Participant Identification Form completed and returned	

4.2.3 Visit 2 Recruitment visit

At the next follow up clinic appointment patients will be asked if they wish to take part in SCOTS. Patients must have been given more than 24 hours to consider their participation.

For patient's in sites where recruitment is carried out by the SCOTS research team the following will occur after the research team receive the participant information form from the local site:

1. SCOTS research team contact patient via telephone
2. Patient provided with an overview of SCOTS
3. Patient provided with opportunity to ask initial questions

Details of all patients who plan to have bariatric surgery will be recorded on a screening log. These logs will recorded on paper. Every patient who plans bariatric surgery will be logged anonymously with details of the individual's age category on date of invitation, gender, calendar quarter surgery planned, if they meet the SCOTS eligibility criteria, if they consent and type of surgery planned (for participants identified via local sites but recruited by the SCOTS team only those giving permission for their details to be forwarded to local teams will have a screening log completed as the research team shall do this). The purpose of this log is to demonstrate if the SCOTS data set is representative of all patients who are having bariatric surgery in Scotland. It will also provide a record of number of patients who consent. A copy of the screening logs will be sent to the Robertson Centre on an annual basis.

This is an anonymised screening record and patients who do not wish to participate will have no further information collected.

4.2.3.1 Informed consent protocol (in sites with local team recruitment)

1. Ask the patient if they have any questions relating to the study documentation. Ensure that the patient fully comprehends the information given.
2. Explain that participation is voluntary and should they decide to proceed with participation in the study, they are free to withdraw at any time without giving an explanation.
3. If a patient chooses to decline, thank them for their time and allow them to leave.
4. If they are unsure, allow further time to consider and arrange a convenient date to meet again reiterating that their participation is voluntary. Offer the option to speak to another member of the study team or an independent advisor.
5. All the check boxes within the consent form must be initialled by the patient to confirm they understand each part of the study. Ask the patient to initial each part of the consent form and sign the bottom section adding their name in block capitals.
6. The person obtaining informed consent signs and dates the consent forms adding their name in block capitals.
7. If a witness is required they can also sign and date the consent form (this may be in the case where an interpreter is required).
8. One copy of the form should be provided to the patient, one filed in the patient's notes and one copy stored in the site folder, and a copy sent to the Robertson Centre for the study files. A copy of the version of the patient information leaflet which was given to the patient should be filed within the site study file.

9. If the patient consents to the study the patient recruitment form should be completed in the presence of the patient. Record patient name and CHI number (for NHS patients), sex, height and weight.
10. Patients should be asked to complete the contact details part of the form including their e-mail and mobile phone contact details and ensure that they identify their preferred route of communication.
11. The Patient recruitment form will be send to the Robertson Centre and a copy filed within the site study file; When they are sent to the Robertson Centre a form will be faxed to show what documentation has been sent in the post, this will be recorded in a postage log.
12. Paper copies of the screening log are returned to the Robertson Centre annually, in self addressed envelopes provided by the SCOTs project team. These will also be recorded in a postage log.

Checklist:	
Check patient name and date of birth correct on patient recruitment form	
Consent from signed and dated at each point	
Patient retains copy of consent form	
Copy of consent form filed in notes	
Copy of consent form in study file	
Copy of consent form in study file sent to Robertson Centre	
Copy of patient recruitment form sent to Robertson Centre	
Copy of patient recruitment form filed in study file	
Consent form and patient recruitment form entered on postage log	

4.2.3.2 Informed consent protocol (in sites with SCOTS research team recruitment)

1. When the Participant Identification Form is received from the surgical team start to complete the SCOTS Recruitment Log and contact the patient via telephone.
2. Ask the patient if they have any questions relating to the study documentation. Ensure that the patient fully comprehends the information given.
3. Explain that participation is voluntary and should they decide to proceed with participation in the study, they are free to withdraw at any time without giving an explanation.
4. Complete the screening log (do not add participant ID as form is to be anonymous)
5. If a patient chooses to decline, thank them for their time and allow them to leave.
6. If they are unsure, allow further time to consider and arrange a convenient date to phone again reiterating that their participation is voluntary. Offer the option to speak to another member of the study team or an independent advisor.
7. All the check boxes within the consent form must be initialled by the patient to confirm they understand each part of the study. Ask the patient to initial each part of the consent form and sign the bottom section adding their name in block capitals.
8. For participants should then be asked post the completed consent form back to the research team in the stamped addressed envelope provided.

If the research team does not receive the completed consent form from the patient within two weeks and cannot reach the patient by telephone, a follow up letter should then be sent.

9. On receipt of the signed form, the person that obtained informed consent signs and dates the consent forms adding their name in block capitals.
10. If a witness is required they can also sign and date the consent form (this may be in the case where an interpreter is required).
11. One copy of the form should be provided to the patient (posted back in case of SCOTS team recruitment), one filed in the patient's notes and one copy stored in the site folder, and a copy sent to the Robertson Centre for the study files. A copy of the version of the patient information leaflet which was given to the patient should be filed within the site study file.
12. If the patient consents to the study the patient recruitment form should be completed with the patient via the telephone. Record patient name and CHI number (for NHS patients), sex, height and weight.
13. Patients should be asked for their contact details including their e-mail and mobile phone contact details and ensure that they identify their preferred route of communication.
14. The Patient recruitment form will be send to the Robertson Centre and a copy filed within the site study file; When they are sent to the Robertson Centre a form will be faxed to show what documentation has been sent in the post, this will be recorded in a postage log.
15. The Letter to Inform Surgeon of Participation should be completed and sent to the surgeon along with the relevant operation details recording form.
16. Paper copies of the screening log are returned to the Robertson Centre annually, in self addressed envelopes provided by the SCOTS project team. These will also be recorded in a postage log.

Checklist:	
Check patient name and date of birth correct on patient recruitment form	
Consent from signed and dated at each point	
Patient sent copy of signed consent form	
Copy of consent form filed in notes	
Copy of consent form in study file	
Copy of consent form in study file sent to Robertson Centre	
Copy of patient recruitment form sent to Robertson Centre	
Copy of patient recruitment form filed in study file	
Consent form and patient recruitment form entered on postage log	

4.2.4 SCOTS study team contact patient

If the patient has consented to Part 2 of the study, the patient will be contacted by staff from the Robertson Centre by their preferred route of communication to invite them to complete a patient reported outcomes questionnaire. They will be provided with a username and initial password which will need to be changed the first time they login to their account. The preadmission questionnaires will be available for the patient to complete. Patients will be able to complete the questionnaire in one or more sittings. If the patients have not logged into the system within two weeks of receiving their information they will be sent a reminder message by their preferred mode of contact. Given the smaller numbers recruited, the patients are now being offered a voucher based incentive to ensure a good return of the two and three year questionnaires. They will be offered an incentive of £30 for completing both the year 2 and 3 questionnaires, and £10 for the year 3 weight follow-up questionnaire (for those no longer in clinical follow-up/not completing PROMS).

Patients will be contacted two and three years after their weight loss operation. Patients will be contacted as part of the study closure phase to thank them for their participation in the study and inform them that they are no longer required to complete the questionnaires.

4.2.5 Visit 3 Weight loss operation

After the patient has their weight loss operation, the clinical teams will enter data onto the SCOTS data collection tool. This will involve the team member who has been delegated to record surgical information on the system. They will log in and be asked for the location of the surgery, which surgeon operated on the patient and their usual operation details will be pre-populated. If the operation is in any way different from the surgeon’s usual technique, an edit function will allow the exact details of the operation to be recorded.

If the operation is a re-operation on an existing SCOTS consented patient, the SCOTS database will be searched to allow the patient’s re-operation to be recorded.

4.2.6 Visit 4 -Post operative follow up

When patients attend follow up visits to see their surgeon, clinical teams will record the patient’s weight and any complications experienced by the patient. If the patient has a gastric band, clinical teams will be able to record band adjustments in the data collection system. Essential fields for the follow-up visits are date of appointment and weight. Within the SCOTS portal it is possible for clinical teams to produce individual reports for patients on their weight loss for inclusion in their medical records, in the letters to GPs or for the patients themselves.

4.2.7 Visit 5 - Re-operation (If applicable)

If a patient requires a re-operation, surgeons will be able to record details of the latest procedure and the reasons for the re-operation. This can happen at any time throughout the study. Details of how the surgeons will record re-operations will be included within the user manual.

Mackenzie RM, *et al. BMJ Open* 2022; 11:e046441. doi: 10.1136/bmjopen-2020-046441

5 STATISTICS AND DATA ANALYSIS

5.1 Statistical Analysis Plan

The study will have separate detailed Statistical Analysis Plans to cover each analyses required for reporting to study committees, for publications and for the final report to the study funder, which will be authored by the study statistician and agreed by the Study Steering Committee before analysis takes place.

An important goal within the funded period is to build various predictive models to use accumulating information to predict good and bad outcomes, so that clinicians can identify treatment opportunities and make better informed intervention management decisions. Generically, these prediction models will relate potentially predictive information – both on entry into the SCOTS database on and around diagnosis, and from subsequent visits and at surgical and other treatment interventions – to outcomes of interest. The mainstay will be Cox proportional hazards time-to-event models, incorporating demographic, clinical, laboratory and life event data (e.g. hospitalisations, strokes, cancers, non-weight loss surgery, and so on) as both fixed and time varying covariates. The influence of missing data on the robustness of predictions in these models will be assessed. Importantly, a hierarchy of models will be built, with ease of use for the clinician in mind, from very simple models built from easy to obtain information, through to more complex models with very many and possibly some difficult and/or expensive to measure covariates.

5.2 Software for statistical analysis

The statistical software to be used for each analysis will be specified in the relevant Statistical Analysis Plan

5.3 Sample size

At the time of grant proposal writing, 230 operations were funded in NHS Scotland each year (of which approximately 60 are bypass). Bariatric surgeons performed an additional 270 private procedures per year and they were willing to commit to entering data (approximately 80 bypass). Therefore 500 procedures per year were expected to be entered into the database with a belief that as numbers of people with severe obesity (BMI >40) are rising rapidly, this number will increase despite financial constraints.

Previous recommendations from the NHS Scotland national planning forum (15)recommended that each health board should aim to provide 9 operations/100,000 population. Scotland's estimated population on the 30th June 2011 was 5,254,800 (16) (http://www.gro-scotland.gov.uk/files2/stats/population-estimates/mid-2011/j22829703.htm). This would mean around 470 operations per year within the NHS alone. This figure was expected to be reached over a number of years. Within private

Mackenzie RM, *et al. BMJ Open* 2022; 11:e046441. doi: 10.1136/bmjopen-2020-046441

healthcare bariatric surgery was estimated to be around 270 per year in Scotland (reported by personal communications from bariatric surgeons).

Given the potential for a surgical learning curve (17), new surgeon's data will be recorded but not analysed for the primary outcome until they have performed 50 operations. Collection of this early data will provide important information on the effects of the learning curve on patient outcomes.

From previous studies (3;4) we expected a 10-year mortality of around 5% (100 deaths). This sample size would allow the mortality rate to be estimated with 95% confidence to within +/- 1% (i.e. for a 5% 10-year death rate, the 95% confidence interval will be between 4% and 6%). We planned to compare this mortality rate with an age-sex matched healthy population from the Registrar General of Scotland's life tables (assumed known with no sampling error). 100 deaths was sufficient to allow us to build a predictive model for death post surgery (conventionally one requires around 10 events per prognostic covariate considered). An initial sample size of 2000 was proposed as it would easily provide adequate power for the original outcomes under investigation.

However, there have been a number of unforeseen recruitment issues that have impacted on the numbers stated above. In order to explore whether the current sample is likely to show meaningful results, the available statistical power for detecting 3 year difference in HbA1c and quality of life (physical and mental components) was calculated using the numbers of participants available as of 29th of July 2016. The majority of data to inform sample size were taken from papers where bariatric procedure was laparoscopic adjustable gastric banding (LAGB) as this is recognised as the least effective of the bariatric procedures so will give a conservative estimate of measure of effect. This shows that there is >99% power to show differences in these outcomes at three years with the current sample size.

In order to explore the likely event rate for cardiovascular events and deaths, we performed health record linkage for currently recruited participants, linking with inpatient care and death records. Follow-up is from the date of surgery, so for those patients that went on to have surery details entered (n=180), there are 272 separate admissions. Of those 272, there are 72 'emergency' admission, and 4 of these, in 3 patients, are 'circulatory disease' using the main condition only. The codes included for these hospital admissions are;

· Angina pectoris (x1)
· Acute MI (x1)
· Pulmonary embolism without mention of acute cor pulmonale (x1)
· Orthostatic hypotension (x1)

Using date of operation as the starting point, we have a total (crude) follow-up time of 203.52 years (the mean is 1.13 years and the median is 1.04 years). As the number of cardiovascular events is so low, it is impossible to extrapolate this to a future event rate at this time. It should be noted that the participants have been cleared as healthy for elective surgery meaning that it is unlikely that there would be many cardiovascular events in early follow-up.

Glasgow Clinical Trials Unit

Following discussions with the NIHR it was agreed that recruitment will stop at approximately 400 patients and these numbers will be sufficient to answer the majority of objectives initially set.

5.4 Management and delivery

The Robertson Centre for Biostatistics, part of the Glasgow Clinical Trials Unit, a fully registered UK CRN Clinical Trials Unit, will manage and analyse trial data.

Mackenzie RM, *et al. BMJ Open* 2022; 11:e046441. doi: 10.1136/bmjopen-2020-046441

6 STUDY CLOSURE

Part 1 Record linkage: under the current funding record linkage will continue until December 2020. If additional funding is available Part 1 record linkage will continue until the end of the patient's life. The end of a patient's life will be shown by linkage to the GRO data set. This will be used to flag deceased patient and they will be removed from the database in Part 3 to ensure patients families are not contacted after their death.

Part 2 Patient reported Questionnaires: The study will end when the steering committee agrees that one or more of the following situations applies:

- Observation has been undertaken until December 2020
- Sufficient patients have completed the 3 year post surgery questionnaire

Patients will be contacted to thank them for their participation in the patient reported outcomes part of the study.

Part 3 Bariatric Surgery Research Studies. Patients contact details will be maintained until the end of the patient's life.

7 Data Handling

Data for the SCOTS study will be collected from a number of sources. These are summarised in Table 3-1. A detailed data management plan will be developed by the Robertson Centre team, which will include verification of paper data entry and validation checks. A separate protocol will be developed for each data linkage.

7.1.1 Surgeon registration data

An electronic data collection form will be developed by the study Data Centre at the Robertson Centre for Biostatistics, University of Glasgow. These data will include the surgeons' usual procedures and registration of the clinical teams. These data will be used to complete the operation details for each patient. It will be the investigators responsibility to ensure that these data are entered correctly and are kept up to date.

7.1.2 Patient recruitment data

A paper case report form (CRF) will be used to collect patient contact details and height and weight at time of recruitment will be completed by the clinical teams when the patient has consented to participate in SCOTS. Patients will be asked to specify their preferred form of communication throughout the study. They will be told that they may be contacted via another route if they do not respond. This CRF will be developed by the study Data Centre at the Robertson Centre for Biostatistics, University of Glasgow. Competed forms will be checked by the clinical teams and sent to the Robertson Centre for initial data entry. These forms will be sent along with signed consent forms. It is the investigator's responsibility to ensure completion and to review and approve all data captured in the CRF. A standard

Mackenzie RM, *et al. BMJ Open* 2022; 11:e046441. doi: 10.1136/bmjopen-2020-046441

operating procedure will be used by the Roberson Centre to ensure that information is entered into the SCOTS database in an accurate and timely manner.

7.1.3 Surgical data collection

An electronic case report form (e-CRF) will be used to collect data on weight loss surgery procedures. On or shortly after the patient undergoes weight loss surgery a member of the clinical team will record specific information on the patient's weight loss operation. Each surgeon will have been asked to register the locations they operate in, which procedures they perform and details of their usual specifications. This will then populate the fields for a particular type of surgery and clinical teams will be asked to confirm that the usual techniques have been applied for each procedure. Access to the e-CRF will be restricted, with only authorised site-specific personnel able to make entries or amendments to their patients' data. It is the investigator's responsibility to ensure completion and to review and approve all data captured in the e-CRF, however, a member from the SCOTS study team, with the appropriate NHS Research Passport permissions, will visit the site staff at least once per year to ensure surgical and follow-up data are complete. Data will be validated at the point of entry into the e-CRF and at regular intervals during the study. Data discrepancies will be flagged to the study site and any data changes will be recorded in order to maintain a complete audit trail (reason for change, date change made, who made change).

7.1.4 Patient Reported Outcomes

An electronic case report form (e-CRF) or a paper case report form (depending on patient preference) will be used to collect patient reported study data. The e-CRF and CRF will be developed by the study data centre at the Robertson Centre for Biostatistics, University of Glasgow and access to the e-CRF will be restricted, with only authorised site-specific personnel able to make entries or amendments to their patients' data. All data handling procedures will be detailed in a study specific Data Management Plan.

7.1.5 Routine clinical information

A number of features have been built into the SCOTS system which will be available for surgeons to use during routine follow up visits. Each time a patient attends, their weight will be recorded. Notes can be included and exported for inclusion within patient notes, provided for patients or within letters to GPs.

7.1.6 Re-operation

An electronic case report form (e-CRF) will be used to collect data on re-operations. On or shortly after the patient undergoes weight loss surgery a member of the clinical team will record specific information on the patients repeat weight loss operation. Not only will the details of the operation be recorded but the reason for operation will be included within the e-CRF.

Mackenzie RM, *et al. BMJ Open* 2022; 11:e046441. doi: 10.1136/bmjopen-2020-046441

7.1.7 Record Retention

To enable evaluations and/or audits from regulatory authorities, the investigator agrees to keep records, including the identity of all participating subjects (sufficient information to link records), all original signed informed consent forms, source documents, and detailed records of treatment disposition in accordance with ICH GCP, local regulations, or as specified in the Clinical Study Agreement, whichever is longer. Data will be retained at the Robertson Centre once the recruitment phase of the study is complete. During recruitment a copy of the patient consent form and the patient registration will be retained within the site file and within the patient notes.

7.1.8 Access to data

Clinical teams will be able to access their own data. All patients who have been entered within the SCOTS system will be viewed by the clinical teams who entered data. Data which is routinely collected for the National Bariatric Surgery Registry (NBSR) can be entered within the system for surgeons who wish to contribute to this process. Surgeons fully anonymised data (no patient identifiable information) can be exported to a file which surgeons can share with the NBSR if they surgeons wish to contribute. The management and approvals for this will be responsibility of the investigating surgeon and data will not be shared by the SCOTS team.

8 SCOTS STUDY MANAGEMENT

8.1 Study Sponsor

NHS Greater Glasgow and Clyde Research and Development Office as study sponsors are responsible for the management and conduct (according to GCP). The SCOTS steering committee (SSC) oversee the management and conduct of the study and report to and advise the sponsor on the study. All minutes from both the SCOTS management group (SMG) and SCOTS steering committee (SSC) are copied to NHS Greater Glasgow and Clyde Research and Development Office

8.2 SCOTS Study Team

A study team comprising of the chief investigator, a research lead and a project manager, in collaboration with the data management team based at the Robertson Centre will run the project on a day to day basis. Issues arising from patients or investigators will be managed by this team and those that cannot be resolved by that team will be discussed with the chief Investigator and escalated to the study management group for discussion.

8.3 SCOTS Principal investigators

Each site will have an identified principal investigator who will oversee the application of this protocol locally. Each member of the clinical team involved in the study will be required to register for a login and password to access the e-CRF through the SCOTS data collection portal. Any member of the local clinical teams who has registered username and password and entered data to the SCOTS data collection portal will be a member of the SCOTS Investigators Group. The investigators will be required to enter some initial registration data to link themselves with the surgeons they work with. Planned publications outlined in Table 15-1 will be written on behalf of the SCOTS Investigators Group.

8.4 Routine management of study: SCOTS Management Group (SMG)

The trial will be coordinated from the University of Glasgow by the SCOTS management group (SMG). The SMG will consist of the principal investigator, other co-applicants, project manager and representatives from the Clinical Trials Unit. The role of the group is to monitor all aspects of the conduct and progress of the trial, ensure that the protocol is adhered to and take appropriate action to safeguard participants and the quality of the trial itself. It will be responsible for the day to day running of the trial and budget. It will meet at least 6 monthly, with more meetings initially and as required, and provide information to the steering committee as to the progress of the study.

Mackenzie RM, *et al. BMJ Open* 2022; 11:e046441. doi: 10.1136/bmjopen-2020-046441

8.5 SCOTS Steering Committee (SSC)

SCOTS Steering Committee (SSC) will be established to provide overall supervision of the study and ensure adherence to GCP. It will have an independent chair, a patient representative, and at least two other independent academics along with the principal investigator. Observers from the HTA programme will be invited to SSC meetings, and all SSC papers will be supplied to the HTA programme. The SSC will meet at the start of the study, and annually thereafter.

The role of the SSC is to provide overall supervision of the trial and ensure that it is being conducted in accordance with the principles of GCP and the relevant regulations. The SSC will:

- agree the trial protocol and any protocol amendments
- provide advice to the investigators on all aspects of the trial
- include members who are independent of the investigators, in particular an independent chairperson.

Decisions about continuation or termination of the trial or substantial amendments to the protocol are usually the responsibility of the SSC who advise the sponsor.

9 STUDY MONITORING/AUDITING

This study is a cohort study. It will therefore be audited on random sampling basis by GG&C auditors on behalf of the GG&C sponsor who audit 10% of sponsored studies per annum.

10 PROTOCOL AMENDMENTS

Any change in the study protocol will require an amendment. Any proposed protocol amendments will be initiated by the CI following discussion with the SSC and any required amendment forms will be submitted to the regulatory authority, ethics committee and sponsor. The CI and the SSC will liaise with study sponsor to determine whether an amendment is non-substantial or substantial. All amended versions of the protocol will be signed by the CI and sponsor representative. Before the amended protocol can be implemented favourable opinion/approval must be sought from the original reviewing Research and Ethics Committee (REC) and Research and Development (R&D) office(s).

11 ETHICAL CONSIDERATIONS

11.1 Ethical conduct of the study

The study will be carried out in accordance with the World Medical Association Declaration of Helsinki (1964) and its revisions (Tokyo [1975], Venice [1983], Hong Kong [1989], South Africa [1996] and Edinburgh [2000]).

Favourable ethical opinion will be sought from an appropriate REC before patients are entered into this study. Patients will only be allowed to enter the study once they have provided written informed consent.

The CI will be responsible for updating the REC of any new information related to the study.

11.2 Informed consent

Patients will be informed of the SCOTS study as they discuss weight loss surgery with their clinical teams. They will be provided with an information sheet on the study by a trained member of the clinical teams during a routine clinic visit or during a group session depending on the local patient pathway. The patient will be given time to consider their participation in the study, review the SCOTS information sheet and ask about any concerns they may have. Patients will then be asked to sign the SCOTS consent form.

Mackenzie RM, *et al. BMJ Open* 2022; 11:e046441. doi: 10.1136/bmjopen-2020-046441

Glasgow Clinical Trials Unit

12 INSURANCE AND INDEMNITY

SCOTS is sponsored by NHS Greater Glasgow & Clyde and NHS indemnity is provided under the Clinical Negligence and Other Risks Indemnity Scheme (CNORIS). In addition, as the protocol authors are employees of the University of Glasgow negligent harm caused by the design of the trial will be covered by the Clinical Trials Insurance policy held by the University of Glasgow.

Investigators working in private healthcare will recruit to this NHS funded study as part of their NHS role and will therefore be covered as detailed above.

13 FUNDING

Funding source: National Institute for Health Research (NIHR) Health Technology Assessment Programme

Grant Award number: 10/42/02

Original Cost: £2,124,030

Amended Cost: £1,516,255.60

URL of this project on the NIHR Website: http://www.hta.ac.uk/project/2608.asp

Start Date: January 2012

End date: June 2021.

Estimated date of publication in HTA journal series: Late 2021. This date takes account of time for report preparation and printing based on current average times for these activities.

International Standard Randomised Controlled Trial Number: ISRCTN 47072588

URL of this project on the Controlled Trials Website: http://www.controlled-trials.com/ISRCTN47072588

14 ANNUAL REPORTS

A bi-annual progress report will be submitted to NIHR (the funder), the first being submitted 6 months from the date that all funding started (January 2012).

Annual reports will be submitted to the ethics committee and sponsor with the first submitted one year after the date that all study related approvals are in place. These reports will follow the template on the IRAS web site.

15 DISSEMINATION OF FINDINGS

Study results whether, negative or positive, will be disseminated. Details of the plan for dissemination are detailed below. Some of the outcomes described may be reported in a number of peer reviewed journal articles. The SCOTS Publication and Presentation SOP (18) describes how this will be managed throughout the study and will ensure that NIHR guidance for publications is adhered to.

TABLE 15-1: SCOTS PUBLICATION PLANS

	Date	Description of publication
1	**Jan 2015**	Methods paper: Outlining the methods used in the study including the IT based nutritional blood monitoring and the dataset being collected. Published – Logue et al BMJ Open 2015;5:e008106
2	**Jul 2017**	Health and socio-economic burden of severe obesity in a population undergoing bariatric surgery.
3	**Jul 2019**	The effect of the pre and post-surgery pathway and surgical experience on weight and complications outcomes after bariatric surgery.
4	**Jun 2021**	Change in health and socioeconomic factors 3 years after bariatric surgery.<br>Full HTA report within the NIHR HTA Journal Series.

16 REFERENCES

(1) Picot J, Jones J, Colquitt JL, Gospodarevskaya E, Loveman E, Baxter L, et al. The clinical effectiveness and cost-effectiveness of bariatric (weight loss) surgery for obesity: a systematic review and economic evaluation. Health Technol Assess 2009 Sep;13(41):1-357, iii.

(2) O'Brien PE, Sawyer SM, Laurie C, Brown WA, Skinner S, Veit F, et al. Laparoscopic adjustable gastric banding in severely obese adolescents: a randomized trial. JAMA 2010 Feb 10;303(6):519-26.

(3) Adams TD, Gress RE, Smith SC, Halverson RC, Simper SC, Rosamond WD, et al. Long-term mortality after gastric bypass surgery. N Engl J Med 2007 Aug 23;357(8):753-61.

(4) Sjostrom L, Narbro K, Sjostrom CD, Karason K, Larsson B, Wedel H, et al. Effects of bariatric surgery on mortality in Swedish obese subjects. N Engl J Med 2007 Aug 23;357(8):741-52.

(5) Dixon JB, O'Brien PE, Playfair J, Chapman L, Schachter LM, Skinner S, et al. Adjustable gastric banding and conventional therapy for type 2 diabetes: a randomized controlled trial. JAMA 2008 Jan 23;299(3):316-23.

(6) Buchwald H, Estok R, Fahrbach K, Banel D, Jensen MD, Pories WJ, et al. Weight and type 2 diabetes after bariatric surgery: systematic review and meta-analysis. Am J Med 2009 Mar;122(3):248-56.

(7) Sjostrom L, Lindroos AK, Peltonen M, Torgerson J, Bouchard C, Carlsson B, et al. Lifestyle, diabetes, and cardiovascular risk factors 10 years after bariatric surgery. N Engl J Med 2004 Dec 23;351(26):2683-93.

(8) Sjostrom L, Lindroos AK, Peltonen M, Torgerson J, Bouchard C, Carlsson B, et al. Lifestyle, diabetes, and cardiovascular risk factors 10 years after bariatric surgery. N Engl J Med 2004 Dec 23;351(26):2683-93.

(9) Mathus-Vliegen EM, de Wit LT. Health-related quality of life after gastric banding. Br J Surg 2007 Apr;94(4):457-65.

(10) Karlsson J, Taft C, Ryden A, Sjostrom L, Sullivan M. Ten-year trends in health-related quality of life after surgical and conventional treatment for severe obesity: the SOS intervention study. Int J Obes (Lond) 2007 Aug;31(8):1248-61.

(11) Ford I, Murray H, Packard CJ, Shepherd J, Macfarlane PW, Cobbe SM. Long-term follow-up of the West of Scotland Coronary Prevention Study. N Engl J Med 2007 Oct 11;357(15):1477-86.

(12) Research Governance Framework for Health and Community Care (Second edition). Scottish Executive Health Department; 2006.

(13) NHS National Services Scotland. ISD Statistical Disclosure Control Protocol. Edinburgh: ISD Scotland Publications; 2010.

(14) Scottish Intercollegiate Guideline Network. Management of Obesity 115 - A national clincal guideline. Edinburgh: Scottish Intercollegiate Guidelines Network; 2010 Feb 1. Report No.: 115.

(15) Knox H, Davies S. Obestity Treatment Best Practice Guidance. Edinburgh: SGHD Quality Unit, Planning and Quality Division; 12 A.D. Jul 1.

(16) Mid-2011 Population Estimates Scotland Population estimates by sex, age and administrative area.

National Statistics publication for Scotland; 2012 May 31.

(17) Chevallier JM, Paita M, Rodde-Dunet MH, Marty M, Nogues F, Slim K, et al. Predictive factors of outcome after gastric banding: a nationwide survey on the role of center activity and patients' behavior. Ann Surg 2007 Dec;246(6):1034-9.

(18) SCOTS Management Team. Surgical Obesity Treatment Study (SCOTS) Publication and Presentation Group Operating Procedures. 2012.

(19) Scottish Government. Scottish Health Survey 2010 - Volume 2: Technical Report. 2008.

(20) General Register Office. Scottish Census 2011 Household Questionnaire. General Register Office, editor. GRO . 2011.

Ref Type: Online Source

(21) Bruce J, Thornton AJ, Scott NW, Marfizo S, Powell R, Johnston M, et al. Chronic preoperative pain and psychological robustness predict acute postoperative pain outcomes after surgery for breast cancer. Br J Cancer 2012 Sep 4;107(6):937-46.

(22) Mercer SW, Watt GC. The inverse care law: clinical primary care encounters in deprived and affluent areas of Scotland. Ann Fam Med 2007 Nov;5(6):503-10.

(23) Wadden TA, Foster GD. Weight and Lifestyle Inventory (WALI). Obesity (Silver Spring) 2006 Mar;14 Suppl 2:99S-118S.

(24) Diabetes UK. Diabetes Risk Score. 2012. Diabetes UK.

Ref Type: Online Source

(25) Ware J, Jr., Kosinski M, Keller SD. A 12-Item Short-Form Health Survey: construction of scales and preliminary tests of reliability and validity. Med Care 1996 Mar;34(3):220-33.

(26) The EuroQol Group. EuroQol-a new facility for the measurement of health-related quality of life. Health Policy 1990;16(3):199-208.

(27) Spitzer RL, Kroenke K, Williams JB. Validation and utility of a self-report version of PRIME-MD: the PHQ primary care study. Primary Care Evaluation of Mental Disorders. Patient Health Questionnaire. JAMA 1999 Nov 10;282(18):1737-44.

(28) Cameron IM, Crawford JR, Lawton K, Reid IC. Psychometric comparison of PHQ-9 and HADS for measuring depression severity in primary care. Br J Gen Pract 2008 Jan;58(546):32-6.

(29) Spitzer RL, Kroenke K, Williams JB, Lowe B. A brief measure for assessing generalized anxiety disorder: the GAD-7. Arch Intern Med 2006 May 22;166(10):1092-7.

(30) Kolotkin RL, Crosby RD. Psychometric evaluation of the impact of weight on quality of life-lite questionnaire (IWQOL-lite) in a community sample. Qual Life Res 2002 Mar;11(2):157-71.

(31) Kolotkin RL, Crosby RD, Pendleton R, Strong M, Gress RE, Adams T. Health-related quality of life in patients seeking gastric bypass surgery vs non-treatment-seeking controls. Obes Surg 2003 Jun;13(3):371-7.

(32) Gosman GG, King WC, Schrope B, Steffen KJ, Strain GW, Courcoulas AP, et al. Reproductive health of women electing bariatric surgery. Fertil Steril 2010 Sep;94(4):1426-31.

(33) Derby CA, Araujo AB, Johannes CB, Feldman HA, McKinlay JB. Measurement of erectile dysfunction in population-based studies: the use of a single question self-assessment in the Massachusetts Male Aging Study. Int J Impot Res 2000 Aug;12(4):197-204.

(34) Barry MJ, Fowler FJ, Jr., O'Leary MP, Bruskewitz RC, Holtgrewe HL, Mebust WK, et al. The American Urological Association symptom index for benign prostatic hyperplasia. The Measurement Committee of the American Urological Association. J Urol 1992 Nov;148(5):1549-57.

(35) Abrams P, Avery K, Gardener N, Donovan J. The International Consultation on Incontinence Modular Questionnaire: www.iciq.net. J Urol 2006 Mar;175(3 Pt 1):1063-6.

(36) Macran S, Wileman S, Barton G, Russell I. The development of a new measure of quality of life in the management of gastro-oesophageal reflux disease: the Reflux questionnaire. Qual Life Res 2007 Mar;16(2):331-43.

(37) Grant A, Wileman S, Ramsay C, Bojke L, Epstein D, Sculpher M, et al. The effectiveness and cost-effectiveness of minimal access surgery amongst people with gastro-oesophageal reflux disease - a UK collaborative study. The REFLUX trial. Health Technol Assess 2008 Sep;12(31):1-iv.

(38) Helander A, Lunner K, Leifman A, Borg S. [Alcohol-related problems in connection with overweight surgery]. Lakartidningen 2011 May 4;108(18):964-5.

(39) Babor TF, Higgins-Biddle JC, Saunders JB, Monteiro MG. The Alcohol Use Disorders Identification Test. World Health Organisation Department of Mental Health and Substance Dependence; 2001 Jan 1. Report No.: WHO/MSD/MSB/01.6a.

(40) Craig CL, Marshall AL, Sjostrom M, Bauman AE, Booth ML, Ainsworth BE, et al. International physical activity questionnaire: 12-country reliability and validity. Med Sci Sports Exerc 2003 Aug;35(8):1381-95.

(41) Roe L, Strong C, Whiteside C, Neil A, Mant D. Dietary intervention in primary care: validity of the DINE method for diet assessment. Fam Pract 1994 Dec;11(4):375-81.

(42) Scheier MF, Carver CS, Bridges MW. Distinguishing optimism from neuroticism (and trait anxiety, self-mastery, and self-esteem): a reevaluation of the Life Orientation Test. J Pers Soc Psychol 1994 Dec;67(6):1063-78.

(43) Foster GD, Wadden TA, Vogt RA, Brewer G. What is a reasonable weight loss? Patients' expectations and evaluations of obesity treatment outcomes. J Consult Clin Psychol 1997 Feb;65(1):79-85.

(44) Health Protection Scotland. The epidemiology of orthopaedic surgical site infection occuring up to one year after surgery: a feasibility study of telephone screening and direct observation by trained healthcare workers. Health Protection Scotland; 2010.

(45) Horan TC, Andrus M, Dudeck MA. CDC/NHSN surveillance definition of health care-associated infection and criteria for specific types of infections in the acute care setting. Am J Infect Control 2008 Jun;36(5):309-32.

(46) Ertlet TW, Marino JM, Mitchell JE. Psychosocial aspects of body contourning surgery after bariatric surgery. In: Shiffman MA, Di Giuseppe A, editors. Body Contouring.Berlin: Springer-Verlag; 2010. p. 633-9.

(47) Keating CL, Moodie ML, Bulfone L, Swinburn BA, Stevenson CE, Peeters A. Healthcare Utilization and Costs in Severely Obese Subjects Before Bariatric Surgery. Obesity (Silver Spring) 2012 May 4.

(48) (DIRUM) Database of Instruments for Resource Use Measurement. 2012. Bangor, Bangor University.

Ref Type: Online Source

(49) Petrou S. WoLFF (Wound dressings in Lower Limb Fracture) Questionnaire. 2012. 1-8-2012.

(50) EPIC Norfolk Study. Food Frequency Questionnaire. Cambridge: University of Cambridge; 2012. Report No.: (CAMB/PQ/6/1205).

Mackenzie RM, *et al. BMJ Open* 2022; 11:e046441. doi: 10.1136/bmjopen-2020-046441

17 Appendix E: Summary of Patient Reported Outcome Questions

Table 17-1 shows a summary table of each set of questions which will be asked of the patient, when they will be asked and if they are validated or not validated.

Questions are classed as not validated in the form they are being asked if they have not been used in a published study and are identified by either a 0 or P in the validated column. 0 means that the set of questions are not validated P means that the questions are partially validated. Partially validated question sets are where a previously published question set has had additional questions added in order to fulfil the SCOTS objectives.

TABLE 17-1: SUMMARY OF SCOTS PATIENT REPORTED OUTCOME MEASURES.

Glasgow Clinical Trials Unit

PROMS	Total	Total Male	Total Femal	Validated	baseline	2 year	3 year	Total
About you								
Demographics – Essential	1	1	1	P	1	1	1	3
Marital Status	1	1	1	1	1	1	1	3
Ethnicity	1	1	1	1	1	0	0	1
Education	1	1	1	1	1	0	0	1
Occupation	1	1	1	P	1	1	1	3
Health History								
Your Medical History	1	1	1	1	1	0	0	1
Your Weight History	1	1	1	1	1	0	0	1
Family History	1	1	1	P	1	0	0	1
Family History of Obesity	1	1	1	0	1	0	0	1
Quality of Life								
SF-12 v2 –licence required	1	1	1	1	1	1	1	3
EQ5D 5L	1	1	1	1	1	1	1	3
Patient Health Questionnaire (PHQ-9) (No permissions Free)	1	1	1	1	1	1	1	3
Generalized Anxiety Disorder GAD-7 (No permissions Free)	1	1	1	1	1	1	1	3
Impact of Weight								
Impact of Weight on Quality of Life (IWQOL-Lite) – Licence required	1	1	1	1	1	1	1	3
Reproductive Health – Females only	1	0	1	1	1	1	1	3
Massachusetts Male Aging Study: Single Question Assessment Males only	1	1	0	1	1	1	1	3
International Prostate Symptom Score (IPSS) Males only	1	1	0	1	1	1	1	3
Continence	1	1	1	1	1	1	1	3
Reflux Questionnaire	1	1	1	1	1	1	1	3
Lifestyle								
Smoking	1	1	1	1	1	1	1	3
The Alcohol Use Disorders Identification Test: Self-Report Version	1	1	1	1	1	1	1	3
Physical Activity IPAC short form self administered	1	1	1	1	1	1	1	3
Nutrition	1	1	1	1	1	1	1	3
Personal Outlook								
Life Orientation Test (REVISED)	1	1	1	1	1	1	0	2
Expectations of Surgery	1	1	1	P	1	0	0	1
Surgical Recovery								
Post operative pain (1 month acute, 6 month-1 year chronic)	1	1	1	0	0	0	0	0
Surgical site infection	1	1	1	1	0	0	0	0
Skin Excess	1	1	1	0	0	0	0	0
Plastic surgery	1	1	1	P	0	0	0	0
Healthcare and Support								
Outpatient care	1	1	1	1	1	1	1	3
Contacts with other health and social care professionals	1	1	1	1	1	1	1	3
Devices and specialist equipment	1	1	1	1	1	1	1	3
Benefits	1	1	1	1	1	1	1	3
Multivitamins and Supplements use	1	1	1	1	1	1	1	3
	34	33	32	**26**	**30**	**23**	**22**	**75**

Mackenzie RM, *et al. BMJ Open* 2022; 11:e046441. doi: 10.1136/bmjopen-2020-046441

This page is intentionally blank

Mackenzie RM, *et al. BMJ Open* 2022; 11:e046441. doi: 10.1136/bmjopen-2020-046441
