# Supplementary material for: SurgiCal Obesity Treatment Study (SCOTS): a prospective, observational cohort study on health and socioeconomic burden in treatment-seeking individuals with severe obesity in Scotland, UK
Source: BMJ Open. 2021 Aug 26;11(8):e046441. doi: 10.1136/bmjopen-2020-046441 (PMC8395268; doi:10.1136/bmjopen-2020-046441)
Supplement: Supplementary data [file bmjopen-2020-046441supp003.pdf]

Medical history questions included in baseline questionnaires/patient reported outcome measures (PROMs).

Why we are asking you these questions?

It is important to know what illness patients have before their surgery so we can look at how much overall health has changed after their weight loss surgery.

Medical History

1. Do you have a history of any of the following? – tick all that apply

- ☐ Deep vein thrombosis (Deep vein thrombosis (DVT) is a blood clot in one of the deep veins in the leg.)
- ☐ Pulmonary embolism (A pulmonary embolism is a blood clot in the pulmonary artery which is the blood vessel that transports blood from the heart to the lungs.)
- ☐ High blood pressure
- ☐ Diabetes
- ☐ Angina/heart attack
- ☐ Heart Failure
- ☐ Stroke/mini-stroke
- ☐ Arthritis
- ☐ Back problems
- ☐ Chronic Bronchitis
- ☐ Eczema/psoriasis
- ☐ Asthma
- ☐ Thyroid problems
- ☐ Migraine
- ☐ Anxiety/depression
- ☐ Kidney disease
- ☐ Liver disease
- ☐ Cancer
- ☐ Irritable bowel syndrome
- ☐ Doctor diagnosed sleep apnoea (Obstructive sleep apnoea (OSA) is a condition that causes interrupted breathing during sleep and requires a machine to keep airways open while you sleep. Only check this box if your doctor has diagnosed sleep apnoea.)
- ☐ Other (please specify _______________)

SCOTS

Patient Questionnaire

Smoking

Draft 01

Lifestyle

This section asks about your smoking, drinking, eating and exercising habits.

Why are we asking you these questions

Smoking habits may alter after weight loss surgery.

Smoking

1. Smoking history

Current ☐ 1
Former ☐ 2
Never ☐ 3

If Current,

1a. How many per day? |_|_|_|

If former,

1a. Year stopped? |_|_|_|_| (Y Y Y Y)

Produced by Robertson Centre for Biostatistics, University of Glasgow

SCOTS

Patient Questionnaire

Work

Draft 02

Why are we asking you these questions

The ability to work or go about usual activity is an important part of a patient's overall health and quality of life. We are interested to see if there is a change in your occupation before and after your weight loss surgery.

<u>Occupation</u>

1. Please select a category that best describes your current employment status

Working full time (30hrs or more per week)	☐ 1	please go to section A
Working part time (less than 30hrs or more per week)	☐ 2	please go to section A
Unable to work because of illness or disability	☐ 3	please go to section B
Student	☐ 4	please go to section A
Unemployed and looking for work	☐ 5	please go to section B
Carer for children or relative	☐ 6	please go to section B
At home and not looking for paid employment	☐ 6	please go to section B
Other	☐ 6	please go to section B

Section A

1. What is your current occupation? Since many people have more than one job at a given time, we would like to know about the job that is your primary source of income

   ____

2. Have you had any days off sick in the last month? Yes ☐ 1 No ☐ 2 Not applicable ☐ 3

   a. If Yes, how many days in total (please only include the number of days you were not able to work)? |_|_|_|

3. Are you receiving benefits? Yes ☐ 1 No ☐ 2

Section B

1. Are you receiving benefits? Yes ☐ 1 No ☐ 2

Produced by Robertson Centre for Biostatistics, University of Glasgow

Social security (benefits) questions included in baseline questionnaires/patient reported outcome measures (PROMs).

Why we are asking you these questions?

Weight loss surgery can be expensive, but often the improvements in patients' health are great. This is to be used in health economic analysis which balances the cost of weight loss surgery with the improvements in patients' health and wellbeing and overall cost of bariatric surgery.

Due to the personal nature of these questions, you may choose not to complete them.

I am happy to complete these questions

- ☐ Yes
- ☐ No

If No, please go to the next section.

Benefits

1. In the past three months, you may have received some benefits from the government to support you. In the table below, please tick all benefits you have received in the past 3 months.

- ☐ Attendance Allowance
- ☐ Carer's Allowance
- ☐ Child Tax Credit
- ☐ Council Tax Benefit
- ☐ Disability Living Allowance—caring
- ☐ Disability Living Allowance—mobility
- ☐ Employment and Support Allowance
- ☐ Housing Benefit
- ☐ Income Support
- ☐ Jobseeker's Allowance
- ☐ Pension Credit
- ☐ Statutory Sick Pay
- ☐ State Pension
- ☐ Other (please specify _______________)
- ☐ None

Healthcare utilisation (devices and specialist equipment) questions included in baseline questionnaires/patient reported outcome measures (PROMs).

Why we are asking you these questions?

Some people may require specialist equipment or devices as a result of their weight and changes in requirements for equipment will be considered in the overall cost of weight loss surgery.

Devices and Specialist Equipment

1	Do you currently use any aids or specialist equipment?	☐ Yes<br>☐ No<br>If No, move to next section	
1a	Stair lift	☐ Yes<br>☐ No	If Yes, funded by:<br>☐ provided by Social Services<br>☐ provided by hospital<br>☐ bought by you
1b.	Grab rail	☐ Yes<br>☐ No	If Yes, funded by:<br>☐ provided by Social Services<br>☐ provided by hospital<br>☐ bought by you
1c.	Bariatric bed	☐ Yes<br>☐ No	If Yes, funded by:<br>☐ provided by Social Services<br>☐ provided by hospital<br>☐ bought by you
1d.	Bariatric shower stool	☐ Yes<br>☐ No	If Yes, funded by:<br>☐ provided by Social Services<br>☐ provided by hospital<br>☐ bought by you
1e.	Bariatric chair	☐ Yes<br>☐ No	If Yes, funded by:<br>☐ provided by Social Services<br>☐ provided by hospital<br>☐ bought by you
1f.	Wheel chair (bariatric)	☐ Yes<br>☐ No	If Yes, funded by:<br>☐ provided by Social Services<br>☐ provided by hospital<br>☐ bought by you
1g.	Mobility scooter	☐ Yes<br>☐ No	If Yes, funded by:<br>☐ provided by Social Services<br>☐ provided by hospital<br>☐ bought by you
1h.	Dressing aids	☐ Yes<br>☐ No	If Yes, funded by:<br>☐ provided by Social Services<br>☐ provided by hospital<br>☐ bought by you
1i.	Other	☐ Yes<br>☐ No	If Yes, please describe any other devices or specialist equipment you use.<br>_______________<br>_______________<br>Funded by:<br>☐ provided by Social Services<br>☐ provided by hospital<br>☐ bought by you
