# Supplementary material for: SurgiCal Obesity Treatment Study (SCOTS): a prospective, observational cohort study on health and socioeconomic burden in treatment-seeking individuals with severe obesity in Scotland, UK
Source: BMJ Open. 2021 Aug 26;11(8):e046441. doi: 10.1136/bmjopen-2020-046441 (PMC8395268; doi:10.1136/bmjopen-2020-046441)
Supplement: Supplementary data [file bmjopen-2020-046441supp005.pdf]

Supplementary Table 3. Surgical Obesity Treatment Study (SCOTS) baseline data by age group.

			Age (years)				
			Group 1	Group 2	Group 3	Group 4	Group 5
		Total Number of Participants Completing Question (N missing)	< 35 N=36	35-44 N=63	45-49 N=63	50-54 N=43	55+ N=44
**Demographics**	Sex: % Male	249 (0)	22.2%	22.2%	28.6%	30.2%	43.2%
	SIMD: % Q1 (Most Deprived)	247 (2)	40.0%	28.6%	14.5%	39.5%	27.3%
**Medical history**	% DVT	249 (0)	2.8%	3.2%	3.2%	0.0%	6.8%
	% Hypertension	249 (0)	11.1%	36.5%	52.4%	44.2%	63.6%
	% Type 2 Diabetes	249 (0)	30.6%	50.8%	54.0%	44.2%	63.6%
	% CVD	249 (0)	0.0%	6.3%	6.3%	9.3%	18.2%
	% Arthritis	249 (0)	5.6%	15.9%	36.5%	27.9%	59.1%
	% Back Problems	249 (0)	47.2%	50.8%	31.7%	39.5%	65.9%
	% Asthma	249 (0)	30.6%	22.2%	31.7%	20.9%	36.4%
	% Migraine	249 (0)	27.8%	17.5%	20.6%	18.6%	15.9%
	% Irritable Bowel Syndrome	249 (0)	8.3%	27.0%	23.8%	4.7%	15.9%
	% Sleep Apnoea	249 (0)	5.6%	20.6%	28.6%	25.6%	50.0%
	% PCOS	167 (10)	26.9%	26.1%	9.3%	7.1%	12.5%
	% Gastroesophageal Reflux	240 (9)	50.0%	50.0%	36.1%	28.6%	37.2%
**Incontinence**	% ICIQ-UI Score ≥6	239 (10)	18.2%	44.3%	50.8%	50.0%	47.6%
	% ICIQ-UI Score ≥6 (females only)	168 (9)	20.0%	51.1%	55.8%	62.1%	50.0%
	% ICIQ-UI Score ≥6 (males only)	71 (1)	12.5%	21.4%	38.9%	23.1%	44.4%
	Median (LQ, UQ) ICIQ-UI Score	239 (10)	0.0 (0.0; 4.0)	5.0 (0.0; 11.0)	6.0 (0.0; 11.0)	5.5 (3.0; 11.0)	4.5 (0.0; 11.0)
**Depression**	Mean (SD) PHQ-9 Score	244 (5)	9.0 (6.2)	10.3 (6.3)	9.2 (5.9)	9.1 (7.0)	10.2 (6.2)
	% PHQ-9 Score ≥10	244 (5)	42.9%	50.0%	37.7%	37.2%	51.2%
**Anxiety**	Median (LQ, UQ) GAD-7 Score	243 (6)	5.0 (2.0; 10.0)	6.0 (3.0; 12.0)	5.0 (3.0; 8.0)	5.0 (1.0; 11.0)	3.0 (1.0; 8.0)
	% GAD-7 Score ≥6	243 (6)	48.6%	54.8%	42.6%	47.6%	39.5%
**Smoking Status**	% Current	240 (9)	0.0%	6.5%	5.0%	0.0%	14.0%
	% Former		34.4%	45.2%	41.7%	48.8%	46.5%
	% Never		65.6%	48.4%	53.3%	51.2%	39.5%
**Quality of Life**							
SF-12	Mean (SD) SF-12 PCS	236 (13)	41.2 (11.4)	38 (10.9)	36.3 (11.8)	37.7 (11.9)	32.5 (10.1)
	Mean (SD) SF-12 MCS	236 (13)	44.9 (10.9)	44.5 (10.1)	46.2 (9.2)	44.7 (10.5)	46.8 (11.5)
EQ-5D-5L	Median (LQ, UQ) EQ-5D-5L Score	237 (12)	0.7 (0.6; 0.8)	0.6 (0.5; 0.8)	0.6 (0.5; 0.8)	0.7 (0.2; 0.8)	0.4 (0.2; 0.7)
	Mean (SD) EQ-5D-5L VAS	237 (12)	58.8 (20.7)	55.4 (21.7)	57.1 (21.7)	55.4 (25.8)	50.0 (20.4)
IWQOL-Lite (Standardised Scoring)	Mean (SD) Physical Function	243 (6)	49.5 (20.9)	54.0 (26.1)	57.2 (26.4)	57.7 (29.3)	66.0 (20.0)
	Median (LQ, UQ) Self Esteem	242 (7)	85.7 (67.9; 92.9)	85.7 (60.7; 96.4)	75.0 (53.6; 92.9)	71.4 (50.0; 92.9)	58.9 (39.3; 82.1)
	Mean (SD) Sexual Life	231 (18)	54.1 (26.5)	56.7 (32.4)	53.2 (33.7)	59.0 (31.9)	65.0 (31.2)
	Mean (SD) Public Distress	243 (6)	62.7 (25.8)	61.5 (26.0)	58 (26.8)	55.7 (31.7)	52.0 (25.6)
	Mean (SD) Work	236 (13)	44.3 (29.2)	42.7 (28.2)	38.8 (26.0)	47.3 (34.5)	47.1 (29.7)
	Mean (SD) Total Score	242 (7)	57.4 (18.8)	59.4 (21.6)	57.0 (22.7)	58.1 (26.0)	60.3 (18.2)

**Healthcare Utilisations**	% Use of Any Aids or Specialist Equipment	232 (17)	16.1%	14.5%	24.1%	42.5%	53.7%
**Social Security**	% Unable to Work Due to Illness or Disability	248 (1)	11.1%	17.5%	25.4%	28.6%	47.7%
	% Disability Living Allowance - Caring	236 (13)	9.4%	19.4%	17.2%	14.3%	31.0%
	% Disability Living Allowance - Mobility	236 (13)	12.5%	17.7%	17.2%	16.7%	35.7%

ICIQ-UI SF score: ≥6 = ‘moderate incontinence’ [1]
PHQ-9 scores: 0-4 = 'minimal depression'; 5-9 = 'mild depression'; 10-14 = 'moderate depression'; 15-19 = 'moderately severe depression'; 20-27 = 'severe depression' [2]
GAD-7 scores: 0-5 = 'mild anxiety'; 6-10 = 'moderate anxiety'; 11-15 = 'moderately severe anxiety'; 15-21 = 'severe anxiety' [3]

References

1. Avery K, Donovan J, Peters TJ, Shaw C, Gotoh M, Abrams P. ICIQ: A brief and robust measure for evaluating the symptoms and impact of urinary incontinence. *Neurourol Urodyn* 2004; **23**(4)**:** 322–330
2. Spitzer RL, Kroenke K, Williams JBW. Validation and utility of a self-report version of PRIME-MD: The PHQ Primary Care Study. *J Am Med Assoc* 1999; **282**(18): 1737-1744
3. Spitzer RL, Kroenke K, Williams JBW, Löwe B. A brief measure for assessing generalized anxiety disorder: The GAD-7. *Arch Intern Med* 2006; **166**(10): 1092-1097
