# Supplementary material for: SurgiCal Obesity Treatment Study (SCOTS): a prospective, observational cohort study on health and socioeconomic burden in treatment-seeking individuals with severe obesity in Scotland, UK
Source: BMJ Open. 2021 Aug 26;11(8):e046441. doi: 10.1136/bmjopen-2020-046441 (PMC8395268; doi:10.1136/bmjopen-2020-046441)
Supplement: Supplementary data [file bmjopen-2020-046441supp006.pdf]

Supplementary Table 4. Significant interactions with smoking as a two-level variable

Outcome	Model	Sub-population	Effect	OR (95% CI)	p-value
Use of aids or specialist equipment	Adjusted model as previously provided (smoke as 3-level)		Age	3.10 (1.94, 4.95)	<0.0001
	Adjusted model (smoke as 2-level)		Age	3.21 (2.01, 5.12)	<0.0001
	Adjusted model extended to include age*smoke and BMI*smoke		Age*smoke		0.044
			BMI*smoke		0.87
	Adjusted model, for the effect of Age in each of the smoking sub-populations	ANY Smoker	Age	2.35 (1.27, 4.36)	0.0065
		NEVER Smoker	Age	6.49 (2.63, 16.00)	<0.0001
PHQ-9 (assume included in the supplementary tables)	Adjusted model as previously provided (smoke as 3-level)		BMI	1.33 (0.96, 1.86)	0.0865
	Adjusted model (smoke as 2-level)		BMI	1.31 (0.94, 1.81)	0.1093
	Adjusted model extended to include age*smoke and BMI*smoke		Age*smoke		0.8800
			BMI*smoke		0.0156
	Adjusted model, for the effect of Age	ANY Smoker	BMI	0.88 (0.56, 1.37)	0.5679

	in each of the smoking sub-populations				
		NEVER Smoker	BMI	2.28 (1.31, 3.96)	0.0037

Unless otherwise noted, smoking status refers to a two-level variable [any smoking (current or former) and never smoked] as opposed to the three-level variable (current, former and never) used in previous analyses.

The sub-population model of never smokers for the use of aids and specialist equipment in the home outcome should be interpreted with caution due to small numbers of events.
