# Supplementary material for: SurgiCal Obesity Treatment Study (SCOTS): a prospective, observational cohort study on health and socioeconomic burden in treatment-seeking individuals with severe obesity in Scotland, UK
Source: BMJ Open. 2021 Aug 26;11(8):e046441. doi: 10.1136/bmjopen-2020-046441 (PMC8395268; doi:10.1136/bmjopen-2020-046441)
Supplement: Supplementary data [file bmjopen-2020-046441supp007.pdf]

Supplementary Table 5. Association of age and body mass index (BMI) with self-reported comorbidities and mental health indicators.

Comorbidities and Mental Health Indicators	Variable	BMI (Model 1[1]) and Age (Model 2[2])		BMI and Age (Model 3[3])	
		Odds Ratio (95% CI)	p-value	Odds Ratio (95% CI)	p-value
**Hypertension (N = 107)**	BMI	0.97 (0.72, 1.31)	0.84	0.89 (0.63, 1.24)	0.49
	Age	2.03 (1.47, 2.79)	<0.0001	2.02 (1.44, 2.85)	<0.0001
**Type 2 Diabetes (N = 124)**	BMI	0.94 (0.69, 1.26)	0.66	0.93 (0.67, 1.30)	0.68
	Age	1.41 (1.06, 1.87)	0.02	1.36 (1.00, 1.85)	0.05
**CVD (N = 20)**	BMI	0.61 (0.31, 1.18)	0.14	0.71 (0.36, 1.42)	0.34
	Age	2.98 (1.52, 5.85)	<0.01	2.27 (1.10, 4.67)	0.03
**Arthritis (N = 73)**	BMI	1.45 (1.05, 2.00)	0.02	1.62 (1.09, 2.40)	0.02
	Age	3.01 (2.00, 4.54)	< 0.0001	3.64 (2.26, 5.88)	< 0.0001
**Back Problems (N = 115)**	BMI	1.09 (0.81, 1.47)	0.57	1.12 (0.81, 1.54)	0.49
	Age	1.21 (0.91, 1.59)	0.19	1.12 (0.83, 1.51)	0.46
**Asthma (N = 70)**	BMI	1.85 (1.32, 2.59)	<0.001	1.81 (1.27, 2.58)	<0.01
	Age	1.12 (0.82, 1.52)	0.49	1.11 (0.79, 1.57)	0.54
**Migraine (N = 49)**	BMI	1.55 (1.09, 2.20)	0.02	1.63 (1.11, 2.38)	0.01
	Age	0.85 (0.60, 1.18)	0.33	0.91 (0.62, 1.33)	0.63
**Irritable Bowel Syndrome (N = 44)**	BMI	0.97 (0.66, 1.44)	0.90	1.00 (0.65, 1.54)	1.00
	Age	0.98 (0.69, 1.41)	0.93	1.07 (0.71, 1.60)	0.76
**Sleep Apnoea (N = 66)**	BMI	1.69 (1.21, 2.36)	<0.01	1.88 (1.27, 2.77)	<0.01
	Age	2.28 (1.55, 3.34)	< 0.0001	2.05 (1.35, 3.11)	<0.001
**PCOS (N = 28)**	BMI	0.72 (0.42, 1.23)	0.23	0.74 (0.42, 1.30)	0.29
	Age	0.56 (0.36, 0.88)	0.01	0.56 (0.35, 0.90)	0.02
**Gastroesophageal Reflux (N = 97)**	BMI	1.02 (0.75, 1.39)	0.88	1.06 (0.76, 1.47)	0.73
	Age	0.73 (0.55, 0.98)	0.04	0.68 (0.50, 0.93)	0.01
**Male Incontinence (ICIQ-UI score ≥ 6) (N = 22)**	BMI	1.58 (0.81, 3.09)	0.18	1.99 (0.92, 4.30)	0.08
	Age	2.05 (1.05, 4.01)	0.04	2.19 (1.06, 4.53)	0.03

**Female Incontinence (ICIQ-UI score ≥ 6) (N = 83)**	BMI	1.45 (1.01, 2.09)	0.05	1.40 (0.94, 2.08)	0.10
	Age	1.59 (1.10, 2.28)	0.01	1.49 (1.02, 2.19)	0.04
**Depression (PHQ-9 score ≥ 10) (N = 107)**	BMI	1.25 (0.92, 1.70)	0.15	1.33 (0.96, 1.86)	0.09
	Age	1.04 (0.79, 1.37)	0.79	0.99 (0.73, 1.34)	0.94
**Anxiety (GAD-7 score ≥ 6) (N = 114)**	BMI	1.42 (1.04, 1.95)	0.03	1.47 (1.05, 2.05)	0.03
	Age	0.91 (0.69, 1.20)	0.49	0.91 (0.67, 1.24)	0.56

[1] Model 1 – Unadjusted model including only the effect of BMI (per 10 kg/m^2) on comorbidity.

[2] Model 2 – Unadjusted model including only the effect of age (per 10 years) on comorbidity.

[3] Model 3 – Model including the effects of age (per 10 years) and BMI (per 10 kg/m^2) on comorbidity, after adjusting additionally for sex, Scottish Index of Multiple Deprivation (SIMD) and smoking status.

Regressions analyses were performed for conditions affecting 10 or more participants only.
