# Supplementary material for: SurgiCal Obesity Treatment Study (SCOTS): a prospective, observational cohort study on health and socioeconomic burden in treatment-seeking individuals with severe obesity in Scotland, UK
Source: BMJ Open. 2021 Aug 26;11(8):e046441. doi: 10.1136/bmjopen-2020-046441 (PMC8395268; doi:10.1136/bmjopen-2020-046441)
Supplement: Author's manuscript [file bmjopen-2020-046441.draft_revisions.pdf]

BMJ Open

BMJ Open is committed to open peer review. As part of this commitment we make the peer review history of every article we publish publicly available.

When an article is published we post the peer reviewers' comments and the authors' responses online. We also post the versions of the paper that were used during peer review. These are the versions that the peer review comments apply to.

The versions of the paper that follow are the versions that were submitted during the peer review process. They are not the versions of record or the final published versions. They should not be cited or distributed as the published version of this manuscript.

BMJ Open is an open access journal and the full, final, typeset and author-corrected version of record of the manuscript is available on our site with no access controls, subscription charges or pay-per-view fees (http://bmjopen.bmj.com).

If you have any questions on BMJ Open's open peer review process please email info.bmjopen@bmj.com

BMJ Open

Health and socio-economic burden in treatment-seeking individuals with severe obesity: profile of the Surgical Obesity Treatment Study (SCOTS) national cohort

Journal:	*BMJ Open*
Manuscript ID	bmjopen-2020-046441
Article Type:	Original research
Date Submitted by the Author:	02-Nov-2020

Complete List of Authors:	Mackenzie, Ruth; University of Glasgow Institute of Cardiovascular and Medical Sciences, BHF Glasgow Cardiovascular Research Centre<br>Greenlaw, Nicola; University of Glasgow Robertson Centre for Biostatistics<br>Ali, Abdulmajid; University Hospital Ayr<br>Bruce, Duff; NHS Grampian<br>Bruce, Julie; University of Warwick Warwick Clinical Trials Unit<br>Grieve, Eleanor; University of Glasgow, Institute of Health and Wellbeing<br>Lean, Mike; University of Glasgow School of Medicine Dentistry and Nursing<br>Lindsay, Robert; University of Glasgow Institute of Cardiovascular and Medical Sciences, BHF Glasgow Cardiovascular Research Centre<br>Sattar, Naveed; University of Glasgow, Institute of Cardiovascular and Medical Sciences<br>Stewart, Sally; Glasgow Caledonian University, School of Health and Life Sciences<br>Ford, Ian; University of Glasgow Robertson Centre for Biostatistics<br>Logue, Jennifer; Lancaster University, Lancaster Medical School

Keywords:	EPIDEMIOLOGY, Adult surgery < SURGERY, Quality in health care < HEALTH SERVICES ADMINISTRATION & MANAGEMENT

SCHOLARONE™ Manuscripts

I, the Submitting Author has the right to grant and does grant on behalf of all authors of the Work (as defined in the below author licence), an exclusive licence and/or a non-exclusive licence for contributions from authors who are: i) UK Crown employees; ii) where BMJ has agreed a CC-BY licence shall apply, and/or iii) in accordance with the terms applicable for US Federal Government officers or employees acting as part of their official duties; on a worldwide, perpetual, irrevocable, royalty-free basis to BMJ Publishing Group Ltd ("BMJ") its licensees and where the relevant Journal is co-owned by BMJ to the co-owners of the Journal, to publish the Work in this journal and any other BMJ products and to exploit all rights, as set out in our licence.

The Submitting Author accepts and understands that any supply made under these terms is made by BMJ to the Submitting Author unless you are acting as an employee on behalf of your employer or a postgraduate student of an affiliated institution which is paying any applicable article publishing charge ("APC") for Open Access articles. Where the Submitting Author wishes to make the Work available on an Open Access basis (and intends to pay the relevant APC), the terms of reuse of such Open Access shall be governed by a Creative Commons licence – details of these licences and which Creative Commons licence will apply to this Work are set out in our licence referred to above.

Other than as permitted in any relevant BMJ Author's Self Archiving Policies, I confirm this Work has not been accepted for publication elsewhere, is not being considered for publication elsewhere and does not duplicate material already published. I confirm all authors consent to publication of this Work and authorise the granting of this licence.

For peer review only

TITLE:

Health and socio-economic burden in treatment-seeking individuals with severe obesity: profile of the Surgical Obesity Treatment Study (SCOTS) national cohort

AUTHORS:

Ruth M. Mackenzie[1], Nicola Greenlaw[2], Abdulmajid Ali[3], Duff Bruce[4], Julie Bruce[5], Eleanor Grieve[6], Mike Lean[7], Robert S. Lindsay[1], Naveed Sattar[1], Sally Stewart[8], Ian Ford[2], Jennifer Logue[9], on behalf of the SCOTS investigators

AFFILIATIONS:

[1]Institute of Cardiovascular and Medical Sciences, University of Glasgow, Glasgow, UK.

[2]Robertson Centre for Biostatistics, University of Glasgow, Glasgow, UK.

[3]University Hospital Ayr, NHS Ayrshire and Arran, Ayr, UK.

[4]NHS Grampian, Aberdeen, UK.

[5]Clinical Trials Unit, University of Warwick, Warwick, UK.

[6]Institute of Health and Wellbeing, University of Glasgow, Glasgow, UK.

[7]School of Medicine, Dentistry and Nursing, University of Glasgow, Glasgow, UK

[8]School of Health and Life Sciences, Glasgow Caledonian University, Glasgow, UK.

[9]Lancaster Medical School, Lancaster University, Lancaster, UK.

For peer review only

CORRESPONDENCE TO:

Jennifer Logue

Lancaster Medical School

Faculty of Health and Medicine

Lancaster University

LA1 4YG

Telephone: 01524 595133

j.logue1@lancaster.ac.uk

WORD COUNT:

4,299 (including strengths and limitations of this study, funding, competing interests, author contribution and acknowledgements statements)

3,913 (excluding strengths and limitations of this study, funding, competing interests, author contribution and acknowledgements statements)

For peer review only

For peer review only - http://bmjopen.bmj.com/site/about/guidelines.xhtml

ABSTRACT

Objectives: There is a lack of evidence to inform the delivery and follow-up of bariatric surgery for people with severe obesity. The SurgiCal Obesity Treatment Study (SCOTS) is a national longitudinal cohort of people undergoing bariatric surgery. Here we describe characteristics of the recruited SCOTS cohort, and the relationship between health and socio-economic status with body mass index (BMI) and age.

Participants/Methods: 445 participants scheduled for bariatric surgery at any of 14 centres in Scotland, UK were recruited between 2013 and 2016 for this longitudinal cohort study (1 withdrawal); 249 completed health-related pre-operative patient reported outcome measures. Regression models were used to estimate the effect of a 10-unit increase in age or BMI, adjusting for BMI, sex, smoking and socio-economic status.

Results: Mean age was 46 years and median BMI was 47 kg/m^2. For each 10 kg/m^2 higher BMI there was a change of -5.21 (95% CI -6.90, -3.52; p<0.0001) in Rand 12-item Short Form Survey Physical Component Summary (SF-12 PCS), -0.11 (95% CI -0.16, -0.06; p<0.0001) in EuroQoL 5-level EQ-5D version index score, 14.20 (95% CI 10.69, 17.70; p<0.0001) in Impact of Weight on Quality of Life-Lite Physical Function score. We observed a 3.1 times increased use of specialist aids and equipment at home (OR 1.94, 95% CI 1.94, 4.95; p < 0.0001). Broadly similar results were seen for each 10-year higher age, including a change of -2.14 (95% CI -3.73, -0.54; p<0.01) in SF-12 PCS.

Conclusions: Higher BMI combined with older age is associated with poor physical functioning and quality of life in people seeking bariatric surgery treatment. Policy makers must consider the health and care needs of these individuals and invest to provide increased access to effective weight management.

For peer review only

Trial registration: This study was registered prospectively at the International Standard Randomised Controlled Trials Number registry: ISRCTN47072588

STRENGTHS AND LIMITATIONS OF THIS STUDY

- A limitation of this study is the fact that selection for bariatric surgery is often based on the presence of comorbidity so these results, while applicable to a treatment-seeking population, may not be directly applicable to the population with severe obesity in general.
- The fact that almost every bariatric surgery patient in Scotland was recruited is a major strength of this study and renders it more representative than many studies undertaken in the field.
- A further strength of this study is that the majority of questionnaires used were externally validated and wide-ranging, containing a number of unique questions covering medical, social, psychological and physical domains.

INTRODUCTION

Obesity (body mass index [BMI] ≥ 30kg/m^2) and, in particular, severe obesity (BMI ≥ 40kg/m^2) are associated with a variety of negative health outcomes, including increased risk of most major chronic diseases(1). In recent years, severe obesity has emerged as a major public health concern with rates increasing rapidly in a number of countries across the world, including the United States (US) where the prevalence of BMI > 40 kg/m^2 rose by 70% between 2000 and 2010(2), and around 7.7% of adults are now considered to have severe obesity(3). Similarly, levels of severe obesity have risen in the United Kingdom (UK) with 2.9% of all adults in England(4) and 4% of all adults in Scotland now estimated to have a BMI ≥ 40 kg/m^2(5).

For peer review only

As the prevalence of severe obesity rises, effective treatment is a priority. The efficacy of bariatric surgery for significant long-term weight loss is well established(6,7). However, for the UK National Health Service (NHS), like many other health systems, the commissioning of bariatric surgery is low priority. Indeed, despite obesity prevalence being amongst the highest in the European Union, the UK performs only nine bariatric surgery procedures per 100 000 people(8,9) while Sweden, a country with a similar health service but lower obesity prevalence, performs 70-80 procedures per 100 000 people(10). In North America, the rate of surgery is around 40-50 per 100 000 people, with the majority of these operations performed in the US(8).

The low prioritisation of bariatric surgery within the UK and the strict criteria for access to surgery, including complex pre-surgical pathways and pre-surgical weight-loss requirements(5), results in low numbers of individuals with severe obesity actually receiving surgery. Those receiving surgery generally do so after many years of alternative conservative interventions, at a point when their mean BMI is extremely high, at around 45 kg/m^2, and they are at a median age of 47 years(11). To date, it is unclear how this delay in treatment impacts on health, physical functioning and quality of life.

The SurgiCal Obesity Treatment Study (SCOTS) is the first national epidemiological study established to investigate long-term outcomes following bariatric surgery. This cohort study collected clinical and patient-reported health outcomes from treatment-seeking individuals from across Scotland with severe obesity before they underwent bariatric surgery(12). The aim of the present study is to describe the health-related characteristics of the recruited SCOTS cohort and to examine relationships between age, pre-operative BMI and other health-related factors in our recruited sample of adults with severe obesity from across Scotland.

PARTICIPANTS AND METHODS

For peer review only - http://bmjopen.bmj.com/site/about/guidelines.xhtml

This study was registered prospectively at the International Standard Randomised Controlled Trials Number (ISRCTN) registry: ISRCTN47072588. In reporting our findings, we have adhered to the STROBE (STrengthening the Reporting of OBservational studies in Epidemiology) Statement(13) (Supplementary Table 1).

Ethics

A favourable ethical opinion for this study was obtained from the West of Scotland Research Ethics Committee 4 on 07/02/2013 (13/WS/0005).

Patient Involvement

Patients identified via bariatric surgery peer support groups in Scotland were involved in the design and conduct of this research. During the protocol development stage, patients provided input with regard to data collection and defining research questions. In addition, methods of recruitment were informed by discussions with patients during two focus group sessions. Patients were subsequently involved in reviewing paperwork for recruitment and appeared in the recruitment video. There is also a patient member of the independent study steering committee and patients were invited to a meeting to discuss plans for dissemination of study results.

Study Design

SCOTS is a national, prospective, observational cohort study of adults aged over 16 years who were eligible for primary bariatric surgery in Scotland. Participants were recruited over a four year period from December 2013 to February 2017. Recruitment to the cohort has now closed although follow-up will continue until October 2020. A detailed protocol for the SCOTS study was published previously(12) and is included as Supplementary File 1, but was amended in 2016 to reflect a smaller cohort size and shorter follow-up. This was in response to changes in national service commissioning contributing to lower numbers of bariatric surgery which, in turn, affected recruitment potential and planned sample size. Any changes from the original protocol are outlined below.

For peer review only

Participant and Centre Eligibility

As outlined in the protocol(12) (Supplementary File 1), patients scheduled to undergo a primary bariatric surgery procedure at any of the ten NHS-funded or four private hospitals in Scotland providing this surgery were eligible for invitation to the study. Inclusion criteria were that patients were aged 16 years or over and undergoing their first bariatric surgery procedure. Patients were required to have capacity to consent, provide written informed consent and be resident in Scotland. Patients who did not meet these criteria or who did not have English language skills to complete written questionnaires were ineligible(12) (Supplementary File 1).

For peer review only

Recruitment Procedures and Consent

Patients were approached about the study at least four weeks prior to their primary bariatric surgical procedure by the clinical bariatric surgery team or by a research nurse in preoperative assessment clinics. Written signed consent for access to medical records, linkage of electronic health records, and for postal questionnaires was obtained on a subsequent clinical visit.

Data Collection

Recruited participants were asked to complete questionnaires preoperatively and at two and three years postoperatively. Completion could be either by post or electronically via a secure link sent by email. Two reminders were sent by the participant's chosen method and a third reminder, if required, was sent by post to all participants. No further strategy was used after three reminders.

Baseline preoperative questionnaires collected health-related information, including weight, medical history, smoking status, alcohol use, gastrointestinal symptoms, urological health, depression, anxiety, health-related quality of life (HRQoL) and obesity-specific quality of life (O-QoL), life optimism, physical activity, healthcare utilisation, employment, and social security. Comorbidity was assessed by self-report using a questionnaire designed specifically for this study (Supplementary File 2). Gastrointestinal symptoms were evaluated using a questionnaire developed for the REFLUX

For peer review only - http://bmjopen.bmj.com/site/about/guidelines.xhtml

trial(14). Urological health was assessed using the International Prostate Symptom Score (IPSS)(15) and the International Consultation on Incontinence Questionnaire-Urinary Incontinence Short Form (ICIQ-UI SF) wherein a score ≥ 6 indicates moderate incontinence(16). Female reproductive health data was obtained using a modified version of the questionnaire developed for the Longitudinal Assessment of Bariatric Surgery (LABS-2) study(17). Information on male erectile dysfunction was obtained using a modified version of the questionnaire developed for the Massachusetts Male Ageing Study(18). Anxiety and depression were assessed using the Generalised Anxiety Disorder Assessment (GAD-7)(19) and Patient Health Questionnaire (PHQ)-9 (PHQ-9)(20) instruments respectively. A PHQ-9 score ≥ 10 is indicative of moderate to severe depression while a GAD-7 score ≥ 6 is reflective of moderate to severe anxiety(19,20). Smoking status was ascertained using a questionnaire specifically developed for this study (Supplementary File 2). Alcohol use was determined using a modified version of the Alcohol Use Disorders Identification Test (AUDIT)(21). HRQoL was assessed using the Rand 12-item Short Form Survey (SF-12)(22) and EuroQoL 5-level EQ-5D version (EQ-5D-5L)(23,24) instruments while O-QoL was assessed using the Impact of Weight on Quality of Life-Lite (IWQOL-Lite) questionnaire(25). Standardised scoring was used when interpreting IWQOL-Lite questionnaires(26). Life optimism was determined using a modified version of the Life Orientation Test (LOT) wherein a score range of 0-13 reflects low optimism (high pessimism)(27). Data on physical activity was obtained using the International Physical Activity Questionnaire (IPAQ) – Short Form(28). Information on participants' employment, social security status, and healthcare utilisation was obtained using questionnaires specifically developed for this study (Supplementary File 2).

Each participant's quintile of the Scottish Index of Multiple Deprivation (SIMD), an area-based measure of socioeconomic status(29), was derived from their postcode. Combining a number of indicators of socioeconomic status across seven domains, the SIMD provides a relative measure of deprivation which can be used to compare data zones by ranking them from most to least deprived.

For peer review only - http://bmjopen.bmj.com/site/about/guidelines.xhtml

The seven domains include income, employment, health, education, skills and training, housing, geographic access, and crime(29).

Height and weight data were collected by clinical staff at recruitment allowing BMI to be calculated.

We herein report baseline data recorded during the recruitment visit for the whole recruited cohort before bariatric surgery, and patient-reported outcomes for the sub-set completing baseline questionnaires.

Statistical Analyses

Continuous data are reported as means and standard deviations (SD) or medians and lower (LQ) and upper (UQ) quartiles depending on data distribution, and counts and percentages are reported for categorical data. Age and BMI data were categorised *a priori*; BMI (kg/m^2) is reported as 5-unit bands (< 40, 40-44, 45-49, 50-54, ≥ 55) and age was categorised using 10-year age bands (< 35 years, 35-44, 45-49, 50-54 and ≥ 55 years). Other baseline demographics are summarised by group.

Linear regression models were used to examine continuous quality of life measures and logistic regression for the binary 'need for specialist aids' and 'equipment in the home to assist with daily living'. Models were used to estimate the effect of a 10 unit increase in age or BMI in 1) an unadjusted model and 2) in a model adjusted for sex, SIMD, smoking status and BMI or age respectively as these factors may have an effect on the prevalence of obesity and related comorbidity. Regression model effect estimates, or odds ratios (ORs), and corresponding 95% confidence intervals (CIs) and associated p-values are provided. Data was analysed as available, without any imputation for missing data. All analyses were performed using SAS (version 9.3).

RESULTS

Over the three year recruitment period, a total of 548 patients were approached and screened for eligibility to participate. Of these, 103/548 (19%) were excluded or declined to participate (Figure 1).

For peer review only - http://bmjopen.bmj.com/site/about/guidelines.xhtml

For peer review only

We recruited 445/548 (81%) participants but one participant withdrew consent leaving a recruited sample of 444 (81%). Of the recruited sample, 413/444 (93%) consented to data linkage and questionnaire follow-up whilst 31/444 (7%) consented to data linkage only. Of these 413 participants, a total of 164/413 (40%) were not included in the subsequent analysis: 129 did not return a baseline questionnaire and 35 had bariatric surgery before their baseline patient reported outcome measures (PROMs) questionnaires were completed. Of the 129 who did not return baseline questionnaires, 84/129 (65%) progressed to surgery, 43/129 (33%) did not progress to surgery and the status of 2/129 (2%) was unknown. Completed preoperative baseline PROMs data for 249/413 participants (60% of those consented) were available for analysis (Figure 1).

Characteristics of recruited and analysed sample

Demographic data are summarised in Table 1. Participant characteristics were similar between the total recruited sample (n= 444) and the analysed sub-set (n = 249) with completed PROMS before bariatric surgery (Table 1). For both samples, mean age was 46 years with a higher proportion of women than men (71% vs 29%). Half of recruited participants were aged 35 to 49 years, with one third being over 50 years. The median BMI was 47 kg/m^2 with more than 21% having a BMI of ≥ 55+ kg/m^2. Over half of the participants (55%) lived in areas of high socio-economic deprivation (SIMD quintiles 1 and 2).

For peer review only

For peer review only - http://bmjopen.bmj.com/site/about/guidelines.xhtml

		Recruited Sample N = 444	Analysed Sample* N = 249
Sex, N (%)	Male	123 (27.7)	72 (28.9)
	Female	321 (72.3)	177 (71.1)
	Missing	0	0
Age (years)	Mean (SD)	46.2 (9.1)	45.9 (9.1)
	Missing	0	0
Age Group, N (%)	< 35 years	61 (13.7)	36 (14.5)
	35-44 years	116 (26.1)	63 (25.3)
	45-49 years	109 (24.5)	63 (25.3)
	50-54 years	79 (17.8)	43 (17.3)
	55+ years	79 (17.8)	44 (17.7)
	Missing	0	0
BMI (kg/m^2)	Median (IQR)	47.2 (42.7 ; 53.6)	47.6 (42.8 ; 53.8)
	Missing	1	0
BMI Group, N (%)	BMI < 40	52 (11.7)	24 (9.6)
	BMI 40-44	115 (26.0)	64 (25.7)
	BMI 45-49	116 (26.2)	64 (25.7)
	BMI 50-54	71 (16.0)	44 (17.7)
	BMI 55+	89 (20.1)	53 (21.3)
	Missing	1	0
SIMD Quintile, N (%)	Quintile 1 (most deprived)	135 (30.5)	70 (28.3)
	Quintile 2	108 (24.4)	65 (26.3)
	Quintile 3	84 (19.0)	51 (20.6)
	Quintile 4	68 (15.4)	34 (13.8)
	Quintile 5 (least deprived)	47 (10.6)	27 (10.9)
	Missing	2	2
Marital Status, N (%)	Married/civil partnership/co-habiting	Not collected	155 (63)
	Single/separated/divorced/ widowed		91 (37)
	Missing		3
Ethnic Group, N (%)	White	Not collected	243 (97.6)
	Mixed		4 (1.6)
	Asian/Asian Scottish/Asian British		1 (0.4)
	African Caribbean/Black		1 (0.4)
	Other		0 (0.0)
	Missing		0
Education, N (%)	School only	Not collected	58 (23.5)
	Formal qualifications through training at work		54 (21.9)
	Qualification (other than a degree from college or university)		64 (25.9)
	Degree from college or university		71 (28.7)
	Missing		2
Current Employment Status, N (%)	Working full time	Not collected	124 (50.0)
	Working part time		24 (9.7)

For peer review only

	Unable to work because of illness or disability		64 (25.8)
	Student/unemployed and seeking employment/ unemployed and not seeking employment/ carer/other		36 (14.5)
	Missing		1

Table 1. Baseline sociodemographic characteristics of recruited (n = 444) and analysed (n = 249) samples of SCOTS participants. Working full time = ≥ 30 hours per week; working part-time = < 30 hours per week. Percentages may not total 100% in all cases due to rounding. *Participants who returned baseline questionnaires prior to their bariatric surgery are included in the analysed sample.

For peer review only

For peer review only - http://bmjopen.bmj.com/site/about/guidelines.xhtml

Comorbidities

For the analysed sample (n = 249), self-reported medical comorbidities, and physical, mental and functional measures are presented in Table 2. Over 40% reported having at least one of hypertension, type 2 diabetes (T2D), back problems, anxiety/depression and gastroesophageal reflux. Over 60% of the cohort reported more than three comorbidities. Over 40% of male participants reported erectile dysfunction while one third of males described urinary incontinence. Half of female participants reported urinary incontinence. Mean depression scores reflected mild depression, although 44% of participants had scores indicating moderate to severe depression. Anxiety scores for all participants were indicative of mild anxiety (median 5.0); with half of participants having scores indicative of moderate to severe anxiety. The mean life optimism score for participants was reflective of low optimism (high pessimism). Very few participants smoked (5%) and, on average, alcohol consumption was moderate.

For peer review only

For peer review only - http://bmjopen.bmj.com/site/about/guidelines.xhtml

Table 2. Preoperative health-related characteristics of N = 249 SCOTS participants undergoing bariatric surgery

		N = 249<br>N (%)	Missing<br>N (%)
**Comorbidity, Self-report**	DVT	8 (3.2)	0
	Pulmonary embolism	4 (1.6)	0
	Hypertension	107 (43.0)	0
	Type 2 diabetes	124 (49.8)	0
	Angina/heart attack	17 (6.8)	0
	Heart failure	2 (0.8)	0
	Stroke/mini stroke	6 (2.4)	0
	Arthritis	73 (29.3)	0
	Back problems	115 (46.2)	0
	Chronic bronchitis	4 (1.6)	0
	Eczema/psoriasis	33 (13.3)	0
	Asthma	70 (28.1)	0
	Thyroid problems	32 (12.9)	0
	Migraine	49 (19.7)	0
	Anxiety/depression	114 (45.8)	0
	Kidney disease	7 (2.8)	0
	Liver disease	2 (0.8)	0
	Cancer	4 (1.6)	0
	Irritable bowel syndrome	44 (17.7)	0
	Sleep apnoea	66 (26.5)	0
	CVD	20 (8.0)	0
**N (%) Self-reported Comorbidities**	None	9 (3.6)	0
	1-2	80 (32.1)	0
	≥ 3	160 (64.3)	0
**Gastrooesophageal Reflux**	Yes	97 (40.4)	9 (3.6)

For peer review only

**Female Reproductive Health N = 75***	Mean (SD) age years, last natural menstrual period	39.4 (10.9)	4 (5.3)
**Female Reproductive Health N = 177**	PCOS, N (%)	28 (16.8)	10 (5.6)
**Male Reproductive Health N = 72**	Impotence, N (%)	28 (41.2)	4 (5.6)
	IPSS score ≥8, N (%)	34 (47.9)	1 (1.4)
**Incontinence**	Median (LQ, UQ) ICIQ-UI SF score	4 (0.0; 10.0)	10 (4.0)
	ICIQ-UI SF score ≥6	105 (43.9)	10 (4.0)
Incontinence, Females N = 177	ICIQ-UI SF score ≥6, N (%)	83 (49.4)	9 (5.1)
Incontenence, Males N = 72	ICIQ-UI SF score ≥6, N (%)	22 (31.0)	1 (1.4)
**Depression**	Mean (SD) PHQ-9 score	9.6 (6.3)	5 (2.0)
	N (%) PHQ-9 score ≥10	107 (43.9)	5 (2.0)
**Anxiety**	Median (LQ, UQ) GAD-7	5 (2.0; 9.0)	6 (2.4)
	N (%) GAD-7 score ≥6	114 (46.9)	6 (2.4)
**Smoking Status**	Current	13 (5.4)	9 (3.6)
	Former	105 (43.8)	
	Never	122 (50.8)	
**Alcohol Use**	Median (LQ, UQ) AUDIT	3 (1.0; 6.0)	20 (8.0)
**Quality of Life**			
SF-12	Mean (SD) PCS	37 (11.4)	13 (5.2)
	Mean (SD) MCS	45.5 (10.3)	13 (5.2)
EQ-5D-5L	Median (LQ, UQ)	0.6 (0.3; 0.8)	12 (4.8)
	Mean (SD) VAS	55.3 (22.1)	12 (4.8)
IWQOL-Lite (Standardised Scoring)	Mean (SD) Physical Function	56.9 (25.4)	6 (2.4)
	Mean (SD) Self Esteem	70.7 (27.1)	7 (2.8)
	Mean (SD) Sexual Life	57.1 (31.7)	18 (7.2)

For peer review only

	Mean (SD) Public Distress	58.1 (27.2)	6 (2.4)
	Mean (SD) Work	43.6 (29.2)	13 (5.2)
	Mean (SD) Total score	58.5 (21.7)	7 (2.8)
**Life Optimism**	Mean (SD) LOT score	13 (4.9)	14 (5.6)
**Physical Activity**	≥1 walking, moderate or vigorous activity in last 7 days	201 (83.4)	8 (3.2)
	Median (LQ, UQ) IPAQ score**	720 (40.0; 1800.0)	6 (3.0)
**Healthcare Utilisations**	Using any aids or specialist equipment	67 (28.9)	17 (6.8)
	Median (LQ, UQ) GP visits in last 3 months	2 (1.0; 3.0)	79 (31.7)
	Median (LQ, UQ) visits to other health/social care providers in last 3 months	3 (1.0; 5.0)	73 (29.3)
**Social Security**	Unable to work due to illness or disability	64 (25.8)	1 (0.4)
	Receiving Disability Living Allowance (caring)	44 (18.6)	13 (5.2)
	Receiving Disability Living Allowance (mobility)	47 (19.9)	13 (5.2)

For peer review only

Table 2. Preoperative health-related characteristics of N = 249 SCOTS participants undergoing bariatric surgery. DVT, deep vein thrombosis; CVD, cardiovascular disease; PCOS, polycystic ovary syndrome; GP, general practitioner. IPSS Scores: 0-7 = 'Mild symptoms'; 8-19 = 'Moderate symptoms'; 20-35 = 'Severe symptoms' [15]. ICIQ-UI SF score: ≥6 = 'moderate incontinence' [16]. PHQ-9 scores: 0-4 = 'minimal depression'; 5-9 = 'mild depression'; 10-14 = 'moderate depression'; 15-19 =

For peer review only - http://bmjopen.bmj.com/site/about/guidelines.xhtml

'moderately severe depression'; 20-27 = 'severe depression' [20]. GAD-7 scores: 0-5 = 'mild anxiety'; 6-10 = 'moderate anxiety'; 11-15 = 'moderately severe anxiety'; 15-21 = 'severe anxiety'[19]. * 75/177 (42%) female participants reported not menstruating in the last 12 months. ** MET minutes/week.

For peer review only

Health and obesity-related quality of life

Mean SF-12 Physical Component Summary (PCS) and Mental Component Summary (MCS) scores were low; PCS 37.0 (11.4), MCS 45.5 (10.3). The median EQ-5D-5L score of sample participants was 0.6 (0.3; 0.8) while the mean EQ-5D-5L visual analogue scale (VAS) was 55.3 (22.1). Participants had a mean IWQOL-Lite Physical Function score of 56.9 (25.4) and a mean total score of 58.5 (21.7) (Table 2) where an increase in IWQOL-Lite score indicates a worsening in quality of life.

Physical activity

Over 80% of SCOTS participants reported undertaking at least 10 minutes of either walking, moderate or vigorous activity in the last seven days and the median IPAQ score for the cohort was 720.0 MET minutes/week. Almost one third (29%) of participants reported using aids or specialist equipment to assist with their daily activities in the home (Table 2).

Comorbidity by BMI and age

Comorbidity data is presented by BMI group and age group in Supplementary Tables 2 and 3 respectively. In order to further investigate the associations between BMI and age on physical, mental and functional measures, and healthcare utilisation within the SCOTS population, regression analyses were performed (Table 3). Higher BMI values and higher ages were negatively associated with physical, but not mental, HRQoL scores (Table 3). For each 10 kg/m^2 higher BMI there was a change of -5.21 (95% CI -6.90, -3.52; p<0.0001) in SF-12 PCS, -0.11 (95% CI -0.16, -0.06; p<0.0001) in EQ-5D-5L score and 14.20 (95% CI 10.69, 17.70; p<0.0001) in IWQOL-Lite Physical Function score (where an increase in score indicates a worsening). We observed a 3.10 times increased use of specialist aids and equipment in the home (OR 1.94, 95% CI 1.94, 4.95; p < 0.0001), adjusting for age, sex, smoking and socio-economic deprivation. For each 10-year higher age, there was a change of -2.14 (95% CI -3.73, -0.54; p<0.01) in SF-12 PCS score, -0.07 (95% CI -0.11, -0.02; p<0.01) in EQ-5D-5L score and 5.01 (95% 1.75, 8.27; p<0.01) in IWQOL-Lite Physical Function score, and a 3.40 (OR 1.94,

For peer review only

For peer review only - http://bmjopen.bmj.com/site/about/guidelines.xhtml

95% CI 1.94, 4.95; p<0.0001) times increased use of specialist aids or equipment in the home, adjusting for BMI, sex, smoking and socio-economic status.

With regard to medical comorbidities, as shown in Supplementary Table 4, increasing BMI had a significant association with increased prevalence of asthma in the SCOTS population, while older age was associated with increased prevalence of hypertension, arthritis and sleep apnoea (Supplementary Table 4).

For peer review only

For peer review only - http://bmjopen.bmj.com/site/about/guidelines.xhtml

Table 3. Association of age and body mass index (BMI) with quality of life, use of specialist equipment in the home and social security.

Quality of Life Indicators and Functional Measures	Variable	BMI (Model 1[1]) and Age (Model 2[2])		BMI and Age (Model 3[3])	
		Regression Coefficient (95% CI)[4]	p-value	Regression Coefficient (95% CI)[4]	p-value
**SF-12 PCS**	BMI	-4.91 (-6.55, -3.28)	<0.0001	-5.21 (-6.90, -3.52)	<0.0001
	Age	-2.44 (-4.03, -0.85)	<0.01	-2.14 (-3.73, -0.54)	<0.01
**SF-12 MCS**	BMI	-0.49 (-2.07, 1.09)	0.54	-0.40 (-2.06, 1.26)	0.64
	Age	0.42 (-1.04, 1.87)	0.58	0.68 (-0.89, 2.25)	0.39
**EQ-5D-5L Score**	BMI	-0.11 (-0.15, -0.06)	<0.0001	-0.11 (-0.16, -0.06)	<0.0001
	Age	-0.08 (-0.12, -0.03)	<0.001	-0.07 (-0.11, -0.02)	<0.01
**EQ-5D-5L VAS**	BMI	-7.08 (-10.38, -3.79)	<0.0001	-7.73 (-11.10, -4.36)	<0.0001
	Age	-2.19 (-5.30, 0.92)	0.17	-0.65 (-3.77, 2.46)	0.68
**IWQOL-Lite Physical function**	BMI	13.72 (10.28, 17.17)	<0.0001	14.20 (10.69, 17.70)	<0.0001
	Age	5.77 (2.28, 9.26)	<0.01	5.01 (1.75, 8.27)	<0.01
**IWQOL-Lite Self Esteem**	BMI	5.09 (1.02, 9.16)	0.01	5.99 (1.86, 10.12)	<0.01
	Age	-4.74 (-8.51, -0.97)	0.01	-5.11 (-8.96, -1.25)	<0.01
**IWQOL-Lite Sexual Life**	BMI	5.56 (0.71, 10.41)	0.02	5.74 (0.82, 10.67)	0.02
	Age	3.86 (-0.75, 8.47)	0.10	3.01 (-1.71, 7.73)	0.21
**IWQOL-Lite Public Distress**	BMI	15.36 (11.72, 19.00)	<0.0001	16.07 (12.34, 19.80)	<0.0001
	Age	-2.78 (-6.57, 1.02)	0.15	-3.04 (-6.51, 0.44)	0.09
**IWQOL-Lite Work**	BMI	9.54 (5.25, 13.84)	<0.0001	9.59 (5.16, 14.02)	<0.0001
	Age	1.95 (-2.25, 6.16)	0.36	1.04 (-3.15, 5.24)	0.62
**IWQOL Total score**	BMI	10.31 (7.29, 13.33)	<0.0001	10.88 (7.79, 13.97)	<0.0001

For peer review only

	Age	1.20 (-1.84, 4.24)	0.44	0.55 (-2.33, 3.43)	0.71
**Use of Aids or Specialist Equipment**	BMI	2.34 (1.62, 3.39)*	<0.0001	3.10 (1.94, 4.95)*	<0.0001
	Age	2.65 (1.76, 4.00)*	<0.0001	3.40 (1.94, 4.95)*	<0.0001
**Disability Living Allowance (Caring)**	BMI	1.19 (0.82, 1.73)*	0.37	1.07 (0.72, 1.59)*	0.74
	Age	1.54 (1.03, 2.31)*	0.03	1.62 (1.08, 2.46)*	0.02
**Disability Living Allowance (Mobility)**	BMI	1.18 (0.82, 1.70)*	0.39	1.13 (0.77, 1.65)*	0.55
	Age	1.64 (1.10, 2.45)*	0.01	1.62 (1.08, 2.44)*	0.02

Table 3. Association of age and body mass index (BMI) with quality of life, use of specialist equipment in the home and social security. [1] Model 1 – Unadjusted model including only the effect of BMI (per 10 kg/m^2) on comorbidity. [2] Model 2 – Unadjusted model including only the effect of age (per 10 years) on comorbidity. [3] Model 3 – Model including the effects of age (per 10 years) and BMI (per 10 kg/m^2) on comorbidity, after adjusting additionally for sex, Scottish Index of Multiple Deprivation (SIMD) and smoking status. [4] Regression coefficient (95% CI) is Estimate (95% CI) for results from the linear regression and Odds Ratio (95% CI) for results from the logistic regression. Odds Ratio results are indicated with a *. N ≥ 10 for all indicators and measures for which regression analyses were performed.

For peer review only

DISCUSSION

Despite escalating levels of severe obesity in Western society and the concomitant increase in bariatric surgical procedures being performed in some countries(30), there is a dearth of information on the health status of people living with severe obesity. This is the first report from the national Scottish cohort study of people seeking surgical treatment for severe obesity. We recruited 444 adults from 14 centres across Scotland over a three year period, including all NHS centres and major private hospitals undertaking bariatric surgery. We found that higher BMI and older age were associated with decreased physical quality of life, increased use of specialist aids and equipment in the home, and a high prevalence of comorbidities.

There has been a significant increase in the prevalence of a BMI ≥ 40kg/m^2 in recent decades; in Scotland, obesity prevalence has trebled in women age 16-64 since 1995. However, it is hard to assess the global increase due to lack of reporting of BMI ≥ 40kg/m^2 in national health survey data(31). While it is known that health care resource use increases in people with a BMI ≥ 30kg/m^2 , with service use estimated to be over 25% higher than for those with a BMI in the normal weight range(32), few data exist for those with BMI 40kg/m^2 and above. In 2016, the Global BMI Mortality Collaboration(33) conducted an individual-participant-data meta-analysis of 239 prospective studies and found a 2.76 times increased risk of all cause mortality for people with a BMI of 40-60kg/m^2. Greive *et al.*(34) conducted a systematic review which focussed on the economic cost of severe obesity (BMI ≥ 40kg/m^2) and found limited literature describing increased prescribing, outpatient utilisation and intensive care admission, and hospital length of stays during critical illness. However, in both studies there was no dissagregation of BMI beyond > 40kg/m^2 meaning that the health consequences of severe obesity are not yet fully described.

There has been extensive research on the relationship between health-related quality of life and obesity(35). Ul-Haq *et al.*(36) performed a meta-analysis of eight studies (43 086 particpants) and found physical quality of life, measured by the Rand 36-item Short Form Health Survey (SF-36), was

For peer review only

For peer review only - http://bmjopen.bmj.com/site/about/guidelines.xhtml

reduced by 9.72 points in those with BMI 40kg/m^2 compared to those with a BMI in the normal range, although, again, there was no disaggregation above BMI 40kg/m^2. Van Nunen *et al.*(37) perfomed a meta-analysis to compare the general, non-treatment seeking population to patients within weight management programmes and those seeking bariatric surgery. They found that those seeking surgical treatment reported the most severely reduced HRQoL, perhaps reflecting their reasons for seeking definitive surgical treatment. Our cohort of treatment-seeking individuals who completed a rich battery of patient reported measures, provides data to show that health-related and obesity-related quality of life of those with the highest body mass is extremely poor and this is compounded by increasing age. Quality of life scores of SCOTS participants in both the upper BMI (≥ 55 kg/m^2) and older age (≥ 55 years) groups included physical scores comparable with those reported by cancer patients receiving palliative care(38), patients with chronic heart failure expressing end of life preferences(39), and patients with end stage kidney disease(40). Furthermore, patients with severe chronic obstructive pulmonary disease (COPD) report higher quality of life scores, indicating a better quality of life, than our cohort of treatment-seeking obese participants(41). As far as we are aware, this is the first study to investigate physical and mental health in patients with severe obesity awaiting bariatric surgery with finer level consideration of BMI up to ≥ 55kg/m^2.

UK guidelines(42) currently indicate that bariatric surgery is a treatment option for those with a BMI ≥ 40 kg/m^2, or between 35 kg/m^2 and 40 kg/m^2 in the presence of other significant diseases (T2D or hypertension, for example) which could be improved if they lost weight. Non-surgical weight management must have been attempted but not resulted in clinically beneficial weight loss before surgery is indicated. However, baseline SCOTS data appear to suggest that the low prioritisation of bariatric surgery and a lengthy preoperative pathway in the UK is associated with surgical treatment being reserved for individuals at an older age with very high BMI. Indeed, in 2018, the Global Registry initiative of the International Federation for the Surgery of Obesity and Metabolic Disorders (IFSO) reported a global median pre-bariatric surgery BMI of 41.7 kg/m^2(43), as compared to 47.6

For peer review only - http://bmjopen.bmj.com/site/about/guidelines.xhtml

kg/m^2 in the SCOTS cohort. Similarly, IFSO reported a median patient age of 42 years at the time of bariatric surgery(43), as compared to a median age of 47 years for SCOTS participants. This combination of higher BMI and older age means that, at the time of surgery, Scottish patients have high levels of comorbidity and poor physical functioning. Bariatric surgery is considered a highly cost-effective intervention(44). However, the health economic models rely on data primarily from US and Scandinavian studies(45–49), where BMI and age at the time of surgery are lower than in the UK. Higher BMI and older age are risk factors for post-operative complications(50,51) and also associated with lower total weight loss(52–54). Type 2 diabetes remission rates are negatively correlated with age(55). As such, focussing bariatric surgery provision on those with older age and higher BMI may result in higher costs of surgery with increased length of hospital stay, higher rates of post-operative complications, lower overall weight loss and lower rates of disease remission. Consequently, the impressive health benefits and resultant cost-savings of bariatric surgery observed in clinical trials and observational cohorts from other countries may not be fully realised for the UK/Scottish population.

The SCOTS dataset represents a unique and rich resource. A major strength of the study is its representativeness. Indeed, every clinical team providing publicly-funded bariatric surgery in Scotland approached their patients for recruitment to the study, rendering it highly representative of the population in comparison to other studies undertaken in the field. However, the number of participants with valid baseline questionnaires was lower than anticipated. In many cases this could be attributed to the participant undergoing surgery before completing the questionnaire, or the participant leaving the bariatric surgery pathway before surgery. Participants living in the most deprived areas were well represented in our cohort and the mean quality of life findings were broadly similar to those of bariatric surgery cohorts from across the world(56–58). A further strength of the study is that questionnaires were externally validated and wide-ranging, containing a number of unique questions covering medical, social, psychological and physical functioning domains. This wide range of self-reported health measures will allow us to account for a range of potentially

For peer review only - http://bmjopen.bmj.com/site/about/guidelines.xhtml

mediating and confounding factors in future analyses. In addition, we have revealed the extent of comorbidities, including musculoskeletal, urinary and mental health problems affecting people with severe obesity.

A limitation of this study is that selection for bariatric surgery is often based on the presence of comorbidity so these results, while applicable to a treatment-seeking population, may not be directly applicable to the whole population with severe obesity in the wider society. While we will have access to medical records via electronic health record data linkage in follow-up, the current analyses are based on self-report of selected comorbidities. It is well known that self-reported weights are underreported, particularly by people with very high BMI(59). However, we are confident of the accuracy of weight and height as these data were collected in clinic during the recruitment visit. With regard to future analyses, follow-up of the SCOTS cohort is ongoing which will allow us to report the longitudinal health trajectory after bariatric surgery. The electronic health record infrastructure in Scotland(60) will also permit us to study health outcomes of SCOTS participants across a range of clinical events and, ultimately, determine the effect of bariatric surgery and other factors associated with health outcomes.

Obesity is a multisystem disease which affects every facet of a person's life. Our data have shown that higher body mass index combined with older age is associated with very poor physical functioning, and health-related and obesity-related quality of life. Indeed, quality of life scores for those living with severe obesity in Scotland are akin to those seen in the end stage of diseases such as cancer and heart failure. The health consequences of severe obesity and the extent to which treatments such as bariatric surgery can improve these are not yet known. Researchers should ensure that they include people with severe obesity in population cohorts and treatment studies, and study the impact of severe obesity in more detail; there are substantial differences in the health

For peer review only

For peer review only - http://bmjopen.bmj.com/site/about/guidelines.xhtml

status of those with a BMI above 50kg/m^2 and those whose BMI is around 40kg/m^2. Policy makers should consider the health and care needs of the growing numbers of inidviduals living with obesity. There will be considerable future demand for health care and services must be designed to accomodate the physical needs of the individuals. Prevention of obesity is clearly paramount to avoid more people developing such a debilitating, chronic condition. Investment is urgently needed, both in the UK and globally, to provide increased access to bariatric surgery and other forms of effective weight management, directly targeting patient groups who will benefit from surgical intervention as early in the disease course as possible.

FUNDING

This work was supported by the National Institute for Health Research (NIHR) Health Technology Assessment Programme (project number 10/42/02). The views expressed are those of the authors and not necessarily those of the NIHR or the Department of Health and Social Care.

COMPETING INTERESTS

Professor Lean has received advisory board and speaker fees from Eli Lilly, Novo Nordisk, Merck and Sanofi. Dr Lindsay has received advisory board and speaker fees from Eli Lilly, Novo Nordisk and Servier. Professor Sattar has consulted for Eli Lilly, Novo Nordisk, Pfizer and Sanofi and received compensation. All other authors declare no potential competing interests.

AUTHOR CONTRIBUTION

All authors were involved in the conception and design of the study, revising it critically and approving the final version for submission. NG conducted the analyses. RMM and JL wrote the first

For peer review only

For peer review only - http://bmjopen.bmj.com/site/about/guidelines.xhtml

draft. All authors agree to be accountable for all aspects of the work and to ensure that questions relating to the accuracy or integrity of any part of the work are appropriately investigated and resolved.

ACKNOWLEDGEMENTS

These data have been made available for analysis by members of the SCOTS Investigators group, Mr Afshin Alijani, Dr Ewan Bell, Professor Andrew Collier, Ms Carol Craig, Dr Jennifer Darrion, Mr David Galloway, Mr Hasan Kasem, Ms Joeleen McKean, Mr Stuart Oglesby, Mr Chris Shearer, Mr Rob Stuart and Mr Bruce Tulloh, due to their hard work in collecting data, and the patients who have participated in the study. The authors also wish to thank Mr Richard Welbourn, Ms Catherine Quinn, Professor David Beard, Dr John Mooney, Ms Elizabeth Thompson, Mrs Joanne O'Donnell, Mrs Jane Munro, Dr Iona Donnelly and Dr Samantha Alvarez Madrazo.

REFERENCES

1 Kitahara CM, Flint AJ, Berrington de Gonzalez A, Bernstein L, et al. Association between Class III Obesity (BMI of 40-59 kg/m2) and Mortality: A Pooled Analysis of 20 Prospective Studies. *PLoS Med* 2014;11(7):e1001673

2 Sturm R, Hattori A. Morbid obesity rates continue to rise rapidly in the United States. *Int J Obes* 2013;37(6):889-891

3 Flegal KM, Kruszon-Moran D, Carroll MD, et al. Trends in obesity among adults in the United States, 2005 to 2014. *JAMA* 2016;315(21):2284-2291

4 Baker C. House of Commons Library | Obesity Statistics. *Obes Stat (Briefing Pap Number 3336)* 2019;3336:1-18

For peer review only

5 Grieve E, Mackenzie RM, Munro J, et al. Variations in bariatric surgical care pathways: A national costing study on the variability of services and impact on costs. *BMC Obes* 2018;5(1):43

6 Colquitt JL, Pickett K, Loveman E, et al. Surgery for weight loss in adults. *Cochrane Database Syst Rev* 2014;issue 8:CD003641

7 O'Brien PE, McPhail T, Chaston TB, et al. Systematic review of medium-term weight loss after bariatric operations. *Obes Surg* 2006;16:1032-1040

8 Angrisani L, Santonicola A, Iovino P, et al. Bariatric Surgery Worldwide 2013. *Obes Surg* 2015;25(10):1822-1832

9 Welbourn R, Le Roux CW, Owen-Smith A, et al. Why the NHS should do more bariatric surgery; how much should we do? *BMJ (Online)* 2016;353:i1472

10 Hedenbro JL, Näslund E, Boman L, et al. Formation of the Scandinavian Obesity Surgery Registry, SOReg. *Obes Surg* 2015;25(10):1893-1900

11 Booth HP, Khan O, Fildes A, et al. Changing Epidemiology of Bariatric Surgery in the UK: Cohort Study Using Primary Care Electronic Health Records. *Obes Surg* 2016;26(8):1900-1905

12 Logue J, Stewart S, Munro J, et al. SurgiCal obesity treatment study (SCOTS): Protocol for a national prospective cohort study of patients undergoing bariatric surgery in Scotland. *BMJ Open* 2015;5(5):e008106

13 von Elm E, Altman DG, Egger M, et al. The Strengthening the Reporting of Observational Studies in Epidemiology (STROBE) statement: guidelines for reporting observational studies. *Lancet* 2007;370(9596):1453-1457

14 Macran S, Wileman S, Barton G, et al. The development of a new measure of quality of life in the management of gastro-oesophageal reflux disease: The Reflux questionnaire. *Qual Life*

For peer review only

Res 2007;16(2):331-343

15 Barry MJ, Fowler FJ, O'leary MP, et al. The American Urological Association Symptom Index for Benign Prostatic Hyperplasia. *J Urol* 2017;197(2):S189-S197

16 Avery K, Donovan J, Peters TJ, et al. ICIQ: A brief and robust measure for evaluating the symptoms and impact of urinary incontinence. *Neurourol Urodyn* 2004;23(4):322–330

17 Gosman GG, King WC, Schrope B, et al. Reproductive health of women electing bariatric surgery. *Fertil Steril* 2010;94(4):1426-1431

18 Feldman HA, Goldstein I, Hatzichristou DG, et al. Impotence and its medical and psychosocial correlates: Results of the Massachusetts Male Aging Study. *J Urol* 1994;151(1):54-61

19 Spitzer RL, Kroenke K, Williams JBW, et al. A brief measure for assessing generalized anxiety disorder: The GAD-7. *Arch Intern Med* 2006;166(10):1092-1097

20 Spitzer RL, Kroenke K, Williams JBW. Validation and utility of a self-report version of PRIME-MD: The PHQ Primary Care Study. *J Am Med Assoc* 1999;282(18):1737-1744

21 Reinert DF, Allen JP. The alcohol use disorders identification test (AUDIT): A review of recent research. *Alcohol Clin Exp Res* 2002;26(2):272–279

22 Ware JE, Kosinski M, Keller SD. A 12-Item Short-Form Health Survey: Construction of Scales and Preliminary Tests of Reliability and Validity. *Med Care* 1996;34(3):220-233

23 Herdman M, Gudex C, Lloyd A, et al. Development and preliminary testing of the new five-level version of EQ-5D (EQ-5D-5L). *Qual Life Res* 2011;20(10):1727-1736

24 Fermont JM, Blazeby JM, Rogers CA, et al. The EQ-5D-5L is a valid approach to measure health related quality of life in patients undergoing bariatric surgery. *PLoS One* 2017;12(12):e0189190

25 Kolotkin RL, Crosby RD. Psychometric evaluation of the impact of weight on quality of life-lite

For peer review only

For peer review only - http://bmjopen.bmj.com/site/about/guidelines.xhtml

questionnaire (IWQOL-Lite) in a community sample. *Qual Life Res* 2002;11(2):157-171

26 Kolotkin RL, Crosby RD. The Impact of Weight on Quality of Life-Lite (IWQOL-Lite): User's Manual. Durham, NC: Obesity and Quality of Life Consulting 2002

27 Scheier MF, Carver CS, Bridges MW. Distinguishing Optimism From Neuroticism (and Trait Anxiety, Self-Mastery, and Self-Esteem): A Reevaluation of the Life Orientation Test. *J Pers Soc Psychol* 1994;67(6):1063-1078

28 Craig CL, Marshall AL, Sjöström M, et al. International physical activity questionnaire: 12-Country reliability and validity. *Med Sci Sports Exerc* 2003;35(8):1381-1395

29 Scottish Index of Multiple Deprivation 2016: introductory booklet. 2016. https://www.gov.scot/publications/scottish-index-multiple-deprivation-2016/ (accessed January 2020)

30 Arterburn DE, Courcoulas AP. Bariatric surgery for obesity and metabolic conditions in adults. *BMJ (Online)* 2014;349:g3961

31 Williamson K, Nimegeer A, Lean M. Rising prevalence of BMI ≥40 kg/m^2: A high-demand epidemic needing better documentation. *Obes Rev* 2020;21(4):e12986

32 le Roux CW, Chubb B, Nørtoft E, et al. Obesity and healthcare resource utilization: results from Clinical Practice Research Database (CPRD). *Obes Sci Pract* 2018;4(5):409-416

33 Di Angelantonio E, Bhupathiraju SN, Wormser D, et al. Body-mass index and all-cause mortality: individual-participant-data meta-analysis of 239 prospective studies in four continents. *Lancet* 2016;388(10046):776-786

34 Grieve E, Fenwick E, Yang HC, et al. The disproportionate economic burden associated with severe and complicated obesity: A systematic review. *Obes Rev* 2013;14(11):883-894

35 Kolotkin RL, Andersen JR. A systematic review of reviews: exploring the relationship between

For peer review only

obesity, weight loss and health-related quality of life. *Clin Obes* 2017;7(5):273-289

36 Ul-Haq Z, Mackay DF, Fenwick E, et al. Meta-analysis of the association between body mass index and health-related quality of life among adults, assessed by the SF-36. *Obesity* 2013;21(3):E322-E327

37 Van Nunen AMA, Wouters EJM, Vingerhoets AJJM, et al. The health-related quality of life of obese persons seeking or not seeking surgical or non-surgical treatment: A meta-analysis. *Obes Surg* 2007;17(10):1357-1366

38 Radbruch L, Sabatowski R, Loick G, et al. Cognitive impairment and its influence on pain and symptom assessment in a palliative care unit: Development of a Minimal Documentation System. *Palliat Med* 2000;14(4):266-276

39 Brunner-La Rocca HP, Rickenbacher P, Muzzarelli S, et al. End-of-life preferences of elderly patients with chronic heart failure. *Eur Heart J* 2012;33(6):752-759

40 Iyasere O, Brown EA, Johansson L, et al. Quality of life with conservative care compared with assisted peritoneal dialysis and haemodialysis. *Clin Kidney J* 2018;12(2):262-268

41 Voll-Aanerud M, Eagan TML, Wentzel-Larsen T, et al. Respiratory symptoms, COPD severity, and health related quality of life in a general population sample. *Respir Med* 2008;102(3):399-406

42 National Institute for Health and Clinical Excellence (NICE). *Weight management: lifestyle services for overweight or obese adults.* 2014. https://www.nice.org.uk/guidance/ph53 (accessed January 2020)

43 Welbourn R, Hollyman M, Kinsman R, et al. Bariatric Surgery Worldwide: Baseline Demographic Description and One-Year Outcomes from the Fourth IFSO Global Registry Report 2018. *Obes Surg* 2019;29(3):782-795

For peer review only

For peer review only - http://bmjopen.bmj.com/site/about/guidelines.xhtml

44 Avenell A, Robertson C, Skea Z, et al. Bariatric surgery, lifestyle interventions and orlistat for severe obesity: The rebalance mixed-methods systematic review and economic evaluation. *Health Technol Assess* 2018;22(68):1-246

45 Alsumali A, Eguale T, Bairdain S, et al. Cost-Effectiveness Analysis of Bariatric Surgery for Morbid Obesity. *Obes Surg* 2018;28(8):2203-2214

46 Maciejewski ML, Arterburn DE. Cost-effectiveness of Bariatric Surgery. *JAMA* 2013;310(7):742-743

47 Keating C, Neovius M, Sjöholm K, et al. Health-care costs over 15 years after bariatric surgery for patients with different baseline glucose status: Results from the Swedish Obese Subjects study. *Lancet Diabetes Endocrinol* 2015;3(11):855-865

48 Hoerger TJ, Zhang P, Segel JE, et al. Cost-effectiveness of bariatric surgery for severely obese adults with diabetes. *Diabetes Care* 2010;33(9):1933-1939

49 Borisenko O, Mann O, Duprée A. Cost-utility analysis of bariatric surgery compared with conventional medical management in Germany: A decision analytic modeling. *BMC Surg* 2017;17(1):87

50 Khan MA, Grinberg R, Johnson S, et al. Perioperative risk factors for 30-day mortality after bariatric surgery: Is functional status important? *Surg Endosc* 2013;27(5):1772-1777

51 Quilliot D, Sirveaux MA, Nomine-Criqui C, et al. Evaluation of risk factors for complications after bariatric surgery. *J Visc Surg* 2018;155(3):201-210

52 Livhits M, Mercado C, Yermilov I, et al. Preoperative predictors of weight loss following bariatric surgery: Systematic review. *Obes Surg* 2012;22:70-89

53 Nickel F, De La Garza JR, Werthmann FS, et al. Predictors of Risk and Success of Obesity Surgery. *Obes Facts* 2019;12(4):427-439

For peer review only

54 Parri A, Benaiges D, Schröder H, et al. Preoperative predictors of weight loss at 4 years following bariatric surgery. *Nutr Clin Pract* 2015;30(3):420-424

55 Jans A, Näslund I, Ottosson J, et al. Duration of type 2 diabetes and remission rates after bariatric surgery in Sweden 2007-2015: A registry-based cohort study. *PLoS Med* 2019;16(11):e1002985

56 Malone M, Alger-Mayer S, Polimeni JM. Health Related Quality of Life After Gastric Bypass Surgery. *Appl Res Qual Life* 2012;7:155-161

57 Pristed SG, Omar HK, Kroustrup JP. Association Between Fulfilment of Expectations and Health-related Quality of Life after Gastric Bypass. *Appl Res Qual Life* 2013;8(1):101-111

58 Julia C, Ciangura C, Capuron L, et al. Quality of life after Roux-en-Y gastric bypass and changes in body mass index and obesity-related comorbidities. *Diabetes Metab* 2013;39(2):148-154

59 Spencer EA, Appleby PN, Davey GK, et al. Validity of self-reported height and weight in 4808 EPIC–Oxford participants. *Public Health Nutr* 2002;5(4):561-565

60 Public Health Scotland Data and Intelligence. https://www.isdscotland.org (accessed January 2020)

For peer review only

FIGURE LEGEND

Figure 1. Screening, consent and follow-up. 548 patients were screened for inclusion in the SurgiCal Obesity Treatment Study. Of these 548, 103 were non-eligible (62 patients had undergone previous bariatric surgery and 1 patient lacked the necessary English language skills) and 40 were eligible but did not consent. 31 patients consented to only part 1 of the study (data linkage) while 413 consented to both part 1 and part 2 (completion of baseline PROMs). 249 baseline PROMs were available for analysis. PROMs, patient reported outcome measures.

For peer review only - http://bmjopen.bmj.com/site/about/guidelines.xhtml

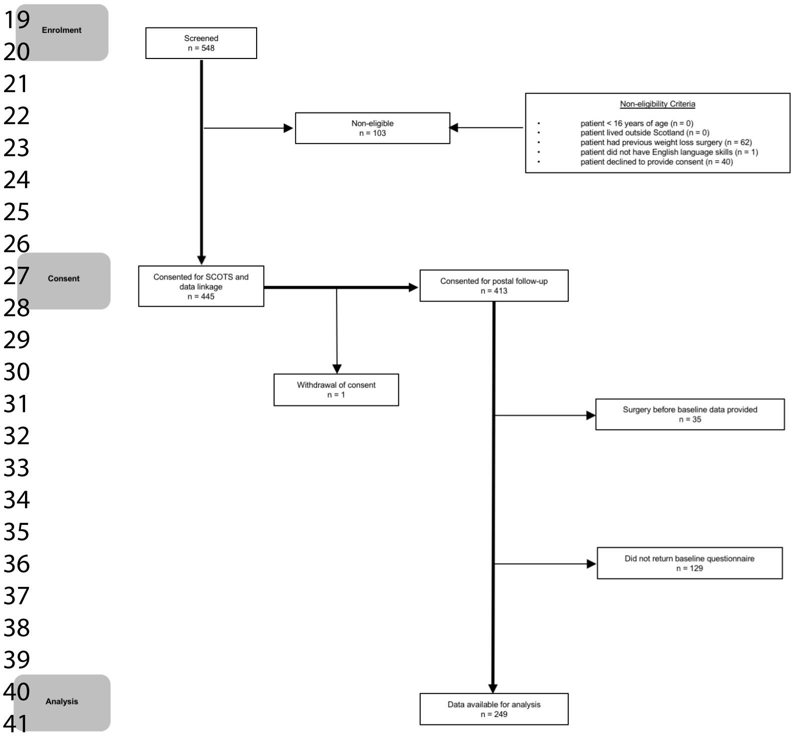
Enrolment
Screened
n = 548
Non-eligible
n = 103
Non-eligibility Criteria
patient < 16 years of age (n = 0)
patient lived outside Scotland (n = 0)
patient had previous weight loss surgery (n = 62)
patient did not have English language skills (n = 1)
patient declined to provide consent (n = 40)
Consent
Consented for SCOTS and data linkage
n = 445
Consented for postal follow-up
n = 413
Withdrawal of consent
n = 1
Surgery before baseline data provided
n = 35
Did not return baseline questionnaire
n = 129
Analysis
Data available for analysis
n = 249

Supplementary Table 1. STROBE Statement completed checklist (from von Elm *et al.* [1]).

SECTION/TOPIC	ITEM No.	STROBE Recommendation	OUR PAPER
**Title and abstract**	1	(a) Indicate the study's design with a commonly used term in the title or the abstract	(a) See title
		(b) Provide in the abstract an informative and balanced summary of what was done and what was found	(b) See abstract
**Introduction**			
Background / rationale	2	Explain the scientific background and rationale for the investigation being reported	Paragraphs 1-3 provide background and paragraph 4 explains rationale
Objectives	3	State specific objectives, including any prespecified hypotheses	Paragraph 4
**Methods**			
Study design	4	Present key elements of study design early in the paper	Paragraph 3/'Study Design' section
Setting	5	Describe the setting, locations, and relevant dates, including periods of recruitment, exposure, follow-up, and data collection	Paragraphs 4-6/'Participant and Centre Eligibility', 'Recruitment Procedures and Consent' and 'Data Collection' sections
Participants	6	(a) Give the eligibility criteria, and the sources and methods of selection of participants. Describe methods of follow-up	(a) Paragraphs 4-6/'Participant and Centre Eligibility', 'Recruitment Procedures and Consent' and 'Data Collection' sections
		(b) For matched studies, give matching criteria and number of exposed and unexposed	(b) Not applicable
Variables	7	Clearly define all outcomes, exposures, predictors, potential confounders, and effect modifiers. Give diagnostic criteria, if applicable	Paragraphs 7-9/'Data Collection' section
Data sources / measurement	8	For each variable of interest, give sources of data and details of methods of assessment (measurement). Describe comparability of assessment methods if there is more than one group	Paragraphs 7 and 8/'Data Collection' section
Bias	9	Describe any efforts to address potential sources of bias	Not applicable
Study size	10	Explain how the study size was arrived at	Paragraph 4/'Patient and Centre Eligibility' section, paragraph 1 of 'Results' section

For peer review only

Quantitative variables	11	Explain how quantitative variables were handled in the analyses. If applicable, describe which groupings were chosen and why	'Statistical Analyses' section
Statistical methods	12	(a) Describe all statistical methods, including those used to control for confounding	(a) 'Statistical Analyses' section
		(b) Describe any methods used to examine subgroups and interactions	(b) Not applicable
		(c) Explain how missing data were addressed	(c) 'Statistical Analyses' section
		(d) If applicable, explain how loss to follow-up was addressed	(d) Not applicable
		(e) Describe any sensitivity analyses	(e) Not applicable
**Results**			
Participants	13	(a) Report numbers of individuals at each stage of study— e.g. numbers potentially eligible, examined for eligibility, confirmed eligible, included in the study, completing follow-up, and analysed	(a) Paragraph 1 and Figure 1
		(b) Give reasons for non-participation at each stage	(b) Paragraph 1 and Figure 1
		(c) Consider use of flow diagram	(c) Figure 1
Descriptive data	14	(a) Give characteristics of study participants (e.g. demographic, clinical, social) and information on exposures and potential confounders	(a) Paragraphs 2-5/'Characteristics of recruited and analysed sample', 'Comorbidities', "Health and obesity-related quality of life' and physical activity' sections, Table 1 and Table 2
		(b) Indicate number of participants with missing data for each variable of interest	(b) Paragraphs 2-7, Table 1, Table 2, Supplementary Table 2 and Supplementary Table 3
		(c) Summarise follow-up time (e.g. average and total amount)	(c) Not applicable
Outcome data	15	Report numbers of outcome events or summary measures over time	Table 2, Supplementary Table 2 and Supplementary Table 3
Main results	16	(a) Give unadjusted estimates and, if applicable, confounder-adjusted estimates and their precision (e.g. 95% confidence interval). Make clear which confounders were adjusted for and why they were included	(a) Table 3 and Supplementary Table 4
		(b) Report category boundaries when continuous variables were categorised	(b) Supplementary Table 4

For peer review only

		(c) If relevant, consider translating estimates of relative risk into absolute risk for a meaningful time period	(c) Not applicable
Other analyses	17	Report other analyses done e.g. analyses of subgroups and interactions, and	Not applicable
**Discussion**			
Key results	18	Summarise key results with reference to study objectives	Paragraph 1
Limitations	19	Discuss limitations of the study, taking into account sources of potential bias or imprecision. Discuss both direction and magnitude of any potential bias	Paragraph 5
Interpretation	20	Give a cautious overall interpretation of results considering objectives, limitations, multiplicity of analyses, results from similar studies, and other relevant evidence	'Conclusion'
Generalisability	21	Discuss the generalisability (external validity) of the study results	Paragraph 5
**Other**			
Funding	22	Give the source of funding and the role of the funders for the present study and, if applicable, for the original study on which the present article is based	'Funding' section

Reference

1) von EE, Altman DG, Egger M, Pocock SJ, Gotzsche PC, Vandenbroucke JP. The Strengthening the Reporting of Observational Studies in Epidemiology (STROBE) statement: guidelines for reporting observational studies. *Lancet* 2007; **370**(9596): 1453-1457

For peer review only

Glasgow Clinical Trials Unit

Surgical Obesity Treatment Study

SCOTS

Running title: **Surgical Obesity Treatment Study**

Protocol Version: **1.6**

Date: 22/11/2017

REC Reference Number: **13/WS/0005**

ISRCTN/Clinical trial.gov: **47072588**

Sponsor's Protocol Number: **GN21CA196**

Sponsor: **NHS Greater Glasgow & Clyde**

Funder: **National Institute for Health Research Health Technology Assessment Programme Ref 10/42/02**

Date	Protocol version	Amendment number & description
14/12/2012	1.0	Original
15/04/2013	1.1	Amendment 1: Minor changes to the format and presentation of the protocol
11/07/2013	1.2	Amendment 2: Correction of typographical errors in the protocol
30/06/2014	1.3	Amendment 3: Change to recruitment process for private hospitals involved in the study. Private hospitals will now become participant identification sites (and any NHS sites which in future are unable to support local recruitment), with participants being recruited and

For peer review only - http://bmjopen.bmj.com/site/about/guidelines.xhtml

		consented by a research team member (usually a research nurse).
03/06/2015	1.4	Amendment 4: Introduction of follow-up letter for patients recruited over the telephone by research nurses at the clinical research facility if completed consent form is not received within two weeks.
07/02/2017	1.5	Amendment 5; Revised study plan (including timetable & outputs). Patient recruitment stopping at a lower number and earlier than originally planned due to unforeseen recruitment issues. Amendment to clinical objectives and outcomes to reflect smaller sample size and shorter follow-up period. Reduction of post-operative questionnaire frequency to 2 and 3 years post-operatively only. Introduction of modest voucher incentives for PROMS completion.
22/11/2017	1.6	Amendment 6; Removal of annual blood test reminder. This procedure is completed as part of the routine clinical care by the patient's GP and therefore, it is not necessary for a reminder to be sent in addition for the SCOTS study.

This study will be performed according to the Research Governance Framework for Health and Community Care (Second edition, 2006) and WORLD MEDICAL ASSOCIATION DECLARATION OF HELSINKI Ethical Principles for Medical Research Involving Human Subjects 1964 (as amended).

For peer review only - http://bmjopen.bmj.com/site/about/guidelines.xhtml

Glasgow Clinical Trials Unit

CONTACTS

Chief Investigator

Dr Jennifer Logue MBChB MRCP MD FRCPath
CSO Clinician Scientist
Clinical Senior Lecturer and Honorary Consultant in Metabolic Medicine
Institute of Cardiovascular and Medical Sciences University of Glasgow
BHF Glasgow Cardiovascular Research Centre University of Glasgow
126 University Place, Room 208
University of Glasgow
Glasgow
G12 8TA

Tel: 0141 330 2569
Fax: 0141 330 6955
E-mail: Jennifer.Logue@Glasgow.ac.uk

Co-investigator

Professor Naveed Sattar
Professor of Metabolic Medicine
Cardiovascular and Medical Sciences
BHF Glasgow Cardiovascular Research Centre
RC214 Level C2
126 University Place, Room 208
University of Glasgow
Glasgow
G12 8TA
Tel: 0141 330 3419
Fax: 0141 330 6972
E-mail: Naveed.Sattar@glasgow.ac.uk

Dr Julie Bruce
Principal Research Fellow
Division of Health Sciences,
Warwick Medical School,
The University of Warwick,
Coventry,
CV4 7AL
Tel: 024 7615 1128
E-mail: julie.bruce@warwick.ac.uk

Mr Duff Bruce
Bariatric Surgeon
Ward 33
Aberdeen Royal Infirmary
Foresterhill
Aberdeen
AB25 2ZN
Tel: 01224 551275
Fax: 01224 443212
E-mail: duff.bruce@nhs.net

For peer review only

For peer review only - http://bmjopen.bmj.com/site/about/guidelines.xhtml

Glasgow Clinical Trials Unit

Dr Robert Lindsay
Reader Diabetes and Endocrinology
Cardiovascular and Medical Sciences
RC337 Level C3
BHF Glasgow Cardiovascular Research Centre
126 University Place, Room 208
University of Glasgow
Glasgow
G12 8TA
Tel: 0141 211 2502
Fax: 0141 330 6997
E-mail: Robert.Lindsay@glasgow.ac.uk

Professor Ian Ford
Professor of Biostatistics
R1122B Level 11
Robertson Centre for Biostatistics
Boyd Orr Building
Glasgow
G12 8QQ
Tel: 0141 330 4048
Fax: 0141 330 5094
E-mail: Ian.Ford@glasgow.ac.uk

Professor Andrew Briggs
Chair in Health Economics
(Health Economics and Health Technology Assessment) HEHTA
Institute of Health & Wellbeing
1 Lilybank Gardens
Glasgow
G12 8RZ
Tel: 0141 330 5017
E-mail: Andrew.Briggs@glasgow.ac.uk

Professor Mike Lean
Professor in Human Nutrition
R408 Level 4
Human Nutrition
Glasgow Royal Infirmary
84 Castle Street Glasgow
G40SF
Tel: 0141 211 4686
Fax: 0141 211 4844
E-mail: Mike.Lean@glasgow.ac.uk

Jane Munro
Research Lead
Cardiovascular and Medical Sciences
BHF Glasgow Cardiovascular Research Centre University of Glasgow
126 University Place, Room 208
University of Glasgow
Glasgow

For peer review only - http://bmjopen.bmj.com/site/about/guidelines.xhtml

Glasgow Clinical Trials Unit

G12 8TA
Tel: 0141 330 1670
E-mail: Jane.Munro@glasgow.ac.uk

Project Research Assistant

Mrs. Joanne O'Donnell
SCOTS Research Assistant
Cardiovascular and Medical Sciences
BHF Glasgow Cardiovascular Research Centre University of Glasgow
126 University Place, Room 208
University of Glasgow
Glasgow
G12 8TA
Tel: 0141 330 1670
Fax: 0141 330 5094
E-mail: Joanne.O'Donnell@glasgow.ac.uk

Study Statistician

Professor Ian Ford
Professor of Biostatistics
R1122B Level 11
Robertson Centre for Biostatistics
Boyd Orr Building
Glasgow
G12 8QQ
Tel: 0141 330 4048
Fax: 0141 330 5094
E-mail: Ian.Ford@glasgow.ac.uk

Data Centre

Alex McConnachie
Assistant Director of Biostatistics (Robertson Centre)
R1122E Level 11
Robertson Centre for Biostatistics
Boyd Orr Building
Glasgow
G12 8QQ
Tel: 01413304744
Fax: 0141 330 5094
E-mail: Alex.McConnachie@glasgow.ac.uk

Chair of the Study Steering Committee

Mr Richard Welbourn
Bariatric Surgeon
Musgrove Park Hospital
Taunton
TA1 5DA
Tel: 01823 343 565
Fax: 01823 353560
E-mail: richardwelbourn@gmail.com

For peer review only

For peer review only - http://bmjopen.bmj.com/site/about/guidelines.xhtml

Glasgow Clinical Trials Unit

Sponsor - NHS Greater Glasgow and Clyde R&D

Sponsor's representative

Dr Maureen Travers
Research Co-ordinator
NHS Greater Glasgow & Clyde
Research and Development Management Office
Tennent Institute
38 Church Street
Western Infirmary
Glasgow G11 6NT
Tel: 0141 211 6389
E-mail: maureen.travers@ggc.scot.nhs.uk

Funding Body

National Institute for Health Research (NIHR)

Funding Body Representative

Mrs Lesley Dodd
Programme Manager
NIHR Co-ordinating Centre for Health Technology Assessment (NETSCC, HTA)
University of Southampton
Alpha House
Enterprise Road
Southampton Science Park
Chilworth
Southampton
SO16 7NS
Tel: 02380 597558
Fax: 02380 595639
E-mail: L.Dodd@southampton.ac.uk

For peer review only - http://bmjopen.bmj.com/site/about/guidelines.xhtml

Glasgow Clinical Trials Unit

PROTOCOL APPROVAL

Surgical Obesity Treatment Study (SCOTS)

Chief Investigator

Dr Jennifer Logue
MBChB MRCP MD FRCPath CSO Clinician Scientist
Clinical Senior Lecturer and Honorary Consultant in Metabolic Medicine
Institute of Cardiovascular and Medical Sciences BHF Glasgow Cardiovascular Research Centre
University of Glasgow
126 University Place, Room 208
University of Glasgow
Glasgow
G12 8QQ

Signature:

Date: Thursday, 25 April 2019~~Wednesday, 29 November 2017~~

Sponsor's representative

Dr Maureen Travers
Research Co-ordinator
NHS Greater Glasgow & Clyde Research and Development Management Office
Tennent Institute
38 Church Street
Western Infirmary
Glasgow
G11 6NT

Signature:

Date:

For peer review only - http://bmjopen.bmj.com/site/about/guidelines.xhtml
For peer review only

TABLE OF CONTENTS

CONTACTS .... **3~~2~~**

PROTOCOL APPROVAL .... **7~~6~~**

TABLE OF CONTENTS .... **8~~7~~**

ABBREVIATIONS .... **11~~10~~**

STUDY SYNOPSIS .... **14~~13~~**

GLOSSARY OF TERMS .... **16~~15~~**

STUDY FLOW CHART .... **19~~18~~**

SCHEDULE OF ASSESSMENTS .... **20~~19~~**

1 INTRODUCTION .... **23~~22~~**

1.1 Background .... 23~~22~~

1.2 Rationale .... 23~~22~~

1.2.1 Definition of bariatric surgery .... 24~~22~~

1.2.2 Clinical Outcomes of weight loss surgery .... 24~~22~~

1.3 Study hypothesis .... 25~~24~~

2 STUDY OBJECTIVES .... **25~~24~~**

2.1 Primary Objective .... 25~~24~~

2.2 Secondary Objectives .... 25~~24~~

2.3 Primary Outcome .... 26~~25~~

2.4 Secondary Outcomes .... 26~~25~~

3 STUDY DESIGN .... **27~~26~~**

3.1 Overview of study design .... 27~~26~~

3.1.1 Part 1 Record Linkage .... 29~~28~~

3.1.2 Part 2 Patient Reported Outcomes (PROMS) Questionnaire .... 29~~28~~

3.1.3 Part 3: Registration for a database of bariatric patients for future research .... 29~~28~~

3.1.4 Service user involvement .... 30~~29~~

3.2 Study Population .... 30~~29~~

For peer review only

For peer review only - http://bmjopen.bmj.com/site/about/guidelines.xhtml

3.3 Reference populations 3029

3.4 Inclusion criteria 3130

3.5 Exclusion criteria........ 3130

3.6 Identification of participants and consent 3130

3.7 Withdrawal of subjects........ 3231

4 STUDY PROCEDURES........ 3332

4.1 Duration of participation 36

4.2 Study schedule........ 37

4.2.1 Start of Study - Surgical team registration........ 37

4.2.2 Visit 1 Routine care invitation visit 38

4.2.3 Visit 2 Recruitment visit 38

4.2.4 SCOTS study team contact patient 42

4.2.5 Visit 3 Weight loss operation........ 42

4.2.6 Visit 4 -Post operative follow up........ 4243

4.2.7 Visit 5 - Re-operation (If applicable) 43

5 STATISTICS AND DATA ANALYSIS........ 44

5.1 Statistical Analysis Plan........ 44

5.2 Software for statistical analysis 44

5.3 Sample size 44

5.4 Management and delivery 4645

6 STUDY CLOSURE 4746

7 Data Handling........ 4746

7.1.1 Surgeon registration data 4746

7.1.2 Patient recruitment data 4746

7.1.3 Surgical data collection........ 4847

7.1.4 Patient Reported Outcomes 4847

7.1.5 Routine clinical information 4847

For peer review only

For peer review only - http://bmjopen.bmj.com/site/about/guidelines.xhtml

7.1.6 Re-operation 4847

7.1.7 Record Retention 4947

7.1.8 Access to data 4948

8 SCOTS STUDY MANAGEMENT 5049

8.1 Study Sponsor 5049

8.2 SCOTS Study Team 5049

8.3 SCOTS Principal investigators 5049

8.4 Routine management of study: SCOTS Management Group (SMG) 5049

8.5 SCOTS Steering Committee (SSC) 5150

9 STUDY MONITORING/AUDITING 5251

10 PROTOCOL AMENDMENTS 5251

11 ETHICAL CONSIDERATIONS 5251

11.1 Ethical conduct of the study 5251

11.2 Informed consent 5251

12 INSURANCE AND INDEMNITY 5352

13 FUNDING 5352

14 ANNUAL REPORTS 5352

15 DISSEMINATION OF FINDINGS 5453

16 REFERENCES 5554

17 Appendix E: Summary of Patient Reported Outcome Questions 5958

For peer review only

For peer review only - http://bmjopen.bmj.com/site/about/guidelines.xhtml

ABBREVIATIONS

BHF GCRC	*British Heart Foundation, Glasgow Cardiovascular Research Centre*
BMI	*Body Mass Index*
BP	*Blood Pressure*
BPD-DS	*BilioPancreatic Diversion with Duodenal Switch*
CHD	*Coronary Heart Disease*
CHI	*Community Health Index*
CHF	*Congestive Heart Failure*
CI	*Chief Investigator*
CKD	*Chronic Kidney Disease*
CNORIS	*Clinical Negligence and Other Risks Indemnity Scheme*
CRF	*Case Report Form*
CSO	*Chief Scientist Office*
CTU	*Clinical Trials Unit*
CVD	*Cardiovascular Disease*
DM	*Diabetes Mellitus*
DM(E)C	*Data Monitoring (Ethics) Committee*
DOH	*Department of Health*
DS	*Duodenal Switch*
e-CRF	*Electronic Case Report Form*
EQ-5D	*EuroQol -5D*
GAD-7	*Generalised Anxiety Disorder – 7*
GCP	*Good Clinical Practice*
GCP	*Good Clinical Practice*
GCTU	*Glasgow Clinical Trials Unit*
GP	*General Practitioner*

For peer review only

For peer review only - http://bmjopen.bmj.com/site/about/guidelines.xhtml

GRDS	*Gastric Reduction Duodenal Switch*
GROS	*General Register Office for Scotland*
HbA1c	*Glycated Haemoglobin A1c*
HDL	*High Density Lipoprotein*
HR-QL	*Health Related- Quality of Life*
HTA	*Health Technology Assessment*
ICMS	*Institute of Cardiovascular and Medicinal Sciences*
IPAQ	*International Physical Activity Questionnaire*
IPSS	*International Prostate Symptom Score*
IRAS	*Integrated Research Application System*
ISD	*Information and Services Division (NHS)*
ISRCTN	*International Standard Randomised Controlled Trial Number*
IWQOL-Lite	*Impact of Weight on Quality of Life Lite Questionnaire*
LOT	*Life orientation Test*
MG	*Management Group*
MGM	*Management Group Meeting*
MI	*Myocardial Infarction*
NBSR	*National Bariatric Surgery Register*
NHS	*National Health Service*
NICE	*National Institute for Health and Clinical Excellence*
NIHR	*National Institute for Health Research*
NIHR CSP	*National Institute for Health Research Coordinated System for Gaining NHS Permission*
PHQ-9	*Patient Health Questionnaire - 9*
PI	*Principal Investigator*
PROMS	*Patient Reported Outcome Measures*
QALY	*Quality Adjusted Life Year*

For peer review only

For peer review only - http://bmjopen.bmj.com/site/about/guidelines.xhtml

Glasgow Clinical Trials Unit

QOL	*Quality Of Life*
R&D	*Research and Development*
RCB	*Robertson Centre for Biostatistics*
REC	*Research Ethics Committee*
SCOTS	*SurgiCal Obesity Treatment Study*
SCR	*Scottish Cancer Registry*
SCI-DC	*Scottish Care Information – Diabetes Collaboration*
SDRN	*Scottish Diabetes Research Network*
SDRN EG	*Scottish Diabetes Research Network Epidemiology Group*
SF-12	*Short Form -12*
SIGN	*Scottish Intercollegiate Guidelines Network*
SMG	*SCOTS Management Group*
SMR	*Scottish Morbidity Record*
SOP	*Standard Operating Procedure*
SSC	*SCOTS Steering Committee*
SSI	*Surgical Site Infection*

For peer review only

For peer review only - http://bmjopen.bmj.com/site/about/guidelines.xhtml

STUDY SYNOPSIS

Title of Study:	**Surgical Obesity Treatment Study**; SCOTS
Study Centre:	University of Glasgow
Duration of Study:	03/01/2012 – 30/06/2026
Primary Objective:	The SurgiCal Obesity Treatment Study (SCOTS) will establish the clinical outcomes and adverse events of different bariatric surgical procedures (taking into consideration the different care pathways), their impact on quality of life and nutritional status, and the effect on co-morbidities in a cohort of 450 patients.
Secondary Objectives:	1. The physical and mental health and social burden of severe obesity.<br>2. Incidence of acute and chronic postoperative complications. Acute complications, defined as up to three months post-surgery, will include surgical site infection; chronic complications will include revisional surgery, plastic surgery and chronic pain, for different bariatric surgical procedures<br>3. The effect of surgical experience and the pre and post-operative care pathway on complication rates and weight loss, for different bariatric surgical procedures.<br>4. The effect of the pre-surgical pathway and criteria on bariatric surgery patient selection.<br>5. Change in health related quality of life, anxiety and depression over time pre- and post-operatively for a mean of 3 years from date of bariatric surgery.<br>6. The weight status pre and post-operatively for 3 years after bariatric surgery.<br>7. The glycaemic control, lipids, blood pressure, medication prescription, and rate of diabetes complications (microalbuminuria and renal disease, and retinopathy) in those that have pre-existing diabetes or develop diabetes for 3 years follow up since bariatric surgery.<br>8. Changes in socio-economic factors (employment, benefit receipt, sick leave, and health care use) for 3 years since bariatric surgery.
Primary Endpoint:	Change in weight from pre-bariatric surgery weight

For peer review only - http://bmjopen.bmj.com/site/about/guidelines.xhtml

Glasgow Clinical Trials Unit

Rationale:	The efficacy of bariatric surgery for large scale, long term weight loss is well established. However, many questions remain over the medium term benefits of that weight loss for health, particularly when accounting for the potential complications of surgery.
Methodology:	Observational cohort study/ registry
Sample Size:	450
Screening:	All new bariatric surgery patients
Registration/Randomisation:	Not applicable
Main Inclusion Criteria:	• Patients 16 years or over undergoing their first bariatric surgery in NHS hospitals or private practice in Scotland.<br>• Capacity to consent<br>• Resident in Scotland<br>• Written informed consent
Main Exclusion Criteria:	• Does not meet inclusion criteria<br>• Patients who have had previous weight loss surgery (at time of potential recruitment are undergoing a repeat procedure)<br>• No written informed consent<br>• Patients under 16 years<br>• Patients who do not have capacity to consent for themselves<br>• Weight loss surgery performed at a site outside Scotland<br>• Patients who live outside Scotland<br>• Patients who do not have strong enough English language skills to understand the patient information leaflet without assistance will be excluded from Part 2 of the study.
Product, Dose, Modes of Administration:	Web based or paper questionnaire to be completed<br>1 month pre-surgery<br>2 years post surgery<br>3 years post surgery
Duration of Treatment:	Observational study so protocol will be applied until a participant has been in study for 3 years or until December 2020 whichever comes first. If funding available record linkage will continue throughout patient's life.
Statistical Analysis:	Detailed statistical analysis plans will be developed by the Robertson Centre for Biostatistics to address each of the objectives described above.

For peer review only - http://bmjopen.bmj.com/site/about/guidelines.xhtml

GLOSSARY OF TERMS

Bariatric Surgical Procedures*: A bariatric surgical procedure is defined as a surgical intervention which has the primary purpose of large scale weight loss in a patient who is obese. Bariatric Surgical procedures eligible for inclusion in SCOTS are:* ***duodenal switch, gastric banding, gastric bypass*** *and* ***sleeve gastrectomy****. The inclusion of emerging techniques in this definition will be decided by the steering committee.*

Biliopancreatic diversion with duodenal switch (BPD-DS): *see* **Duodenal Switch***:*

Body mass index (BMI) *is a simple index of weight-for-height that is commonly used to classify* ***overweight*** *and* ***obesity*** *in adults. It is defined as a person's weight in kilograms divided by the square of his height in meters (kg/m^2). BMI provides the most useful population-level measure of overweight and obesity as it is the same for both sexes and for all ages of adults. However, it should be considered a rough guide because it may not correspond to the same degree of fatness in different individuals.*

Co-morbidity*: is either the presence of one or more disorders (or diseases) in addition to a primary disease or disorder, or the effect of such additional disorders or diseases.*

Duodenal Switch*: (DS) is a weight loss surgery procedure that is composed of a restrictive and a malbsorbative aspect. The restrictive portion of the surgery involves removing approximately 70% of the stomach along the greater curvature. The malabsorptive portion of the surgery reroutes a lengthy portion of the small intestine, creating two separate pathways and one common channel. Duodenal switch (DS) is also known as biliopancreatic diversion with duodenal switch (BPD-DS) or gastric reduction duodenal switch (GRDS).*

Endoluminal barrier: *is a liner that fits inside a section of the intestine, creating a barrier between the intestinal wall and the food you eat. It helps patients to lose weight by preventing food from coming into contact with the intestinal wall thus delaying digestion, which will alter the activation of hormonal signals that originate in the intestine.*

Gastric balloon*: An inflatable device implanted in the stomach as an adjunct to therapy of morbid obesity. For the purposes of the SCOTS study a balloon treatment is not considered to be bariatric surgery. Use of a balloon before or after surgery will be recorded as a pre-operative treatment but a balloon alone would not meet the criteria for inclusion in SCOTS.*

Gastric band: *adjustable gastric banding surgery, is a restrictive weight loss surgery that works by decreasing the amount of food that is able to be consumed before feeling full. During gastric banding surgery, a firm silicone band with an inner inflatable ring is placed around the stomach to slow the passage of food from the upper portion of the stomach to the lower portion*

Gastric bypass*: a surgical bypass operation that typically involves reducing the size of the stomach and reconnecting the smaller stomach to bypass the first portion of the small*

For peer review only - http://bmjopen.bmj.com/site/about/guidelines.xhtml

intestine so as to restrict food intake and reduce caloric absorption in cases of severe obesity. This includes Gastric bypass, Roux en-Y (proximal), Gastric bypass, Roux en-Y (distal) and Mini-gastric bypass" (MGB)

Gastric reduction duodenal switch (GRDS): see duodenal switch

Glycaemic control*: is a medical term referring to the typical levels of blood sugar (glucose) in a person with diabetes mellitus. Much evidence suggests that many of the long-term complications of diabetes, especially the microvascular complications, result from many years of hyperglycemia (elevated levels of glucose in the blood).*

Hemoglobin A1c (HbA1c): *Blood sugar levels fluctuate throughout the day and glucose records are imperfect indicators of these changes, the percentage of haemoglobin which is glycosylated is used as a proxy measure of long-term glycemic control in research trials and clinical care of people with diabetes. This test, the hemoglobin A1c or glycosylated hemoglobin reflects average glucoses over the preceding 2–3 months. From 1 October 2011 the way in which HbA1c results are expressed in the UK has changed. Results are now reported in the IFCC reference method of mmol/L, rather than the DCCT units as a percentage. The equivalent of the current HbA1c targets of 6.5% and 7.5% are therefore 48mmol/mol and 58mmol/mol in the new units for diabetic patients, with the non-diabetic reference range of 4.0% to 6.0% being 20mmol/L to 42mmol/L.*

HDL cholesterol: *High density lipoprotein cholesterol. Lipoproteins, which are combinations of fats (lipids) and proteins, are the form in which lipids are transported in the blood. HDLs transport cholesterol from the tissues of the body to the liver, so the cholesterol can be eliminated in the bile. HDL cholesterol is therefore considered the 'good' cholesterol: The higher the HDL cholesterol level, the lower the risk of coronary artery disease. The average man has an HDL cholesterol level of 40 to 50 mg/dL. In the average woman, HDL levels range from 50 to 60 mg/dL.*

Health Technology Assessment*: is a multidisciplinary process that summarises information about the medical, social, economic and ethical issues related to the use of a health technology in a systematic, transparent, unbiased, robust manner. Its aim is to inform the formulation of safe, effective, health policies that are patient focused and seek to achieve best value.*

Hypertriglycerideamia*: denotes high (''hyper-'') blood levels (''-aemia'') of triglycerides, the most abundant fatty molecule in most organisms.*

Laparoscopic gastric banding*: Lap Band surgery, or adjustable gastric banding surgery, is a restrictive weight loss surgery that works by decreasing the amount of food that is able to be consumed before feeling full. During gastric banding surgery, a firm silicone band with an inner inflatable ring is placed around the stomach to slow the passage of food from the upper portion of the stomach to the lower portion. Most surgeries are performed laparoscopically, which allows the surgeon to work using long instruments placed in the body via incisions a few centimetres long.*

For peer review only - http://bmjopen.bmj.com/site/about/guidelines.xhtml

***Laparoscopy**: is a type of surgical procedure that allows a surgeon to access the inside of the abdomen and pelvis without having to make large incisions in the skin. It is also known as keyhole surgery. Laparoscopy is minimally invasive. This is made possible with an instrument called a laparoscope.*

***Morbidity**: Morbidity is an incidence of ill health.*

***Mortality**: is incidence of death in a population.*

***NHS patient**: A patient who receives free healthcare through the National Health Service*

***Nutritional Status**: The state of a person's health in terms of the nutrients stored in his or her body.*

***Obesity**: Obesity is a term used to describe somebody who is very overweight with a high degree of body fat. There are a number of ways a person's weight can be assessed. The most widely used method is the body mass index.*

***Overweight**: a BMI of between 25 and 29, would be considered overweight*

***Obese**: a BMI of 30 or above would be considered obese*

***Private sector**: healthcare and medicine provided by entities other than the government and paid for by the patient or a private insurance company.*

***Prospective cohort study**: a study designed to determine the relationship between a condition and a characteristic shared by some members of a group. The population selected is healthy at the beginning of the study. Members share a particular characteristic. The study follows the population groups over a long period, noting the rate at which a condition occurs in the population.*

***Revisional surgery**: a bariatric surgical procedure on a patient who has already had bariatric surgery*

***Roux-en-Y gastric bypass:** a small part of the stomach is used to create a new stomach pouch, roughly the size of an egg. The smaller stomach is connected directly to the middle portion of the small intestine (jejunum), bypassing the rest of the stomach and the upper portion of the small intestine (duodenum).*

***Sleeve gastrectomy**: is a surgical weight-loss procedure in which the stomach is reduced to about 25% of its original size, by surgical removal of a large portion of the stomach, following the major curve. The open edges are then attached together (often with surgical staples) to form a sleeve or tube with a banana shape. The procedure permanently reduces the size of the stomach. The procedure is performed laparoscopically and is not reversible.*

For peer review only - http://bmjopen.bmj.com/site/about/guidelines.xhtml

Glasgow Clinical Trials Unit

STUDY FLOW CHART

FIGURE 0-1 THIS STUDY FLOW CHART SUMMARISES THE PATIENT JOURNEY TRHOUGHOUT THE STUDY

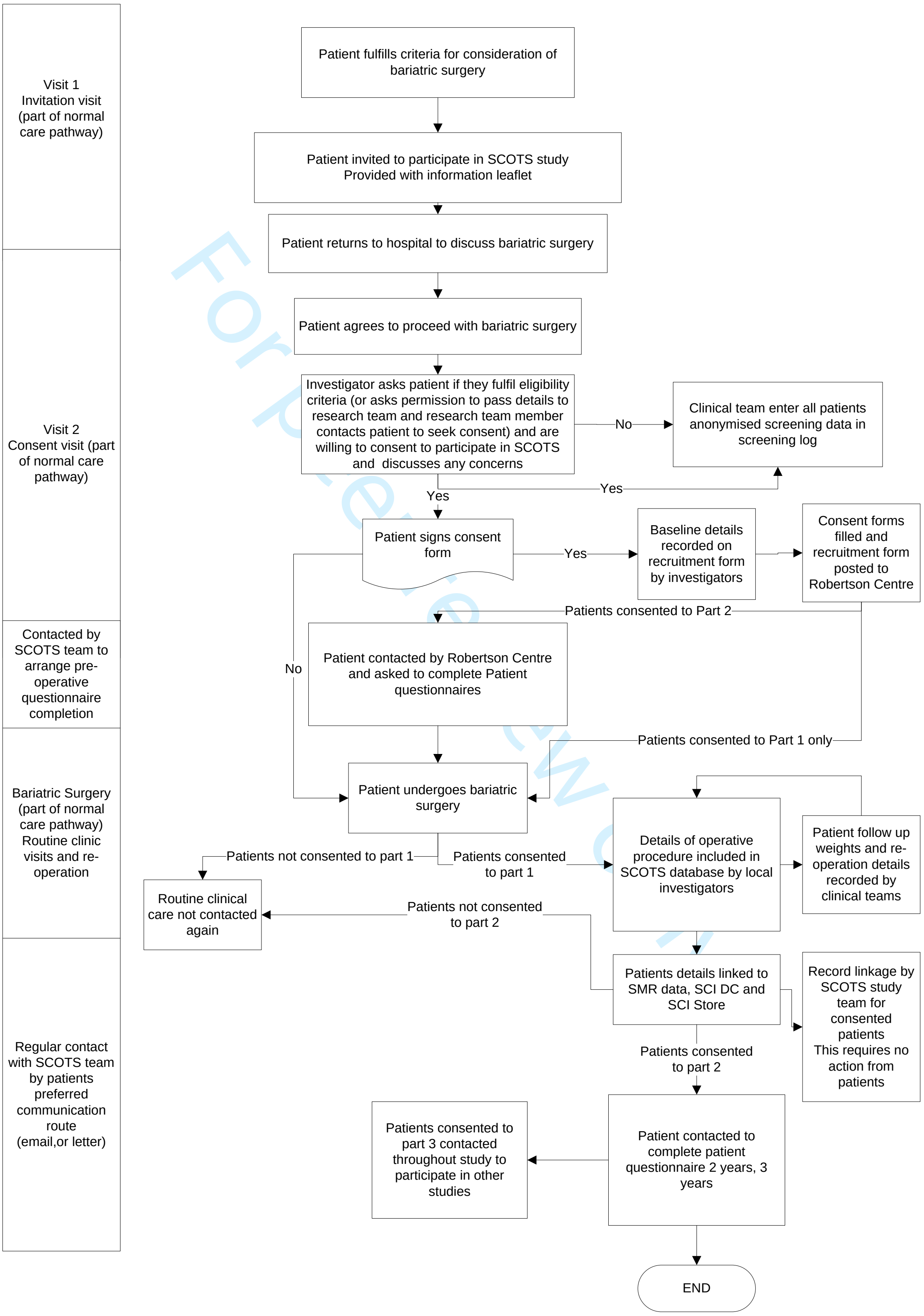

For peer review only - http://bmjopen.bmj.com/site/about/guidelines.xhtml

SCHEDULE OF ASSESSMENTS

TABLE 0-1: THIS SCHEDULE SHOWS EACH EVENT THAT INVOLVES THE PATIENT AND STUDY TEAM.

	Pre-surgery					Post surgery SCOTS Questionnaires	
Study Procedure	Pre-surgery Visit 1 1 year to six weeks before surgery	Pre-surgery Visit 2 1 year to six weeks before surgery	Pre-surgery Contact1	Pre-surgery Contact2	Surgery T0	2yr	3yr
Patients agree to proceed to bariatric surgery with clinical team	√						
Review Inclusion/Exclusion Criteria	√[a]						
Patients provided with patient information sheet	√						

For peer review only - http://bmjopen.bmj.com/site/about/guidelines.xhtml

Glasgow Clinical Trials Unit

Patient data entered onto screening log for SCOTS		√					
Patient return for pre-surgery clinic		√					
Clinical team ask permission from patients to pass details to research team who will contact patient (if not recruiting in local site)		(√)					
Obtain Informed Consent to participate in SCOTS study		√					
Patient baseline and contact details entered on registration form and sent to Robertson Centre for Biostatistics		√					
Consent form passed filed in Site File, copy given to patient and copy sent to RCB		√					
Contacted by SCOTS team to ask to complete pre-surgery questionnaire			√				

For peer review only

For peer review only - http://bmjopen.bmj.com/site/about/guidelines.xhtml

Patient completes pre-surgery questionnaire				√			
Patient undergoes bariatric surgery					√		
Clinical teams record operative details for patient post surgery					√		
Clinical teams record weight and re-operation details						√	√
Patients contacted to remind them to complete post surgery questionnaire						√	√
Part 3 consented patients may be invited to participate in other studies	Ongoing throughout study						
Record linkage to SCI-Store, SCI – DC, SMR (ISD), GRO throughout study	Ongoing throughout study, no interaction with patient unless to clarify if admission was a complication of bariatric surgery.						

For peer review only

For peer review only - http://bmjopen.bmj.com/site/about/guidelines.xhtml

1 INTRODUCTION

1.1 Background

SCOTS is a prospective cohort study commissioned by the Department of Health (DoH) National Institute of Health Research (NIHR) which will follow bariatric surgery patients for 3 years after their surgery. The NIHR commissioning brief for this study stated: "Obesity is a growing problem in the United Kingdom, with a growing number of people among the morbidly obese. Concomitantly, there is an increase in requests for bariatric surgery but insufficient evidence of long term effectiveness and safety of these procedures. There are existing registries of bariatric surgery, but these suffer from problems of collected data, completeness of follow- up, or data availability for secondary analysis. There is a need for a long term study of bariatric surgery, so that the outcomes and complications of different procedures, their impact on quality of life and nutritional status, and the effect on co-morbidities can be monitored in both the short and the long term." A recent Health Technology Assessment (HTA) (1) concluded that "Bariatric surgery is a more clinically effective intervention for treating obesity compared to non-surgical options" however they qualified this with "Uncertainties remain over the long-term outcomes of bariatric surgical interventions, particularly their impact on weight-related health problems and patient quality of life. Further studies will be required". This study was awarded NIHR HTA funding to address some of the uncertainties around the clinical effectiveness of bariatric surgery in the longer term. The original plan included a 10 year follow up period, however, due to unforeseen recruitment issues (reductions in both NHS & private surgical numbers) the study & statistical plans were reviewed. Following discussions with the NIHR it was agreed that many objectives could still be met from the number of patients currently recruited over a shorter period of follow up (3 years). The protocol has been updated to reflect these changes. Clearly some patients will have completed the 1 month, 6 month and 1 year post-operative questionnaire already. Where appropriate, these data will be used to supplement the year 2 and 3 questionnaires and the health record data in order to deliver the study objectives.

1.2 Rationale

The efficacy of bariatric surgery for large scale, long term weight loss is well established (2). However, many questions remain over the medium term benefits of that weight loss for health, particularly when accounting for the potential complications of surgery. Given that the proportions of the population who are obese is increasingly rapidly, further information is required as to who benefits most from bariatric surgery.

For peer review only - http://bmjopen.bmj.com/site/about/guidelines.xhtml

1.2.1 Definition of bariatric surgery

A bariatric surgical procedure is defined as a surgical intervention which has the primary purpose of large scale weight loss in a patient who is obese. Currently that includes gastric bypass, sleeve gastrectomy and laparoscopic gastric banding. The inclusion of emerging techniques in this definition will be decided by the SCOTS Steering Committee (SCC).

1.2.2 Clinical Outcomes of weight loss surgery

There are some pre-existing data on clinical outcomes with 10 year mortality rates being the best known. Overall mortality was found to be 29-40% lower in patients receiving bariatric surgery compared to Body Mass Index (BMI)-matched subjects not receiving surgery (3;4). Although impressive, these figures are from studies which initially recruited in the 1980s and early 1990s and further work is required to update the mortality rate in the current population, now that primary prevention measures for cardiovascular disease, such as statins and ACE-inhibitors, have lowered the risk of premature mortality in at risk subjects, including those who are obese or have diabetes.

Diabetes improvement post bariatric surgery has also been of interest. Dixon et al conducted a randomised controlled trial of laparoscopic gastric banding in patients with newly diagnosed type 2 diabetes resulted in a 73% remission rate after 2 years (5). Similar results have been shown for gastric bypass using meta-analysis of heterogeneous, mainly retrospective, cohort studies (6). Whether remission is maintained beyond the first few years post surgery is unknown. However, the main outcomes of interest are the longer term effects on the development of diabetes complications such as retinopathy, nephropathy and cardiovascular disease and those are not established.

The effect of bariatric surgery on cardiovascular outcomes has not been ascertained. It is known that HDL cholesterol is less likely to be low and hypertriglycerideamia is less common after surgery (7), as expected by a procedure which will reduce intra-abdominal adiposity and thus insulin resistance, but a reduction in cardiovascular events has not been shown. Hypertension, which would be presumed to decrease, in fact shows no difference 8 years post surgery, once the initial antihypertensive effects of acute weight loss in the first few years post-surgery are accounted for (8). Many have used cardiovascular risk factors measured during the acute weight loss phase, applied a population based cardiovascular risk score and stated a reduction in cardiovascular risk; this is of course a flawed use of such a score based on temporarily reduced cardiovascular risk factors. So, while the general population and the majority of physicians may assume that weight loss with bariatric surgery reduces the risk of a cardiovascular event, this is currently unknown.

What is established is that health related quality of life (HR-QoL) is increased compared to pre-operative levels (9). When patients who decide to proceed to surgery are compared to those who opt for non-surgical treatment only, the baseline HR-QoL is far lower in those

For peer review only - http://bmjopen.bmj.com/site/about/guidelines.xhtml

who select surgery, showing at least a perception of reduced quality of life in these individuals (10). To what extent the improvements seen in HR-QoL correlate with weight loss, complications and clinical outcomes is not known.

The complication rates of bariatric surgery are poorly reported in the literature, leading to polarised views on their true incidence. Mortality rates in the first year post surgery have been reported as being similar to those of BMI-matched controls (11); however, the same results may not be found today due to the decreased mortality rates as a result of primary preventative. There is little report of specific wound complications and re-operation rates, with a general belief that the low rates reported by the few studies releasing this data would not be replicated in routine practice.

1.3 Study hypothesis

This study is a registry of bariatric surgery and is therefore the study design is based on objectives but not hypotheses.

2 STUDY OBJECTIVES

SCOTS is a prospective observational cohort study of 450 patients undergoing bariatric surgery in Scotland which will be used to assess medium term outcomes and complications of bariatric surgery. This is in order to identify risks and complications of bariatric surgery and develop an evidence base to support clinical teams in building a patient centred, safe, effective and efficient bariatric surgery service.

2.1 Primary Objective

The SurgiCal Obesity Treatment Study (SCOTS) will establish the clinical outcomes and adverse events of different bariatric surgical procedures (taking in to consideration different care pathways), their impact on quality of life and nutritional status, and the effect on co-morbidities in a cohort of 450 patients.

2.2 Secondary Objectives

To establish in a cohort of obese patients who are undergoing bariatric surgery:

1. The physical and mental health and social burden of severe obesity.
2. Incidence of acute and chronic postoperative complications. Acute complications, defined as up to three months post-surgery, will include surgical site infection; chronic complications will include revisional surgery, plastic surgery and chronic pain, for different bariatric surgical procedures.

For peer review only - http://bmjopen.bmj.com/site/about/guidelines.xhtml

3. The effect of surgical experience and the pre and post-operative care pathway on complication rates and weight loss, for different bariatric surgical procedures.
4. The effect of the pre-surgical pathway and criteria on bariatric surgery patient selection. Change in health related quality of life, anxiety and depression over time pre- and post-operatively for a mean of 3 years from date of bariatric surgery.
5. The weight status pre and post-operatively for 3 years after bariatric surgery.
6. The glycaemic control, lipids, blood pressure, medication prescription, and rate of diabetes complications (microalbuminuria and renal disease, and retinopathy) in those that have pre-existing diabetes or develop diabetes for 3 years follow up since bariatric surgery.
7. Changes in socio-economic factors (employment, benefit receipt, sick leave, and health care use) for 3 years since bariatric surgery.

All the above objectives are due to be achieved by December 2020 (Start Jan 2012, 2 years development, 3 years recruitment finishing March 2017, mean 3 years post-operative follow-up finishing December 2020).

2.3 Primary Outcome

Change in weight from pre-bariatric surgery weight.

2.4 Secondary Outcomes

1. Incidence of physical and psychological comorbidity in a population seeking bariatric surgery

In the 3 years following bariatric surgery:

2. Change in weight/BMI from pre-bariatric surgery weight
3. Rate of incident type 2 diabetes
4. Change in incidence of depression and anxiety compared to baseline level pre-operatively
5. Incidence of complications immediately post-operatively and the need for readmission for revisional procedures
6. Change in health-related quality of life compared to baseline level pre-operatively
7. Change in glycaemic control, cardiovascular risk factors, CKD, retinopathy and medications prescribed in patients with diabetes compared to equally obese patient with diabetes who did not have bariatric surgery
8. Cost of the procedure and follow-up

Outcomes, including their source and frequency of measurement, are summarised in table 1.

For peer review only - http://bmjopen.bmj.com/site/about/guidelines.xhtml

3 STUDY DESIGN

3.1 Overview of study design

This 3 year prospective cohort study will collect data on the medium term outcomes of bariatric surgery in a minimum of 400 individuals. The study will be performed according to the Research Governance Framework for Health and Community Care (Second edition, 2006) (12). The information required to meet the objectives will be collected by a combination of sources including: bariatric surgery specific information entered by clinical teams; data linkage to routine clinical data and patient reported outcome measure questionnaires (PROMS). The outcomes and the data source are described in detail in Table 3-1~~Table 3-1~~.

TABLE 3-1: PRIMARY AND SECONDARY OBJECTIVES OF SCOTS DESCRIBING SOURCES OF OUTCOME DATA

	Outcome	Data source	Frequency
1	Incidence of physical and psychological comorbidity in a population seeking bariatric surgery.	SCI-DC Scottish National dataset, eDRIS at National Service Scotland, Patient reported questionnaires.	Continuous throughout study
2.1	Change in weight/BMI from pre-bariatric surgery	Clinical teams	At recruitment, annual check up
2.2	Rate of incidence of type 2 diabetes	SCI-DC Scottish National dataset	Continuous throughout study
2.3	Change in incidence of depression and anxiety compared to baseline levels pre-operatively	eDRIS at National Service Scotland, Patient reported questionnaires	Pre-operatively, ,2,3 years post operatively
2.4	Incidence of complications immediately post-operatively and the need for revisional procedures	Patient reported questionnaires, surgical teams	Pre-operatively, 2, 3 years post operatively, at time of re-operation.
2.5	Change in health related quality of life compared to baseline	Patient reported questionnaires	Pre-operatively, 2,3years post operatively
2.6	Change in glycaemic control, cardiovascular risk factors, chronic kidney disease (CKD), retinopathy, medications in patients with	SCI-DC Scottish National dataset	Pre-operatively, 2, 3 years post operatively, at time of re-operation.

For peer review only - http://bmjopen.bmj.com/site/about/guidelines.xhtml

	diabetes compared with non-obese cohort		
2.7	Cost of the procedure and follow-up (to inform cost effectiveness analysis)	Clinical teams, e-prescribing record linkage, patient reported outcomes	At time of patients operation, continuous linkage throughout the study

This protocol describes the objectives of the study, the sources of data collection for each objective, and the input required from patients and clinical teams throughout the study. A data collection portal will be developed for the study with secure logins for clinical teams and participating patients. A detailed user manual will be developed for the operation of this system will be produced by the Robertson Centre for Biostatistics - University of Glasgow.

A SCOTS web based portal will be developed which will present information on the study to the public and other researchers. The portal will contain secure logins to the Robertson Centre databases which will allow clinical teams to enter data. Clinical teams will be asked to register for a user ID and password. These will be issued once the team have undergone the necessary training for their delegated role. Everyone who has a user ID and login will be a member of the SCOTS investigators group.

The SCOTS portal will include an area which will allow patients who have agreed to complete the patient questionnaires to login securely and complete the patient reported outcome questionnaires. If patients prefer to complete the questionnaires on paper the data will be entered by staff at the Robertson Centre into the database. Patients will be kept up to date with progress of the study via updates on the study website. Patients will be sent reminders through their preferred route of communication to let them know when they are due to complete a questionnaire.

Patient identifiable information will be stored separately from the clinical outcome data. The information will be linked using a database code. Within the Robertson Centre individuals who can access patient information to send reminder letters will not have access to any clinical information. Any clinical information to be analysed will have all patient contact details remove. Production of any reports will follow the NHS Information and Statistics division (ISD) Statistical Disclosure Control protocol (13) to ensure that patient confidentially is maintained, (this is a requirement of the record linkage arrangement with ISD).

SCOTS has three distinct parts. All adult patients who are having weight loss surgery in Scotland will be invited to participate in three parts of the study. These parts are described in detail below.

For peer review only - http://bmjopen.bmj.com/site/about/guidelines.xhtml

3.1.1 Part 1 Record Linkage

The study will collect data by record linkage to patients' clinical outcomes. Information will be recorded by the clinical team on the operation they have had. They will be followed using their medical records until at least December 2020 and if funding is available for the rest of their life. This will include all hospital admissions, blood test results, diagnostic test results, diabetes complications, medications they are prescribed, pregnancy, psychiatry and mortality. This part of the study will observe patients care. The study will not alter their planned care in any way. No additional tests or treatments will be given to patients who consent to be part of the study. (This will fulfil the primary objective and objectives 1, 2, 3 and 4). If patients do not consent to participate in this part of the study they will not be asked to participate in Part 2 or Part 3.

Each record linkage requires approvals to access data including a favourable ethical opinion. These will be sought by the SCOTS Project team. A separate procedure for each data linkage will be developed by the Robertson Centre for Biostatistics. Patient identifiable information is required to identify study participants. The information required is patient CHI number, family name, given name, patient date of birth, gender, postcode, consent data and unique study identifier.

3.1.2 Part 2 Patient Reported Outcomes (PROMS) Questionnaire

We will ask patients if they are willing to complete a questionnaire which will include questions on quality of life, overall health and fitness, diet, and anxiety and depression, healthcare utilisation and ability to work. They will also be asked about problems they may have experienced since their surgery such as pain and surgical site infection post discharge.). Where patients are not completing PROMS and there is no reliable clinical weight record, we will write after 3 years requesting completion of a weight questionnaire (simply asking their current weight). The patients will be offered a voucher based incentive of £30 for completing both the year 2 and 3 questionnaires (high street voucher), and £10 for the year 3 weight follow-up questionnaire (for those no longer in clinical follow-up/not completing PROMS)

3.1.3 Part 3: Registration for a database of bariatric patients for future research

Patients who have agreed to Parts 1 and 2 will be asked if they are willing to be contacted about possibly taking part in other studies. This will not be an obligation to take part, just an expression of interest in being invited to take part. This will create a database of patients who have had bariatric surgery and are willing to consider participation in future research. Ethical opinion will be sought for the development of these study protocols.

For peer review only - http://bmjopen.bmj.com/site/about/guidelines.xhtml

3.1.4 Service user involvement

SCOTS will uphold the principles embodied by NIHR INVOLVE in relation to user involvement in research, to provide training and support, negotiated roles, time and equitable access for service users involved in this study. SCOTS recruited a patient focus group made of service users who have undergone different procedures both in NHS and private healthcare.

The SCOTS patient focus group have commented on the design of the study, in particular by providing advice on the acceptability of the patient reported outcome questionnaires, the burden to patients and on material and resources that will be given to potential participants. Future sessions are planned to discuss patient recruitment and retention strategies. SCOTS have included a service user on the SCOTS Steering Committee.

3.2 Study Population

All patients due to have a bariatric surgical procedure in Scotland will be invited to participate - both NHS and private sector.

A bariatric surgical procedure is defined as a surgical intervention which has the primary purpose of large scale weight loss in a patient who is obese. Currently those procedures include: duodenal switch; gastric banding; gastric bypass and sleeve gastrectomy. The inclusion of emerging techniques in this definition will be decided by the steering committee.

3.3 Reference populations

Within this protocol the objectives will be met by comparing before and after surgery outcomes for the SCOTS cohort. Additional funding shall be sought to develop protocols to allow comparisons with the following groups for a final Health Technology Assessment report.

1. SCOTS will compare outcomes with the IT based non-surgical weight management database in Greater Glasgow and Clyde (treatment seeking population with current data on 10,000 patients and 3,000 new referrals per year) which has prospective consent for research allowing data linkage for morbidity and mortality along with adjustment for smoking, deprivation, BMI, age and sex.

2. Standardised mortality ratios are available for a BMI matched cohort from the Scottish Health Survey.

For peer review only - http://bmjopen.bmj.com/site/about/guidelines.xhtml

3. The Scottish diabetes database allows an anonymous control group matched for key demographics to be created with prospective follow up of diabetes care and complications, compared to those with diabetes who had bariatric surgery.

3.4 Inclusion criteria

- Patients aged 16 years or over undergoing their first bariatric surgery in NHS hospitals or private practice in Scotland.
- Capacity to consent
- Resident in Scotland
- Written informed consent

3.5 Exclusion criteria

- Does not meet inclusion criteria
- Patients who have had previous weight loss surgery (at time of potential recruitment are undergoing a repeat procedure)
- No written informed consent
- Patients under 16 years
- Patients who do not have capacity to consent for themselves
- Weight loss surgery performed at a site outside Scotland
- Patients who live outside Scotland
- Patients who do not have strong enough English language skills to understand the patient information leaflet without assistance will be excluded from Part 2 of the study, the patient questionnaires. If they require a translator to understand the information they may be able to participate in Part 1 of the study which requires no further interaction from the patient. This decision will be made by the clinical teams caring for patients.

3.6 Identification of participants and consent

Patients will be prospectively identified by clinical teams as they are referred for surgery. Patients will be recruited from both NHS and private bariatric surgical practice in Scotland. They will either be recruited by a member of the surgical team prior to undergoing a bariatric surgical procedure, including any significant pre-operative weight loss (such as a very low energy diet for liver shrinkage or use of an intra-gastric balloon) which is considered part of the surgical pathway, or the surgical team will ask the patient's permission to pass over their name and contact details to the SCOTS research team who will then contact the patient directly to give further information and obtain fully informed consent.

For peer review only - http://bmjopen.bmj.com/site/about/guidelines.xhtml

Depending on local patient pathways patient information sheets will be provided to patients at a clinic visits or group session. This will be provided at least 24 hours before the patient will be asked to consent. In the case of those patients who are to be recruited by the research team (identified by surgical team), a patient information sheet will be provided when the permission is sought to hand over contact details to the research team. Patients who meet the eligibility criteria will be identified by their local clinical care teams. All patients undergoing bariatric surgery will be recorded on the screening log at their next appointment. This will allow patients to consider their participation and ask any additional questions they may have about the study face to face. An independent contact is provided on the patient information leaflet who patients can contact to discuss participation in research studies.

They will be asked to give fully informed consent for clinical data linkage (Part 1) and to be contacted by Glasgow University by post, email or phone about to completing the Patient questionnaires (Part 2) and for permission to contact them about future studies (Part 3). Patients who require a translator will only be asked to participate in Part 1. Only patients who agree to Part 1 will be eligible for Part 2 or Part 3.

3.7 Withdrawal of subjects

Participants have the right to withdraw from the study at any point for any reason. The SCOTS web site and patient information sheets will provide contact details for patients if they wish to withdraw from the study. If patients have asked to complete the forms by paper, contact details will be included within each paper questionnaire. The SCOTS team will provide patients with a form to complete, if they are contacted with a request to withdraw from the study.

If the SCOTS team are contacted by a patient who no longer wishes to participate in the study, they will be contact by the SCOTS team at University of Glasgow and asked to specify which following options they would like to apply to their data:

A. (For those within Part 1) I no longer wish to continue the study but I am content to allow my existing data to remain within the study for analysis.

B. (For those consented to Part 1 and 2). I am content for my records to be linked but I no longer wish to complete the patient questionnaires.

C. (For those consented to Part 1, 2 and 3). I am content for my records to be linked but I no longer wish to complete the patient questionnaires or be contacted regarding future studies.

D. For any patient I would like to completely remove my all data from the study and wish not to be contacted again by the study.

For peer review only - http://bmjopen.bmj.com/site/about/guidelines.xhtml

When a request is received from a patient to withdraw from the SCOTS study, a standardised form will be sent to the patient by their preferred communication route. The form will be completed by the patient including the patients name date of birth and address, and the on receipt data manager will flag the patient as having withdrawn from the study. The patient will be asked if they would like to be informed of the findings of the study even though they will no longer be participating in the study.

4 STUDY PROCEDURES

The study design involves local clinical teams identifying and potentially recruiting participants (some participants will be recruited by the SCOTS research team after identification by the local clinical team). Patients who consent to participate will have recruitment data collected by their clinical care teams or the SCOTS research team. Following their operation patients will have their weight loss surgery details entered into the database. The clinical teams then use the system to include follow up weights and re-operations. For the remainder of the study, patients who have consented to part 2 of the study will be asked to complete questionnaires and their health outcomes will be recorded by record linkage to routine data sets. A detailed breakdown of patient contacts with the study is described in Table 4-1~~Table 4-1~~ below.

TABLE 4-1: SCOTS PATIENT VISIT SCHEDULE: DESCRIBES EACH INTERATION WITH PATIENTS IF THEY CONSENT TO PART 1 AND 2 OF THE STUDY.

Patient contact	Time	Routine care	SCOTS	Part 1	Part 2	Part 3
1	1 year to 6 weeks before surgery	Agree bariatric surgery	Patients given/sent Invitation to participate letter and patient information sheet (and asked if they are to be contacted by SCOTS research team for further information and informed consent if no local recruitment)	1	1	
2	1 year to 6 weeks before surgery	Patient pre-surgical clinic visit, dietician, psychologist if local recruitment; phone call from SCOTS research team member if research team recruitment	Patients asked if they are willing to consent to participate in SCOTS, Patient signs consent forms, clinical team and patient complete contact details and baseline height and weight.	1	1	
3	At least one month before surgery		Patient contacted by SCOTS team to complete pre-operative questionnaire	1	1	
4	Date of surgery	Patient has weight loss operation	Clinical team enters details of weight loss operation to SCOTS portal	1	1	

For peer review only - http://bmjopen.bmj.com/site/about/guidelines.xhtml

5	Patient admitted to hospital	Identified by record linkage	If patient hospital admission is possibly related to bariatric surgery, patient will be contacted by SCOTS Team.	1	1	
7	At routine follow up visits	Patients attend routine clinical visits	Clinical teams enter weight and re-operation details		1	
8	Two year post surgery	Patients have routine annual blood test and results are made available via SCI-store.	Patients complete two year post surgical questionnaire		1	
9	Three years post surgery	Patients have routine annual blood test and results are made available via SCI-store.	Patients complete three year post surgical questionnaire		1	
10	Any point after recruitment		Possibly contacted to participate in future studies			1
11	Three years onward	Patient continues to have routine annual blood test every year	Patient contacted to thank them for completing the PROMS. Record linkage continues. Patients will be informed about future SCOTS publications.		1	
Total				5	9	1

Figure **4-1**~~Figure 4-1~~ shows what happens at each point within the study. This includes background record linkage and patient contacts.

FIGURE 4-1: BASELINE – PRIOR TO OPERATIVE WEIGHT LOSS (INCLUDING PRE LIVER-PREP DIET OR BALLOON PHASE)

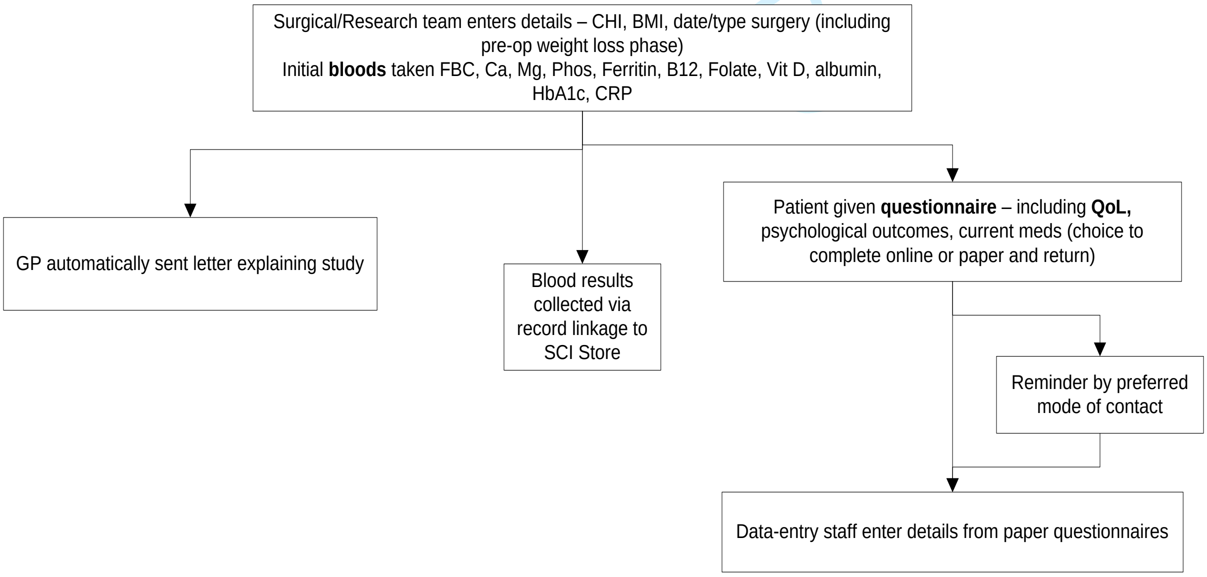

For peer review only - http://bmjopen.bmj.com/site/about/guidelines.xhtml

For peer review o

FIGURE 4-2: 2 AND 3 YEARS POST DEFINITIVE SURGERY

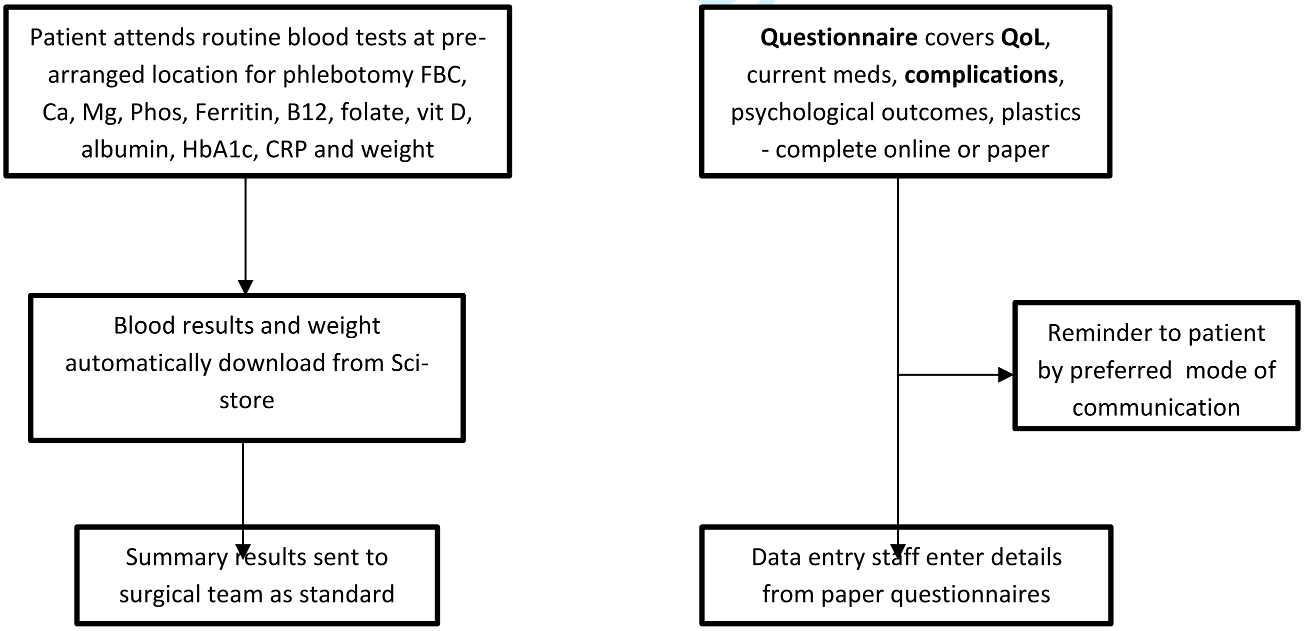

For peer review only - http://bmjopen.bmj.com/site/about/guidelines.xhtml

FIGURE 4-3: PART 3 IF NEW BARIATRIC SURGERY STUDY BEGINS

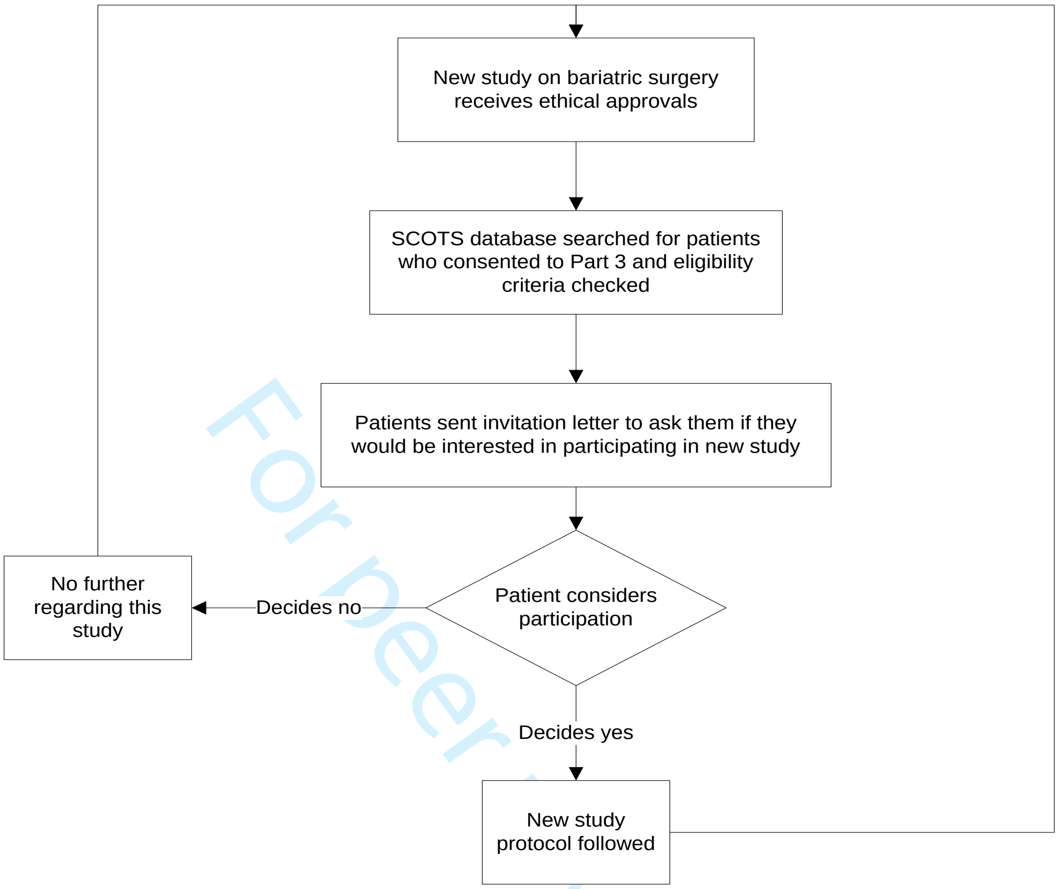

4.1 Duration of participation

Part 1: if funding is available, record linkage, follow up of patients will continue throughout their life. The end of a patient's life will be shown by linkage to the GRO data set. This will be used to flag deceased patients.

Part 2: patient reported outcome measures (PROMS) questionnaires will be collected for a maximum of 3 years. Collection of PROMS will stop after December 2020.

Part 3: Patient contact details will be kept by the study team until the end of the patient's life. The end of a patient's life will be shown by linkage to the GRO data set. This will be used to flag deceased patients and they will be removed from the database in Part 3 to ensure patients families are not contacted after their death. Patient will only be contacted for future studies if record linkage is available to show that they are still living.

Patients will be able to withdraw from the study at any point. Data collected will be retained unless explicitly requested to be removed from the study by the patient.

For peer review only - http://bmjopen.bmj.com/site/about/guidelines.xhtml

4.2 Study schedule

This section provides a brief overview of the procedures for investigators at each point within the study. SCOTS will integrate into routine care pathways for bariatric surgery as much as possible. No additional clinic visits will be required. There are five points within the patient journey that the clinical/research team are required to undertake any tasks. These points are described in detail in the following sections.

4.2.1 Start of Study - Surgical team registration

Each site will be defined before the start of the study and login and passwords will be created for each principal investigator and their clinical teams.

A training plan will be developed for clinical teams. Clinical teams will be provided with an overview of the study provided with a training package which will be developed electronically, to be completed at their convenience, electronic guidance will be integrated into the data collection tool to assist them. A user manual will be created which will explain the data which is required for each field and define each data field.

An electronic form will be used to collect surgeon specific data the first time they log onto the bariatric data collections system. This form will be developed by the study Data Centre at the Robertson Centre for Biostatistics, University of Glasgow and access to the form will be restricted, with only authorised site-specific personnel able to make entries or amendments to their data. A manual will be developed to assist investigators with use of the electronic data collection system. It is the investigator's responsibility to ensure completion and to review and approve all data captured. Surgeons will be asked to outline which locations they work in, which procedures they use, and the details of these procedures. They will also be asked information on their surgical experience including date when they qualified, number of procedures the previously year, total number of procedures undertaken in the last five years.

The aim of this task is to ensure that surgeons' data is entered at the start of the study and patient records can be pre-populated to ensure that data can be collected as efficiently as possible.

Checklist:	
Surgeon registers on SCOTS system	
Surgeon enters names and email addresses of clinical team who will be part of SCOTS investigators team	
Investigators have the opportunity to view SCOTS training materials	
SCOTS resource pack is available at the site	

For peer review only - http://bmjopen.bmj.com/site/about/guidelines.xhtml

4.2.2 Visit 1 Routine care invitation visit

Patient pathways vary throughout Scotland, invitation and introduction should take place at either an individual's clinic visit or at a group session discussing weight loss surgery. The aim of this visit is to ensure that patients who are planning weight loss surgery are aware of the SCOTS study, why it is being undertaken and what would be required from them.

4.2.2.1 Invitation visit protocol (in sites with local team recruitment)

1. Patient attends routine clinic to discuss bariatric surgery with clinical team.
2. Patient provided with an overview of SCOTS
3. Patient provided with opportunity to ask initial questions
4. Patient provided with invitation to participate in SCOTS and patient information leaflet (pre-prepared pack).

Checklist:	
Patients provided with overview of SCOTS	
Patient provided with SCOTS information pack	

4.2.2.2 Invitation visit protocol (in sites with SCOTS research team recruitment)

5. Patient attends routine clinic to discuss bariatric surgery with clinical team.
6. Patient provided with a summary overview of SCOTS
7. Patient provided with invitation to participate in SCOTS and patient information leaflet (pre-prepared pack).
8. Patient asked permission for surgical team to pass on contact details to SCOTS research team
9. Participant Identification form completed and returned to research team

Checklist:	
Patients provided with a summary overview of SCOTS	
Patient provided with SCOTS information pack	
Permission sought and Participant Identification Form completed and returned	

4.2.3 Visit 2 Recruitment visit

At the next follow up clinic appointment patients will be asked if they wish to take part in SCOTS. Patients must have been given more than 24 hours to consider their participation.

For peer review only - http://bmjopen.bmj.com/site/about/guidelines.xhtml

For patient's in sites where recruitment is carried out by the SCOTS research team the following will occur after the research team receive the participant information form from the local site:

1. SCOTS research team contact patient via telephone
2. Patient provided with an overview of SCOTS
3. Patient provided with opportunity to ask initial questions

Details of all patients who plan to have bariatric surgery will be recorded on a screening log. These logs will recorded on paper. Every patient who plans bariatric surgery will be logged anonymously with details of the individual's age category on date of invitation, gender, calendar quarter surgery planned, if they meet the SCOTS eligibility criteria, if they consent and type of surgery planned (for participants identified via local sites but recruited by the SCOTS team only those giving permission for their details to be forwarded to local teams will have a screening log completed as the research team shall do this). The purpose of this log is to demonstrate if the SCOTS data set is representative of all patients who are having bariatric surgery in Scotland. It will also provide a record of number of patients who consent. A copy of the screening logs will be sent to the Robertson Centre on an annual basis.

This is an anonymised screening record and patients who do not wish to participate will have no further information collected.

4.2.3.1 Informed consent protocol (in sites with local team recruitment)

1. Ask the patient if they have any questions relating to the study documentation. Ensure that the patient fully comprehends the information given.
2. Explain that participation is voluntary and should they decide to proceed with participation in the study, they are free to withdraw at any time without giving an explanation.
3. If a patient chooses to decline, thank them for their time and allow them to leave.
4. If they are unsure, allow further time to consider and arrange a convenient date to meet again reiterating that their participation is voluntary. Offer the option to speak to another member of the study team or an independent advisor.
5. All the check boxes within the consent form must be initialled by the patient to confirm they understand each part of the study. Ask the patient to initial each part of the consent form and sign the bottom section adding their name in block capitals.
6. The person obtaining informed consent signs and dates the consent forms adding their name in block capitals.
7. If a witness is required they can also sign and date the consent form (this may be in the case where an interpreter is required).
8. One copy of the form should be provided to the patient, one filed in the patient's notes and one copy stored in the site folder, and a copy sent to the Robertson Centre for the study files. A copy of the version of the patient information leaflet which was given to the patient should be filed within the site study file.

For peer review only - http://bmjopen.bmj.com/site/about/guidelines.xhtml

9. If the patient consents to the study the patient recruitment form should be completed in the presence of the patient. Record patient name and CHI number (for NHS patients), sex, height and weight.
10. Patients should be asked to complete the contact details part of the form including their e-mail and mobile phone contact details and ensure that they identify their preferred route of communication.
11. The Patient recruitment form will be send to the Robertson Centre and a copy filed within the site study file; When they are sent to the Robertson Centre a form will be faxed to show what documentation has been sent in the post, this will be recorded in a postage log.
12. Paper copies of the screening log are returned to the Robertson Centre annually, in self addressed envelopes provided by the SCOTs project team. These will also be recorded in a postage log.

Checklist:	
Check patient name and date of birth correct on patient recruitment form	
Consent from signed and dated at each point	
Patient retains copy of consent form	
Copy of consent form filed in notes	
Copy of consent form in study file	
Copy of consent form in study file sent to Robertson Centre	
Copy of patient recruitment form sent to Robertson Centre	
Copy of patient recruitment form filed in study file	
Consent form and patient recruitment form entered on postage log	

4.2.3.2 Informed consent protocol (in sites with SCOTS research team recruitment)

1. When the Participant Identification Form is received from the surgical team start to complete the SCOTS Recruitment Log and contact the patient via telephone.
2. Ask the patient if they have any questions relating to the study documentation. Ensure that the patient fully comprehends the information given.
3. Explain that participation is voluntary and should they decide to proceed with participation in the study, they are free to withdraw at any time without giving an explanation.
4. Complete the screening log (do not add participant ID as form is to be anonymous)
5. If a patient chooses to decline, thank them for their time and allow them to leave.
6. If they are unsure, allow further time to consider and arrange a convenient date to phone again reiterating that their participation is voluntary. Offer the option to speak to another member of the study team or an independent advisor.
7. All the check boxes within the consent form must be initialled by the patient to confirm they understand each part of the study. Ask the patient to initial each part of the consent form and sign the bottom section adding their name in block capitals.
8. For participants should then be asked post the completed consent form back to the research team in the stamped addressed envelope provided.

For peer review only - http://bmjopen.bmj.com/site/about/guidelines.xhtml

If the research team does not receive the completed consent form from the patient within two weeks and cannot reach the patient by telephone, a follow up letter should then be sent.

9. On receipt of the signed form, the person that obtained informed consent signs and dates the consent forms adding their name in block capitals.
10. If a witness is required they can also sign and date the consent form (this may be in the case where an interpreter is required).
11. One copy of the form should be provided to the patient (posted back in case of SCOTS team recruitment), one filed in the patient's notes and one copy stored in the site folder, and a copy sent to the Robertson Centre for the study files. A copy of the version of the patient information leaflet which was given to the patient should be filed within the site study file.
12. If the patient consents to the study the patient recruitment form should be completed with the patient via the telephone. Record patient name and CHI number (for NHS patients), sex, height and weight.
13. Patients should be asked for their contact details including their e-mail and mobile phone contact details and ensure that they identify their preferred route of communication.
14. The Patient recruitment form will be send to the Robertson Centre and a copy filed within the site study file; When they are sent to the Robertson Centre a form will be faxed to show what documentation has been sent in the post, this will be recorded in a postage log.
15. The Letter to Inform Surgeon of Participation should be completed and sent to the surgeon along with the relevant operation details recording form.
16. Paper copies of the screening log are returned to the Robertson Centre annually, in self addressed envelopes provided by the SCOTS project team. These will also be recorded in a postage log.

Checklist:	
Check patient name and date of birth correct on patient recruitment form	
Consent from signed and dated at each point	
Patient sent copy of signed consent form	
Copy of consent form filed in notes	
Copy of consent form in study file	
Copy of consent form in study file sent to Robertson Centre	
Copy of patient recruitment form sent to Robertson Centre	
Copy of patient recruitment form filed in study file	
Consent form and patient recruitment form entered on postage log	

For peer review only - http://bmjopen.bmj.com/site/about/guidelines.xhtml

4.2.4 SCOTS study team contact patient

If the patient has consented to Part 2 of the study, the patient will be contacted by staff from the Robertson Centre by their preferred route of communication to invite them to complete a patient reported outcomes questionnaire. They will be provided with a username and initial password which will need to be changed the first time they login to their account. The preadmission questionnaires will be available for the patient to complete. Patients will be able to complete the questionnaire in one or more sittings. If the patients have not logged into the system within two weeks of receiving their information they will be sent a reminder message by their preferred mode of contact. Given the smaller numbers recruited, the patients are now being offered a voucher based incentive to ensure a good return of the two and three year questionnaires. They will be offered an incentive of £30 for completing both the year 2 and 3 questionnaires, and £10 for the year 3 weight follow-up questionnaire (for those no longer in clinical follow-up/not completing PROMS).

Patients will be contacted two and three years after their weight loss operation. Patients will be contacted as part of the study closure phase to thank them for their participation in the study and inform them that they are no longer required to complete the questionnaires.

4.2.5 Visit 3 Weight loss operation

After the patient has their weight loss operation, the clinical teams will enter data onto the SCOTS data collection tool. This will involve the team member who has been delegated to record surgical information on the system. They will log in and be asked for the location of the surgery, which surgeon operated on the patient and their usual operation details will be pre-populated. If the operation is in any way different from the surgeon's usual technique, an edit function will allow the exact details of the operation to be recorded.

If the operation is a re-operation on an existing SCOTS consented patient, the SCOTS database will be searched to allow the patient's re-operation to be recorded.

4.2.6 Visit 4 -Post operative follow up

When patients attend follow up visits to see their surgeon, clinical teams will record the patient's weight and any complications experienced by the patient. If the patient has a gastric band, clinical teams will be able to record band adjustments in the data collection system. Essential fields for the follow-up visits are date of appointment and weight. Within the SCOTS portal it is possible for clinical teams to produce individual reports for patients on their weight loss for inclusion in their medical records, in the letters to GPs or for the patients themselves.

For peer review only - http://bmjopen.bmj.com/site/about/guidelines.xhtml

4.2.7 Visit 5 - Re-operation (If applicable)

If a patient requires a re-operation, surgeons will be able to record details of the latest procedure and the reasons for the re-operation. This can happen at any time throughout the study. Details of how the surgeons will record re-operations will be included within the user manual.

For peer review only

For peer review only - http://bmjopen.bmj.com/site/about/guidelines.xhtml

5 STATISTICS AND DATA ANALYSIS

5.1 Statistical Analysis Plan

The study will have separate detailed Statistical Analysis Plans to cover each analyses required for reporting to study committees, for publications and for the final report to the study funder, which will be authored by the study statistician and agreed by the Study Steering Committee before analysis takes place.

An important goal within the funded period is to build various predictive models to use accumulating information to predict good and bad outcomes, so that clinicians can identify treatment opportunities and make better informed intervention management decisions. Generically, these prediction models will relate potentially predictive information – both on entry into the SCOTS database on and around diagnosis, and from subsequent visits and at surgical and other treatment interventions – to outcomes of interest. The mainstay will be Cox proportional hazards time-to-event models, incorporating demographic, clinical, laboratory and life event data (e.g. hospitalisations, strokes, cancers, non-weight loss surgery, and so on) as both fixed and time varying covariates. The influence of missing data on the robustness of predictions in these models will be assessed. Importantly, a hierarchy of models will be built, with ease of use for the clinician in mind, from very simple models built from easy to obtain information, through to more complex models with very many and possibly some difficult and/or expensive to measure covariates.

5.2 Software for statistical analysis

The statistical software to be used for each analysis will be specified in the relevant Statistical Analysis Plan

5.3 Sample size

At the time of grant proposal writing, 230 operations were funded in NHS Scotland each year (of which approximately 60 are bypass). Bariatric surgeons performed an additional 270 private procedures per year and they were willing to commit to entering data (approximately 80 bypass). Therefore 500 procedures per year were expected to be entered into the database with a belief that as numbers of people with severe obesity (BMI >40) are rising rapidly, this number will increase despite financial constraints.

Previous recommendations from the NHS Scotland national planning forum (15)recommended that each health board should aim to provide 9 operations/100,000 population. Scotland's estimated population on the 30th June 2011 was 5,254,800 (16) (http://www.gro-scotland.gov.uk/files2/stats/population-estimates/mid-2011/j22829703.htm). This would mean around 470 operations per year within the NHS alone. This figure was expected to be reached over a number of years. Within private

For peer review only - http://bmjopen.bmj.com/site/about/guidelines.xhtml

healthcare bariatric surgery was estimated to be around 270 per year in Scotland (reported by personal communications from bariatric surgeons).

Given the potential for a surgical learning curve (17), new surgeon's data will be recorded but not analysed for the primary outcome until they have performed 50 operations. Collection of this early data will provide important information on the effects of the learning curve on patient outcomes.

From previous studies (3;4) we expected a 10-year mortality of around 5% (100 deaths). This sample size would allow the mortality rate to be estimated with 95% confidence to within +/- 1% (i.e. for a 5% 10-year death rate, the 95% confidence interval will be between 4% and 6%). We planned to compare this mortality rate with an age-sex matched healthy population from the Registrar General of Scotland's life tables (assumed known with no sampling error). 100 deaths was sufficient to allow us to build a predictive model for death post surgery (conventionally one requires around 10 events per prognostic covariate considered). An initial sample size of 2000 was proposed as it would easily provide adequate power for the original outcomes under investigation.

However, there have been a number of unforeseen recruitment issues that have impacted on the numbers stated above. In order to explore whether the current sample is likely to show meaningful results, the available statistical power for detecting 3 year difference in HbA1c and quality of life (physical and mental components) was calculated using the numbers of participants available as of 29th of July 2016. The majority of data to inform sample size were taken from papers where bariatric procedure was laparoscopic adjustable gastric banding (LAGB) as this is recognised as the least effective of the bariatric procedures so will give a conservative estimate of measure of effect. This shows that there is >99% power to show differences in these outcomes at three years with the current sample size.

In order to explore the likely event rate for cardiovascular events and deaths, we performed health record linkage for currently recruited participants, linking with inpatient care and death records. Follow-up is from the date of surgery, so for those patients that went on to have surery details entered (n=180), there are 272 separate admissions. Of those 272, there are 72 'emergency' admission, and 4 of these, in 3 patients, are 'circulatory disease' using the main condition only. The codes included for these hospital admissions are;

· Angina pectoris (x1)
· Acute MI (x1)
· Pulmonary embolism without mention of acute cor pulmonale (x1)
· Orthostatic hypotension (x1)

Using date of operation as the starting point, we have a total (crude) follow-up time of 203.52 years (the mean is 1.13 years and the median is 1.04 years). As the number of cardiovascular events is so low, it is impossible to extrapolate this to a future event rate at this time. It should be noted that the participants have been cleared as healthy for elective surgery meaning that it is unlikely that there would be many cardiovascular events in early follow-up.

For peer review only - http://bmjopen.bmj.com/site/about/guidelines.xhtml

Following discussions with the NIHR it was agreed that recruitment will stop at approximately 400 patients and these numbers will be sufficient to answer the majority of objectives initially set.

5.4 Management and delivery

The Robertson Centre for Biostatistics, part of the Glasgow Clinical Trials Unit, a fully registered UK CRN Clinical Trials Unit, will manage and analyse trial data.

For peer review only

For peer review only - http://bmjopen.bmj.com/site/about/guidelines.xhtml

6 STUDY CLOSURE

Part 1 Record linkage: under the current funding record linkage will continue until December 2020. If additional funding is available Part 1 record linkage will continue until the end of the patient's life. The end of a patient's life will be shown by linkage to the GRO data set. This will be used to flag deceased patient and they will be removed from the database in Part 3 to ensure patients families are not contacted after their death.

Part 2 Patient reported Questionnaires: The study will end when the steering committee agrees that one or more of the following situations applies:

- Observation has been undertaken until December 2020
- Sufficient patients have completed the 3 year post surgery questionnaire

Patients will be contacted to thank them for their participation in the patient reported outcomes part of the study.

Part 3 Bariatric Surgery Research Studies. Patients contact details will be maintained until the end of the patient's life.

7 Data Handling

Data for the SCOTS study will be collected from a number of sources. These are summarised in Table 3-1~~Table 3-1~~. A detailed data management plan will be developed by the Robertson Centre team, which will include verification of paper data entry and validation checks. A separate protocol will be developed for each data linkage.

7.1.1 Surgeon registration data

An electronic data collection form will be developed by the study Data Centre at the Robertson Centre for Biostatistics, University of Glasgow. These data will include the surgeons' usual procedures and registration of the clinical teams. These data will be used to complete the operation details for each patient. It will be the investigators responsibility to ensure that these data are entered correctly and are kept up to date.

7.1.2 Patient recruitment data

A paper case report form (CRF) will be used to collect patient contact details and height and weight at time of recruitment will be completed by the clinical teams when the patient has consented to participate in SCOTS. Patients will be asked to specify their preferred form of communication throughout the study. They will be told that they may be contacted via another route if they do not respond. This CRF will be developed by the study Data Centre at the Robertson Centre for Biostatistics, University of Glasgow. Competed forms will be checked by the clinical teams and sent to the Robertson Centre for initial data entry. These forms will be sent along with signed consent forms. It is the investigator's responsibility to ensure completion and to review and approve all data captured in the CRF. A standard

For peer review only - http://bmjopen.bmj.com/site/about/guidelines.xhtml

operating procedure will be used by the Roberson Centre to ensure that information is entered into the SCOTS database in an accurate and timely manner.

7.1.3 Surgical data collection

An electronic case report form (e-CRF) will be used to collect data on weight loss surgery procedures. On or shortly after the patient undergoes weight loss surgery a member of the clinical team will record specific information on the patient's weight loss operation. Each surgeon will have been asked to register the locations they operate in, which procedures they perform and details of their usual specifications. This will then populate the fields for a particular type of surgery and clinical teams will be asked to confirm that the usual techniques have been applied for each procedure. Access to the e-CRF will be restricted, with only authorised site-specific personnel able to make entries or amendments to their patients' data. It is the investigator's responsibility to ensure completion and to review and approve all data captured in the e-CRF, however, a member from the SCOTS study team, with the appropriate NHS Research Passport permissions, will visit the site staff at least once per year to ensure surgical and follow-up data are complete. Data will be validated at the point of entry into the e-CRF and at regular intervals during the study. Data discrepancies will be flagged to the study site and any data changes will be recorded in order to maintain a complete audit trail (reason for change, date change made, who made change).

7.1.4 Patient Reported Outcomes

An electronic case report form (e-CRF) or a paper case report form (depending on patient preference) will be used to collect patient reported study data. The e-CRF and CRF will be developed by the study data centre at the Robertson Centre for Biostatistics, University of Glasgow and access to the e-CRF will be restricted, with only authorised site-specific personnel able to make entries or amendments to their patients' data. All data handling procedures will be detailed in a study specific Data Management Plan.

7.1.5 Routine clinical information

A number of features have been built into the SCOTS system which will be available for surgeons to use during routine follow up visits. Each time a patient attends, their weight will be recorded. Notes can be included and exported for inclusion within patient notes, provided for patients or within letters to GPs.

7.1.6 Re-operation

An electronic case report form (e-CRF) will be used to collect data on re-operations. On or shortly after the patient undergoes weight loss surgery a member of the clinical team will record specific information on the patients repeat weight loss operation. Not only will the details of the operation be recorded but the reason for operation will be included within the e-CRF.

For peer review only - http://bmjopen.bmj.com/site/about/guidelines.xhtml

7.1.7 Record Retention

To enable evaluations and/or audits from regulatory authorities, the investigator agrees to keep records, including the identity of all participating subjects (sufficient information to link records), all original signed informed consent forms, source documents, and detailed records of treatment disposition in accordance with ICH GCP, local regulations, or as specified in the Clinical Study Agreement, whichever is longer. Data will be retained at the Robertson Centre once the recruitment phase of the study is complete. During recruitment a copy of the patient consent form and the patient registration will be retained within the site file and within the patient notes.

7.1.8 Access to data

Clinical teams will be able to access their own data. All patients who have been entered within the SCOTS system will be viewed by the clinical teams who entered data. Data which is routinely collected for the National Bariatric Surgery Registry (NBSR) can be entered within the system for surgeons who wish to contribute to this process. Surgeons fully anonymised data (no patient identifiable information) can be exported to a file which surgeons can share with the NBSR if they surgeons wish to contribute. The management and approvals for this will be responsibility of the investigating surgeon and data will not be shared by the SCOTS team.

For peer review only

For peer review only - http://bmjopen.bmj.com/site/about/guidelines.xhtml

8 SCOTS STUDY MANAGEMENT

8.1 Study Sponsor

NHS Greater Glasgow and Clyde Research and Development Office as study sponsors are responsible for the management and conduct (according to GCP). The SCOTS steering committee (SSC) oversee the management and conduct of the study and report to and advise the sponsor on the study. All minutes from both the SCOTS management group (SMG) and SCOTS steering committee (SSC) are copied to NHS Greater Glasgow and Clyde Research and Development Office

8.2 SCOTS Study Team

A study team comprising of the chief investigator, a research lead and a project manager, in collaboration with the data management team based at the Robertson Centre will run the project on a day to day basis. Issues arising from patients or investigators will be managed by this team and those that cannot be resolved by that team will be discussed with the chief Investigator and escalated to the study management group for discussion.

8.3 SCOTS Principal investigators

Each site will have an identified principal investigator who will oversee the application of this protocol locally. Each member of the clinical team involved in the study will be required to register for a login and password to access the e-CRF through the SCOTS data collection portal. Any member of the local clinical teams who has registered username and password and entered data to the SCOTS data collection portal will be a member of the SCOTS Investigators Group. The investigators will be required to enter some initial registration data to link themselves with the surgeons they work with. Planned publications outlined in Table 15-1~~Table 15-1~~ will be written on behalf of the SCOTS Investigators Group.

8.4 Routine management of study: SCOTS Management Group (SMG)

The trial will be coordinated from the University of Glasgow by the SCOTS management group (SMG). The SMG will consist of the principal investigator, other co-applicants, project manager and representatives from the Clinical Trials Unit. The role of the group is to monitor all aspects of the conduct and progress of the trial, ensure that the protocol is adhered to and take appropriate action to safeguard participants and the quality of the trial itself. It will be responsible for the day to day running of the trial and budget. It will meet at least 6 monthly, with more meetings initially and as required, and provide information to the steering committee as to the progress of the study.

For peer review only - http://bmjopen.bmj.com/site/about/guidelines.xhtml

8.5 SCOTS Steering Committee (SSC)

SCOTS Steering Committee (SSC) will be established to provide overall supervision of the study and ensure adherence to GCP. It will have an independent chair, a patient representative, and at least two other independent academics along with the principal investigator. Observers from the HTA programme will be invited to SSC meetings, and all SSC papers will be supplied to the HTA programme. The SSC will meet at the start of the study, and annually thereafter.

The role of the SSC is to provide overall supervision of the trial and ensure that it is being conducted in accordance with the principles of GCP and the relevant regulations. The SSC will:

- agree the trial protocol and any protocol amendments
- provide advice to the investigators on all aspects of the trial
- include members who are independent of the investigators, in particular an independent chairperson.

Decisions about continuation or termination of the trial or substantial amendments to the protocol are usually the responsibility of the SSC who advise the sponsor.

For peer review only

For peer review only - http://bmjopen.bmj.com/site/about/guidelines.xhtml

9 STUDY MONITORING/AUDITING

This study is a cohort study. It will therefore be audited on random sampling basis by GG&C auditors on behalf of the GG&C sponsor who audit 10% of sponsored studies per annum.

10 PROTOCOL AMENDMENTS

Any change in the study protocol will require an amendment. Any proposed protocol amendments will be initiated by the CI following discussion with the SSC and any required amendment forms will be submitted to the regulatory authority, ethics committee and sponsor. The CI and the SSC will liaise with study sponsor to determine whether an amendment is non-substantial or substantial. All amended versions of the protocol will be signed by the CI and sponsor representative. Before the amended protocol can be implemented favourable opinion/approval must be sought from the original reviewing Research and Ethics Committee (REC) and Research and Development (R&D) office(s).

11 ETHICAL CONSIDERATIONS

11.1 Ethical conduct of the study

The study will be carried out in accordance with the World Medical Association Declaration of Helsinki (1964) and its revisions (Tokyo [1975], Venice [1983], Hong Kong [1989], South Africa [1996] and Edinburgh [2000]).

Favourable ethical opinion will be sought from an appropriate REC before patients are entered into this study. Patients will only be allowed to enter the study once they have provided written informed consent.

The CI will be responsible for updating the REC of any new information related to the study.

11.2 Informed consent

Patients will be informed of the SCOTS study as they discuss weight loss surgery with their clinical teams. They will be provided with an information sheet on the study by a trained member of the clinical teams during a routine clinic visit or during a group session depending on the local patient pathway. The patient will be given time to consider their participation in the study, review the SCOTS information sheet and ask about any concerns they may have. Patients will then be asked to sign the SCOTS consent form.

For peer review only - http://bmjopen.bmj.com/site/about/guidelines.xhtml

12 INSURANCE AND INDEMNITY

SCOTS is sponsored by NHS Greater Glasgow & Clyde and NHS indemnity is provided under the Clinical Negligence and Other Risks Indemnity Scheme (CNORIS). In addition, as the protocol authors are employees of the University of Glasgow negligent harm caused by the design of the trial will be covered by the Clinical Trials Insurance policy held by the University of Glasgow.

Investigators working in private healthcare will recruit to this NHS funded study as part of their NHS role and will therefore be covered as detailed above.

13 FUNDING

Funding source: National Institute for Health Research (NIHR) Health Technology Assessment Programme

Grant Award number: 10/42/02

Original Cost: £2,124,030

Amended Cost: £1,516,255.60

URL of this project on the NIHR Website: http://www.hta.ac.uk/project/2608.asp

Start Date: January 2012

End date: June 2021.

Estimated date of publication in HTA journal series: Late 2021. This date takes account of time for report preparation and printing based on current average times for these activities.

International Standard Randomised Controlled Trial Number: ISRCTN 47072588

URL of this project on the Controlled Trials Website: http://www.controlled-trials.com/ISRCTN47072588

14 ANNUAL REPORTS

A bi-annual progress report will be submitted to NIHR (the funder), the first being submitted 6 months from the date that all funding started (January 2012).

Annual reports will be submitted to the ethics committee and sponsor with the first submitted one year after the date that all study related approvals are in place. These reports will follow the template on the IRAS web site.

For peer review only - http://bmjopen.bmj.com/site/about/guidelines.xhtml

15 DISSEMINATION OF FINDINGS

Study results whether, negative or positive, will be disseminated. Details of the plan for dissemination are detailed below. Some of the outcomes described may be reported in a number of peer reviewed journal articles. The SCOTS Publication and Presentation SOP (18) describes how this will be managed throughout the study and will ensure that NIHR guidance for publications is adhered to.

TABLE 15-1: SCOTS PUBLICATION PLANS

	Date	Description of publication
1	**Jan 2015**	Methods paper: Outlining the methods used in the study including the IT based nutritional blood monitoring and the dataset being collected. Published – Logue et al BMJ Open 2015;5:e008106
2	**Jul 2017**	Health and socio-economic burden of severe obesity in a population undergoing bariatric surgery.
3	**Jul 2019**	The effect of the pre and post-surgery pathway and surgical experience on weight and complications outcomes after bariatric surgery.
4	**Jun 2021**	Change in health and socioeconomic factors 3 years after bariatric surgery.<br>Full HTA report within the NIHR HTA Journal Series.

For peer review only - http://bmjopen.bmj.com/site/about/guidelines.xhtml

16 REFERENCES

(1) Picot J, Jones J, Colquitt JL, Gospodarevskaya E, Loveman E, Baxter L, et al. The clinical effectiveness and cost-effectiveness of bariatric (weight loss) surgery for obesity: a systematic review and economic evaluation. Health Technol Assess 2009 Sep;13(41):1-357, iii.

(2) O'Brien PE, Sawyer SM, Laurie C, Brown WA, Skinner S, Veit F, et al. Laparoscopic adjustable gastric banding in severely obese adolescents: a randomized trial. JAMA 2010 Feb 10;303(6):519-26.

(3) Adams TD, Gress RE, Smith SC, Halverson RC, Simper SC, Rosamond WD, et al. Long-term mortality after gastric bypass surgery. N Engl J Med 2007 Aug 23;357(8):753-61.

(4) Sjostrom L, Narbro K, Sjostrom CD, Karason K, Larsson B, Wedel H, et al. Effects of bariatric surgery on mortality in Swedish obese subjects. N Engl J Med 2007 Aug 23;357(8):741-52.

(5) Dixon JB, O'Brien PE, Playfair J, Chapman L, Schachter LM, Skinner S, et al. Adjustable gastric banding and conventional therapy for type 2 diabetes: a randomized controlled trial. JAMA 2008 Jan 23;299(3):316-23.

(6) Buchwald H, Estok R, Fahrbach K, Banel D, Jensen MD, Pories WJ, et al. Weight and type 2 diabetes after bariatric surgery: systematic review and meta-analysis. Am J Med 2009 Mar;122(3):248-56.

(7) Sjostrom L, Lindroos AK, Peltonen M, Torgerson J, Bouchard C, Carlsson B, et al. Lifestyle, diabetes, and cardiovascular risk factors 10 years after bariatric surgery. N Engl J Med 2004 Dec 23;351(26):2683-93.

(8) Sjostrom L, Lindroos AK, Peltonen M, Torgerson J, Bouchard C, Carlsson B, et al. Lifestyle, diabetes, and cardiovascular risk factors 10 years after bariatric surgery. N Engl J Med 2004 Dec 23;351(26):2683-93.

(9) Mathus-Vliegen EM, de Wit LT. Health-related quality of life after gastric banding. Br J Surg 2007 Apr;94(4):457-65.

(10) Karlsson J, Taft C, Ryden A, Sjostrom L, Sullivan M. Ten-year trends in health-related quality of life after surgical and conventional treatment for severe obesity: the SOS intervention study. Int J Obes (Lond) 2007 Aug;31(8):1248-61.

(11) Ford I, Murray H, Packard CJ, Shepherd J, Macfarlane PW, Cobbe SM. Long-term follow-up of the West of Scotland Coronary Prevention Study. N Engl J Med 2007 Oct 11;357(15):1477-86.

(12) Research Governance Framework for Health and Community Care (Second edition). Scottish Executive Health Department; 2006.

For peer review only - http://bmjopen.bmj.com/site/about/guidelines.xhtml

(13) NHS National Services Scotland. ISD Statistical Disclosure Control Protocol. Edinburgh: ISD Scotland Publications; 2010.

(14) Scottish Intercollegiate Guideline Network. Management of Obesity 115 - A national clincal guideline. Edinburgh: Scottish Intercollegiate Guidelines Network; 2010 Feb 1. Report No.: 115.

(15) Knox H, Davies S. Obestity Treatment Best Practice Guidance. Edinburgh: SGHD Quality Unit, Planning and Quality Division; 12 A.D. Jul 1.

(16) Mid-2011 Population Estimates Scotland Population estimates by sex, age and administrative area.

National Statistics publication for Scotland; 2012 May 31.

(17) Chevallier JM, Paita M, Rodde-Dunet MH, Marty M, Nogues F, Slim K, et al. Predictive factors of outcome after gastric banding: a nationwide survey on the role of center activity and patients' behavior. Ann Surg 2007 Dec;246(6):1034-9.

(18) SCOTS Management Team. Surgical Obesity Treatment Study (SCOTS) Publication and Presentation Group Operating Procedures. 2012.

(19) Scottish Government. Scottish Health Survey 2010 - Volume 2: Technical Report. 2008.

(20) General Register Office. Scottish Census 2011 Household Questionnaire. General Register Office, editor. GRO . 2011.

Ref Type: Online Source

(21) Bruce J, Thornton AJ, Scott NW, Marfizo S, Powell R, Johnston M, et al. Chronic preoperative pain and psychological robustness predict acute postoperative pain outcomes after surgery for breast cancer. Br J Cancer 2012 Sep 4;107(6):937-46.

(22) Mercer SW, Watt GC. The inverse care law: clinical primary care encounters in deprived and affluent areas of Scotland. Ann Fam Med 2007 Nov;5(6):503-10.

(23) Wadden TA, Foster GD. Weight and Lifestyle Inventory (WALI). Obesity (Silver Spring) 2006 Mar;14 Suppl 2:99S-118S.

(24) Diabetes UK. Diabetes Risk Score. 2012. Diabetes UK.

Ref Type: Online Source

(25) Ware J, Jr., Kosinski M, Keller SD. A 12-Item Short-Form Health Survey: construction of scales and preliminary tests of reliability and validity. Med Care 1996 Mar;34(3):220-33.

(26) The EuroQol Group. EuroQol-a new facility for the measurement of health-related quality of life. Health Policy 1990;16(3):199-208.

For peer review only - http://bmjopen.bmj.com/site/about/guidelines.xhtml

(27) Spitzer RL, Kroenke K, Williams JB. Validation and utility of a self-report version of PRIME-MD: the PHQ primary care study. Primary Care Evaluation of Mental Disorders. Patient Health Questionnaire. JAMA 1999 Nov 10;282(18):1737-44.

(28) Cameron IM, Crawford JR, Lawton K, Reid IC. Psychometric comparison of PHQ-9 and HADS for measuring depression severity in primary care. Br J Gen Pract 2008 Jan;58(546):32-6.

(29) Spitzer RL, Kroenke K, Williams JB, Lowe B. A brief measure for assessing generalized anxiety disorder: the GAD-7. Arch Intern Med 2006 May 22;166(10):1092-7.

(30) Kolotkin RL, Crosby RD. Psychometric evaluation of the impact of weight on quality of life-lite questionnaire (IWQOL-lite) in a community sample. Qual Life Res 2002 Mar;11(2):157-71.

(31) Kolotkin RL, Crosby RD, Pendleton R, Strong M, Gress RE, Adams T. Health-related quality of life in patients seeking gastric bypass surgery vs non-treatment-seeking controls. Obes Surg 2003 Jun;13(3):371-7.

(32) Gosman GG, King WC, Schrope B, Steffen KJ, Strain GW, Courcoulas AP, et al. Reproductive health of women electing bariatric surgery. Fertil Steril 2010 Sep;94(4):1426-31.

(33) Derby CA, Araujo AB, Johannes CB, Feldman HA, McKinlay JB. Measurement of erectile dysfunction in population-based studies: the use of a single question self-assessment in the Massachusetts Male Aging Study. Int J Impot Res 2000 Aug;12(4):197-204.

(34) Barry MJ, Fowler FJ, Jr., O'Leary MP, Bruskewitz RC, Holtgrewe HL, Mebust WK, et al. The American Urological Association symptom index for benign prostatic hyperplasia. The Measurement Committee of the American Urological Association. J Urol 1992 Nov;148(5):1549-57.

(35) Abrams P, Avery K, Gardener N, Donovan J. The International Consultation on Incontinence Modular Questionnaire: www.iciq.net. J Urol 2006 Mar;175(3 Pt 1):1063-6.

(36) Macran S, Wileman S, Barton G, Russell I. The development of a new measure of quality of life in the management of gastro-oesophageal reflux disease: the Reflux questionnaire. Qual Life Res 2007 Mar;16(2):331-43.

(37) Grant A, Wileman S, Ramsay C, Bojke L, Epstein D, Sculpher M, et al. The effectiveness and cost-effectiveness of minimal access surgery amongst people with gastro-oesophageal reflux disease - a UK collaborative study. The REFLUX trial. Health Technol Assess 2008 Sep;12(31):1-iv.

(38) Helander A, Lunner K, Leifman A, Borg S. [Alcohol-related problems in connection with overweight surgery]. Lakartidningen 2011 May 4;108(18):964-5.

For peer review only - http://bmjopen.bmj.com/site/about/guidelines.xhtml

(39) Babor TF, Higgins-Biddle JC, Saunders JB, Monteiro MG. The Alcohol Use Disorders Identification Test. World Health Organisation Department of Mental Health and Substance Dependence; 2001 Jan 1. Report No.: WHO/MSD/MSB/01.6a.

(40) Craig CL, Marshall AL, Sjostrom M, Bauman AE, Booth ML, Ainsworth BE, et al. International physical activity questionnaire: 12-country reliability and validity. Med Sci Sports Exerc 2003 Aug;35(8):1381-95.

(41) Roe L, Strong C, Whiteside C, Neil A, Mant D. Dietary intervention in primary care: validity of the DINE method for diet assessment. Fam Pract 1994 Dec;11(4):375-81.

(42) Scheier MF, Carver CS, Bridges MW. Distinguishing optimism from neuroticism (and trait anxiety, self-mastery, and self-esteem): a reevaluation of the Life Orientation Test. J Pers Soc Psychol 1994 Dec;67(6):1063-78.

(43) Foster GD, Wadden TA, Vogt RA, Brewer G. What is a reasonable weight loss? Patients' expectations and evaluations of obesity treatment outcomes. J Consult Clin Psychol 1997 Feb;65(1):79-85.

(44) Health Protection Scotland. The epidemiology of orthopaedic surgical site infection occuring up to one year after surgery: a feasibility study of telephone screening and direct observation by trained healthcare workers. Health Protection Scotland; 2010.

(45) Horan TC, Andrus M, Dudeck MA. CDC/NHSN surveillance definition of health care-associated infection and criteria for specific types of infections in the acute care setting. Am J Infect Control 2008 Jun;36(5):309-32.

(46) Ertlet TW, Marino JM, Mitchell JE. Psychosocial aspects of body contourning surgery after bariatric surgery. In: Shiffman MA, Di Giuseppe A, editors. Body Contouring.Berlin: Springer-Verlag; 2010. p. 633-9.

(47) Keating CL, Moodie ML, Bulfone L, Swinburn BA, Stevenson CE, Peeters A. Healthcare Utilization and Costs in Severely Obese Subjects Before Bariatric Surgery. Obesity (Silver Spring) 2012 May 4.

(48) (DIRUM) Database of Instruments for Resource Use Measurement. 2012. Bangor, Bangor University.

Ref Type: Online Source

(49) Petrou S. WoLFF (Wound dressings in Lower Limb Fracture) Questionnaire. 2012. 1-8-2012.

(50) EPIC Norfolk Study. Food Frequency Questionnaire. Cambridge: University of Cambridge; 2012. Report No.: (CAMB/PQ/6/1205).

For peer review only - http://bmjopen.bmj.com/site/about/guidelines.xhtml

17 Appendix E: Summary of Patient Reported Outcome Questions

Table 17-1~~Table 17-1~~ shows a summary table of each set of questions which will be asked of the patient, when they will be asked and if they are validated or not validated.

Questions are classed as not validated in the form they are being asked if they have not been used in a published study and are identified by either a 0 or P in the validated column. 0 means that the set of questions are not validated P means that the questions are partially validated. Partially validated question sets are where a previously published question set has had additional questions added in order to fulfil the SCOTS objectives.

TABLE 17-1: SUMMARY OF SCOTS PATIENT REPORTED OUTCOME MEASURES.

For peer review only - http://bmjopen.bmj.com/site/about/guidelines.xhtml

Glasgow Clinical Trials Unit

PROMS	Total	Total Male	Total Femal	Validated	baseline	2 year	3 year	Total
About you								
Demographics – Essential	1	1	1	P	1	1	1	3
Marital Status	1	1	1	1	1	1	1	3
Ethnicity	1	1	1	1	1	0	0	1
Education	1	1	1	1	1	0	0	1
Occupation	1	1	1	P	1	1	1	3
Health History								
Your Medical History	1	1	1	1	1	0	0	1
Your Weight History	1	1	1	1	1	0	0	1
Family History	1	1	1	P	1	0	0	1
Family History of Obesity	1	1	1	0	1	0	0	1
Quality of Life								
SF-12 v2 –licence required	1	1	1	1	1	1	1	3
EQ5D 5L	1	1	1	1	1	1	1	3
Patient Health Questionnaire (PHQ-9) (No permissions Free)	1	1	1	1	1	1	1	3
Generalized Anxiety Disorder GAD-7 (No permissions Free)	1	1	1	1	1	1	1	3
Impact of Weight								
Impact of Weight on Quality of Life (IWQOL-Lite) – Licence required	1	1	1	1	1	1	1	3
Reproductive Health – Females only	1	0	1	1	1	1	1	3
Massachusetts Male Aging Study: Single Question Assessment Males only	1	1	0	1	1	1	1	3
International Prostate Symptom Score (IPSS) Males only	1	1	0	1	1	1	1	3
Continence	1	1	1	1	1	1	1	3
Reflux Questionnaire	1	1	1	1	1	1	1	3
Lifestyle								
Smoking	1	1	1	1	1	1	1	3
The Alcohol Use Disorders Identification Test: Self-Report Version	1	1	1	1	1	1	1	3
Physical Activity IPAC short form self administered	1	1	1	1	1	1	1	3
Nutrition	1	1	1	1	1	1	1	3
Personal Outlook								
Life Orientation Test (REVISED)	1	1	1	1	1	1	0	2
Expectations of Surgery	1	1	1	P	1	0	0	1
Surgical Recovery								
Post operative pain (1 month acute, 6 month-1 year chronic)	1	1	1	0	0	0	0	0
Surgical site infection	1	1	1	1	0	0	0	0
Skin Excess	1	1	1	0	0	0	0	0
Plastic surgery	1	1	1	P	0	0	0	0
Healthcare and Support								
Outpatient care	1	1	1	1	1	1	1	3
Contacts with other health and social care professionals	1	1	1	1	1	1	1	3
Devices and specialist equipment	1	1	1	1	1	1	1	3
Benefits	1	1	1	1	1	1	1	3
Multivitamins and Supplements use	1	1	1	1	1	1	1	3
	34	33	32	**26**	**30**	**23**	**22**	**75**

For peer review only - http://bmjopen.bmj.com/site/about/guidelines.xhtml

This page is intentionally blank

For peer review only

For peer review only - http://bmjopen.bmj.com/site/about/guidelines.xhtml

Medical history questions included in baseline questionnaires/patient reported outcome measures (PROMs).

Why we are asking you these questions?

It is important to know what illness patients have before their surgery so we can look at how much overall health has changed after their weight loss surgery.

Medical History

1. Do you have a history of any of the following? – tick all that apply

☐ Deep vein thrombosis	(Deep vein thrombosis (DVT) is a blood clot in one of the deep veins in the leg.)
☐ Pulmonary embolism	(A pulmonary embolism is a blood clot in the pulmonary artery which is the blood vessel that transports blood from the heart to the lungs.)
☐ High blood pressure	
☐ Diabetes	
☐ Angina/heart attack	
☐ Heart Failure	
☐ Stroke/mini-stroke	
☐ Arthritis	
☐ Back problems	
☐ Chronic Bronchitis	
☐ Eczema/psoriasis	
☐ Asthma	
☐ Thyroid problems	
☐ Migraine	
☐ Anxiety/depression	
☐ Kidney disease	
☐ Liver disease	
☐ Cancer	
☐ Irritable bowel syndrome	
☐ Doctor diagnosed sleep apnoea	(Obstructive sleep apnoea (OSA) is a condition that causes interrupted breathing during sleep and requires a machine to keep airways open while you sleep. Only check this box if your doctor has diagnosed sleep apnoea.)
☐ Other (please specify _______________)	

For peer review only

SCOTS

Smoking

Patient Questionnaire

Draft 01

Lifestyle

This section asks about your smoking, drinking, eating and exercising habits.

Why are we asking you these questions

Smoking habits may alter after weight loss surgery.

Smoking

1. Smoking history

Current ☐ 1

Former ☐ 2

Never ☐ 3

If Current,

1a. How many per day? |_|_|_|

If former,

1a. Year stopped? |_|_|_|_| Y Y Y Y

For peer review only

SCOTS

Work

Patient Questionnaire

Draft 02

Why are we asking you these questions

The ability to work or go about usual activity is an important part of a patient's overall health and quality of life. We are interested to see if there is a change in your occupation before and after your weight loss surgery.

Occupation

1. Please select a category that best describes your current employment status

Working full time (30hrs or more per week)	☐1	please go to section A
Working part time (less than 30hrs or more per week)	☐2	please go to section A
Unable to work because of illness or disability	☐3	please go to section B
Student	☐4	please go to section A
Unemployed and looking for work	☐5	please go to section B
Carer for children or relative	☐6	please go to section B
At home and not looking for paid employment	☐6	please go to section B
Other	☐6	please go to section B

For peer review only

Section A

1. What is your current occupation? Since many people have more than one job at a given time, we would like to know about the job that is your primary source of income

____

2. Have you had any days off sick in the last month? Yes ☐1 No ☐2 Not applicable ☐3

   a. If Yes, how many days in total (please only include the number of days you were not able to work)? |_|_|_|

3. Are you receiving benefits? Yes ☐1 No ☐2

Section B

1. Are you receiving benefits? Yes ☐1 No ☐2

Produced by Robertson Centre for Biostatistics, University of Glasgow

Social security (benefits) questions included in baseline questionnaires/patient reported outcome measures (PROMs).

Why we are asking you these questions?

Weight loss surgery can be expensive, but often the improvements in patients' health are great. This is to be used in health economic analysis which balances the cost of weight loss surgery with the improvements in patients' health and wellbeing and overall cost of bariatric surgery.

Due to the personal nature of these questions, you may choose not to complete them.

I am happy to complete these questions

- ☐ Yes
- ☐ No

If No, please go to the next section.

Benefits

1. In the past three months, you may have received some benefits from the government to support you. In the table below, please tick all benefits you have received in the past 3 months.

- ☐ Attendance Allowance
- ☐ Carer's Allowance
- ☐ Child Tax Credit
- ☐ Council Tax Benefit
- ☐ Disability Living Allowance—caring
- ☐ Disability Living Allowance—mobility
- ☐ Employment and Support Allowance
- ☐ Housing Benefit
- ☐ Income Support
- ☐ Jobseeker's Allowance
- ☐ Pension Credit
- ☐ Statutory Sick Pay
- ☐ State Pension
- ☐ Other (please specify _______________)
- ☐ None

For peer review only

Healthcare utilisation (devices and specialist equipment) questions included in baseline questionnaires/patient reported outcome measures (PROMs).

Why we are asking you these questions?

Some people may require specialist equipment or devices as a result of their weight and changes in requirements for equipment will be considered in the overall cost of weight loss surgery.

<u>Devices and Specialist Equipment</u>

1	Do you currently use any aids or specialist equipment?	☐ Yes<br>☐ No<br>If No, move to next section	
1a	Stair lift	☐ Yes<br>☐ No	If Yes, funded by:<br>☐ provided by Social Services<br>☐ provided by hospital<br>☐ bought by you
1b.	Grab rail	☐ Yes<br>☐ No	If Yes, funded by:<br>☐ provided by Social Services<br>☐ provided by hospital<br>☐ bought by you
1c.	Bariatric bed	☐ Yes<br>☐ No	If Yes, funded by:<br>☐ provided by Social Services<br>☐ provided by hospital<br>☐ bought by you
1d.	Bariatric shower stool	☐ Yes<br>☐ No	If Yes, funded by:<br>☐ provided by Social Services<br>☐ provided by hospital<br>☐ bought by you
1e.	Bariatric chair	☐ Yes<br>☐ No	If Yes, funded by:<br>☐ provided by Social Services<br>☐ provided by hospital<br>☐ bought by you
1f.	Wheel chair (bariatric)	☐ Yes<br>☐ No	If Yes, funded by:<br>☐ provided by Social Services<br>☐ provided by hospital<br>☐ bought by you
1g.	Mobility scooter	☐ Yes<br>☐ No	If Yes, funded by:<br>☐ provided by Social Services<br>☐ provided by hospital<br>☐ bought by you
1h.	Dressing aids	☐ Yes<br>☐ No	If Yes, funded by:<br>☐ provided by Social Services<br>☐ provided by hospital<br>☐ bought by you
1i.	Other	☐ Yes<br>☐ No	If Yes, please describe any other devices or specialist equipment you use.<br>________________<br>________________<br>Funded by:<br>☐ provided by Social Services<br>☐ provided by hospital<br>☐ bought by you

For peer review only

Supplementary Table 2. Surgical Obesity Treatment Study (SCOTS) baseline data by body mass index (BMI) group.

			BMI (kg/m^2)				
			Group 1	Group 2	Group 3	Group 4	Group 5
		Total Number of Participants Completing Question (N missing)	< 40<br>N=24	40-44<br>N=64	45-49<br>N=64	50-54<br>N=44	55+<br>N=53
**Demographics**	Sex: % Male	249 (0)	33.3%	23.4%	31.3%	38.6%	22.6%
	SIMD: % Q1 (Most Deprived)	247 (2)	20.8%	20.3%	25.0%	30.2%	44.2%
**Medical history**	% DVT	249 (0)	8.3%	1.6%	3.1%	4.5%	1.9%
	% Hypertension	249 (0)	45.8%	32.8%	51.6%	45.5%	41.5%
	% Type 2 Diabetes	249 (0)	45.8%	39.1%	60.9%	61.4%	41.5%
	% CVD	249 (0)	16.7%	9.4%	6.3%	6.8%	5.7%
	% Arthritis	249 (0)	16.7%	23.4%	35.9%	22.7%	39.6%
	% Back Problems	249 (0)	41.7%	45.3%	50.0%	38.6%	50.9%
	% Asthma	249 (0)	0.0%	25.0%	29.7%	27.3%	43.4%
	% Migraine	249 (0)	20.8%	10.9%	17.2%	25.0%	28.3%
	% Irritable Bowel Syndrome	249 (0)	16.7%	18.8%	20.3%	18.2%	13.2%
	% Sleep Apnoea	249 (0)	16.7%	20.3%	28.1%	27.3%	35.8%
	% PCOS	167 (10)	46.7%	8.3%	17.1%	20.0%	13.2%
	% Gastroesophageal Reflux	240 (9)	26.1%	38.1%	52.5%	39.5%	36.0%
**Incontinence**	% ICIQ-UI SF Score ≥6	239 (10)	26.1%	42.2%	46.7%	40.5%	54.0%
	% ICIQ-UI SF Score ≥6 (females only)	168 (9)	40.0%	42.9%	50.0%	56.0%	56.4%
	% ICIQ-UI SF Score ≥6 (males only)	71 (1)	0.0%	40.0%	40.0%	17.6%	45.5%
	Median (LQ, UQ) ICIQ-UI SF Score	239 (10)	0.0 (0.0; 6.0)	4.0 (0.0; 9.5)	5.0 (0.0; 8.0)	3.0 (0.0; 12.0)	9.0 (3.0; 13.0)
**Depression**	Mean (SD) PHQ-9 Score	244 (5)	7.4 (6.6)	9.5 (6.4)	9.0 (6.1)	10.9 (6.5)	10.5 (5.9)
	% PHQ-9 Score ≥10	244 (5)	26.1%	46.9%	38.1%	48.8%	51.0%
**Anxiety**	Median (LQ, UQ) GAD-7 Score	243 (6)	4.0 (1.0; 6.0)	5.0 (2.0; 10.5)	5.5 (2.0; 9.0)	4.0 (2.0; 12.0)	7.0 (3.0; 10.0)
	% GAD-7 Score ≥6	243 (6)	30.4%	42.2%	50.0%	39.5%	62.7%
**Smoking Status**	% Current	240 (9)	17.4%	4.8%	4.8%	2.3%	4.0%
	% Former		30.4%	45.2%	46.8%	39.5%	48.0%
	% Never		52.2%	50.0%	48.4%	58.1%	48.0%
**Quality of Life**							
SF-12	Mean (SD) SF-12 PCS	236 (13)	43.5 (11.3)	40.3 (11.0)	37.6 (10.6)	35.3 (11.6)	31.1 (10.3)
	Mean (SD) SF-12 MCS	236 (13)	48.7 (7.7)	44.8 (9.1)	45.7 (11.9)	43.8 (10.8)	46.1 (10.2)
EQ-5D-5L	Median (LQ, UQ) EQ-5D-5L Score	237 (12)	0.8 (0.7; 0.9)	0.7 (0.3; 0.8)	0.6 (0.4; 0.8)	0.6 (0.4; 0.7)	0.5 (0.1; 0.6)
	Mean (SD) EQ-5D-5L VAS	237 (12)	64.9 (22.6)	58.8 (22.3)	56.5 (22.5)	51.7 (18.9)	47.9 (21.8)
IWQOL-Lite (Standardised Scoring)	Mean (SD) Physical Function	243 (6)	35 (24.9)	50.5 (23.3)	54.2 (24.5)	61.1 (21.6)	74.8 (20.6)
	Median (LQ, UQ) Self Esteem	242 (7)	60.7 (42.9; 85.7)	71.4 (53.6; 89.3)	71.4 (39.3; 92.9)	89.3 (71.4; 96.4)	85.7 (71.4; 96.4)
	Mean (SD) Sexual Life	231 (18)	40.8 (33.4)	58.8 (30.0)	54.2 (31.2)	60.4 (34.7)	63.7 (28.8)
	Mean (SD) Public Distress	243 (6)	28.9 (25.7)	48.1 (22.4)	55.9 (25.7)	70.1 (24.1)	76.1 (19.8)
	Mean (SD) Work	236 (13)	31 (30.1)	38.9 (29.3)	40 (29.2)	47.5 (25.1)	55.5 (28.6)
	Mean (SD) Total Score	242 (7)	40.5 (22.5)	53.7 (18.9)	55.3 (22.0)	64.1 (19.0)	71.8 (17.5)

For peer review only

**Healthcare Utilisations**	% Use of Any Aids or Specialist Equipment	232 (17)	9.1%	21.3%	27.9%	25.6%	51.0%
**Social Security**	% Unable to Work Due to Illness or Disability	248 (1)	25.0%	23.4%	23.4%	23.3%	34.0%
	% Disability Living Allowance - Caring	236 (13)	17.4%	17.7%	21.3%	17.5%	18.0%
	% Disability Living Allowance - Mobility	236 (13)	17.4%	19.4%	23.0%	17.5%	20.0%

ICIQ-UI SF score: ≥6 = 'moderate incontinence' [1]
PHQ-9 scores: 0-4 = 'minimal depression'; 5-9 = 'mild depression'; 10-14 = 'moderate depression'; 15-19 = 'moderately severe depression'; 20-27 = 'severe depression' [2]
GAD-7 scores: 0-5 = 'mild anxiety'; 6-10 = 'moderate anxiety'; 11-15 = 'moderately severe anxiety'; 15-21 = 'severe anxiety' [3]

References

1. Avery K, Donovan J, Peters TJ, Shaw C, Gotoh M, Abrams P. ICIQ: A brief and robust measure for evaluating the symptoms and impact of urinary incontinence. *Neurourol Urodyn* 2004; **23**(4)**:** 322–330

2. Spitzer RL, Kroenke K, Williams JBW. Validation and utility of a self-report version of PRIME-MD: The PHQ Primary Care Study. *J Am Med Assoc* 1999; **282**(18): 1737-1744

3. Spitzer RL, Kroenke K, Williams JBW, Löwe B. A brief measure for assessing generalized anxiety disorder: The GAD-7. *Arch Intern Med* 2006; **166**(10): 1092-1097

For peer review only

Supplementary Table 3. Surgical Obesity Treatment Study (SCOTS) baseline data by age group.

			Age (years)				
			Group 1	Group 2	Group 3	Group 4	Group 5
		Total Number of Participants Completing Question (N missing)	< 35 N=36	35-44 N=63	45-49 N=63	50-54 N=43	55+ N=44
**Demographics**	Sex: % Male	249 (0)	22.2%	22.2%	28.6%	30.2%	43.2%
	SIMD: % Q1 (Most Deprived)	247 (2)	40.0%	28.6%	14.5%	39.5%	27.3%
**Medical history**	% DVT	249 (0)	2.8%	3.2%	3.2%	0.0%	6.8%
	% Hypertension	249 (0)	11.1%	36.5%	52.4%	44.2%	63.6%
	% Type 2 Diabetes	249 (0)	30.6%	50.8%	54.0%	44.2%	63.6%
	% CVD	249 (0)	0.0%	6.3%	6.3%	9.3%	18.2%
	% Arthritis	249 (0)	5.6%	15.9%	36.5%	27.9%	59.1%
	% Back Problems	249 (0)	47.2%	50.8%	31.7%	39.5%	65.9%
	% Asthma	249 (0)	30.6%	22.2%	31.7%	20.9%	36.4%
	% Migraine	249 (0)	27.8%	17.5%	20.6%	18.6%	15.9%
	% Irritable Bowel Syndrome	249 (0)	8.3%	27.0%	23.8%	4.7%	15.9%
	% Sleep Apnoea	249 (0)	5.6%	20.6%	28.6%	25.6%	50.0%
	% PCOS	167 (10)	26.9%	26.1%	9.3%	7.1%	12.5%
	% Gastroesophageal Reflux	240 (9)	50.0%	50.0%	36.1%	28.6%	37.2%
**Incontinence**	% ICIQ-UI Score ≥6	239 (10)	18.2%	44.3%	50.8%	50.0%	47.6%
	% ICIQ-UI Score ≥6 (females only)	168 (9)	20.0%	51.1%	55.8%	62.1%	50.0%
	% ICIQ-UI Score ≥6 (males only)	71 (1)	12.5%	21.4%	38.9%	23.1%	44.4%
	Median (LQ, UQ) ICIQ-UI Score	239 (10)	0.0 (0.0; 4.0)	5.0 (0.0; 11.0)	6.0 (0.0; 11.0)	5.5 (3.0; 11.0)	4.5 (0.0; 11.0)
**Depression**	Mean (SD) PHQ-9 Score	244 (5)	9.0 (6.2)	10.3 (6.3)	9.2 (5.9)	9.1 (7.0)	10.2 (6.2)
	% PHQ-9 Score ≥10	244 (5)	42.9%	50.0%	37.7%	37.2%	51.2%
**Anxiety**	Median (LQ, UQ) GAD-7 Score	243 (6)	5.0 (2.0; 10.0)	6.0 (3.0; 12.0)	5.0 (3.0; 8.0)	5.0 (1.0; 11.0)	3.0 (1.0; 8.0)
	% GAD-7 Score ≥6	243 (6)	48.6%	54.8%	42.6%	47.6%	39.5%
**Smoking Status**	% Current	240 (9)	0.0%	6.5%	5.0%	0.0%	14.0%
	% Former		34.4%	45.2%	41.7%	48.8%	46.5%
	% Never		65.6%	48.4%	53.3%	51.2%	39.5%
**Quality of Life**							
SF-12	Mean (SD) SF-12 PCS	236 (13)	41.2 (11.4)	38 (10.9)	36.3 (11.8)	37.7 (11.9)	32.5 (10.1)
	Mean (SD) SF-12 MCS	236 (13)	44.9 (10.9)	44.5 (10.1)	46.2 (9.2)	44.7 (10.5)	46.8 (11.5)
EQ-5D-5L	Median (LQ, UQ) EQ-5D-5L Score	237 (12)	0.7 (0.6; 0.8)	0.6 (0.5; 0.8)	0.6 (0.5; 0.8)	0.7 (0.2; 0.8)	0.4 (0.2; 0.7)
	Mean (SD) EQ-5D-5L VAS	237 (12)	58.8 (20.7)	55.4 (21.7)	57.1 (21.7)	55.4 (25.8)	50.0 (20.4)
IWQOL-Lite (Standardised Scoring)	Mean (SD) Physical Function	243 (6)	49.5 (20.9)	54.0 (26.1)	57.2 (26.4)	57.7 (29.3)	66.0 (20.0)
	Median (LQ, UQ) Self Esteem	242 (7)	85.7 (67.9; 92.9)	85.7 (60.7; 96.4)	75.0 (53.6; 92.9)	71.4 (50.0; 92.9)	58.9 (39.3; 82.1)
	Mean (SD) Sexual Life	231 (18)	54.1 (26.5)	56.7 (32.4)	53.2 (33.7)	59.0 (31.9)	65.0 (31.2)
	Mean (SD) Public Distress	243 (6)	62.7 (25.8)	61.5 (26.0)	58 (26.8)	55.7 (31.7)	52.0 (25.6)
	Mean (SD) Work	236 (13)	44.3 (29.2)	42.7 (28.2)	38.8 (26.0)	47.3 (34.5)	47.1 (29.7)
	Mean (SD) Total Score	242 (7)	57.4 (18.8)	59.4 (21.6)	57.0 (22.7)	58.1 (26.0)	60.3 (18.2)

For peer review only

**Healthcare Utilisations**	% Use of Any Aids or Specialist Equipment	232 (17)	16.1%	14.5%	24.1%	42.5%	53.7%
**Social Security**	% Unable to Work Due to Illness or Disability	248 (1)	11.1%	17.5%	25.4%	28.6%	47.7%
	% Disability Living Allowance - Caring	236 (13)	9.4%	19.4%	17.2%	14.3%	31.0%
	% Disability Living Allowance - Mobility	236 (13)	12.5%	17.7%	17.2%	16.7%	35.7%

ICIQ-UI SF score: ≥6 = 'moderate incontinence' [1]
PHQ-9 scores: 0-4 = 'minimal depression'; 5-9 = 'mild depression'; 10-14 = 'moderate depression'; 15-19 = 'moderately severe depression'; 20-27 = 'severe depression' [2]
GAD-7 scores: 0-5 = 'mild anxiety'; 6-10 = 'moderate anxiety'; 11-15 = 'moderately severe anxiety'; 15-21 = 'severe anxiety' [3]

References

1. Avery K, Donovan J, Peters TJ, Shaw C, Gotoh M, Abrams P. ICIQ: A brief and robust measure for evaluating the symptoms and impact of urinary incontinence. *Neurourol Urodyn* 2004; **23**(4)**:** 322–330
2. Spitzer RL, Kroenke K, Williams JBW. Validation and utility of a self-report version of PRIME-MD: The PHQ Primary Care Study. *J Am Med Assoc* 1999; **282**(18): 1737-1744
3. Spitzer RL, Kroenke K, Williams JBW, Löwe B. A brief measure for assessing generalized anxiety disorder: The GAD-7. *Arch Intern Med* 2006; **166**(10): 1092-1097

For peer review only

Supplementary Table 4. Association of age and body mass index (BMI) with self-reported comorbidities and mental health indicators.

Comorbidities and Mental Health Indicators	Variable	BMI (Model 1[1]) and Age (Model 2[2])		BMI and Age (Model 3[3])	
		Odds Ratio (95% CI)	p-value	Odds Ratio (95% CI)	p-value
Hypertension (N = 107)	BMI	0.97 (0.72, 1.31)	0.84	0.89 (0.63, 1.24)	0.49
	Age	2.03 (1.47, 2.79)	<0.0001	2.02 (1.44, 2.85)	<0.0001
Type 2 Diabetes (N = 124)	BMI	0.94 (0.69, 1.26)	0.66	0.93 (0.67, 1.30)	0.68
	Age	1.41 (1.06, 1.87)	0.02	1.36 (1.00, 1.85)	0.05
CVD (N = 20)	BMI	0.61 (0.31, 1.18)	0.14	0.71 (0.36, 1.42)	0.34
	Age	2.98 (1.52, 5.85)	<0.01	2.27 (1.10, 4.67)	0.03
Arthritis (N = 73)	BMI	1.45 (1.05, 2.00)	0.02	1.62 (1.09, 2.40)	0.02
	Age	3.01 (2.00, 4.54)	< 0.0001	3.64 (2.26, 5.88)	< 0.0001
Back Problems (N = 115)	BMI	1.09 (0.81, 1.47)	0.57	1.12 (0.81, 1.54)	0.49
	Age	1.21 (0.91, 1.59)	0.19	1.12 (0.83, 1.51)	0.46
Asthma (N = 70)	BMI	1.85 (1.32, 2.59)	<0.001	1.81 (1.27, 2.58)	<0.01
	Age	1.12 (0.82, 1.52)	0.49	1.11 (0.79, 1.57)	0.54
Migraine (N = 49)	BMI	1.55 (1.09, 2.20)	0.02	1.63 (1.11, 2.38)	0.01
	Age	0.85 (0.60, 1.18)	0.33	0.91 (0.62, 1.33)	0.63
Irritable Bowel Syndrome (N = 44)	BMI	0.97 (0.66, 1.44)	0.9	1.00 (0.65, 1.54)	1.00
	Age	0.98 (0.69, 1.41)	0.93	1.07 (0.71, 1.60)	0.76
Sleep Apnoea (N = 66)	BMI	1.69 (1.21, 2.36)	<0.01	1.88 (1.27, 2.77)	<0.01
	Age	2.28 (1.55, 3.34)	< 0.0001	2.05 (1.35, 3.11)	<0.001
PCOS (N = 28)	BMI	0.72 (0.42, 1.23)	0.23	0.74 (0.42, 1.30)	0.29
	Age	0.56 (0.36, 0.88)	0.01	0.56 (0.35, 0.90)	0.02
Gastroesophageal Reflux (N = 97)	BMI	1.02 (0.75, 1.39)	0.88	1.06 (0.76, 1.47)	0.73
	Age	0.73 (0.55, 0.98)	0.04	0.68 (0.50, 0.93)	0.01
Male Incontinence (ICIQ-UI score ≥ 6) (N = 22)	BMI	1.58 (0.81, 3.09)	0.18	1.99 (0.92, 4.30)	0.08
	Age	2.05 (1.05, 4.01)	0.04	2.19 (1.06, 4.53)	0.03
Female Incontinence (ICIQ-UI score ≥ 6) (N = 83)	BMI	1.45 (1.01, 2.09)	0.05	1.40 (0.94, 2.08)	0.10
	Age	1.59 (1.10, 2.28)	0.01	1.49 (1.02, 2.19)	0.04

For peer review only - http://bmjopen.bmj.com/site/about/guidelines.xhtml

**Depression (PHQ-9 score ≥ 10) (N = 107)**	BMI	1.25 (0.92, 1.70)	0.15	1.33 (0.96, 1.86)	0.09
	Age	1.04 (0.79, 1.37)	0.79	0.99 (0.73, 1.34)	0.94
**Anxiety (GAD-7 score ≥ 6) (N = 114)**	BMI	1.42 (1.04, 1.95)	0.03	1.47 (1.05, 2.05)	0.03
	Age	0.91 (0.69, 1.20)	0.49	0.91 (0.67, 1.24)	0.56

[1] Model 1 – Unadjusted model including only the effect of BMI (per 10 kg/m^2) on comorbidity.

[2] Model 2 – Unadjusted model including only the effect of age (per 10 years) on comorbidity.

[3] Model 3 – Model including the effects of age (per 10 years) and BMI (per 10 kg/m^2) on comorbidity, after adjusting additionally for sex, Scottish Index of Multiple Deprivation (SIMD) and smoking status.

Regressions analyses were performed for conditions affecting 10 or more participants only.

For peer review only

BMJ Open

Health and socio-economic burden in treatment-seeking individuals with severe obesity: profile of the Surgical Obesity Treatment Study (SCOTS) national cohort

Journal:	*BMJ Open*
Manuscript ID	bmjopen-2020-046441.R1
Article Type:	Original research
Date Submitted by the Author:	18-Feb-2021

Complete List of Authors:	Mackenzie, Ruth; University of Glasgow Institute of Cardiovascular and Medical Sciences, BHF Glasgow Cardiovascular Research Centre<br>Greenlaw, Nicola; University of Glasgow Robertson Centre for Biostatistics<br>Ali, Abdulmajid; University Hospital Ayr<br>Bruce, Duff; NHS Grampian<br>Bruce, Julie; University of Warwick Warwick Clinical Trials Unit<br>Grieve, Eleanor; University of Glasgow, Institute of Health and Wellbeing<br>Lean, Mike; University of Glasgow School of Medicine Dentistry and Nursing<br>Lindsay, Robert; University of Glasgow Institute of Cardiovascular and Medical Sciences, BHF Glasgow Cardiovascular Research Centre<br>Sattar, Naveed; University of Glasgow, Institute of Cardiovascular and Medical Sciences<br>Stewart, Sally; Glasgow Caledonian University, School of Health and Life Sciences<br>Ford, Ian; University of Glasgow Robertson Centre for Biostatistics<br>Logue, Jennifer; Lancaster University, Lancaster Medical School

**Primary Subject Heading**:	Epidemiology
Secondary Subject Heading:	Surgery
Keywords:	EPIDEMIOLOGY, Adult surgery < SURGERY, Quality in health care < HEALTH SERVICES ADMINISTRATION & MANAGEMENT

SCHOLARONE™
Manuscripts

I, the Submitting Author has the right to grant and does grant on behalf of all authors of the Work (as defined in the below author licence), an exclusive licence and/or a non-exclusive licence for contributions from authors who are: i) UK Crown employees; ii) where BMJ has agreed a CC-BY licence shall apply, and/or iii) in accordance with the terms applicable for US Federal Government officers or employees acting as part of their official duties; on a worldwide, perpetual, irrevocable, royalty-free basis to BMJ Publishing Group Ltd ("BMJ") its licensees and where the relevant Journal is co-owned by BMJ to the co-owners of the Journal, to publish the Work in this journal and any other BMJ products and to exploit all rights, as set out in our licence.

The Submitting Author accepts and understands that any supply made under these terms is made by BMJ to the Submitting Author unless you are acting as an employee on behalf of your employer or a postgraduate student of an affiliated institution which is paying any applicable article publishing charge ("APC") for Open Access articles. Where the Submitting Author wishes to make the Work available on an Open Access basis (and intends to pay the relevant APC), the terms of reuse of such Open Access shall be governed by a Creative Commons licence – details of these licences and which Creative Commons licence will apply to this Work are set out in our licence referred to above.

Other than as permitted in any relevant BMJ Author's Self Archiving Policies, I confirm this Work has not been accepted for publication elsewhere, is not being considered for publication elsewhere and does not duplicate material already published. I confirm all authors consent to publication of this Work and authorise the granting of this licence.

For peer review only

TITLE:

Health and socio-economic burden in treatment-seeking individuals with severe obesity: profile of the Surgical Obesity Treatment Study (SCOTS) national cohort

AUTHORS:

Ruth M. Mackenzie[1], Nicola Greenlaw[2], Abdulmajid Ali[3], Duff Bruce[4], Julie Bruce[5], Eleanor Grieve[6], Mike Lean[7], Robert S. Lindsay[1], Naveed Sattar[1], Sally Stewart[8], Ian Ford[2], Jennifer Logue[9], on behalf of the SCOTS investigators

AFFILIATIONS:

[1]Institute of Cardiovascular and Medical Sciences, University of Glasgow, Glasgow, UK.

[2]Robertson Centre for Biostatistics, University of Glasgow, Glasgow, UK.

[3]University Hospital Ayr, NHS Ayrshire and Arran, Ayr, UK.

[4]NHS Grampian, Aberdeen, UK.

[5]Clinical Trials Unit, University of Warwick, Warwick, UK.

[6]Institute of Health and Wellbeing, University of Glasgow, Glasgow, UK.

[7]School of Medicine, Dentistry and Nursing, University of Glasgow, Glasgow, UK

[8]School of Health and Life Sciences, Glasgow Caledonian University, Glasgow, UK.

[9]Lancaster Medical School, Lancaster University, Lancaster, UK.

For peer review only - http://bmjopen.bmj.com/site/about/guidelines.xhtml

CORRESPONDENCE TO:

Jennifer Logue

Lancaster Medical School

Faculty of Health and Medicine

Lancaster University

LA1 4YG

Telephone: 01524 595133

j.logue1@lancaster.ac.uk

WORD COUNT:

4,469 (including strengths and limitations of this study, funding, data availability, competing interests, author contribution and acknowledgements statements)

4,013 (excluding strengths and limitations of this study, funding, data availability, competing interests, author contribution and acknowledgements statements)

For peer review only

ABSTRACT

Objectives: There is a lack of evidence to inform the delivery and follow-up of bariatric surgery for people with severe obesity. The SurgiCal Obesity Treatment Study (SCOTS) is a national longitudinal cohort of people undergoing bariatric surgery. Here we describe characteristics of the recruited SCOTS cohort, and the relationship between health and socio-economic status with body mass index (BMI) and age.

Participants/Methods: 445 participants scheduled for bariatric surgery at any of 14 centres in Scotland, UK were recruited between 2013 and 2016 for this longitudinal cohort study (1 withdrawal); 249 completed health-related preoperative patient reported outcome measures. Regression models were used to estimate the effect of a 10-unit increase in age or BMI, adjusting for sex, smoking and socio-economic status.

Results: Mean age was 46 years and median BMI was 47 kg/m^2. For each 10 kg/m^2 higher BMI there was a change of -5.2 (95% CI -6.9, -3.5; $p<0.0001$) in Rand 12-item Short Form Survey Physical Component Summary (SF-12 PCS), -0.1 (95% CI -0.2, -0.1; $p<0.0001$) in EuroQoL 5-level EQ-5D version index score, 14.2 (95% CI 10.7, 17.7; $p<0.0001$) in Impact of Weight on Quality of Life-Lite Physical Function score. We observed a 3.1 times higher use of specialist aids and equipment at home (OR 3.1, 95% CI 1.9, 5.0; $p < 0.0001$). Broadly similar results were seen for each 10-year higher age, including a change of -2.1 (95% CI -3.7, -0.5; $p<0.01$) in SF-12 PCS.

Conclusions: Higher BMI combined with older age is associated with poor physical functioning and quality of life in people seeking bariatric surgery treatment. Policy makers must consider the health and care needs of these individuals and invest to provide increased access to effective weight management.

For peer review only

Trial registration: This study was registered prospectively at the International Standard Randomised Controlled Trials Number registry: ISRCTN47072588

STRENGTHS AND LIMITATIONS OF THIS STUDY

- A limitation is that selection for bariatric surgery is often based on the presence of comorbidity so these results, while applicable to a treatment-seeking population, may not be directly applicable to the population with severe obesity in general.
- The fact that almost every bariatric surgery patient in Scotland was recruited is a major strength of this study and renders it more representative than many studies undertaken in the field.
- A further strength is that the majority of questionnaires used were externally validated and wide-ranging, containing a number of unique questions covering medical, social, psychological and physical domains.

INTRODUCTION

Obesity (body mass index [BMI] ≥ 30kg/m^2) and, in particular, severe obesity (BMI ≥ 40kg/m^2) are associated with a variety of negative health outcomes, including increased risk of most major chronic diseases(1). In recent years, severe obesity has emerged as a major public health concern with rates increasing rapidly in a number of countries across the world, including the United States (US) where the prevalence of BMI > 40 kg/m^2 rose by 70% between 2000 and 2010(2), and around 7.7% of adults are now considered to have severe obesity(3). Similarly, levels of severe obesity have risen in the United Kingdom (UK) with 2.9% of all adults in England(4) and 4% of all adults in Scotland now estimated to have a BMI ≥ 40 kg/m^2(5).

For peer review only - http://bmjopen.bmj.com/site/about/guidelines.xhtml

As the prevalence of severe obesity rises, effective treatment is a priority. The efficacy of bariatric surgery for significant long-term weight loss is well established(6,7). However, for the UK National Health Service (NHS), like many other health systems, the commissioning of bariatric surgery is low priority. Indeed, despite obesity prevalence being amongst the highest in the European Union, the UK performs only nine bariatric surgery procedures per 100 000 people(8,9) while Sweden, a country with a similar health service but lower obesity prevalence, performs 70-80 procedures per 100 000 people(10). In North America, the rate of surgery is around 40-50 per 100 000 people, with the majority of these operations performed in the US(8).

The low prioritisation of bariatric surgery within the UK and the strict criteria for access to surgery, including complex pre-surgical pathways and pre-surgical weight-loss requirements(5), results in low numbers of individuals with severe obesity actually receiving surgery. Those receiving surgery generally do so after many years of alternative conservative interventions, at a point when their mean BMI is extremely high, at around 45 kg/m^2, and they are at a median age of 47 years(11). To date, it is unclear how this delay in treatment impacts on health, physical functioning and quality of life.

The SurgiCal Obesity Treatment Study (SCOTS) is the first national epidemiological study established to investigate long-term outcomes following bariatric surgery. This cohort study collected clinical and patient-reported health outcomes from treatment-seeking individuals from across Scotland with severe obesity before they underwent bariatric surgery(12). The aim of the present study is to describe the health-related characteristics of the recruited SCOTS cohort and to examine relationships between age, preoperative BMI and other health-related factors in our recruited sample of adults with severe obesity from across Scotland.

PARTICIPANTS AND METHODS

For peer review only - http://bmjopen.bmj.com/site/about/guidelines.xhtml

This study was registered prospectively at the International Standard Randomised Controlled Trials Number (ISRCTN) registry: ISRCTN47072588. In reporting our findings, we have adhered to the STROBE (STrengthening the Reporting of OBservational studies in Epidemiology) Statement(13) (Supplementary Table 1).

Ethics

A favourable ethical opinion for this study was obtained from the West of Scotland Research Ethics Committee 4 on 07/02/2013 (13/WS/0005).

Patient Involvement

Patients identified via bariatric surgery peer support groups in Scotland were involved in the design and conduct of this research. During the protocol development stage, patients provided input with regard to data collection and defining research questions. In addition, methods of recruitment were informed by discussions with patients during two focus group sessions. Patients were subsequently involved in reviewing paperwork for recruitment and appeared in the recruitment video. There is also a patient member of the independent study steering committee and patients were invited to a meeting to discuss plans for dissemination of study results.

Study Design

SCOTS is a national, prospective, observational cohort study of adults aged over 16 years who were eligible for primary bariatric surgery in Scotland. Participants were recruited over a four year period from December 2013 to February 2017. Recruitment to the cohort has now closed although follow-up will continue until October 2020. A detailed protocol for the SCOTS study was published previously(12) and is included as Supplementary File 1, but was amended in 2016 to reflect a smaller cohort size and shorter follow-up. This was in response to changes in national service commissioning contributing to lower numbers of bariatric surgery which, in turn, affected recruitment potential and planned sample size. Any changes from the original protocol are outlined below.

For peer review only

For peer review only - http://bmjopen.bmj.com/site/about/guidelines.xhtml

Participant and Centre Eligibility

As outlined in the protocol(12) (Supplementary File 1), patients scheduled to undergo a primary bariatric surgery procedure at any of the ten NHS-funded or four private hospitals in Scotland providing this surgery were eligible for invitation to the study. Inclusion criteria were that patients were aged 16 years or over, and undergoing their first bariatric surgery procedure. Patients were required to have capacity to consent, provide written informed consent and be resident in Scotland. Patients who did not meet these criteria or who did not have English language skills to complete written questionnaires were ineligible(12) (Supplementary File 1).

Recruitment Procedures and Consent

Patients were approached about the study at least four weeks prior to their primary bariatric surgical procedure by the clinical bariatric surgery team or by a research nurse in preoperative assessment clinics. Written signed consent for access to medical records, linkage of electronic health records, and for postal questionnaires was obtained on a subsequent clinical visit.

Data Collection

Recruited participants were asked to complete questionnaires preoperatively and at two and three years postoperatively. Prior to receiving questionnaires, participants were made aware (via patient information leaflets) that the estimated time for completion of each questionnaire was one hour. The approximate time it would take to complete the questionnaire (one hour) was also stated clearly on the first page of each questionnaire received by participants. Completion could be either by post or electronically via a secure link sent by email. Two reminders were sent by the participant's chosen method and a third reminder, if required, was sent by post to all participants. No further strategy was used after three reminders.

Baseline preoperative questionnaires collected health-related information, including weight, medical history, smoking status, alcohol use, gastrointestinal symptoms, urological health, depression,

For peer review only

anxiety, health-related quality of life (HRQoL) and obesity-specific quality of life (O-QoL), life optimism, physical activity, healthcare utilisation, employment, and social security. Comorbidity was assessed by self-report using a questionnaire designed specifically for this study (Supplementary File 2). Gastrointestinal symptoms were evaluated using a questionnaire developed for the REFLUX trial(14). Urological health was assessed using the International Prostate Symptom Score (IPSS)(15) and the International Consultation on Incontinence Questionnaire-Urinary Incontinence Short Form (ICIQ-UI SF) wherein a score ≥ 6 indicates moderate incontinence(16). Female reproductive health data was obtained using a modified version of the questionnaire developed for the Longitudinal Assessment of Bariatric Surgery (LABS-2) study(17). Information on male erectile dysfunction was obtained using a modified version of the questionnaire developed for the Massachusetts Male Ageing Study(18). Anxiety and depression were assessed using the Generalised Anxiety Disorder Assessment (GAD-7)(19) and Patient Health Questionnaire (PHQ)-9 (PHQ-9)(20) instruments respectively. A PHQ-9 score ≥ 10 is indicative of moderate to severe depression while a GAD-7 score ≥ 6 is reflective of moderate to severe anxiety(19,20). Smoking status was ascertained using a questionnaire specifically developed for this study (Supplementary File 2). Alcohol use was determined using a modified version of the Alcohol Use Disorders Identification Test (AUDIT)(21). HRQoL was assessed using the Rand 12-item Short Form Survey (SF-12)(22) and EuroQoL 5-level EQ-5D version (EQ-5D-5L)(23,24) instruments while O-QoL was assessed using the Impact of Weight on Quality of Life-Lite (IWQOL-Lite) questionnaire(25). Standardised scoring was used when interpreting IWQOL-Lite questionnaires(26). Life optimism was determined using a modified version of the Life Orientation Test (LOT) wherein a score range of 0-13 reflects low optimism (high pessimism)(27). Data on physical activity was obtained using the International Physical Activity Questionnaire (IPAQ) – Short Form(28). Information on participants’ employment, social security status, and healthcare utilisation was obtained using questionnaires specifically developed for this study (Supplementary File 2).

For peer review only - http://bmjopen.bmj.com/site/about/guidelines.xhtml

Each participant's quintile of the Scottish Index of Multiple Deprivation (SIMD), an area-based measure of socioeconomic status(29), was derived from their postcode. Combining a number of indicators of socioeconomic status across seven domains, the SIMD provides a relative measure of deprivation which can be used to compare data zones by ranking them from most to least deprived. The seven domains include income, employment, health, education, skills and training, housing, geographic access, and crime(29).

Height and weight data were collected by clinical staff at recruitment allowing BMI to be calculated.

We herein report baseline data recorded during the recruitment visit for the whole recruited cohort before bariatric surgery, and patient-reported outcomes for the sub-set completing baseline questionnaires.

Statistical Analyses

Continuous data are reported as means and standard deviations (SD) or medians and lower (Q1) and upper (Q3) quartiles depending on data distribution, and counts and percentages are reported for categorical data. Age and BMI data were categorised *a priori*; BMI (kg/m^2) is reported as 5-unit bands (< 40, 40-44, 45-49, 50-54, ≥ 55) and age was categorised using 10-year age bands (< 35 years, 35-44, 45-49, 50-54 and ≥ 55 years). Other baseline demographics are summarised by group.

Linear regression models were used to examine continuous quality of life measures and logistic regression for the binary 'need for specialist aids' and 'equipment in the home to assist with daily living'. Models were used to estimate the effect of a 10 unit increase in age or BMI in 1) an unadjusted model and 2) in a model adjusted for sex, SIMD, smoking status and BMI or age respectively as these factors may be associated with obesity and related comorbidity. Regression model effect estimates, or odds ratios (ORs), and corresponding 95% confidence intervals (CIs) and associated p-values are provided. Data was analysed as available, without any imputation for missing data. All analyses were performed using SAS (version 9.3).

For peer review only

RESULTS

Over the three year recruitment period, a total of 548 patients were approached and screened for eligibility to participate. Of these, 103/548 (19%) were excluded or declined to participate (Figure 1). We recruited 445/548 (81%) participants but one participant withdrew consent leaving a recruited sample of 444 (81%). Of the recruited sample, 413/444 (93%) consented to data linkage and questionnaire follow-up whilst 31/444 (7%) consented to data linkage only. Of these 413 participants, a total of 164/413 (40%) were not included in the subsequent analysis: 129 did not return a baseline questionnaire and 35 had bariatric surgery before their baseline patient reported outcome measures (PROMs) questionnaires were completed. Of the 129 who did not return baseline questionnaires, 84/129 (65%) progressed to surgery, 43/129 (33%) did not progress to surgery and the status of 2/129 (2%) was unknown. Completed preoperative baseline PROMs data for 249/413 participants (60% of those consented) were available for analysis (Figure 1).

Characteristics of recruited and analysed sample

Demographic data are summarised in Table 1. Participant characteristics were similar between the total recruited sample (n= 444) and the analysed sub-set (n = 249) with completed PROMS before bariatric surgery (Table 1). Mean age was 46 years (SD 9.1 years) with a higher proportion of women than men (71% vs 29%). Half of recruited participants were aged 35 to 49 years, with one third being over 50 years. The median BMI was 47 kg/m^2 (Q1 43; Q3 54) with more than 21% having a BMI of ≥ 55+ kg/m^2. Over half of the participants (55%) lived in areas of high socio-economic deprivation (SIMD quintiles 1 and 2). There were no statistically significant differences between the analysed sub-set (n = 249) and the non-analysed sub-set (n = 195).

For peer review only

For peer review only - http://bmjopen.bmj.com/site/about/guidelines.xhtml

Table 1. Baseline sociodemographic characteristics of recruited (n = 444) and analysed (n = 249) samples of SCOTS participants.

Characteristic		Recruited Sample N = 444	Analysed Sample* N = 249
Sex, N (%)	Male	123 (27.7)	72 (28.9)
	Female	321 (72.3)	177 (71.1)
	Missing	0	0
Age (years)	Mean (SD)	46.2 (9.1)	45.9 (9.1)
	Missing	0	0
Age Group, N (%)	< 35 years	61 (13.7)	36 (14.5)
	35-44 years	116 (26.1)	63 (25.3)
	45-49 years	109 (24.5)	63 (25.3)
	50-54 years	79 (17.8)	43 (17.3)
	55+ years	79 (17.8)	44 (17.7)
	Missing	0	0
BMI (kg/m^2)	Median (Q1, Q3)	47.2 (42.7 ; 53.6)	47.6 (42.8 ; 53.8)
	Missing	1	0
BMI Group, N (%)	BMI < 40	52 (11.7)	24 (9.6)
	BMI 40-44	115 (26.0)	64 (25.7)
	BMI 45-49	116 (26.2)	64 (25.7)
	BMI 50-54	71 (16.0)	44 (17.7)
	BMI 55+	89 (20.1)	53 (21.3)
	Missing	1	0
SIMD Quintile, N (%)	Quintile 1 (most deprived)	135 (30.5)	70 (28.3)
	Quintile 2	108 (24.4)	65 (26.3)
	Quintile 3	84 (19.0)	51 (20.6)
	Quintile 4	68 (15.4)	34 (13.8)
	Quintile 5 (least deprived)	47 (10.6)	27 (10.9)
	Missing	2	2
Marital Status, N (%)	Married/civil partnership/co-habiting	Not collected	155 (63)
	Single/separated/divorced/ widowed		91 (37)
	Missing		3
Ethnic Group, N (%)	White	Not collected	243 (97.6)
	Mixed		4 (1.6)
	Asian/Asian Scottish/Asian British		1 (0.4)
	African Caribbean/Black		1 (0.4)
	Other		0 (0.0)
	Missing		0
Education, N (%)	School only	Not collected	58 (23.5)
	Formal qualifications through training at work		54 (21.9)
	Qualification (other than a degree from college or university)		64 (25.9)
			71 (28.7)

For peer review only

For peer review only - http://bmjopen.bmj.com/site/about/guidelines.xhtml

	Degree from college or university<br>Missing		 2
Current Employment Status, N (%)	Working full time<br>Working part time<br>Unable to work because of illness or disability<br>Student/unemployed and seeking employment/ unemployed and not seeking employment/ carer/other<br>Missing	Not collected	124 (50.0)<br>24 (9.7)<br>64 (25.8)<br>36 (14.5)<br>1

Working full time = ≥ 30 hours per week; working part-time = < 30 hours per week. Percentages may not total 100% in all cases due to rounding. *Participants who returned baseline questionnaires prior to their bariatric surgery are included in the analysed sample.

For peer review only

Comorbidities

For the analysed sample (n = 249), self-reported medical comorbidities, and physical, mental and functional measures are presented in Table 2. Over 40% reported having at least one of hypertension, type 2 diabetes (T2D), back problems, anxiety/depression and gastroesophageal reflux. Over 60% of the sample reported more than three comorbidities. Over 40% of male participants reported erectile dysfunction while one third of males described urinary incontinence. Half of female participants reported urinary incontinence. Mean depression scores reflected mild depression, although 44% of participants had scores indicating moderate to severe depression. Anxiety scores for all participants were indicative of mild anxiety (median 5.0); with half of participants having scores indicative of moderate to severe anxiety. The mean life optimism score for participants was reflective of low optimism (high pessimism). Very few participants smoked (5%) and, on average, alcohol consumption was moderate.

For peer review only

For peer review only - http://bmjopen.bmj.com/site/about/guidelines.xhtml

246 **Table 2. Preoperative health-related characteristics of SCOTS participants undergoing bariatric**
247 **surgery (n = 249).**

		**N = 249**<br>**N (%)**	**Missing**<br>**N (%)**
**Comorbidity, Self-report**	DVT	8 (3.2)	0
	Pulmonary embolism	4 (1.6)	0
	Hypertension	107 (43.0)	0
	Type 2 diabetes	124 (49.8)	0
	Angina/heart attack	17 (6.8)	0
	Heart failure	2 (0.8)	0
	Stroke/mini stroke	6 (2.4)	0
	Arthritis	73 (29.3)	0
	Back problems	115 (46.2)	0
	Chronic bronchitis	4 (1.6)	0
	Eczema/psoriasis	33 (13.3)	0
	Asthma	70 (28.1)	0
	Thyroid problems	32 (12.9)	0
	Migraine	49 (19.7)	0
	Anxiety/depression	114 (45.8)	0
	Kidney disease	7 (2.8)	0
	Liver disease	2 (0.8)	0
	Cancer	4 (1.6)	0
	Irritable bowel syndrome	44 (17.7)	0
	Sleep apnoea	66 (26.5)	0
	CVD	20 (8.0)	0
**N (%) Self-reported Comorbidities**	None	9 (3.6)	0
	1-2	80 (32.1)	0
	≥ 3	160 (64.3)	0
**Gastrooesophageal Reflux**	Yes	97 (40.4)	9 (3.6)

For peer review only - http://bmjopen.bmj.com/site/about/guidelines.xhtml

**Female Reproductive Health N = 75***	Mean (SD) age years, last natural menstrual period	39.4 (10.9)	4 (5.3)
**Female Reproductive Health N = 177**	PCOS, N (%)	28 (16.8)	10 (5.6)
**Male Reproductive Health N = 72**	Impotence, N (%)	28 (41.2)	4 (5.6)
	IPSS score ≥8, N (%)	34 (47.9)	1 (1.4)
**Incontinence**	Median (Q1, Q3) ICIQ-UI SF score	4 (0.0; 10.0)	10 (4.0)
	ICIQ-UI SF score ≥6	105 (43.9)	10 (4.0)
Incontinence, Females N = 177	ICIQ-UI SF score ≥6, N (%)	83 (49.4)	9 (5.1)
Incontenence, Males N = 72	ICIQ-UI SF score ≥6, N (%)	22 (31.0)	1 (1.4)
**Depression**	Mean (SD) PHQ-9 score	9.6 (6.3)	5 (2.0)
	N (%) PHQ-9 score ≥10	107 (43.9)	5 (2.0)
**Anxiety**	Median (Q1, Q3) GAD-7	5 (2.0; 9.0)	6 (2.4)
	N (%) GAD-7 score ≥6	114 (46.9)	6 (2.4)
**Smoking Status**	Current	13 (5.4)	9 (3.6)
	Former	105 (43.8)	
	Never	122 (50.8)	
**Alcohol Use**	Median (Q1, Q3) AUDIT	3 (1.0; 6.0)	20 (8.0)
**Quality of Life**			
SF-12	Mean (SD) PCS	37 (11.4)	13 (5.2)
	Mean (SD) MCS	45.5 (10.3)	13 (5.2)
EQ-5D-5L	Median (Q1, Q3)	0.6 (0.3; 0.8)	12 (4.8)
	Mean (SD) VAS	55.3 (22.1)	12 (4.8)
IWQOL-Lite (Standardised Scoring)	Mean (SD) Physical Function	56.9 (25.4)	6 (2.4)
	Mean (SD) Self Esteem	70.7 (27.1)	7 (2.8)
	Mean (SD) Sexual Life	57.1 (31.7)	18 (7.2)

For peer review only

	Mean (SD) Public Distress	58.1 (27.2)	6 (2.4)
	Mean (SD) Work	43.6 (29.2)	13 (5.2)
	Mean (SD) Total score	58.5 (21.7)	7 (2.8)
**Life Optimism**	Mean (SD) LOT score	13 (4.9)	14 (5.6)
**Physical Activity**	≥1 walking, moderate or vigorous activity in last 7 days	201 (83.4)	8 (3.2)
	Median (Q1, Q3) IPAQ score**	720 (40.0; 1800.0)	6 (3.0)
**Healthcare Utilisations**	Using any aids or specialist equipment	67 (28.9)	17 (6.8)
	Median (Q1, Q3) GP visits in last 3 months	2 (1.0; 3.0)	79 (31.7)
	Median (Q1, Q3) visits to other health/social care providers in last 3 months	3 (1.0; 5.0)	73 (29.3)
**Social Security**	Unable to work due to illness or disability	64 (25.8)	1 (0.4)
	Receiving Disability Living Allowance (caring)	44 (18.6)	13 (5.2)
	Receiving Disability Living Allowance (mobility)	47 (19.9)	13 (5.2)

DVT, deep vein thrombosis; CVD, cardiovascular disease; PCOS, polycystic ovary syndrome; GP, general practitioner. IPSS Scores: 0-7 = 'Mild symptoms'; 8-19 = 'Moderate symptoms'; 20-35 = 'Severe symptoms' [15]. ICIQ-UI SF score: ≥6 = ‘moderate incontinence’ [16]. PHQ-9 scores: 0-4 = 'minimal depression'; 5-9 = 'mild depression'; 10-14 = 'moderate depression'; 15-19 = 'moderately severe depression'; 20-27 = 'severe depression' [20]. GAD-7 scores: 0-5 = 'mild anxiety'; 6-10 = 'moderate

For peer review only

anxiety'; 11-15 = 'moderately severe anxiety'; 15-21 = 'severe anxiety'[19]. * 75/177 (42%) female participants reported not menstruating in the last 12 months. ** MET minutes/week.

For peer review only

Health and obesity-related quality of life

Mean SF-12 Physical Component Summary (PCS) and Mental Component Summary (MCS) scores were low; PCS 37.0 (11.4), MCS 45.5 (10.3). The median EQ-5D-5L score of sample participants was 0.6 (0.3; 0.8) while the mean EQ-5D-5L visual analogue scale (VAS) was 55.3 (22.1). Participants had a mean IWQOL-Lite Physical Function score of 56.9 (25.4) and a mean total score of 58.5 (21.7) (Table 2) where an increase in IWQOL-Lite score indicates a worsening in quality of life.

Physical activity

Over 80% of SCOTS participants reported undertaking at least 10 minutes of either walking, moderate or vigorous activity in the last seven days and the median IPAQ score for the sample was 720.0 MET minutes/week. Almost one third (29%) of participants reported using aids or specialist equipment to assist with their daily activities in the home (Table 2).

Comorbidity by BMI and age

Comorbidity data is presented by BMI group and age group in Supplementary Tables 2 and 3 respectively. In order to further investigate the associations between BMI and age on physical, mental and functional measures, and healthcare utilisation within the SCOTS population, regression analyses were performed (Table 3). Higher BMI values and higher ages were negatively associated with physical, but not mental, HRQoL scores (Table 3). For each 10 kg/m^2 higher BMI there was a change of -5.2 (95% CI -6.9, -3.5; p<0.0001) in SF-12 PCS, -0.1 (95% CI -0.2, -0.1; p<0.0001) in EQ-5D-5L score and 14.2 (95% CI 10.7, 17.7; p<0.0001) in IWQOL-Lite Physical Function score (where an increase in score indicates a worsening). We observed a 3.1 times higher use of specialist aids and equipment in the home (OR 3.1, 95% CI 1.9, 5.0; p < 0.0001), adjusting for age, sex, smoking and socio-economic deprivation. For each 10-year higher age, there was a change of -2.1 (95% CI -3.7, -0.5; p<0.01) in SF-12 PCS score, -0.1 (95% CI -0.1, 0.0; p<0.01) in EQ-5D-5L score and 5.01 (95% 1.8, 8.3; p<0.01) in IWQOL-Lite Physical Function score, and a 3.4 (OR 3.4, 95% CI 1.9, 5.0; p<0.0001)

For peer review only - http://bmjopen.bmj.com/site/about/guidelines.xhtml

times higher use of specialist aids or equipment in the home, adjusting for BMI, sex, smoking and socio-economic status.

With regard to medical comorbidities, as shown in Supplementary Table 4, higher BMI had a significant association with higher prevalence of asthma in the SCOTS population, while older age was associated with higher prevalence of hypertension, arthritis and sleep apnoea (Supplementary Table 4).

For peer review only

For peer review only - http://bmjopen.bmj.com/site/about/guidelines.xhtml

Table 3. Association of age and body mass index (BMI) with quality of life (QoL), use of specialist equipment in the home and social security.

QoL Indicators and Functional Measures	Variable	Unadjusted Models[1]	Adjusted Model[2]
		**Regression Coefficient (95% CI)[3]**	**Regression Coefficient (95% CI)[3]**
**SF-12 PCS**	BMI	-4.91 (-6.55, -3.28)	-5.21 (-6.90, -3.52)
	Age	-2.44 (-4.03, -0.85)	-2.14 (-3.73, -0.54)
**SF-12 MCS**	BMI	-0.49 (-2.07, 1.09)	-0.40 (-2.06, 1.26)
	Age	0.42 (-1.04, 1.87)	0.68 (-0.89, 2.25)
**EQ-5D-5L Score**	BMI	-0.11 (-0.15, -0.06)	-0.11 (-0.16, -0.06)
	Age	-0.08 (-0.12, -0.03)	-0.07 (-0.11, -0.02)
**EQ-5D-5L VAS**	BMI	-7.08 (-10.38, -3.79)	-7.73 (-11.10, -4.36)
	Age	-2.19 (-5.30, 0.92)	-0.65 (-3.77, 2.46)
**IWQOL-Lite Physical Function**	BMI	13.72 (10.28, 17.17)	14.20 (10.69, 17.70)
	Age	5.77 (2.28, 9.26)	5.01 (1.75, 8.27)
**IWQOL-Lite Self Esteem**	BMI	5.09 (1.02, 9.16)	5.99 (1.86, 10.12)
	Age	-4.74 (-8.51, -0.97)	-5.11 (-8.96, -1.25)
**IWQOL-Lite Sexual Life**	BMI	5.56 (0.71, 10.41)	5.74 (0.82, 10.67)
	Age	3.86 (-0.75, 8.47)	3.01 (-1.71, 7.73)
**IWQOL-Lite Public Distress**	BMI	15.36 (11.72, 19.00)	16.07 (12.34, 19.80)
	Age	-2.78 (-6.57, 1.02)	-3.04 (-6.51, 0.44)
**IWQOL-Lite Work**	BMI	9.54 (5.25, 13.84)	9.59 (5.16, 14.02)
	Age	1.95 (-2.25, 6.16)	1.04 (-3.15, 5.24)
**IWQOL Total Score**	BMI	10.31 (7.29, 13.33)	10.88 (7.79, 13.97)
	Age	1.20 (-1.84, 4.24)	0.55 (-2.33, 3.43)
**Use of Aids or Specialist Equipment**	BMI	2.34 (1.62, 3.39)*	3.10 (1.94, 4.95)*
	Age	2.65 (1.76, 4.00)*	3.40 (1.94, 4.95)*
**Disability Living Allowance (Caring)**	BMI	1.19 (0.82, 1.73)*	1.07 (0.72, 1.59)*
	Age	1.54 (1.03, 2.31)*	1.62 (1.08, 2.46)*
**Disability Living Allowance (Mobility)**	BMI	1.18 (0.82, 1.70)*	1.13 (0.77, 1.65)*
	Age	1.64 (1.10, 2.45)*	1.62 (1.08, 2.44)*

[1] Unadjusted models including only the effect of BMI (per 10 kg/m^2) or age (per 10 years) on QoL indicators and functional measures. [2] Adjusted model including the effects of BMI (per 10 kg/m^2) and age (per 10 years) on QoL indicators and functional measures, after adjusting additionally for sex, Scottish Index of Multiple Deprivation (SIMD) and smoking status. [3] Regression coefficient (95% CI) is Estimate (95% CI) for results from the linear regression and Odds Ratio (95% CI) for results from the logistic regression. Odds Ratio results are indicated with a *. N ≥ 10 for all indicators and measures for which regression analyses were performed.

For peer review only

DISCUSSION

Despite escalating levels of severe obesity in Western society and the concomitant increase in bariatric surgical procedures being performed in some countries(30), there is a dearth of information on the health status of people living with severe obesity. This is the first report from the national Scottish cohort study of people seeking surgical treatment for severe obesity. We recruited 444 adults from 14 centres across Scotland over a three year period, including all NHS centres and major private hospitals undertaking bariatric surgery. We found that higher BMI and older age were associated with decreased physical quality of life, increased use of specialist aids and equipment in the home, and a high prevalence of comorbidities.

There has been a significant increase in the prevalence of a BMI ≥ 40kg/m^2 in recent decades; in Scotland, obesity prevalence has trebled in women age 16-64 since 1995. However, it is hard to assess the global increase due to lack of reporting of BMI ≥ 40kg/m^2 in national health survey data(31). While it is known that health care resource use increases in people with a BMI ≥ 30kg/m^2 , with service use estimated to be over 25% higher than for those with a BMI in the normal weight range(32), few data exist for those with BMI 40kg/m^2 and above. In 2016, the Global BMI Mortality Collaboration(33) conducted an individual-participant-data meta-analysis of 239 prospective studies and found a 2.8 times increased risk of all cause mortality for people with a BMI of 40-60kg/m^2. Greive *et al.*(34) conducted a systematic review which focussed on the economic cost of severe obesity (BMI ≥ 40kg/m^2) and found limited literature describing increased prescribing, outpatient utilisation and intensive care admission, and hospital length of stays during critical illness. However, in both studies there was no dissagregation of BMI beyond > 40kg/m^2 meaning that the health consequences of severe obesity are not yet fully described.

There has been extensive research on the relationship between health-related quality of life and obesity(35). Ul-Haq *et al.*(36) performed a meta-analysis of eight studies (43 086 particpants) and found physical quality of life, measured by the Rand 36-item Short Form Health Survey (SF-36), was

For peer review only

reduced by 9.7 points in those with BMI 40kg/m^2 compared to those with a BMI in the normal range, although, again, there was no disaggregation above BMI 40kg/m^2. Van Nunen *et al.*(37) perfomed a meta-analysis to compare the general, non-treatment seeking population to patients within weight management programmes and those seeking bariatric surgery. They found that those seeking surgical treatment reported the most severely reduced HRQoL, perhaps reflecting their reasons for seeking definitive surgical treatment. Our cohort of treatment-seeking individuals who completed a rich battery of patient reported measures, provides data to show that health-related and obesity-related quality of life of those with the highest body mass is extremely poor and this is compounded by increasing age. Quality of life scores of SCOTS participants in both the upper BMI (≥ 55 kg/m^2) and older age (≥ 55 years) groups included physical scores comparable with those reported by cancer patients receiving palliative care(38), patients with chronic heart failure expressing end of life preferences(39), and patients with end stage kidney disease(40). Furthermore, patients with severe chronic obstructive pulmonary disease (COPD) report higher quality of life scores, indicating a better quality of life, than our cohort of treatment-seeking obese participants(41). As far as we are aware, this is the first study to investigate physical and mental health in patients with severe obesity awaiting bariatric surgery with finer level consideration of BMI up to ≥ 55kg/m^2.

UK guidelines(42) currently indicate that bariatric surgery is a treatment option for those with a BMI ≥ 40 kg/m^2, or between 35 kg/m^2 and 40 kg/m^2 in the presence of other significant diseases (T2D or hypertension, for example) which could be improved if they lost weight. Non-surgical weight management must have been attempted but not resulted in clinically beneficial weight loss before surgery is indicated. However, baseline SCOTS data appear to suggest that the low prioritisation of bariatric surgery and a lengthy preoperative pathway in the UK is associated with surgical treatment being reserved for individuals at an older age with very high BMI. Indeed, in 2018, the Global Registry initiative of the International Federation for the Surgery of Obesity and Metabolic Disorders (IFSO) reported a global median pre-bariatric surgery BMI of 41.7 kg/m^2(43), as compared to 47.6 kg/m^2 in the SCOTS cohort. Similarly, IFSO reported a median patient age of 42 years at the time of

For peer review only - http://bmjopen.bmj.com/site/about/guidelines.xhtml

bariatric surgery(43), as compared to a median age of 47 years for SCOTS participants. This combination of higher BMI and older age means that, at the time of surgery, Scottish patients have high levels of comorbidity and poor physical functioning. Bariatric surgery is considered a highly cost-effective intervention(44). However, the health economic models rely on data primarily from US and Scandinavian studies(45–49), where BMI and age at the time of surgery are lower than in the UK. Higher BMI and older age are risk factors for post-operative complications(50,51) and also associated with lower total weight loss(52–54). Type 2 diabetes remission rates are negatively correlated with age(55). As such, focussing bariatric surgery provision on those with older age and higher BMI may result in higher costs of surgery with increased length of hospital stay, higher rates of post-operative complications, lower overall weight loss and lower rates of disease remission. Consequently, the impressive health benefits and resultant cost-savings of bariatric surgery observed in clinical trials and observational cohorts from other countries may not be fully realised for the UK/Scottish population.

The SCOTS dataset represents a unique and rich resource. A major strength of the study is its representativeness. Indeed, every clinical team providing publicly-funded bariatric surgery in Scotland approached their patients for recruitment to the study, rendering it highly representative of the population in comparison to other studies undertaken in the field. However, the number of participants with valid baseline questionnaires was lower than anticipated. In many cases this could be attributed to the participant undergoing surgery before completing the questionnaire, or the participant leaving the bariatric surgery pathway before surgery. The overall length of the questionnaire may have also played a role. Participants living in the most deprived areas were well represented in our cohort and the mean quality of life findings were broadly similar to those of bariatric surgery cohorts from across the world(56–58). A further strength of the study is that questionnaires were externally validated and wide-ranging, containing a number of unique questions covering medical, social, psychological and physical functioning domains. This wide range of self-reported health measures will allow us to account for a range of potentially mediating and

For peer review only - http://bmjopen.bmj.com/site/about/guidelines.xhtml

confounding factors in future analyses. In addition, we have revealed the extent of comorbidities, including musculoskeletal, urinary and mental health problems affecting people with severe obesity. Low numbers of some comorbidities meant that this could not be a focus of this analysis.

A limitation of this study is that selection for bariatric surgery is often based on the presence of comorbidity so these results, while applicable to a treatment-seeking population, may not be directly applicable to the whole population with severe obesity in the wider society. While we will have access to medical records via electronic health record data linkage in follow-up, the current analyses are based on self-report of selected comorbidities. It is well known that self-reported weights are underreported, particularly by people with very high BMI(59). However, we are confident of the accuracy of weight and height as these data were collected in clinic during the recruitment visit. With regard to future analyses, follow-up of the SCOTS cohort is ongoing which will allow us to report the longitudinal health trajectory after bariatric surgery. The electronic health record infrastructure in Scotland(60) will also permit us to study health outcomes of SCOTS participants across a range of clinical events and, ultimately, determine the effect of bariatric surgery and other factors associated with health outcomes.

Obesity is a multisystem disease which affects every facet of a person’s life. Our data have shown that higher body mass index combined with older age is associated with very poor physical functioning, and health-related and obesity-related quality of life. Indeed, quality of life scores for those living with severe obesity in Scotland are akin to those seen in the end stage of diseases such as cancer and heart failure. The health consequences of severe obesity and the extent to which treatments such as bariatric surgery can improve these are not yet known. Researchers should ensure that they include people with severe obesity in population cohorts and treatment studies, and study the impact of severe obesity in more detail; there are substantial differences in the health

For peer review only

status of those with a BMI above 50kg/m^2 and those whose BMI is around 40kg/m^2. Policy makers should consider the health and care needs of the growing numbers of inidviduals living with obesity. There will be considerable future demand for health care and services must be designed to accomodate the physical needs of the individuals. While primary prevention of obesity is clearly paramount to avoid more people developing such a debilitating, chronic condition, investment is urgently needed, both in the UK and globally, to provide increased access to bariatric surgery and other forms of effective weight management, directly targeting patient groups who will benefit from surgical intervention as early in the disease course as possible.

FUNDING

This work was supported by the National Institute for Health Research (NIHR) Health Technology Assessment Programme (project number 10/42/02). The views expressed are those of the authors and not necessarily those of the NIHR or the Department of Health and Social Care. Professor J. Bruce is supported from NIHR Research Capability Funding via University Hospitals Coventry and Warwickshire.

DATA AVAILABILITY

Deidentified participant data will be available upon reasonable request by bona fide researchers after completion of the study and our current funded analyses (estimated early 2022). Data requests should be submitted in the first instance to j.logue1@lancaster.ac.uk and will be reviewed by the study's publication committee.

COMPETING INTERESTS

For peer review only

Professor Lean has received advisory board and speaker fees from Eli Lilly, Novo Nordisk, Merck and Sanofi. Dr Lindsay has received advisory board and speaker fees from Eli Lilly, Novo Nordisk and Servier. Professor Sattar has consulted for Eli Lilly, Novo Nordisk, Pfizer and Sanofi and received compensation. All other authors declare no potential competing interests.

AUTHOR CONTRIBUTION

RMM, NG, AA, DB, JB, EG, ML, RSL, NS, SS, IF and JL were involved in the conception, planning and design of the study, revising it critically and approving the final version for submission. NG conducted the analyses. RMM and JL wrote the first draft. All authors agree to be accountable for all aspects of the work and to ensure that questions relating to the accuracy or integrity of any part of the work are appropriately investigated and resolved.

ACKNOWLEDGEMENTS

These data have been made available for analysis by members of the SCOTS Investigators group, Mr Afshin Alijani, Dr Ewan Bell, Professor Andrew Collier, Ms Carol Craig, Dr Jennifer Darrion, Mr David Galloway, Mr Hasan Kasem, Ms Joeleen McKean, Mr Stuart Oglesby, Mr Chris Shearer, Mr Rob Stuart and Mr Bruce Tulloh, due to their hard work in collecting data, and the patients who have participated in the study. The authors also wish to thank Mr Richard Welbourn, Ms Catherine Quinn, Professor David Beard, Dr John Mooney, Ms Elizabeth Thompson, Mrs Joanne O'Donnell, Mrs Jane Munro, Dr Iona Donnelly and Dr Samantha Alvarez Madrazo.

REFERENCES

1 Kitahara CM, Flint AJ, Berrington de Gonzalez A, Bernstein L, et al. Association between Class

For peer review only

For peer review only - http://bmjopen.bmj.com/site/about/guidelines.xhtml

III Obesity (BMI of 40-59 kg/m2) and Mortality: A Pooled Analysis of 20 Prospective Studies. *PLoS Med* 2014;11(7):e1001673

2 Sturm R, Hattori A. Morbid obesity rates continue to rise rapidly in the United States. *Int J Obes* 2013;37(6):889-891

3 Flegal KM, Kruszon-Moran D, Carroll MD, et al. Trends in obesity among adults in the United States, 2005 to 2014. *JAMA* 2016;315(21):2284-2291

4 Baker C. House of Commons Library | Obesity Statistics. *Obes Stat (Briefing Pap Number 3336)* 2019;3336:1-18

5 Grieve E, Mackenzie RM, Munro J, et al. Variations in bariatric surgical care pathways: A national costing study on the variability of services and impact on costs. *BMC Obes* 2018;5(1):43

6 Colquitt JL, Pickett K, Loveman E, et al. Surgery for weight loss in adults. *Cochrane Database Syst Rev* 2014;issue 8:CD003641

7 O'Brien PE, McPhail T, Chaston TB, et al. Systematic review of medium-term weight loss after bariatric operations. *Obes Surg* 2006;16:1032-1040

8 Angrisani L, Santonicola A, Iovino P, et al. Bariatric Surgery Worldwide 2013. *Obes Surg* 2015;25(10):1822-1832

9 Welbourn R, Le Roux CW, Owen-Smith A, et al. Why the NHS should do more bariatric surgery; how much should we do? *BMJ (Online)* 2016;353:i1472

10 Hedenbro JL, Näslund E, Boman L, et al. Formation of the Scandinavian Obesity Surgery Registry, SOReg. *Obes Surg* 2015;25(10):1893-1900

11 Booth HP, Khan O, Fildes A, et al. Changing Epidemiology of Bariatric Surgery in the UK: Cohort Study Using Primary Care Electronic Health Records. *Obes Surg* 2016;26(8):1900-1905

For peer review only

For peer review only - http://bmjopen.bmj.com/site/about/guidelines.xhtml

12 Logue J, Stewart S, Munro J, et al. SurgiCal obesity treatment study (SCOTS): Protocol for a national prospective cohort study of patients undergoing bariatric surgery in Scotland. *BMJ Open* 2015;5(5):e008106

13 von Elm E, Altman DG, Egger M, et al. The Strengthening the Reporting of Observational Studies in Epidemiology (STROBE) statement: guidelines for reporting observational studies. *Lancet* 2007;370(9596):1453-1457

14 Macran S, Wileman S, Barton G, et al. The development of a new measure of quality of life in the management of gastro-oesophageal reflux disease: The Reflux questionnaire. *Qual Life Res* 2007;16(2):331-343

15 Barry MJ, Fowler FJ, O'leary MP, et al. The American Urological Association Symptom Index for Benign Prostatic Hyperplasia. *J Urol* 2017;197(2):S189-S197

16 Avery K, Donovan J, Peters TJ, et al. ICIQ: A brief and robust measure for evaluating the symptoms and impact of urinary incontinence. *Neurourol Urodyn* 2004;23(4):322–330

17 Gosman GG, King WC, Schrope B, et al. Reproductive health of women electing bariatric surgery. *Fertil Steril* 2010;94(4):1426-1431

18 Feldman HA, Goldstein I, Hatzichristou DG, et al. Impotence and its medical and psychosocial correlates: Results of the Massachusetts Male Aging Study. *J Urol* 1994;151(1):54-61

19 Spitzer RL, Kroenke K, Williams JBW, et al. A brief measure for assessing generalized anxiety disorder: The GAD-7. *Arch Intern Med* 2006;166(10):1092-1097

20 Spitzer RL, Kroenke K, Williams JBW. Validation and utility of a self-report version of PRIME-MD: The PHQ Primary Care Study. *J Am Med Assoc* 1999;282(18):1737-1744

21 Reinert DF, Allen JP. The alcohol use disorders identification test (AUDIT): A review of recent research. *Alcohol Clin Exp Res* 2002;26(2):272–279

For peer review only

For peer review only - http://bmjopen.bmj.com/site/about/guidelines.xhtml

22 Ware JE, Kosinski M, Keller SD. A 12-Item Short-Form Health Survey: Construction of Scales and Preliminary Tests of Reliability and Validity. *Med Care* 1996;34(3):220-233

23 Herdman M, Gudex C, Lloyd A, et al. Development and preliminary testing of the new five-level version of EQ-5D (EQ-5D-5L). *Qual Life Res* 2011;20(10):1727-1736

24 Fermont JM, Blazeby JM, Rogers CA, et al. The EQ-5D-5L is a valid approach to measure health related quality of life in patients undergoing bariatric surgery. *PLoS One* 2017;12(12):e0189190

25 Kolotkin RL, Crosby RD. Psychometric evaluation of the impact of weight on quality of life-lite questionnaire (IWQOL-Lite) in a community sample. *Qual Life Res* 2002;11(2):157-171

26 Kolotkin RL, Crosby RD. The Impact of Weight on Quality of Life-Lite (IWQOL-Lite): User's Manual. Durham, NC: Obesity and Quality of Life Consulting 2002

27 Scheier MF, Carver CS, Bridges MW. Distinguishing Optimism From Neuroticism (and Trait Anxiety, Self-Mastery, and Self-Esteem): A Reevaluation of the Life Orientation Test. *J Pers Soc Psychol* 1994;67(6):1063-1078

28 Craig CL, Marshall AL, Sjöström M, et al. International physical activity questionnaire: 12-Country reliability and validity. *Med Sci Sports Exerc* 2003;35(8):1381-1395

29 Scottish Index of Multiple Deprivation 2016: introductory booklet. 2016. https://www.gov.scot/publications/scottish-index-multiple-deprivation-2016/ (accessed January 2020)

30 Arterburn DE, Courcoulas AP. Bariatric surgery for obesity and metabolic conditions in adults. *BMJ (Online)* 2014;349:g3961

31 Williamson K, Nimegeer A, Lean M. Rising prevalence of BMI ≥40 kg/m^2: A high-demand epidemic needing better documentation. *Obes Rev* 2020;21(4):e12986

For peer review only

32 le Roux CW, Chubb B, Nørtoft E, et al. Obesity and healthcare resource utilization: results from Clinical Practice Research Database (CPRD). *Obes Sci Pract* 2018;4(5):409-416

33 Di Angelantonio E, Bhupathiraju SN, Wormser D, et al. Body-mass index and all-cause mortality: individual-participant-data meta-analysis of 239 prospective studies in four continents. *Lancet* 2016;388(10046):776-786

34 Grieve E, Fenwick E, Yang HC, et al. The disproportionate economic burden associated with severe and complicated obesity: A systematic review. *Obes Rev* 2013;14(11):883-894

35 Kolotkin RL, Andersen JR. A systematic review of reviews: exploring the relationship between obesity, weight loss and health-related quality of life. *Clin Obes* 2017;7(5):273-289

36 Ul-Haq Z, Mackay DF, Fenwick E, et al. Meta-analysis of the association between body mass index and health-related quality of life among adults, assessed by the SF-36. *Obesity* 2013;21(3):E322-E327

37 Van Nunen AMA, Wouters EJM, Vingerhoets AJJM, et al. The health-related quality of life of obese persons seeking or not seeking surgical or non-surgical treatment: A meta-analysis. *Obes Surg* 2007;17(10):1357-1366

38 Radbruch L, Sabatowski R, Loick G, et al. Cognitive impairment and its influence on pain and symptom assessment in a palliative care unit: Development of a Minimal Documentation System. *Palliat Med* 2000;14(4):266-276

39 Brunner-La Rocca HP, Rickenbacher P, Muzzarelli S, et al. End-of-life preferences of elderly patients with chronic heart failure. *Eur Heart J* 2012;33(6):752-759

40 Iyasere O, Brown EA, Johansson L, et al. Quality of life with conservative care compared with assisted peritoneal dialysis and haemodialysis. *Clin Kidney J* 2018;12(2):262-268

41 Voll-Aanerud M, Eagan TML, Wentzel-Larsen T, et al. Respiratory symptoms, COPD severity,

For peer review only

and health related quality of life in a general population sample. *Respir Med* 2008;102(3):399-406

42 National Institute for Health and Clinical Excellence (NICE). *Weight management: lifestyle services for overweight or obese adults.* 2014. https://www.nice.org.uk/guidance/ph53 (accessed January 2020)

43 Welbourn R, Hollyman M, Kinsman R, et al. Bariatric Surgery Worldwide: Baseline Demographic Description and One-Year Outcomes from the Fourth IFSO Global Registry Report 2018. *Obes Surg* 2019;29(3):782-795

44 Avenell A, Robertson C, Skea Z, et al. Bariatric surgery, lifestyle interventions and orlistat for severe obesity: The rebalance mixed-methods systematic review and economic evaluation. *Health Technol Assess* 2018;22(68):1-246

45 Alsumali A, Eguale T, Bairdain S, et al. Cost-Effectiveness Analysis of Bariatric Surgery for Morbid Obesity. *Obes Surg* 2018;28(8):2203-2214

46 Maciejewski ML, Arterburn DE. Cost-effectiveness of Bariatric Surgery. *JAMA* 2013;310(7):742-743

47 Keating C, Neovius M, Sjöholm K, et al. Health-care costs over 15 years after bariatric surgery for patients with different baseline glucose status: Results from the Swedish Obese Subjects study. *Lancet Diabetes Endocrinol* 2015;3(11):855-865

48 Hoerger TJ, Zhang P, Segel JE, et al. Cost-effectiveness of bariatric surgery for severely obese adults with diabetes. *Diabetes Care* 2010;33(9):1933-1939

49 Borisenko O, Mann O, Duprée A. Cost-utility analysis of bariatric surgery compared with conventional medical management in Germany: A decision analytic modeling. *BMC Surg* 2017;17(1):87

For peer review only

50 Khan MA, Grinberg R, Johnson S, et al. Perioperative risk factors for 30-day mortality after bariatric surgery: Is functional status important? *Surg Endosc* 2013;27(5):1772-1777

51 Quilliot D, Sirveaux MA, Nomine-Criqui C, et al. Evaluation of risk factors for complications after bariatric surgery. *J Visc Surg* 2018;155(3):201-210

52 Livhits M, Mercado C, Yermilov I, et al. Preoperative predictors of weight loss following bariatric surgery: Systematic review. *Obes Surg* 2012;22:70-89

53 Nickel F, De La Garza JR, Werthmann FS, et al. Predictors of Risk and Success of Obesity Surgery. *Obes Facts* 2019;12(4):427-439

54 Parri A, Benaiges D, Schröder H, et al. Preoperative predictors of weight loss at 4 years following bariatric surgery. *Nutr Clin Pract* 2015;30(3):420-424

55 Jans A, Näslund I, Ottosson J, et al. Duration of type 2 diabetes and remission rates after bariatric surgery in Sweden 2007-2015: A registry-based cohort study. *PLoS Med* 2019;16(11):e1002985

56 Malone M, Alger-Mayer S, Polimeni JM. Health Related Quality of Life After Gastric Bypass Surgery. *Appl Res Qual Life* 2012;7:155-161

57 Pristed SG, Omar HK, Kroustrup JP. Association Between Fulfilment of Expectations and Health-related Quality of Life after Gastric Bypass. *Appl Res Qual Life* 2013;8(1):101-111

58 Julia C, Ciangura C, Capuron L, et al. Quality of life after Roux-en-Y gastric bypass and changes in body mass index and obesity-related comorbidities. *Diabetes Metab* 2013;39(2):148-154

59 Spencer EA, Appleby PN, Davey GK, et al. Validity of self-reported height and weight in 4808 EPIC–Oxford participants. *Public Health Nutr* 2002;5(4):561-565

60 Public Health Scotland Data and Intelligence. https://www.isdscotland.org (accessed January 2020)

For peer review only

FIGURE LEGEND

Figure 1. Screening, consent and follow-up. 548 patients were screened for inclusion in the SurgiCal Obesity Treatment Study. Of these 548, 103 were non-eligible (62 patients had undergone previous bariatric surgery and 1 patient lacked the necessary English language skills) and 40 were eligible but did not consent. 31 patients consented to only part 1 of the study (data linkage) while 413 consented to both part 1 and part 2 (completion of baseline PROMs). 249 baseline PROMs were available for analysis. PROMs, patient reported outcome measures.

For peer review only - http://bmjopen.bmj.com/site/about/guidelines.xhtml

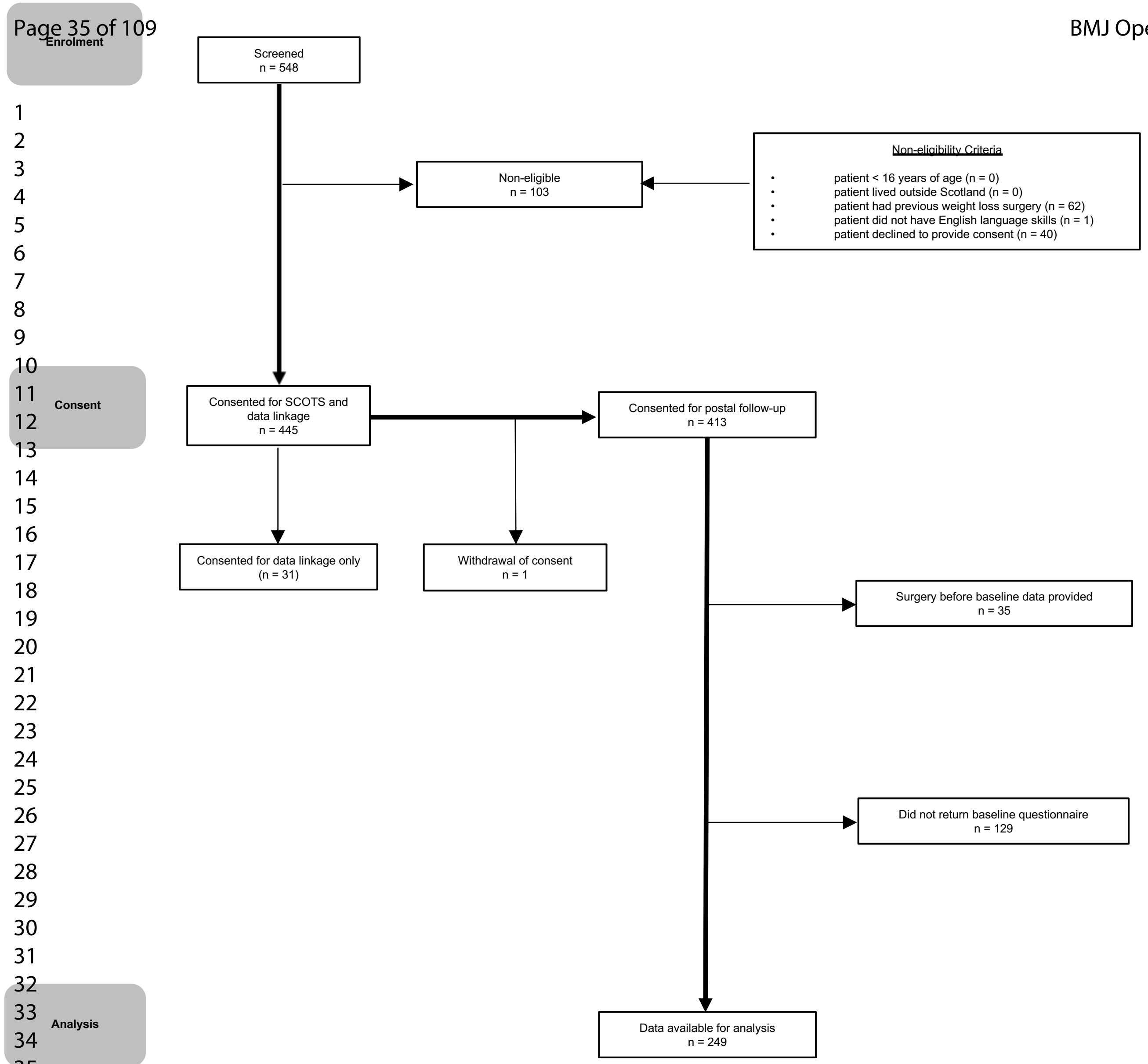
Enrolment
Screened
n = 548
Non-eligible
n = 103
Non-eligibility Criteria
• patient < 16 years of age (n = 0)
• patient lived outside Scotland (n = 0)
• patient had previous weight loss surgery (n = 62)
• patient did not have English language skills (n = 1)
• patient declined to provide consent (n = 40)
Consent
Consented for SCOTS and data linkage
n = 445
Consented for postal follow-up
n = 413
Consented for data linkage only
(n = 31)
Withdrawal of consent
n = 1
Surgery before baseline data provided
n = 35
Did not return baseline questionnaire
n = 129
Analysis
Data available for analysis
n = 249

Surgical Obesity Treatment Study

SCOTS

**Running title:**	**Surgical Obesity Treatment Study**
**Protocol Version:**	**1.6**
**Date:**	22/11/2017
**REC Reference Number:**	**13/WS/0005**
**ISRCTN/Clinical trial.gov:**	**47072588**
**Sponsor's Protocol Number:**	**GN21CA196**
**Sponsor:**	**NHS Greater Glasgow & Clyde**
**Funder:**	**National Institute for Health Research Health Technology Assessment Programme Ref 10/42/02**

Date	Protocol version	Amendment number & description
14/12/2012	1.0	Original
15/04/2013	1.1	Amendment 1: Minor changes to the format and presentation of the protocol
11/07/2013	1.2	Amendment 2: Correction of typographical errors in the protocol
30/06/2014	1.3	Amendment 3: Change to recruitment process for private hospitals involved in the study. Private hospitals will now become participant identification sites (and any NHS sites which in future are unable to support local recruitment), with participants being recruited and

For peer review only - http://bmjopen.bmj.com/site/about/guidelines.xhtml

Glasgow Clinical Trials Unit

		consented by a research team member (usually a research nurse).
03/06/2015	1.4	Amendment 4: Introduction of follow-up letter for patients recruited over the telephone by research nurses at the clinical research facility if completed consent form is not received within two weeks.
07/02/2017	1.5	Amendment 5; Revised study plan (including timetable & outputs). Patient recruitment stopping at a lower number and earlier than originally planned due to unforeseen recruitment issues. Amendment to clinical objectives and outcomes to reflect smaller sample size and shorter follow-up period. Reduction of post-operative questionnaire frequency to 2 and 3 years post-operatively only. Introduction of modest voucher incentives for PROMS completion.
22/11/2017	1.6	Amendment 6; Removal of annual blood test reminder. This procedure is completed as part of the routine clinical care by the patient's GP and therefore, it is not necessary for a reminder to be sent in addition for the SCOTS study.

This study will be performed according to the Research Governance Framework for Health and Community Care (Second edition, 2006) and WORLD MEDICAL ASSOCIATION DECLARATION OF HELSINKI Ethical Principles for Medical Research Involving Human Subjects 1964 (as amended).

For peer review only - http://bmjopen.bmj.com/site/about/guidelines.xhtml

Glasgow Clinical Trials Unit

CONTACTS

Chief Investigator

Dr Jennifer Logue MBChB MRCP MD FRCPath
CSO Clinician Scientist
Clinical Senior Lecturer and Honorary Consultant in Metabolic Medicine
Institute of Cardiovascular and Medical Sciences University of Glasgow
BHF Glasgow Cardiovascular Research Centre University of Glasgow
126 University Place, Room 208
University of Glasgow
Glasgow
G12 8TA

Tel: 0141 330 2569
Fax: 0141 330 6955
E-mail: Jennifer.Logue@Glasgow.ac.uk

Co-investigator

Professor Naveed Sattar
Professor of Metabolic Medicine
Cardiovascular and Medical Sciences
BHF Glasgow Cardiovascular Research Centre
RC214 Level C2
126 University Place, Room 208
University of Glasgow
Glasgow
G12 8TA
Tel: 0141 330 3419
Fax: 0141 330 6972
E-mail: Naveed.Sattar@glasgow.ac.uk

Dr Julie Bruce
Principal Research Fellow
Division of Health Sciences,
Warwick Medical School,
The University of Warwick,
Coventry,
CV4 7AL
Tel: 024 7615 1128
E-mail: julie.bruce@warwick.ac.uk

Mr Duff Bruce
Bariatric Surgeon
Ward 33
Aberdeen Royal Infirmary
Foresterhill
Aberdeen
AB25 2ZN
Tel: 01224 551275
Fax: 01224 443212
E-mail: duff.bruce@nhs.net

For peer review only

For peer review only - http://bmjopen.bmj.com/site/about/guidelines.xhtml

Glasgow Clinical Trials Unit

Dr Robert Lindsay
Reader Diabetes and Endocrinology
Cardiovascular and Medical Sciences
RC337 Level C3
BHF Glasgow Cardiovascular Research Centre
126 University Place, Room 208
University of Glasgow
Glasgow
G12 8TA
Tel: 0141 211 2502
Fax: 0141 330 6997
E-mail: Robert.Lindsay@glasgow.ac.uk

Professor Ian Ford
Professor of Biostatistics
R1122B Level 11
Robertson Centre for Biostatistics
Boyd Orr Building
Glasgow
G12 8QQ
Tel: 0141 330 4048
Fax: 0141 330 5094
E-mail: Ian.Ford@glasgow.ac.uk

Professor Andrew Briggs
Chair in Health Economics
(Health Economics and Health Technology Assessment) HEHTA
Institute of Health & Wellbeing
1 Lilybank Gardens
Glasgow
G12 8RZ
Tel: 0141 330 5017
E-mail: Andrew.Briggs@glasgow.ac.uk

Professor Mike Lean
Professor in Human Nutrition
R408 Level 4
Human Nutrition
Glasgow Royal Infirmary
84 Castle Street Glasgow
G40SF
Tel: 0141 211 4686
Fax: 0141 211 4844
E-mail: Mike.Lean@glasgow.ac.uk

Jane Munro
Research Lead
Cardiovascular and Medical Sciences
BHF Glasgow Cardiovascular Research Centre University of Glasgow
126 University Place, Room 208
University of Glasgow
Glasgow

For peer review only - http://bmjopen.bmj.com/site/about/guidelines.xhtml

Glasgow Clinical Trials Unit

G12 8TA
Tel: 0141 330 1670
E-mail: Jane.Munro@glasgow.ac.uk

Project Research Assistant

Mrs. Joanne O'Donnell
SCOTS Research Assistant
Cardiovascular and Medical Sciences
BHF Glasgow Cardiovascular Research Centre University of Glasgow
126 University Place, Room 208
University of Glasgow
Glasgow
G12 8TA
Tel: 0141 330 1670
Fax: 0141 330 5094
E-mail: Joanne.O'Donnell@glasgow.ac.uk

Study Statistician

Professor Ian Ford
Professor of Biostatistics
R1122B Level 11
Robertson Centre for Biostatistics
Boyd Orr Building
Glasgow
G12 8QQ
Tel: 0141 330 4048
Fax: 0141 330 5094
E-mail: Ian.Ford@glasgow.ac.uk

Data Centre

Alex McConnachie
Assistant Director of Biostatistics (Robertson Centre)
R1122E Level 11
Robertson Centre for Biostatistics
Boyd Orr Building
Glasgow
G12 8QQ
Tel: 01413304744
Fax: 0141 330 5094
E-mail: Alex.McConnachie@glasgow.ac.uk

Chair of the Study Steering Committee

Mr Richard Welbourn
Bariatric Surgeon
Musgrove Park Hospital
Taunton
TA1 5DA
Tel: 01823 343 565
Fax: 01823 353560
E-mail: richardwelbourn@gmail.com

For peer review only - http://bmjopen.bmj.com/site/about/guidelines.xhtml

Glasgow Clinical Trials Unit

Sponsor - NHS Greater Glasgow and Clyde R&D

Sponsor's representative

Dr Maureen Travers
Research Co-ordinator
NHS Greater Glasgow & Clyde
Research and Development Management Office
Tennent Institute
38 Church Street
Western Infirmary
Glasgow G11 6NT
Tel: 0141 211 6389
E-mail: maureen.travers@ggc.scot.nhs.uk

Funding Body

National Institute for Health Research (NIHR)

Funding Body Representative

Mrs Lesley Dodd
Programme Manager
NIHR Co-ordinating Centre for Health Technology Assessment (NETSCC, HTA)
University of Southampton
Alpha House
Enterprise Road
Southampton Science Park
Chilworth
Southampton
SO16 7NS
Tel: 02380 597558
Fax: 02380 595639
E-mail: L.Dodd@southampton.ac.uk

For peer review only

For peer review only - http://bmjopen.bmj.com/site/about/guidelines.xhtml

PROTOCOL APPROVAL

Surgical Obesity Treatment Study (SCOTS)

Chief Investigator

Dr Jennifer Logue
MBChB MRCP MD FRCPath CSO Clinician Scientist
Clinical Senior Lecturer and Honorary Consultant in Metabolic Medicine
Institute of Cardiovascular and Medical Sciences BHF Glasgow Cardiovascular Research Centre
University of Glasgow
126 University Place, Room 208
University of Glasgow
Glasgow
G12 8QQ

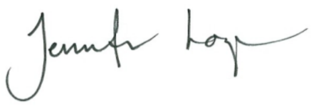

Signature:

Date: Thursday, 25 April 2019~~Wednesday, 29 November 2017~~

Sponsor's representative

Dr Maureen Travers
Research Co-ordinator
NHS Greater Glasgow & Clyde Research and Development Management Office
Tennent Institute
38 Church Street
Western Infirmary
Glasgow
G11 6NT

Signature:

Date:

For peer review only

For peer review only - http://bmjopen.bmj.com/site/about/guidelines.xhtml

Glasgow Clinical Trials Unit

TABLE OF CONTENTS

CONTACTS 32

PROTOCOL APPROVAL 76

TABLE OF CONTENTS 87

ABBREVIATIONS 1110

STUDY SYNOPSIS 1413

GLOSSARY OF TERMS 1615

STUDY FLOW CHART 1918

SCHEDULE OF ASSESSMENTS 2019

1 INTRODUCTION 2322

1.1 Background 2322

1.2 Rationale 2322

1.2.1 Definition of bariatric surgery 2422

1.2.2 Clinical Outcomes of weight loss surgery 2422

1.3 Study hypothesis 2524

2 STUDY OBJECTIVES 2524

2.1 Primary Objective 2524

2.2 Secondary Objectives 2524

2.3 Primary Outcome 2625

2.4 Secondary Outcomes 2625

3 STUDY DESIGN 2726

3.1 Overview of study design 2726

3.1.1 Part 1 Record Linkage 2928

3.1.2 Part 2 Patient Reported Outcomes (PROMS) Questionnaire 2928

3.1.3 Part 3: Registration for a database of bariatric patients for future research 2928

3.1.4 Service user involvement 3029

3.2 Study Population 3029

For peer review only

For peer review only - http://bmjopen.bmj.com/site/about/guidelines.xhtml

3.3 Reference populations 3029

3.4 Inclusion criteria 3130

3.5 Exclusion criteria..................... 3130

3.6 Identification of participants and consent 3130

3.7 Withdrawal of subjects..................... 3231

4 STUDY PROCEDURES 3332

4.1 Duration of participation 36

4.2 Study schedule..................... 37

4.2.1 Start of Study - Surgical team registration..................... 37

4.2.2 Visit 1 Routine care invitation visit 38

4.2.3 Visit 2 Recruitment visit..................... 38

4.2.4 SCOTS study team contact patient 42

4.2.5 Visit 3 Weight loss operation..................... 42

4.2.6 Visit 4 -Post operative follow up..................... 4243

4.2.7 Visit 5 - Re-operation (If applicable) 43

5 STATISTICS AND DATA ANALYSIS..................... 44

5.1 Statistical Analysis Plan..................... 44

5.2 Software for statistical analysis 44

5.3 Sample size 44

5.4 Management and delivery 4645

6 STUDY CLOSURE 4746

7 Data Handling..................... 4746

7.1.1 Surgeon registration data 4746

7.1.2 Patient recruitment data 4746

7.1.3 Surgical data collection..................... 4847

7.1.4 Patient Reported Outcomes 4847

7.1.5 Routine clinical information 4847

For peer review only

7.1.6 Re-operation .... 48~~47~~

7.1.7 Record Retention .... 49~~47~~

7.1.8 Access to data .... 49~~48~~

8 SCOTS STUDY MANAGEMENT .... 50~~49~~

8.1 Study Sponsor .... 50~~49~~

8.2 SCOTS Study Team .... 50~~49~~

8.3 SCOTS Principal investigators .... 50~~49~~

8.4 Routine management of study: SCOTS Management Group (SMG) .... 50~~49~~

8.5 SCOTS Steering Committee (SSC) .... 51~~50~~

9 STUDY MONITORING/AUDITING .... 52~~51~~

10 PROTOCOL AMENDMENTS .... 52~~51~~

11 ETHICAL CONSIDERATIONS .... 52~~51~~

11.1 Ethical conduct of the study .... 52~~51~~

11.2 Informed consent .... 52~~51~~

12 INSURANCE AND INDEMNITY .... 53~~52~~

13 FUNDING .... 53~~52~~

14 ANNUAL REPORTS .... 53~~52~~

15 DISSEMINATION OF FINDINGS .... 54~~53~~

16 REFERENCES .... 55~~54~~

17 Appendix E: Summary of Patient Reported Outcome Questions .... 59~~58~~

For peer review only

For peer review only - http://bmjopen.bmj.com/site/about/guidelines.xhtml

ABBREVIATIONS

BHF GCRC	*British Heart Foundation, Glasgow Cardiovascular Research Centre*
BMI	*Body Mass Index*
BP	*Blood Pressure*
BPD-DS	*BilioPancreatic Diversion with Duodenal Switch*
CHD	*Coronary Heart Disease*
CHI	*Community Health Index*
CHF	*Congestive Heart Failure*
CI	*Chief Investigator*
CKD	*Chronic Kidney Disease*
CNORIS	*Clinical Negligence and Other Risks Indemnity Scheme*
CRF	*Case Report Form*
CSO	*Chief Scientist Office*
CTU	*Clinical Trials Unit*
CVD	*Cardiovascular Disease*
DM	*Diabetes Mellitus*
DM(E)C	*Data Monitoring (Ethics) Committee*
DOH	*Department of Health*
DS	*Duodenal Switch*
e-CRF	*Electronic Case Report Form*
EQ-5D	*EuroQol -5D*
GAD-7	*Generalised Anxiety Disorder – 7*
GCP	*Good Clinical Practice*
GCP	*Good Clinical Practice*
GCTU	*Glasgow Clinical Trials Unit*
GP	*General Practitioner*

For peer review only

For peer review only - http://bmjopen.bmj.com/site/about/guidelines.xhtml

Glasgow Clinical Trials Unit

GRDS	*Gastric Reduction Duodenal Switch*
GROS	*General Register Office for Scotland*
HbA1c	*Glycated Haemoglobin A1c*
HDL	*High Density Lipoprotein*
HR-QL	*Health Related- Quality of Life*
HTA	*Health Technology Assessment*
ICMS	*Institute of Cardiovascular and Medicinal Sciences*
IPAQ	*International Physical Activity Questionnaire*
IPSS	*International Prostate Symptom Score*
IRAS	*Integrated Research Application System*
ISD	*Information and Services Division (NHS)*
ISRCTN	*International Standard Randomised Controlled Trial Number*
IWQOL-Lite	*Impact of Weight on Quality of Life Lite Questionnaire*
LOT	*Life orientation Test*
MG	*Management Group*
MGM	*Management Group Meeting*
MI	*Myocardial Infarction*
NBSR	*National Bariatric Surgery Register*
NHS	*National Health Service*
NICE	*National Institute for Health and Clinical Excellence*
NIHR	*National Institute for Health Research*
NIHR CSP	*National Institute for Health Research Coordinated System for Gaining NHS Permission*
PHQ-9	*Patient Health Questionnaire - 9*
PI	*Principal Investigator*
PROMS	*Patient Reported Outcome Measures*
QALY	*Quality Adjusted Life Year*

For peer review only

For peer review only - http://bmjopen.bmj.com/site/about/guidelines.xhtml

Glasgow Clinical Trials Unit

QOL	*Quality Of Life*
R&D	*Research and Development*
RCB	*Robertson Centre for Biostatistics*
REC	*Research Ethics Committee*
SCOTS	*SurgiCal Obesity Treatment Study*
SCR	*Scottish Cancer Registry*
SCI-DC	*Scottish Care Information – Diabetes Collaboration*
SDRN	*Scottish Diabetes Research Network*
SDRN EG	*Scottish Diabetes Research Network Epidemiology Group*
SF-12	*Short Form -12*
SIGN	*Scottish Intercollegiate Guidelines Network*
SMG	*SCOTS Management Group*
SMR	*Scottish Morbidity Record*
SOP	*Standard Operating Procedure*
SSC	*SCOTS Steering Committee*
SSI	*Surgical Site Infection*

For peer review only

For peer review only - http://bmjopen.bmj.com/site/about/guidelines.xhtml

Glasgow Clinical Trials Unit

STUDY SYNOPSIS

Title of Study:	**Surgical Obesity Treatment Study**; SCOTS
Study Centre:	University of Glasgow
Duration of Study:	03/01/2012 – 30/06/2026
Primary Objective:	The SurgiCal Obesity Treatment Study (SCOTS) will establish the clinical outcomes and adverse events of different bariatric surgical procedures (taking into consideration the different care pathways), their impact on quality of life and nutritional status, and the effect on co-morbidities in a cohort of 450 patients.
Secondary Objectives:	1. The physical and mental health and social burden of severe obesity.<br>2. Incidence of acute and chronic postoperative complications. Acute complications, defined as up to three months post-surgery, will include surgical site infection; chronic complications will include revisional surgery, plastic surgery and chronic pain, for different bariatric surgical procedures<br>3. The effect of surgical experience and the pre and post-operative care pathway on complication rates and weight loss, for different bariatric surgical procedures.<br>4. The effect of the pre-surgical pathway and criteria on bariatric surgery patient selection.<br>5. Change in health related quality of life, anxiety and depression over time pre- and post-operatively for a mean of 3 years from date of bariatric surgery.<br>6. The weight status pre and post-operatively for 3 years after bariatric surgery.<br>7. The glycaemic control, lipids, blood pressure, medication prescription, and rate of diabetes complications (microalbuminuria and renal disease, and retinopathy) in those that have pre-existing diabetes or develop diabetes for 3 years follow up since bariatric surgery.<br>8. Changes in socio-economic factors (employment, benefit receipt, sick leave, and health care use) for 3 years since bariatric surgery.
Primary Endpoint:	Change in weight from pre-bariatric surgery weight

For peer review only - http://bmjopen.bmj.com/site/about/guidelines.xhtml

For peer review only

Rationale:	The efficacy of bariatric surgery for large scale, long term weight loss is well established. However, many questions remain over the medium term benefits of that weight loss for health, particularly when accounting for the potential complications of surgery.
Methodology:	Observational cohort study/ registry
Sample Size:	450
Screening:	All new bariatric surgery patients
Registration/Randomisation:	Not applicable
Main Inclusion Criteria:	• Patients 16 years or over undergoing their first bariatric surgery in NHS hospitals or private practice in Scotland.<br>• Capacity to consent<br>• Resident in Scotland<br>• Written informed consent
Main Exclusion Criteria:	• Does not meet inclusion criteria<br>• Patients who have had previous weight loss surgery (at time of potential recruitment are undergoing a repeat procedure)<br>• No written informed consent<br>• Patients under 16 years<br>• Patients who do not have capacity to consent for themselves<br>• Weight loss surgery performed at a site outside Scotland<br>• Patients who live outside Scotland<br>• Patients who do not have strong enough English language skills to understand the patient information leaflet without assistance will be excluded from Part 2 of the study.
Product, Dose, Modes of Administration:	Web based or paper questionnaire to be completed<br>1 month pre-surgery<br>2 years post surgery<br>3 years post surgery
Duration of Treatment:	Observational study so protocol will be applied until a participant has been in study for 3 years or until December 2020 whichever comes first. If funding available record linkage will continue throughout patient's life.
Statistical Analysis:	Detailed statistical analysis plans will be developed by the Robertson Centre for Biostatistics to address each of the objectives described above.

For peer review only - http://bmjopen.bmj.com/site/about/guidelines.xhtml

GLOSSARY OF TERMS

Bariatric Surgical Procedures: *A bariatric surgical procedure is defined as a surgical intervention which has the primary purpose of large scale weight loss in a patient who is obese. Bariatric Surgical procedures eligible for inclusion in SCOTS are:* ***duodenal switch, gastric banding, gastric bypass*** *and* ***sleeve gastrectomy****. The inclusion of emerging techniques in this definition will be decided by the steering committee.*

Biliopancreatic diversion with duodenal switch (BPD-DS): *see* **Duodenal Switch***:*

Body mass index (BMI) *is a simple index of weight-for-height that is commonly used to classify* ***overweight*** *and* ***obesity*** *in adults. It is defined as a person's weight in kilograms divided by the square of his height in meters (kg/m^2). BMI provides the most useful population-level measure of overweight and obesity as it is the same for both sexes and for all ages of adults. However, it should be considered a rough guide because it may not correspond to the same degree of fatness in different individuals.*

Co-morbidity: *is either the presence of one or more disorders (or diseases) in addition to a primary disease or disorder, or the effect of such additional disorders or diseases.*

Duodenal Switch: *(DS) is a weight loss surgery procedure that is composed of a restrictive and a malbsorbative aspect. The restrictive portion of the surgery involves removing approximately 70% of the stomach along the greater curvature. The malabsorptive portion of the surgery reroutes a lengthy portion of the small intestine, creating two separate pathways and one common channel. Duodenal switch (DS) is also known as biliopancreatic diversion with duodenal switch (BPD-DS) or gastric reduction duodenal switch (GRDS).*

Endoluminal barrier: *is a liner that fits inside a section of the intestine, creating a barrier between the intestinal wall and the food you eat. It helps patients to lose weight by preventing food from coming into contact with the intestinal wall thus delaying digestion, which will alter the activation of hormonal signals that originate in the intestine.*

Gastric balloon: *An inflatable device implanted in the stomach as an adjunct to therapy of morbid obesity. For the purposes of the SCOTS study a balloon treatment is not considered to be bariatric surgery. Use of a balloon before or after surgery will be recorded as a pre-operative treatment but a balloon alone would not meet the criteria for inclusion in SCOTS.*

Gastric band: *adjustable gastric banding surgery, is a restrictive weight loss surgery that works by decreasing the amount of food that is able to be consumed before feeling full. During gastric banding surgery, a firm silicone band with an inner inflatable ring is placed around the stomach to slow the passage of food from the upper portion of the stomach to the lower portion*

Gastric bypass: *a surgical bypass operation that typically involves reducing the size of the stomach and reconnecting the smaller stomach to bypass the first portion of the small*

For peer review only - http://bmjopen.bmj.com/site/about/guidelines.xhtml

intestine so as to restrict food intake and reduce caloric absorption in cases of severe obesity. This includes Gastric bypass, Roux en-Y (proximal), Gastric bypass, Roux en-Y (distal) and Mini-gastric bypass" (MGB)

Gastric reduction duodenal switch (GRDS): see duodenal switch

Glycaemic control*: is a medical term referring to the typical levels of blood sugar (glucose) in a person with diabetes mellitus. Much evidence suggests that many of the long-term complications of diabetes, especially the microvascular complications, result from many years of hyperglycemia (elevated levels of glucose in the blood).*

Hemoglobin A1c (HbA1c): *Blood sugar levels fluctuate throughout the day and glucose records are imperfect indicators of these changes, the percentage of haemoglobin which is glycosylated is used as a proxy measure of long-term glycemic control in research trials and clinical care of people with diabetes. This test, the hemoglobin A1c or glycosylated hemoglobin reflects average glucoses over the preceding 2–3 months. From 1 October 2011 the way in which HbA1c results are expressed in the UK has changed. Results are now reported in the IFCC reference method of mmol/L, rather than the DCCT units as a percentage. The equivalent of the current HbA1c targets of 6.5% and 7.5% are therefore 48mmol/mol and 58mmol/mol in the new units for diabetic patients, with the non-diabetic reference range of 4.0% to 6.0% being 20mmol/L to 42mmol/L.*

HDL cholesterol: *High density lipoprotein cholesterol. Lipoproteins, which are combinations of fats (lipids) and proteins, are the form in which lipids are transported in the blood. HDLs transport cholesterol from the tissues of the body to the liver, so the cholesterol can be eliminated in the bile. HDL cholesterol is therefore considered the 'good' cholesterol: The higher the HDL cholesterol level, the lower the risk of coronary artery disease. The average man has an HDL cholesterol level of 40 to 50 mg/dL. In the average woman, HDL levels range from 50 to 60 mg/dL.*

Health Technology Assessment*: is a multidisciplinary process that summarises information about the medical, social, economic and ethical issues related to the use of a health technology in a systematic, transparent, unbiased, robust manner. Its aim is to inform the formulation of safe, effective, health policies that are patient focused and seek to achieve best value.*

Hypertriglycerideamia*: denotes high (''hyper-'') blood levels (''-aemia'') of triglycerides, the most abundant fatty molecule in most organisms.*

Laparoscopic gastric banding*: Lap Band surgery, or adjustable gastric banding surgery, is a restrictive weight loss surgery that works by decreasing the amount of food that is able to be consumed before feeling full. During gastric banding surgery, a firm silicone band with an inner inflatable ring is placed around the stomach to slow the passage of food from the upper portion of the stomach to the lower portion. Most surgeries are performed laparoscopically, which allows the surgeon to work using long instruments placed in the body via incisions a few centimetres long.*

For peer review only - http://bmjopen.bmj.com/site/about/guidelines.xhtml

***Laparoscopy**: is a type of surgical procedure that allows a surgeon to access the inside of the abdomen and pelvis without having to make large incisions in the skin. It is also known as keyhole surgery. Laparoscopy is minimally invasive. This is made possible with an instrument called a laparoscope.*

***Morbidity**: Morbidity is an incidence of ill health.*

***Mortality**: is incidence of death in a population.*

***NHS patient**: A patient who receives free healthcare through the National Health Service*

***Nutritional Status**: The state of a person's health in terms of the nutrients stored in his or her body.*

***Obesity**: Obesity is a term used to describe somebody who is very overweight with a high degree of body fat. There are a number of ways a person's weight can be assessed. The most widely used method is the body mass index.*

***Overweight**: a BMI of between 25 and 29, would be considered overweight*

***Obese**: a BMI of 30 or above would be considered obese*

***Private sector**: healthcare and medicine provided by entities other than the government and paid for by the patient or a private insurance company.*

***Prospective cohort study**: a study designed to determine the relationship between a condition and a characteristic shared by some members of a group. The population selected is healthy at the beginning of the study. Members share a particular characteristic. The study follows the population groups over a long period, noting the rate at which a condition occurs in the population.*

***Revisional surgery**: a bariatric surgical procedure on a patient who has already had bariatric surgery*

***Roux-en-Y gastric bypass:** a small part of the stomach is used to create a new stomach pouch, roughly the size of an egg. The smaller stomach is connected directly to the middle portion of the small intestine (jejunum), bypassing the rest of the stomach and the upper portion of the small intestine (duodenum).*

***Sleeve gastrectomy**: is a surgical weight-loss procedure in which the stomach is reduced to about 25% of its original size, by surgical removal of a large portion of the stomach, following the major curve. The open edges are then attached together (often with surgical staples) to form a sleeve or tube with a banana shape. The procedure permanently reduces the size of the stomach. The procedure is performed laparoscopically and is not reversible.*

For peer review only - http://bmjopen.bmj.com/site/about/guidelines.xhtml

Glasgow Clinical Trials Unit

STUDY FLOW CHART

FIGURE 0-1 THIS STUDY FLOW CHART SUMMARISES THE PATIENT JOURNEY TRHOUGHOUT THE STUDY

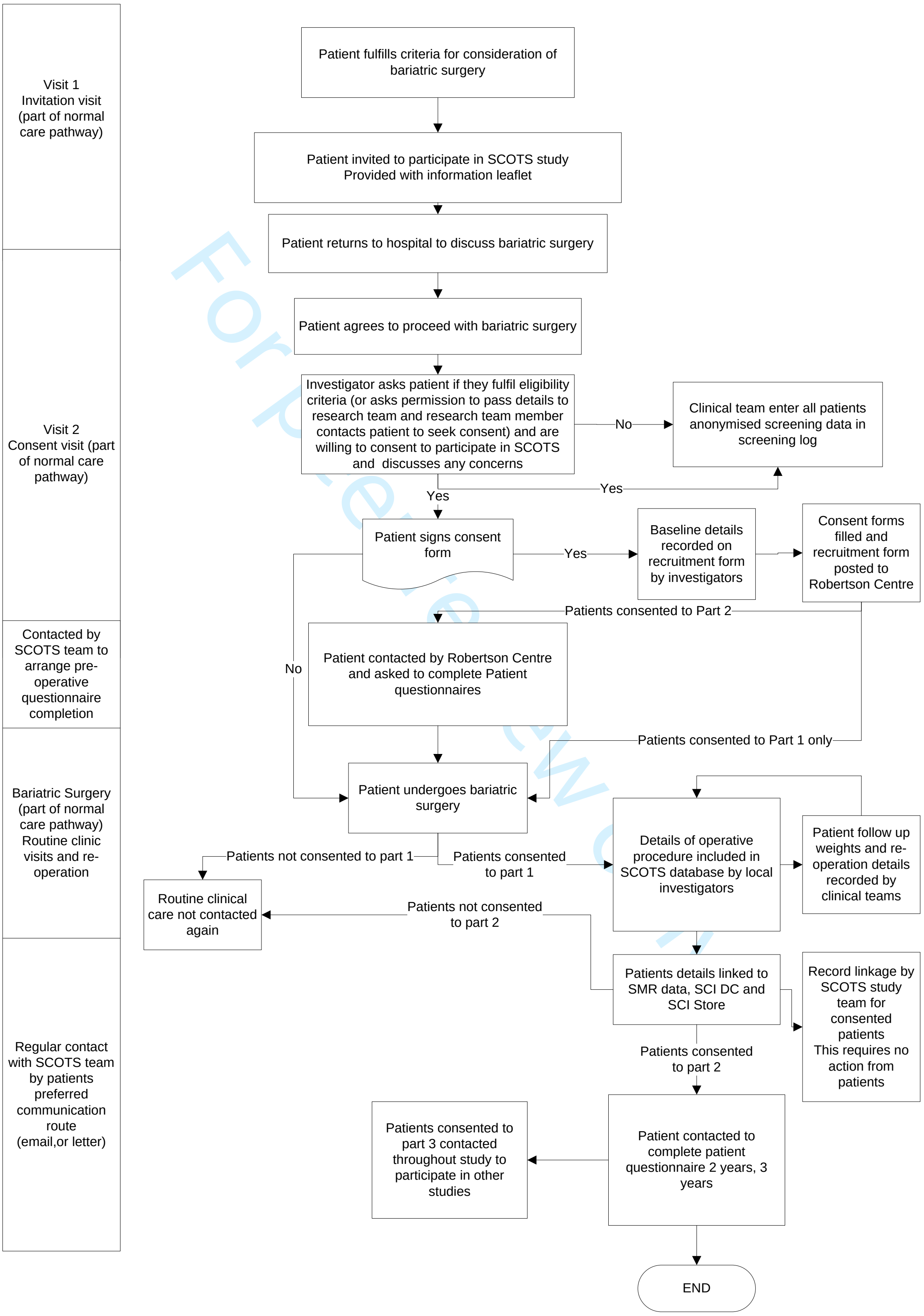

For peer review only - http://bmjopen.bmj.com/site/about/guidelines.xhtml

SCHEDULE OF ASSESSMENTS

TABLE 0-1: THIS SCHEDULE SHOWS EACH EVENT THAT INVOLVES THE PATIENT AND STUDY TEAM.

	Pre-surgery					Post surgery SCOTS Questionnaires	
Study Procedure	Pre-surgery Visit 1 1 year to six weeks before surgery	Pre-surgery Visit 2 1 year to six weeks before surgery	Pre-surgery Contact1	Pre-surgery Contact2	Surgery T0	2yr	3yr
Patients agree to proceed to bariatric surgery with clinical team	√						
Review Inclusion/Exclusion Criteria	√[a]						
Patients provided with patient information sheet	√						

For peer review only - http://bmjopen.bmj.com/site/about/guidelines.xhtml

Glasgow Clinical Trials Unit

Patient data entered onto screening log for SCOTS		√					
Patient return for pre-surgery clinic		√					
Clinical team ask permission from patients to pass details to research team who will contact patient (if not recruiting in local site)		(√)					
Obtain Informed Consent to participate in SCOTS study		√					
Patient baseline and contact details entered on registration form and sent to Robertson Centre for Biostatistics		√					
Consent form passed filed in Site File, copy given to patient and copy sent to RCB		√					
Contacted by SCOTS team to ask to complete pre-surgery questionnaire			√				

For peer review only

For peer review only - http://bmjopen.bmj.com/site/about/guidelines.xhtml

Glasgow Clinical Trials Unit

Patient completes pre-surgery questionnaire				√			
Patient undergoes bariatric surgery					√		
Clinical teams record operative details for patient post surgery					√		
Clinical teams record weight and re-operation details						√	√
Patients contacted to remind them to complete post surgery questionnaire						√	√
Part 3 consented patients may be invited to participate in other studies	Ongoing throughout study						
Record linkage to SCI-Store, SCI – DC, SMR (ISD), GRO throughout study	Ongoing throughout study, no interaction with patient unless to clarify if admission was a complication of bariatric surgery.						

For peer review only

For peer review only - http://bmjopen.bmj.com/site/about/guidelines.xhtml

1 INTRODUCTION

1.1 Background

SCOTS is a prospective cohort study commissioned by the Department of Health (DoH) National Institute of Health Research (NIHR) which will follow bariatric surgery patients for 3 years after their surgery. The NIHR commissioning brief for this study stated: “Obesity is a growing problem in the United Kingdom, with a growing number of people among the morbidly obese. Concomitantly, there is an increase in requests for bariatric surgery but insufficient evidence of long term effectiveness and safety of these procedures. There are existing registries of bariatric surgery, but these suffer from problems of collected data, completeness of follow- up, or data availability for secondary analysis. There is a need for a long term study of bariatric surgery, so that the outcomes and complications of different procedures, their impact on quality of life and nutritional status, and the effect on co-morbidities can be monitored in both the short and the long term.” A recent Health Technology Assessment (HTA) (1) concluded that “Bariatric surgery is a more clinically effective intervention for treating obesity compared to non-surgical options” however they qualified this with “Uncertainties remain over the long-term outcomes of bariatric surgical interventions, particularly their impact on weight-related health problems and patient quality of life. Further studies will be required”. This study was awarded NIHR HTA funding to address some of the uncertainties around the clinical effectiveness of bariatric surgery in the longer term. The original plan included a 10 year follow up period, however, due to unforeseen recruitment issues (reductions in both NHS & private surgical numbers) the study & statistical plans were reviewed. Following discussions with the NIHR it was agreed that many objectives could still be met from the number of patients currently recruited over a shorter period of follow up (3 years). The protocol has been updated to reflect these changes. Clearly some patients will have completed the 1 month, 6 month and 1 year post-operative questionnaire already. Where appropriate, these data will be used to supplement the year 2 and 3 questionnaires and the health record data in order to deliver the study objectives.

1.2 Rationale

The efficacy of bariatric surgery for large scale, long term weight loss is well established (2). However, many questions remain over the medium term benefits of that weight loss for health, particularly when accounting for the potential complications of surgery. Given that the proportions of the population who are obese is increasingly rapidly, further information is required as to who benefits most from bariatric surgery.

For peer review only - http://bmjopen.bmj.com/site/about/guidelines.xhtml

1.2.1 Definition of bariatric surgery

A bariatric surgical procedure is defined as a surgical intervention which has the primary purpose of large scale weight loss in a patient who is obese. Currently that includes gastric bypass, sleeve gastrectomy and laparoscopic gastric banding. The inclusion of emerging techniques in this definition will be decided by the SCOTS Steering Committee (SCC).

1.2.2 Clinical Outcomes of weight loss surgery

There are some pre-existing data on clinical outcomes with 10 year mortality rates being the best known. Overall mortality was found to be 29-40% lower in patients receiving bariatric surgery compared to Body Mass Index (BMI)-matched subjects not receiving surgery (3;4). Although impressive, these figures are from studies which initially recruited in the 1980s and early 1990s and further work is required to update the mortality rate in the current population, now that primary prevention measures for cardiovascular disease, such as statins and ACE-inhibitors, have lowered the risk of premature mortality in at risk subjects, including those who are obese or have diabetes.

Diabetes improvement post bariatric surgery has also been of interest. Dixon et al conducted a randomised controlled trial of laparoscopic gastric banding in patients with newly diagnosed type 2 diabetes resulted in a 73% remission rate after 2 years (5). Similar results have been shown for gastric bypass using meta-analysis of heterogeneous, mainly retrospective, cohort studies (6). Whether remission is maintained beyond the first few years post surgery is unknown. However, the main outcomes of interest are the longer term effects on the development of diabetes complications such as retinopathy, nephropathy and cardiovascular disease and those are not established.

The effect of bariatric surgery on cardiovascular outcomes has not been ascertained. It is known that HDL cholesterol is less likely to be low and hypertriglycerideamia is less common after surgery (7), as expected by a procedure which will reduce intra-abdominal adiposity and thus insulin resistance, but a reduction in cardiovascular events has not been shown. Hypertension, which would be presumed to decrease, in fact shows no difference 8 years post surgery, once the initial antihypertensive effects of acute weight loss in the first few years post-surgery are accounted for (8). Many have used cardiovascular risk factors measured during the acute weight loss phase, applied a population based cardiovascular risk score and stated a reduction in cardiovascular risk; this is of course a flawed use of such a score based on temporarily reduced cardiovascular risk factors. So, while the general population and the majority of physicians may assume that weight loss with bariatric surgery reduces the risk of a cardiovascular event, this is currently unknown.

What is established is that health related quality of life (HR-QoL) is increased compared to pre-operative levels (9). When patients who decide to proceed to surgery are compared to those who opt for non-surgical treatment only, the baseline HR-QoL is far lower in those

For peer review only - http://bmjopen.bmj.com/site/about/guidelines.xhtml

who select surgery, showing at least a perception of reduced quality of life in these individuals (10). To what extent the improvements seen in HR-QoL correlate with weight loss, complications and clinical outcomes is not known.

The complication rates of bariatric surgery are poorly reported in the literature, leading to polarised views on their true incidence. Mortality rates in the first year post surgery have been reported as being similar to those of BMI-matched controls (11); however, the same results may not be found today due to the decreased mortality rates as a result of primary preventative. There is little report of specific wound complications and re-operation rates, with a general belief that the low rates reported by the few studies releasing this data would not be replicated in routine practice.

1.3 Study hypothesis

This study is a registry of bariatric surgery and is therefore the study design is based on objectives but not hypotheses.

2 STUDY OBJECTIVES

SCOTS is a prospective observational cohort study of 450 patients undergoing bariatric surgery in Scotland which will be used to assess medium term outcomes and complications of bariatric surgery. This is in order to identify risks and complications of bariatric surgery and develop an evidence base to support clinical teams in building a patient centred, safe, effective and efficient bariatric surgery service.

2.1 Primary Objective

The SurgiCal Obesity Treatment Study (SCOTS) will establish the clinical outcomes and adverse events of different bariatric surgical procedures (taking in to consideration different care pathways), their impact on quality of life and nutritional status, and the effect on co-morbidities in a cohort of 450 patients.

2.2 Secondary Objectives

To establish in a cohort of obese patients who are undergoing bariatric surgery:

1. The physical and mental health and social burden of severe obesity.
2. Incidence of acute and chronic postoperative complications. Acute complications, defined as up to three months post-surgery, will include surgical site infection; chronic complications will include revisional surgery, plastic surgery and chronic pain, for different bariatric surgical procedures.

For peer review only - http://bmjopen.bmj.com/site/about/guidelines.xhtml

3. The effect of surgical experience and the pre and post-operative care pathway on complication rates and weight loss, for different bariatric surgical procedures.
4. The effect of the pre-surgical pathway and criteria on bariatric surgery patient selection. Change in health related quality of life, anxiety and depression over time pre- and post-operatively for a mean of 3 years from date of bariatric surgery.
5. The weight status pre and post-operatively for 3 years after bariatric surgery.
6. The glycaemic control, lipids, blood pressure, medication prescription, and rate of diabetes complications (microalbuminuria and renal disease, and retinopathy) in those that have pre-existing diabetes or develop diabetes for 3 years follow up since bariatric surgery.
7. Changes in socio-economic factors (employment, benefit receipt, sick leave, and health care use) for 3 years since bariatric surgery.

All the above objectives are due to be achieved by December 2020 (Start Jan 2012, 2 years development, 3 years recruitment finishing March 2017, mean 3 years post-operative follow-up finishing December 2020).

2.3 Primary Outcome

Change in weight from pre-bariatric surgery weight.

2.4 Secondary Outcomes

1. Incidence of physical and psychological comorbidity in a population seeking bariatric surgery

In the 3 years following bariatric surgery:

2. Change in weight/BMI from pre-bariatric surgery weight
3. Rate of incident type 2 diabetes
4. Change in incidence of depression and anxiety compared to baseline level pre-operatively
5. Incidence of complications immediately post-operatively and the need for readmission for revisional procedures
6. Change in health-related quality of life compared to baseline level pre-operatively
7. Change in glycaemic control, cardiovascular risk factors, CKD, retinopathy and medications prescribed in patients with diabetes compared to equally obese patient with diabetes who did not have bariatric surgery
8. Cost of the procedure and follow-up

Outcomes, including their source and frequency of measurement, are summarised in table 1.

For peer review only - http://bmjopen.bmj.com/site/about/guidelines.xhtml

3 STUDY DESIGN

3.1 Overview of study design

This 3 year prospective cohort study will collect data on the medium term outcomes of bariatric surgery in a minimum of 400 individuals. The study will be performed according to the Research Governance Framework for Health and Community Care (Second edition, 2006) (12). The information required to meet the objectives will be collected by a combination of sources including: bariatric surgery specific information entered by clinical teams; data linkage to routine clinical data and patient reported outcome measure questionnaires (PROMS). The outcomes and the data source are described in detail in Table 3-1~~Table 3-1~~.

TABLE 3-1: PRIMARY AND SECONDARY OBJECTIVES OF SCOTS DESCRIBING SOURCES OF OUTCOME DATA

	Outcome	Data source	Frequency
1	Incidence of physical and psychological comorbidity in a population seeking bariatric surgery.	SCI-DC Scottish National dataset, eDRIS at National Service Scotland, Patient reported questionnaires.	Continuous throughout study
2.1	Change in weight/BMI from pre-bariatric surgery	Clinical teams	At recruitment, annual check up
2.2	Rate of incidence of type 2 diabetes	SCI-DC Scottish National dataset	Continuous throughout study
2.3	Change in incidence of depression and anxiety compared to baseline levels pre-operatively	eDRIS at National Service Scotland, Patient reported questionnaires	Pre-operatively, ,2,3 years post operatively
2.4	Incidence of complications immediately post-operatively and the need for revisional procedures	Patient reported questionnaires, surgical teams	Pre-operatively, 2, 3 years post operatively, at time of re-operation.
2.5	Change in health related quality of life compared to baseline	Patient reported questionnaires	Pre-operatively, 2,3years post operatively
2.6	Change in glycaemic control, cardiovascular risk factors, chronic kidney disease (CKD), retinopathy, medications in patients with	SCI-DC Scottish National dataset	Pre-operatively, 2, 3 years post operatively, at time of re-operation.

For peer review only - http://bmjopen.bmj.com/site/about/guidelines.xhtml

	diabetes compared with non-obese cohort		
2.7	Cost of the procedure and follow-up (to inform cost effectiveness analysis)	Clinical teams, e-prescribing record linkage, patient reported outcomes	At time of patients operation, continuous linkage throughout the study

This protocol describes the objectives of the study, the sources of data collection for each objective, and the input required from patients and clinical teams throughout the study. A data collection portal will be developed for the study with secure logins for clinical teams and participating patients. A detailed user manual will be developed for the operation of this system will be produced by the Robertson Centre for Biostatistics - University of Glasgow.

A SCOTS web based portal will be developed which will present information on the study to the public and other researchers. The portal will contain secure logins to the Robertson Centre databases which will allow clinical teams to enter data. Clinical teams will be asked to register for a user ID and password. These will be issued once the team have undergone the necessary training for their delegated role. Everyone who has a user ID and login will be a member of the SCOTS investigators group.

The SCOTS portal will include an area which will allow patients who have agreed to complete the patient questionnaires to login securely and complete the patient reported outcome questionnaires. If patients prefer to complete the questionnaires on paper the data will be entered by staff at the Robertson Centre into the database. Patients will be kept up to date with progress of the study via updates on the study website. Patients will be sent reminders through their preferred route of communication to let them know when they are due to complete a questionnaire.

Patient identifiable information will be stored separately from the clinical outcome data. The information will be linked using a database code. Within the Robertson Centre individuals who can access patient information to send reminder letters will not have access to any clinical information. Any clinical information to be analysed will have all patient contact details remove. Production of any reports will follow the NHS Information and Statistics division (ISD) Statistical Disclosure Control protocol (13) to ensure that patient confidentially is maintained, (this is a requirement of the record linkage arrangement with ISD).

SCOTS has three distinct parts. All adult patients who are having weight loss surgery in Scotland will be invited to participate in three parts of the study. These parts are described in detail below.

For peer review only

For peer review only - http://bmjopen.bmj.com/site/about/guidelines.xhtml

3.1.1 Part 1 Record Linkage

The study will collect data by record linkage to patients' clinical outcomes. Information will be recorded by the clinical team on the operation they have had. They will be followed using their medical records until at least December 2020 and if funding is available for the rest of their life. This will include all hospital admissions, blood test results, diagnostic test results, diabetes complications, medications they are prescribed, pregnancy, psychiatry and mortality. This part of the study will observe patients care. The study will not alter their planned care in any way. No additional tests or treatments will be given to patients who consent to be part of the study. (This will fulfil the primary objective and objectives 1, 2, 3 and 4). If patients do not consent to participate in this part of the study they will not be asked to participate in Part 2 or Part 3.

Each record linkage requires approvals to access data including a favourable ethical opinion. These will be sought by the SCOTS Project team. A separate procedure for each data linkage will be developed by the Robertson Centre for Biostatistics. Patient identifiable information is required to identify study participants. The information required is patient CHI number, family name, given name, patient date of birth, gender, postcode, consent data and unique study identifier.

3.1.2 Part 2 Patient Reported Outcomes (PROMS) Questionnaire

We will ask patients if they are willing to complete a questionnaire which will include questions on quality of life, overall health and fitness, diet, and anxiety and depression, healthcare utilisation and ability to work. They will also be asked about problems they may have experienced since their surgery such as pain and surgical site infection post discharge.). Where patients are not completing PROMS and there is no reliable clinical weight record, we will write after 3 years requesting completion of a weight questionnaire (simply asking their current weight). The patients will be offered a voucher based incentive of £30 for completing both the year 2 and 3 questionnaires (high street voucher), and £10 for the year 3 weight follow-up questionnaire (for those no longer in clinical follow-up/not completing PROMS)

3.1.3 Part 3: Registration for a database of bariatric patients for future research

Patients who have agreed to Parts 1 and 2 will be asked if they are willing to be contacted about possibly taking part in other studies. This will not be an obligation to take part, just an expression of interest in being invited to take part. This will create a database of patients who have had bariatric surgery and are willing to consider participation in future research. Ethical opinion will be sought for the development of these study protocols.

For peer review only - http://bmjopen.bmj.com/site/about/guidelines.xhtml

3.1.4 Service user involvement

SCOTS will uphold the principles embodied by NIHR INVOLVE in relation to user involvement in research, to provide training and support, negotiated roles, time and equitable access for service users involved in this study. SCOTS recruited a patient focus group made of service users who have undergone different procedures both in NHS and private healthcare.

The SCOTS patient focus group have commented on the design of the study, in particular by providing advice on the acceptability of the patient reported outcome questionnaires, the burden to patients and on material and resources that will be given to potential participants. Future sessions are planned to discuss patient recruitment and retention strategies. SCOTS have included a service user on the SCOTS Steering Committee.

3.2 Study Population

All patients due to have a bariatric surgical procedure in Scotland will be invited to participate - both NHS and private sector.

A bariatric surgical procedure is defined as a surgical intervention which has the primary purpose of large scale weight loss in a patient who is obese. Currently those procedures include: duodenal switch; gastric banding; gastric bypass and sleeve gastrectomy. The inclusion of emerging techniques in this definition will be decided by the steering committee.

3.3 Reference populations

Within this protocol the objectives will be met by comparing before and after surgery outcomes for the SCOTS cohort. Additional funding shall be sought to develop protocols to allow comparisons with the following groups for a final Health Technology Assessment report.

1. SCOTS will compare outcomes with the IT based non-surgical weight management database in Greater Glasgow and Clyde (treatment seeking population with current data on 10,000 patients and 3,000 new referrals per year) which has prospective consent for research allowing data linkage for morbidity and mortality along with adjustment for smoking, deprivation, BMI, age and sex.

2. Standardised mortality ratios are available for a BMI matched cohort from the Scottish Health Survey.

For peer review only - http://bmjopen.bmj.com/site/about/guidelines.xhtml

3. The Scottish diabetes database allows an anonymous control group matched for key demographics to be created with prospective follow up of diabetes care and complications, compared to those with diabetes who had bariatric surgery.

3.4 Inclusion criteria

- Patients aged 16 years or over undergoing their first bariatric surgery in NHS hospitals or private practice in Scotland.
- Capacity to consent
- Resident in Scotland
- Written informed consent

3.5 Exclusion criteria

- Does not meet inclusion criteria
- Patients who have had previous weight loss surgery (at time of potential recruitment are undergoing a repeat procedure)
- No written informed consent
- Patients under 16 years
- Patients who do not have capacity to consent for themselves
- Weight loss surgery performed at a site outside Scotland
- Patients who live outside Scotland
- Patients who do not have strong enough English language skills to understand the patient information leaflet without assistance will be excluded from Part 2 of the study, the patient questionnaires. If they require a translator to understand the information they may be able to participate in Part 1 of the study which requires no further interaction from the patient. This decision will be made by the clinical teams caring for patients.

3.6 Identification of participants and consent

Patients will be prospectively identified by clinical teams as they are referred for surgery. Patients will be recruited from both NHS and private bariatric surgical practice in Scotland. They will either be recruited by a member of the surgical team prior to undergoing a bariatric surgical procedure, including any significant pre-operative weight loss (such as a very low energy diet for liver shrinkage or use of an intra-gastric balloon) which is considered part of the surgical pathway, or the surgical team will ask the patient's permission to pass over their name and contact details to the SCOTS research team who will then contact the patient directly to give further information and obtain fully informed consent.

For peer review only - http://bmjopen.bmj.com/site/about/guidelines.xhtml

Depending on local patient pathways patient information sheets will be provided to patients at a clinic visits or group session. This will be provided at least 24 hours before the patient will be asked to consent. In the case of those patients who are to be recruited by the research team (identified by surgical team), a patient information sheet will be provided when the permission is sought to hand over contact details to the research team. Patients who meet the eligibility criteria will be identified by their local clinical care teams. All patients undergoing bariatric surgery will be recorded on the screening log at their next appointment. This will allow patients to consider their participation and ask any additional questions they may have about the study face to face. An independent contact is provided on the patient information leaflet who patients can contact to discuss participation in research studies.

They will be asked to give fully informed consent for clinical data linkage (Part 1) and to be contacted by Glasgow University by post, email or phone about to completing the Patient questionnaires (Part 2) and for permission to contact them about future studies (Part 3). Patients who require a translator will only be asked to participate in Part 1. Only patients who agree to Part 1 will be eligible for Part 2 or Part 3.

3.7 Withdrawal of subjects

Participants have the right to withdraw from the study at any point for any reason. The SCOTS web site and patient information sheets will provide contact details for patients if they wish to withdraw from the study. If patients have asked to complete the forms by paper, contact details will be included within each paper questionnaire. The SCOTS team will provide patients with a form to complete, if they are contacted with a request to withdraw from the study.

If the SCOTS team are contacted by a patient who no longer wishes to participate in the study, they will be contact by the SCOTS team at University of Glasgow and asked to specify which following options they would like to apply to their data:

A. (For those within Part 1) I no longer wish to continue the study but I am content to allow my existing data to remain within the study for analysis.

B. (For those consented to Part 1 and 2). I am content for my records to be linked but I no longer wish to complete the patient questionnaires.

C. (For those consented to Part 1, 2 and 3). I am content for my records to be linked but I no longer wish to complete the patient questionnaires or be contacted regarding future studies.

D. For any patient I would like to completely remove my all data from the study and wish not to be contacted again by the study.

For peer review only - http://bmjopen.bmj.com/site/about/guidelines.xhtml

When a request is received from a patient to withdraw from the SCOTS study, a standardised form will be sent to the patient by their preferred communication route. The form will be completed by the patient including the patients name date of birth and address, and the on receipt data manager will flag the patient as having withdrawn from the study. The patient will be asked if they would like to be informed of the findings of the study even though they will no longer be participating in the study.

4 STUDY PROCEDURES

The study design involves local clinical teams identifying and potentially recruiting participants (some participants will be recruited by the SCOTS research team after identification by the local clinical team). Patients who consent to participate will have recruitment data collected by their clinical care teams or the SCOTS research team. Following their operation patients will have their weight loss surgery details entered into the database. The clinical teams then use the system to include follow up weights and re-operations. For the remainder of the study, patients who have consented to part 2 of the study will be asked to complete questionnaires and their health outcomes will be recorded by record linkage to routine data sets. A detailed breakdown of patient contacts with the study is described in Table 4-1~~Table 4-1~~ below.

TABLE 4-1: SCOTS PATIENT VISIT SCHEDULE: DESCRIBES EACH INTERATION WITH PATIENTS IF THEY CONSENT TO PART 1 AND 2 OF THE STUDY.

Patient contact	Time	Routine care	SCOTS	Part 1	Part 2	Part 3
1	1 year to 6 weeks before surgery	Agree bariatric surgery	Patients given/sent Invitation to participate letter and patient information sheet (and asked if they are to be contacted by SCOTS research team for further information and informed consent if no local recruitment)	1	1	
2	1 year to 6 weeks before surgery	Patient pre-surgical clinic visit, dietician, psychologist if local recruitment; phone call from SCOTS research team member if research team recruitment	Patients asked if they are willing to consent to participate in SCOTS, Patient signs consent forms, clinical team and patient complete contact details and baseline height and weight.	1	1	
3	At least one month before surgery		Patient contacted by SCOTS team to complete pre-operative questionnaire	1	1	
4	Date of surgery	Patient has weight loss operation	Clinical team enters details of weight loss operation to SCOTS portal	1	1	

For peer review only - http://bmjopen.bmj.com/site/about/guidelines.xhtml

5	Patient admitted to hospital	Identified by record linkage	If patient hospital admission is possibly related to bariatric surgery, patient will be contacted by SCOTS Team.	1	1	
7	At routine follow up visits	Patients attend routine clinical visits	Clinical teams enter weight and re-operation details		1	
8	Two year post surgery	Patients have routine annual blood test and results are made available via SCI-store.	Patients complete two year post surgical questionnaire		1	
9	Three years post surgery	Patients have routine annual blood test and results are made available via SCI-store.	Patients complete three year post surgical questionnaire		1	
10	Any point after recruitment		Possibly contacted to participate in future studies			1
11	Three years onward	Patient continues to have routine annual blood test every year	Patient contacted to thank them for completing the PROMS. Record linkage continues. Patients will be informed about future SCOTS publications.		1	
Total				5	9	1

Figure 4-1~~Figure 4-1~~ shows what happens at each point within the study. This includes background record linkage and patient contacts.

FIGURE 4-1: BASELINE – PRIOR TO OPERATIVE WEIGHT LOSS (INCLUDING PRE LIVER-PREP DIET OR BALLOON PHASE)

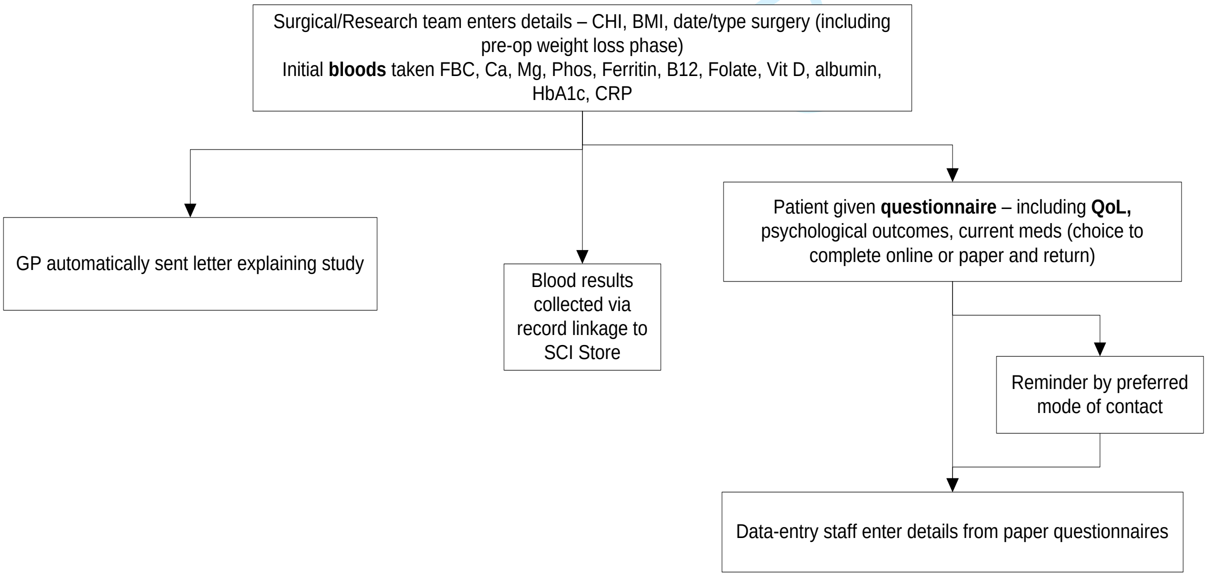

For peer review only - http://bmjopen.bmj.com/site/about/guidelines.xhtml

For peer review only

FIGURE 4-2: 2 AND 3 YEARS POST DEFINITIVE SURGERY

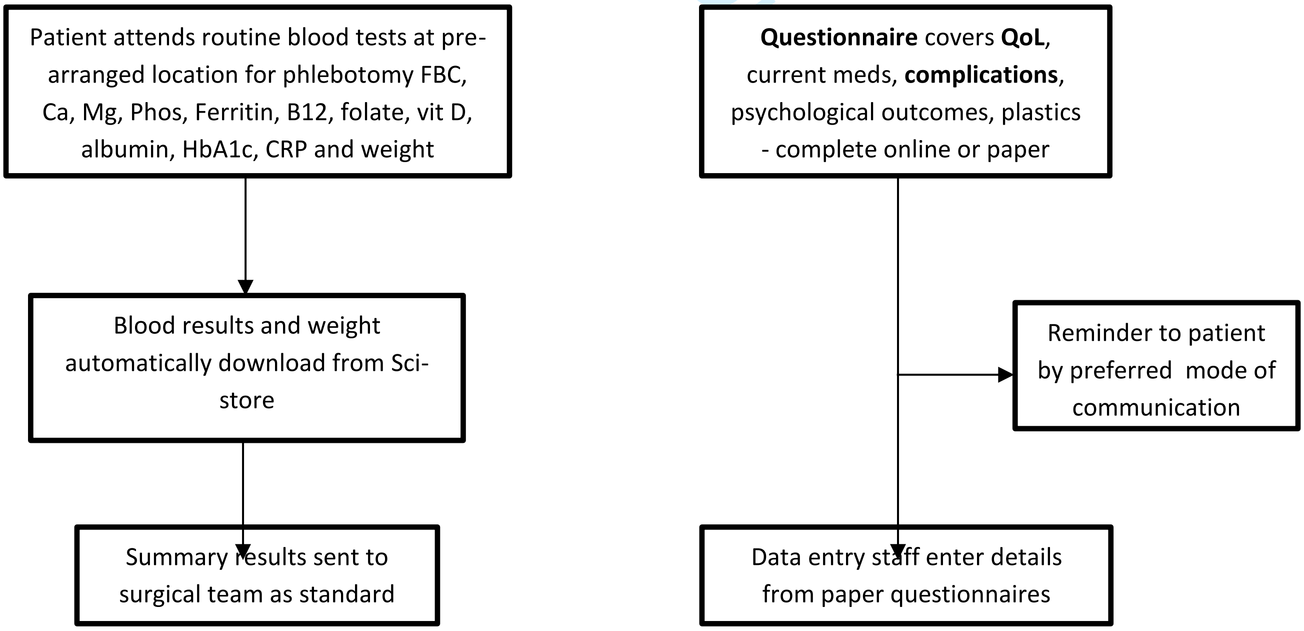

For peer review only - http://bmjopen.bmj.com/site/about/guidelines.xhtml

FIGURE 4-3: PART 3 IF NEW BARIATRIC SURGERY STUDY BEGINS

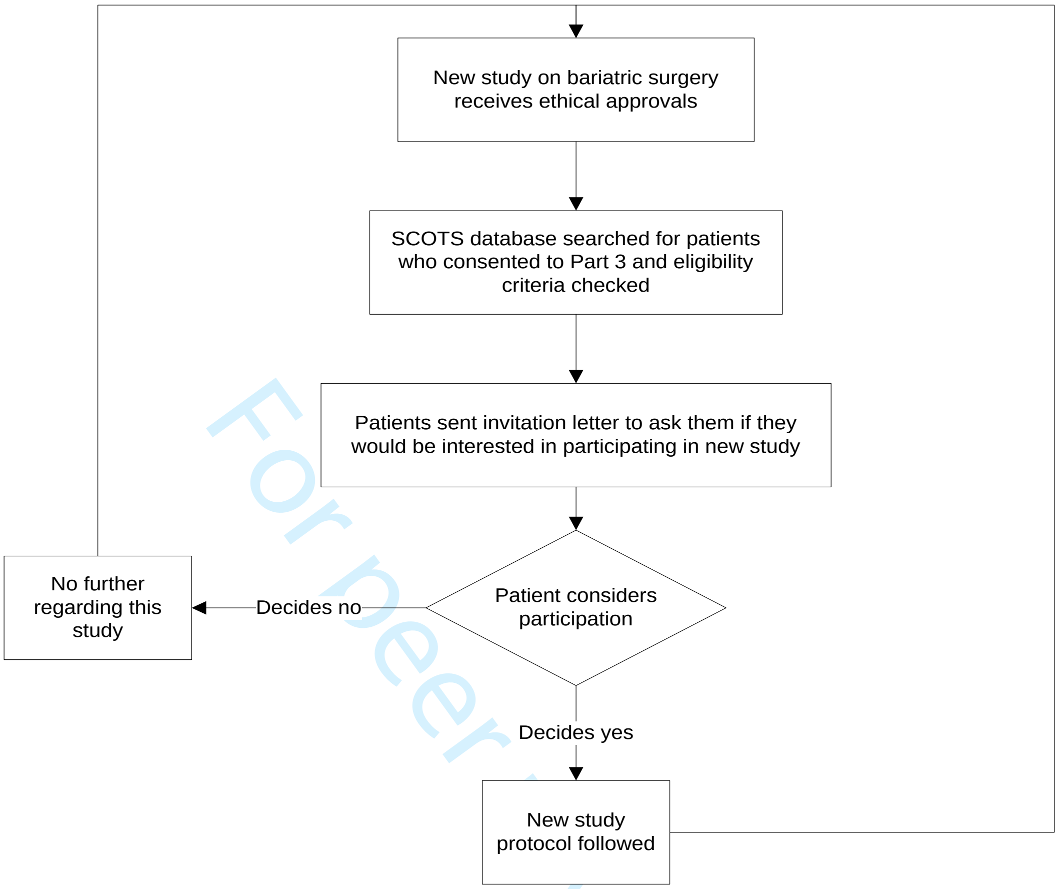

4.1 Duration of participation

Part 1: if funding is available, record linkage, follow up of patients will continue throughout their life. The end of a patient's life will be shown by linkage to the GRO data set. This will be used to flag deceased patients.

Part 2: patient reported outcome measures (PROMS) questionnaires will be collected for a maximum of 3 years. Collection of PROMS will stop after December 2020.

Part 3: Patient contact details will be kept by the study team until the end of the patient's life. The end of a patient's life will be shown by linkage to the GRO data set. This will be used to flag deceased patients and they will be removed from the database in Part 3 to ensure patients families are not contacted after their death. Patient will only be contacted for future studies if record linkage is available to show that they are still living.

Patients will be able to withdraw from the study at any point. Data collected will be retained unless explicitly requested to be removed from the study by the patient.

For peer review only

For peer review only - http://bmjopen.bmj.com/site/about/guidelines.xhtml

4.2 Study schedule

This section provides a brief overview of the procedures for investigators at each point within the study. SCOTS will integrate into routine care pathways for bariatric surgery as much as possible. No additional clinic visits will be required. There are five points within the patient journey that the clinical/research team are required to undertake any tasks. These points are described in detail in the following sections.

4.2.1 Start of Study - Surgical team registration

Each site will be defined before the start of the study and login and passwords will be created for each principal investigator and their clinical teams.

A training plan will be developed for clinical teams. Clinical teams will be provided with an overview of the study provided with a training package which will be developed electronically, to be completed at their convenience, electronic guidance will be integrated into the data collection tool to assist them. A user manual will be created which will explain the data which is required for each field and define each data field.

An electronic form will be used to collect surgeon specific data the first time they log onto the bariatric data collections system. This form will be developed by the study Data Centre at the Robertson Centre for Biostatistics, University of Glasgow and access to the form will be restricted, with only authorised site-specific personnel able to make entries or amendments to their data. A manual will be developed to assist investigators with use of the electronic data collection system. It is the investigator's responsibility to ensure completion and to review and approve all data captured. Surgeons will be asked to outline which locations they work in, which procedures they use, and the details of these procedures. They will also be asked information on their surgical experience including date when they qualified, number of procedures the previously year, total number of procedures undertaken in the last five years.

The aim of this task is to ensure that surgeons' data is entered at the start of the study and patient records can be pre-populated to ensure that data can be collected as efficiently as possible.

Checklist:	
Surgeon registers on SCOTS system	
Surgeon enters names and email addresses of clinical team who will be part of SCOTS investigators team	
Investigators have the opportunity to view SCOTS training materials	
SCOTS resource pack is available at the site	

For peer review only

For peer review only - http://bmjopen.bmj.com/site/about/guidelines.xhtml

4.2.2 Visit 1 Routine care invitation visit

Patient pathways vary throughout Scotland, invitation and introduction should take place at either an individual's clinic visit or at a group session discussing weight loss surgery. The aim of this visit is to ensure that patients who are planning weight loss surgery are aware of the SCOTS study, why it is being undertaken and what would be required from them.

4.2.2.1 Invitation visit protocol (in sites with local team recruitment)

1. Patient attends routine clinic to discuss bariatric surgery with clinical team.
2. Patient provided with an overview of SCOTS
3. Patient provided with opportunity to ask initial questions
4. Patient provided with invitation to participate in SCOTS and patient information leaflet (pre-prepared pack).

Checklist:	
Patients provided with overview of SCOTS	
Patient provided with SCOTS information pack	

4.2.2.2 Invitation visit protocol (in sites with SCOTS research team recruitment)

5. Patient attends routine clinic to discuss bariatric surgery with clinical team.
6. Patient provided with a summary overview of SCOTS
7. Patient provided with invitation to participate in SCOTS and patient information leaflet (pre-prepared pack).
8. Patient asked permission for surgical team to pass on contact details to SCOTS research team
9. Participant Identification form completed and returned to research team

Checklist:	
Patients provided with a summary overview of SCOTS	
Patient provided with SCOTS information pack	
Permission sought and Participant Identification Form completed and returned	

4.2.3 Visit 2 Recruitment visit

At the next follow up clinic appointment patients will be asked if they wish to take part in SCOTS. Patients must have been given more than 24 hours to consider their participation.

For peer review only - http://bmjopen.bmj.com/site/about/guidelines.xhtml

For patient's in sites where recruitment is carried out by the SCOTS research team the following will occur after the research team receive the participant information form from the local site:

1. SCOTS research team contact patient via telephone
2. Patient provided with an overview of SCOTS
3. Patient provided with opportunity to ask initial questions

Details of all patients who plan to have bariatric surgery will be recorded on a screening log. These logs will recorded on paper. Every patient who plans bariatric surgery will be logged anonymously with details of the individual's age category on date of invitation, gender, calendar quarter surgery planned, if they meet the SCOTS eligibility criteria, if they consent and type of surgery planned (for participants identified via local sites but recruited by the SCOTS team only those giving permission for their details to be forwarded to local teams will have a screening log completed as the research team shall do this). The purpose of this log is to demonstrate if the SCOTS data set is representative of all patients who are having bariatric surgery in Scotland. It will also provide a record of number of patients who consent. A copy of the screening logs will be sent to the Robertson Centre on an annual basis.

This is an anonymised screening record and patients who do not wish to participate will have no further information collected.

4.2.3.1 Informed consent protocol (in sites with local team recruitment)

1. Ask the patient if they have any questions relating to the study documentation. Ensure that the patient fully comprehends the information given.
2. Explain that participation is voluntary and should they decide to proceed with participation in the study, they are free to withdraw at any time without giving an explanation.
3. If a patient chooses to decline, thank them for their time and allow them to leave.
4. If they are unsure, allow further time to consider and arrange a convenient date to meet again reiterating that their participation is voluntary. Offer the option to speak to another member of the study team or an independent advisor.
5. All the check boxes within the consent form must be initialled by the patient to confirm they understand each part of the study. Ask the patient to initial each part of the consent form and sign the bottom section adding their name in block capitals.
6. The person obtaining informed consent signs and dates the consent forms adding their name in block capitals.
7. If a witness is required they can also sign and date the consent form (this may be in the case where an interpreter is required).
8. One copy of the form should be provided to the patient, one filed in the patient's notes and one copy stored in the site folder, and a copy sent to the Robertson Centre for the study files. A copy of the version of the patient information leaflet which was given to the patient should be filed within the site study file.

For peer review only - http://bmjopen.bmj.com/site/about/guidelines.xhtml

9. If the patient consents to the study the patient recruitment form should be completed in the presence of the patient. Record patient name and CHI number (for NHS patients), sex, height and weight.
10. Patients should be asked to complete the contact details part of the form including their e-mail and mobile phone contact details and ensure that they identify their preferred route of communication.
11. The Patient recruitment form will be send to the Robertson Centre and a copy filed within the site study file; When they are sent to the Robertson Centre a form will be faxed to show what documentation has been sent in the post, this will be recorded in a postage log.
12. Paper copies of the screening log are returned to the Robertson Centre annually, in self addressed envelopes provided by the SCOTs project team. These will also be recorded in a postage log.

Checklist:	
Check patient name and date of birth correct on patient recruitment form	
Consent from signed and dated at each point	
Patient retains copy of consent form	
Copy of consent form filed in notes	
Copy of consent form in study file	
Copy of consent form in study file sent to Robertson Centre	
Copy of patient recruitment form sent to Robertson Centre	
Copy of patient recruitment form filed in study file	
Consent form and patient recruitment form entered on postage log	

4.2.3.2 Informed consent protocol (in sites with SCOTS research team recruitment)

1. When the Participant Identification Form is received from the surgical team start to complete the SCOTS Recruitment Log and contact the patient via telephone.
2. Ask the patient if they have any questions relating to the study documentation. Ensure that the patient fully comprehends the information given.
3. Explain that participation is voluntary and should they decide to proceed with participation in the study, they are free to withdraw at any time without giving an explanation.
4. Complete the screening log (do not add participant ID as form is to be anonymous)
5. If a patient chooses to decline, thank them for their time and allow them to leave.
6. If they are unsure, allow further time to consider and arrange a convenient date to phone again reiterating that their participation is voluntary. Offer the option to speak to another member of the study team or an independent advisor.
7. All the check boxes within the consent form must be initialled by the patient to confirm they understand each part of the study. Ask the patient to initial each part of the consent form and sign the bottom section adding their name in block capitals.
8. For participants should then be asked post the completed consent form back to the research team in the stamped addressed envelope provided.

For peer review only - http://bmjopen.bmj.com/site/about/guidelines.xhtml

If the research team does not receive the completed consent form from the patient within two weeks and cannot reach the patient by telephone, a follow up letter should then be sent.

9. On receipt of the signed form, the person that obtained informed consent signs and dates the consent forms adding their name in block capitals.
10. If a witness is required they can also sign and date the consent form (this may be in the case where an interpreter is required).
11. One copy of the form should be provided to the patient (posted back in case of SCOTS team recruitment), one filed in the patient's notes and one copy stored in the site folder, and a copy sent to the Robertson Centre for the study files. A copy of the version of the patient information leaflet which was given to the patient should be filed within the site study file.
12. If the patient consents to the study the patient recruitment form should be completed with the patient via the telephone. Record patient name and CHI number (for NHS patients), sex, height and weight.
13. Patients should be asked for their contact details including their e-mail and mobile phone contact details and ensure that they identify their preferred route of communication.
14. The Patient recruitment form will be send to the Robertson Centre and a copy filed within the site study file; When they are sent to the Robertson Centre a form will be faxed to show what documentation has been sent in the post, this will be recorded in a postage log.
15. The Letter to Inform Surgeon of Participation should be completed and sent to the surgeon along with the relevant operation details recording form.
16. Paper copies of the screening log are returned to the Robertson Centre annually, in self addressed envelopes provided by the SCOTS project team. These will also be recorded in a postage log.

Checklist:	
Check patient name and date of birth correct on patient recruitment form	
Consent from signed and dated at each point	
Patient sent copy of signed consent form	
Copy of consent form filed in notes	
Copy of consent form in study file	
Copy of consent form in study file sent to Robertson Centre	
Copy of patient recruitment form sent to Robertson Centre	
Copy of patient recruitment form filed in study file	
Consent form and patient recruitment form entered on postage log	

For peer review only - http://bmjopen.bmj.com/site/about/guidelines.xhtml

4.2.4 SCOTS study team contact patient

If the patient has consented to Part 2 of the study, the patient will be contacted by staff from the Robertson Centre by their preferred route of communication to invite them to complete a patient reported outcomes questionnaire. They will be provided with a username and initial password which will need to be changed the first time they login to their account. The preadmission questionnaires will be available for the patient to complete. Patients will be able to complete the questionnaire in one or more sittings. If the patients have not logged into the system within two weeks of receiving their information they will be sent a reminder message by their preferred mode of contact. Given the smaller numbers recruited, the patients are now being offered a voucher based incentive to ensure a good return of the two and three year questionnaires. They will be offered an incentive of £30 for completing both the year 2 and 3 questionnaires, and £10 for the year 3 weight follow-up questionnaire (for those no longer in clinical follow-up/not completing PROMS).

Patients will be contacted two and three years after their weight loss operation. Patients will be contacted as part of the study closure phase to thank them for their participation in the study and inform them that they are no longer required to complete the questionnaires.

4.2.5 Visit 3 Weight loss operation

After the patient has their weight loss operation, the clinical teams will enter data onto the SCOTS data collection tool. This will involve the team member who has been delegated to record surgical information on the system. They will log in and be asked for the location of the surgery, which surgeon operated on the patient and their usual operation details will be pre-populated. If the operation is in any way different from the surgeon's usual technique, an edit function will allow the exact details of the operation to be recorded.

If the operation is a re-operation on an existing SCOTS consented patient, the SCOTS database will be searched to allow the patient's re-operation to be recorded.

4.2.6 Visit 4 -Post operative follow up

When patients attend follow up visits to see their surgeon, clinical teams will record the patient's weight and any complications experienced by the patient. If the patient has a gastric band, clinical teams will be able to record band adjustments in the data collection system. Essential fields for the follow-up visits are date of appointment and weight. Within the SCOTS portal it is possible for clinical teams to produce individual reports for patients on their weight loss for inclusion in their medical records, in the letters to GPs or for the patients themselves.

For peer review only - http://bmjopen.bmj.com/site/about/guidelines.xhtml

4.2.7 Visit 5 - Re-operation (If applicable)

If a patient requires a re-operation, surgeons will be able to record details of the latest procedure and the reasons for the re-operation. This can happen at any time throughout the study. Details of how the surgeons will record re-operations will be included within the user manual.

For peer review only

For peer review only - http://bmjopen.bmj.com/site/about/guidelines.xhtml

5 STATISTICS AND DATA ANALYSIS

5.1 Statistical Analysis Plan

The study will have separate detailed Statistical Analysis Plans to cover each analyses required for reporting to study committees, for publications and for the final report to the study funder, which will be authored by the study statistician and agreed by the Study Steering Committee before analysis takes place.

An important goal within the funded period is to build various predictive models to use accumulating information to predict good and bad outcomes, so that clinicians can identify treatment opportunities and make better informed intervention management decisions. Generically, these prediction models will relate potentially predictive information – both on entry into the SCOTS database on and around diagnosis, and from subsequent visits and at surgical and other treatment interventions – to outcomes of interest. The mainstay will be Cox proportional hazards time-to-event models, incorporating demographic, clinical, laboratory and life event data (e.g. hospitalisations, strokes, cancers, non-weight loss surgery, and so on) as both fixed and time varying covariates. The influence of missing data on the robustness of predictions in these models will be assessed. Importantly, a hierarchy of models will be built, with ease of use for the clinician in mind, from very simple models built from easy to obtain information, through to more complex models with very many and possibly some difficult and/or expensive to measure covariates.

5.2 Software for statistical analysis

The statistical software to be used for each analysis will be specified in the relevant Statistical Analysis Plan

5.3 Sample size

At the time of grant proposal writing, 230 operations were funded in NHS Scotland each year (of which approximately 60 are bypass). Bariatric surgeons performed an additional 270 private procedures per year and they were willing to commit to entering data (approximately 80 bypass). Therefore 500 procedures per year were expected to be entered into the database with a belief that as numbers of people with severe obesity (BMI >40) are rising rapidly, this number will increase despite financial constraints.

Previous recommendations from the NHS Scotland national planning forum (15)recommended that each health board should aim to provide 9 operations/100,000 population. Scotland's estimated population on the 30th June 2011 was 5,254,800 (16) (http://www.gro-scotland.gov.uk/files2/stats/population-estimates/mid-2011/j22829703.htm). This would mean around 470 operations per year within the NHS alone. This figure was expected to be reached over a number of years. Within private

For peer review only - http://bmjopen.bmj.com/site/about/guidelines.xhtml

healthcare bariatric surgery was estimated to be around 270 per year in Scotland (reported by personal communications from bariatric surgeons).

Given the potential for a surgical learning curve (17), new surgeon's data will be recorded but not analysed for the primary outcome until they have performed 50 operations. Collection of this early data will provide important information on the effects of the learning curve on patient outcomes.

From previous studies (3;4) we expected a 10-year mortality of around 5% (100 deaths). This sample size would allow the mortality rate to be estimated with 95% confidence to within +/- 1% (i.e. for a 5% 10-year death rate, the 95% confidence interval will be between 4% and 6%). We planned to compare this mortality rate with an age-sex matched healthy population from the Registrar General of Scotland's life tables (assumed known with no sampling error). 100 deaths was sufficient to allow us to build a predictive model for death post surgery (conventionally one requires around 10 events per prognostic covariate considered). An initial sample size of 2000 was proposed as it would easily provide adequate power for the original outcomes under investigation.

However, there have been a number of unforeseen recruitment issues that have impacted on the numbers stated above. In order to explore whether the current sample is likely to show meaningful results, the available statistical power for detecting 3 year difference in HbA1c and quality of life (physical and mental components) was calculated using the numbers of participants available as of 29th of July 2016. The majority of data to inform sample size were taken from papers where bariatric procedure was laparoscopic adjustable gastric banding (LAGB) as this is recognised as the least effective of the bariatric procedures so will give a conservative estimate of measure of effect. This shows that there is >99% power to show differences in these outcomes at three years with the current sample size.

In order to explore the likely event rate for cardiovascular events and deaths, we performed health record linkage for currently recruited participants, linking with inpatient care and death records. Follow-up is from the date of surgery, so for those patients that went on to have surery details entered (n=180), there are 272 separate admissions. Of those 272, there are 72 'emergency' admission, and 4 of these, in 3 patients, are 'circulatory disease' using the main condition only. The codes included for these hospital admissions are;

· Angina pectoris (x1)
· Acute MI (x1)
· Pulmonary embolism without mention of acute cor pulmonale (x1)
· Orthostatic hypotension (x1)

Using date of operation as the starting point, we have a total (crude) follow-up time of 203.52 years (the mean is 1.13 years and the median is 1.04 years). As the number of cardiovascular events is so low, it is impossible to extrapolate this to a future event rate at this time. It should be noted that the participants have been cleared as healthy for elective surgery meaning that it is unlikely that there would be many cardiovascular events in early follow-up.

For peer review only - http://bmjopen.bmj.com/site/about/guidelines.xhtml

Glasgow Clinical Trials Unit

Following discussions with the NIHR it was agreed that recruitment will stop at approximately 400 patients and these numbers will be sufficient to answer the majority of objectives initially set.

5.4 Management and delivery

The Robertson Centre for Biostatistics, part of the Glasgow Clinical Trials Unit, a fully registered UK CRN Clinical Trials Unit, will manage and analyse trial data.

For peer review only

For peer review only - http://bmjopen.bmj.com/site/about/guidelines.xhtml

6 STUDY CLOSURE

Part 1 Record linkage: under the current funding record linkage will continue until December 2020. If additional funding is available Part 1 record linkage will continue until the end of the patient's life. The end of a patient's life will be shown by linkage to the GRO data set. This will be used to flag deceased patient and they will be removed from the database in Part 3 to ensure patients families are not contacted after their death.

Part 2 Patient reported Questionnaires: The study will end when the steering committee agrees that one or more of the following situations applies:

- Observation has been undertaken until December 2020
- Sufficient patients have completed the 3 year post surgery questionnaire

Patients will be contacted to thank them for their participation in the patient reported outcomes part of the study.

Part 3 Bariatric Surgery Research Studies. Patients contact details will be maintained until the end of the patient's life.

7 Data Handling

Data for the SCOTS study will be collected from a number of sources. These are summarised in Table 3-1~~Table 3-1~~. A detailed data management plan will be developed by the Robertson Centre team, which will include verification of paper data entry and validation checks. A separate protocol will be developed for each data linkage.

7.1.1 Surgeon registration data

An electronic data collection form will be developed by the study Data Centre at the Robertson Centre for Biostatistics, University of Glasgow. These data will include the surgeons' usual procedures and registration of the clinical teams. These data will be used to complete the operation details for each patient. It will be the investigators responsibility to ensure that these data are entered correctly and are kept up to date.

7.1.2 Patient recruitment data

A paper case report form (CRF) will be used to collect patient contact details and height and weight at time of recruitment will be completed by the clinical teams when the patient has consented to participate in SCOTS. Patients will be asked to specify their preferred form of communication throughout the study. They will be told that they may be contacted via another route if they do not respond. This CRF will be developed by the study Data Centre at the Robertson Centre for Biostatistics, University of Glasgow. Competed forms will be checked by the clinical teams and sent to the Robertson Centre for initial data entry. These forms will be sent along with signed consent forms. It is the investigator's responsibility to ensure completion and to review and approve all data captured in the CRF. A standard

For peer review only - http://bmjopen.bmj.com/site/about/guidelines.xhtml

operating procedure will be used by the Roberson Centre to ensure that information is entered into the SCOTS database in an accurate and timely manner.

7.1.3 Surgical data collection

An electronic case report form (e-CRF) will be used to collect data on weight loss surgery procedures. On or shortly after the patient undergoes weight loss surgery a member of the clinical team will record specific information on the patient's weight loss operation. Each surgeon will have been asked to register the locations they operate in, which procedures they perform and details of their usual specifications. This will then populate the fields for a particular type of surgery and clinical teams will be asked to confirm that the usual techniques have been applied for each procedure. Access to the e-CRF will be restricted, with only authorised site-specific personnel able to make entries or amendments to their patients' data. It is the investigator's responsibility to ensure completion and to review and approve all data captured in the e-CRF, however, a member from the SCOTS study team, with the appropriate NHS Research Passport permissions, will visit the site staff at least once per year to ensure surgical and follow-up data are complete. Data will be validated at the point of entry into the e-CRF and at regular intervals during the study. Data discrepancies will be flagged to the study site and any data changes will be recorded in order to maintain a complete audit trail (reason for change, date change made, who made change).

7.1.4 Patient Reported Outcomes

An electronic case report form (e-CRF) or a paper case report form (depending on patient preference) will be used to collect patient reported study data. The e-CRF and CRF will be developed by the study data centre at the Robertson Centre for Biostatistics, University of Glasgow and access to the e-CRF will be restricted, with only authorised site-specific personnel able to make entries or amendments to their patients' data. All data handling procedures will be detailed in a study specific Data Management Plan.

7.1.5 Routine clinical information

A number of features have been built into the SCOTS system which will be available for surgeons to use during routine follow up visits. Each time a patient attends, their weight will be recorded. Notes can be included and exported for inclusion within patient notes, provided for patients or within letters to GPs.

7.1.6 Re-operation

An electronic case report form (e-CRF) will be used to collect data on re-operations. On or shortly after the patient undergoes weight loss surgery a member of the clinical team will record specific information on the patients repeat weight loss operation. Not only will the details of the operation be recorded but the reason for operation will be included within the e-CRF.

For peer review only - http://bmjopen.bmj.com/site/about/guidelines.xhtml

7.1.7 Record Retention

To enable evaluations and/or audits from regulatory authorities, the investigator agrees to keep records, including the identity of all participating subjects (sufficient information to link records), all original signed informed consent forms, source documents, and detailed records of treatment disposition in accordance with ICH GCP, local regulations, or as specified in the Clinical Study Agreement, whichever is longer. Data will be retained at the Robertson Centre once the recruitment phase of the study is complete. During recruitment a copy of the patient consent form and the patient registration will be retained within the site file and within the patient notes.

7.1.8 Access to data

Clinical teams will be able to access their own data. All patients who have been entered within the SCOTS system will be viewed by the clinical teams who entered data. Data which is routinely collected for the National Bariatric Surgery Registry (NBSR) can be entered within the system for surgeons who wish to contribute to this process. Surgeons fully anonymised data (no patient identifiable information) can be exported to a file which surgeons can share with the NBSR if they surgeons wish to contribute. The management and approvals for this will be responsibility of the investigating surgeon and data will not be shared by the SCOTS team.

For peer review only

For peer review only - http://bmjopen.bmj.com/site/about/guidelines.xhtml

8 SCOTS STUDY MANAGEMENT

8.1 Study Sponsor

NHS Greater Glasgow and Clyde Research and Development Office as study sponsors are responsible for the management and conduct (according to GCP). The SCOTS steering committee (SSC) oversee the management and conduct of the study and report to and advise the sponsor on the study. All minutes from both the SCOTS management group (SMG) and SCOTS steering committee (SSC) are copied to NHS Greater Glasgow and Clyde Research and Development Office

8.2 SCOTS Study Team

A study team comprising of the chief investigator, a research lead and a project manager, in collaboration with the data management team based at the Robertson Centre will run the project on a day to day basis. Issues arising from patients or investigators will be managed by this team and those that cannot be resolved by that team will be discussed with the chief Investigator and escalated to the study management group for discussion.

8.3 SCOTS Principal investigators

Each site will have an identified principal investigator who will oversee the application of this protocol locally. Each member of the clinical team involved in the study will be required to register for a login and password to access the e-CRF through the SCOTS data collection portal. Any member of the local clinical teams who has registered username and password and entered data to the SCOTS data collection portal will be a member of the SCOTS Investigators Group. The investigators will be required to enter some initial registration data to link themselves with the surgeons they work with. Planned publications outlined in Table 15-1~~Table 15-1~~ will be written on behalf of the SCOTS Investigators Group.

8.4 Routine management of study: SCOTS Management Group (SMG)

The trial will be coordinated from the University of Glasgow by the SCOTS management group (SMG). The SMG will consist of the principal investigator, other co-applicants, project manager and representatives from the Clinical Trials Unit. The role of the group is to monitor all aspects of the conduct and progress of the trial, ensure that the protocol is adhered to and take appropriate action to safeguard participants and the quality of the trial itself. It will be responsible for the day to day running of the trial and budget. It will meet at least 6 monthly, with more meetings initially and as required, and provide information to the steering committee as to the progress of the study.

For peer review only - http://bmjopen.bmj.com/site/about/guidelines.xhtml

8.5 SCOTS Steering Committee (SSC)

SCOTS Steering Committee (SSC) will be established to provide overall supervision of the study and ensure adherence to GCP. It will have an independent chair, a patient representative, and at least two other independent academics along with the principal investigator. Observers from the HTA programme will be invited to SSC meetings, and all SSC papers will be supplied to the HTA programme. The SSC will meet at the start of the study, and annually thereafter.

The role of the SSC is to provide overall supervision of the trial and ensure that it is being conducted in accordance with the principles of GCP and the relevant regulations. The SSC will:

- agree the trial protocol and any protocol amendments
- provide advice to the investigators on all aspects of the trial
- include members who are independent of the investigators, in particular an independent chairperson.

Decisions about continuation or termination of the trial or substantial amendments to the protocol are usually the responsibility of the SSC who advise the sponsor.

For peer review only

For peer review only - http://bmjopen.bmj.com/site/about/guidelines.xhtml

9 STUDY MONITORING/AUDITING

This study is a cohort study. It will therefore be audited on random sampling basis by GG&C auditors on behalf of the GG&C sponsor who audit 10% of sponsored studies per annum.

10 PROTOCOL AMENDMENTS

Any change in the study protocol will require an amendment. Any proposed protocol amendments will be initiated by the CI following discussion with the SSC and any required amendment forms will be submitted to the regulatory authority, ethics committee and sponsor. The CI and the SSC will liaise with study sponsor to determine whether an amendment is non-substantial or substantial. All amended versions of the protocol will be signed by the CI and sponsor representative. Before the amended protocol can be implemented favourable opinion/approval must be sought from the original reviewing Research and Ethics Committee (REC) and Research and Development (R&D) office(s).

11 ETHICAL CONSIDERATIONS

11.1 Ethical conduct of the study

The study will be carried out in accordance with the World Medical Association Declaration of Helsinki (1964) and its revisions (Tokyo [1975], Venice [1983], Hong Kong [1989], South Africa [1996] and Edinburgh [2000]).

Favourable ethical opinion will be sought from an appropriate REC before patients are entered into this study. Patients will only be allowed to enter the study once they have provided written informed consent.

The CI will be responsible for updating the REC of any new information related to the study.

11.2 Informed consent

Patients will be informed of the SCOTS study as they discuss weight loss surgery with their clinical teams. They will be provided with an information sheet on the study by a trained member of the clinical teams during a routine clinic visit or during a group session depending on the local patient pathway. The patient will be given time to consider their participation in the study, review the SCOTS information sheet and ask about any concerns they may have. Patients will then be asked to sign the SCOTS consent form.

For peer review only - http://bmjopen.bmj.com/site/about/guidelines.xhtml

12 INSURANCE AND INDEMNITY

SCOTS is sponsored by NHS Greater Glasgow & Clyde and NHS indemnity is provided under the Clinical Negligence and Other Risks Indemnity Scheme (CNORIS). In addition, as the protocol authors are employees of the University of Glasgow negligent harm caused by the design of the trial will be covered by the Clinical Trials Insurance policy held by the University of Glasgow.

Investigators working in private healthcare will recruit to this NHS funded study as part of their NHS role and will therefore be covered as detailed above.

13 FUNDING

Funding source: National Institute for Health Research (NIHR) Health Technology Assessment Programme

Grant Award number: 10/42/02

Original Cost: £2,124,030

Amended Cost: £1,516,255.60

URL of this project on the NIHR Website: http://www.hta.ac.uk/project/2608.asp

Start Date: January 2012

End date: June 2021.

Estimated date of publication in HTA journal series: Late 2021. This date takes account of time for report preparation and printing based on current average times for these activities.

International Standard Randomised Controlled Trial Number: ISRCTN 47072588

URL of this project on the Controlled Trials Website: http://www.controlled-trials.com/ISRCTN47072588

14 ANNUAL REPORTS

A bi-annual progress report will be submitted to NIHR (the funder), the first being submitted 6 months from the date that all funding started (January 2012).

Annual reports will be submitted to the ethics committee and sponsor with the first submitted one year after the date that all study related approvals are in place. These reports will follow the template on the IRAS web site.

For peer review only - http://bmjopen.bmj.com/site/about/guidelines.xhtml

15 DISSEMINATION OF FINDINGS

Study results whether, negative or positive, will be disseminated. Details of the plan for dissemination are detailed below. Some of the outcomes described may be reported in a number of peer reviewed journal articles. The SCOTS Publication and Presentation SOP (18) describes how this will be managed throughout the study and will ensure that NIHR guidance for publications is adhered to.

TABLE 15-1: SCOTS PUBLICATION PLANS

	Date	Description of publication
1	**Jan 2015**	Methods paper: Outlining the methods used in the study including the IT based nutritional blood monitoring and the dataset being collected. Published – Logue et al BMJ Open 2015;5:e008106
2	**Jul 2017**	Health and socio-economic burden of severe obesity in a population undergoing bariatric surgery.
3	**Jul 2019**	The effect of the pre and post-surgery pathway and surgical experience on weight and complications outcomes after bariatric surgery.
4	**Jun 2021**	Change in health and socioeconomic factors 3 years after bariatric surgery.<br>Full HTA report within the NIHR HTA Journal Series.

For peer review only - http://bmjopen.bmj.com/site/about/guidelines.xhtml

16 REFERENCES

(1) Picot J, Jones J, Colquitt JL, Gospodarevskaya E, Loveman E, Baxter L, et al. The clinical effectiveness and cost-effectiveness of bariatric (weight loss) surgery for obesity: a systematic review and economic evaluation. Health Technol Assess 2009 Sep;13(41):1-357, iii.

(2) O'Brien PE, Sawyer SM, Laurie C, Brown WA, Skinner S, Veit F, et al. Laparoscopic adjustable gastric banding in severely obese adolescents: a randomized trial. JAMA 2010 Feb 10;303(6):519-26.

(3) Adams TD, Gress RE, Smith SC, Halverson RC, Simper SC, Rosamond WD, et al. Long-term mortality after gastric bypass surgery. N Engl J Med 2007 Aug 23;357(8):753-61.

(4) Sjostrom L, Narbro K, Sjostrom CD, Karason K, Larsson B, Wedel H, et al. Effects of bariatric surgery on mortality in Swedish obese subjects. N Engl J Med 2007 Aug 23;357(8):741-52.

(5) Dixon JB, O'Brien PE, Playfair J, Chapman L, Schachter LM, Skinner S, et al. Adjustable gastric banding and conventional therapy for type 2 diabetes: a randomized controlled trial. JAMA 2008 Jan 23;299(3):316-23.

(6) Buchwald H, Estok R, Fahrbach K, Banel D, Jensen MD, Pories WJ, et al. Weight and type 2 diabetes after bariatric surgery: systematic review and meta-analysis. Am J Med 2009 Mar;122(3):248-56.

(7) Sjostrom L, Lindroos AK, Peltonen M, Torgerson J, Bouchard C, Carlsson B, et al. Lifestyle, diabetes, and cardiovascular risk factors 10 years after bariatric surgery. N Engl J Med 2004 Dec 23;351(26):2683-93.

(8) Sjostrom L, Lindroos AK, Peltonen M, Torgerson J, Bouchard C, Carlsson B, et al. Lifestyle, diabetes, and cardiovascular risk factors 10 years after bariatric surgery. N Engl J Med 2004 Dec 23;351(26):2683-93.

(9) Mathus-Vliegen EM, de Wit LT. Health-related quality of life after gastric banding. Br J Surg 2007 Apr;94(4):457-65.

(10) Karlsson J, Taft C, Ryden A, Sjostrom L, Sullivan M. Ten-year trends in health-related quality of life after surgical and conventional treatment for severe obesity: the SOS intervention study. Int J Obes (Lond) 2007 Aug;31(8):1248-61.

(11) Ford I, Murray H, Packard CJ, Shepherd J, Macfarlane PW, Cobbe SM. Long-term follow-up of the West of Scotland Coronary Prevention Study. N Engl J Med 2007 Oct 11;357(15):1477-86.

(12) Research Governance Framework for Health and Community Care (Second edition). Scottish Executive Health Department; 2006.

For peer review only - http://bmjopen.bmj.com/site/about/guidelines.xhtml

(13) NHS National Services Scotland. ISD Statistical Disclosure Control Protocol. Edinburgh: ISD Scotland Publications; 2010.

(14) Scottish Intercollegiate Guideline Network. Management of Obesity 115 - A national clincal guideline. Edinburgh: Scottish Intercollegiate Guidelines Network; 2010 Feb 1. Report No.: 115.

(15) Knox H, Davies S. Obestity Treatment Best Practice Guidance. Edinburgh: SGHD Quality Unit, Planning and Quality Division; 12 A.D. Jul 1.

(16) Mid-2011 Population Estimates Scotland Population estimates by sex, age and administrative area.

National Statistics publication for Scotland; 2012 May 31.

(17) Chevallier JM, Paita M, Rodde-Dunet MH, Marty M, Nogues F, Slim K, et al. Predictive factors of outcome after gastric banding: a nationwide survey on the role of center activity and patients' behavior. Ann Surg 2007 Dec;246(6):1034-9.

(18) SCOTS Management Team. Surgical Obesity Treatment Study (SCOTS) Publication and Presentation Group Operating Procedures. 2012.

(19) Scottish Government. Scottish Health Survey 2010 - Volume 2: Technical Report. 2008.

(20) General Register Office. Scottish Census 2011 Household Questionnaire. General Register Office, editor. GRO . 2011.

Ref Type: Online Source

(21) Bruce J, Thornton AJ, Scott NW, Marfizo S, Powell R, Johnston M, et al. Chronic preoperative pain and psychological robustness predict acute postoperative pain outcomes after surgery for breast cancer. Br J Cancer 2012 Sep 4;107(6):937-46.

(22) Mercer SW, Watt GC. The inverse care law: clinical primary care encounters in deprived and affluent areas of Scotland. Ann Fam Med 2007 Nov;5(6):503-10.

(23) Wadden TA, Foster GD. Weight and Lifestyle Inventory (WALI). Obesity (Silver Spring) 2006 Mar;14 Suppl 2:99S-118S.

(24) Diabetes UK. Diabetes Risk Score. 2012. Diabetes UK.

Ref Type: Online Source

(25) Ware J, Jr., Kosinski M, Keller SD. A 12-Item Short-Form Health Survey: construction of scales and preliminary tests of reliability and validity. Med Care 1996 Mar;34(3):220-33.

(26) The EuroQol Group. EuroQol-a new facility for the measurement of health-related quality of life. Health Policy 1990;16(3):199-208.

For peer review only - http://bmjopen.bmj.com/site/about/guidelines.xhtml

(27) Spitzer RL, Kroenke K, Williams JB. Validation and utility of a self-report version of PRIME-MD: the PHQ primary care study. Primary Care Evaluation of Mental Disorders. Patient Health Questionnaire. JAMA 1999 Nov 10;282(18):1737-44.

(28) Cameron IM, Crawford JR, Lawton K, Reid IC. Psychometric comparison of PHQ-9 and HADS for measuring depression severity in primary care. Br J Gen Pract 2008 Jan;58(546):32-6.

(29) Spitzer RL, Kroenke K, Williams JB, Lowe B. A brief measure for assessing generalized anxiety disorder: the GAD-7. Arch Intern Med 2006 May 22;166(10):1092-7.

(30) Kolotkin RL, Crosby RD. Psychometric evaluation of the impact of weight on quality of life-lite questionnaire (IWQOL-lite) in a community sample. Qual Life Res 2002 Mar;11(2):157-71.

(31) Kolotkin RL, Crosby RD, Pendleton R, Strong M, Gress RE, Adams T. Health-related quality of life in patients seeking gastric bypass surgery vs non-treatment-seeking controls. Obes Surg 2003 Jun;13(3):371-7.

(32) Gosman GG, King WC, Schrope B, Steffen KJ, Strain GW, Courcoulas AP, et al. Reproductive health of women electing bariatric surgery. Fertil Steril 2010 Sep;94(4):1426-31.

(33) Derby CA, Araujo AB, Johannes CB, Feldman HA, McKinlay JB. Measurement of erectile dysfunction in population-based studies: the use of a single question self-assessment in the Massachusetts Male Aging Study. Int J Impot Res 2000 Aug;12(4):197-204.

(34) Barry MJ, Fowler FJ, Jr., O'Leary MP, Bruskewitz RC, Holtgrewe HL, Mebust WK, et al. The American Urological Association symptom index for benign prostatic hyperplasia. The Measurement Committee of the American Urological Association. J Urol 1992 Nov;148(5):1549-57.

(35) Abrams P, Avery K, Gardener N, Donovan J. The International Consultation on Incontinence Modular Questionnaire: www.iciq.net. J Urol 2006 Mar;175(3 Pt 1):1063-6.

(36) Macran S, Wileman S, Barton G, Russell I. The development of a new measure of quality of life in the management of gastro-oesophageal reflux disease: the Reflux questionnaire. Qual Life Res 2007 Mar;16(2):331-43.

(37) Grant A, Wileman S, Ramsay C, Bojke L, Epstein D, Sculpher M, et al. The effectiveness and cost-effectiveness of minimal access surgery amongst people with gastro-oesophageal reflux disease - a UK collaborative study. The REFLUX trial. Health Technol Assess 2008 Sep;12(31):1-iv.

(38) Helander A, Lunner K, Leifman A, Borg S. [Alcohol-related problems in connection with overweight surgery]. Lakartidningen 2011 May 4;108(18):964-5.

For peer review only - http://bmjopen.bmj.com/site/about/guidelines.xhtml

(39) Babor TF, Higgins-Biddle JC, Saunders JB, Monteiro MG. The Alcohol Use Disorders Identification Test. World Health Organisation Department of Mental Health and Substance Dependence; 2001 Jan 1. Report No.: WHO/MSD/MSB/01.6a.

(40) Craig CL, Marshall AL, Sjostrom M, Bauman AE, Booth ML, Ainsworth BE, et al. International physical activity questionnaire: 12-country reliability and validity. Med Sci Sports Exerc 2003 Aug;35(8):1381-95.

(41) Roe L, Strong C, Whiteside C, Neil A, Mant D. Dietary intervention in primary care: validity of the DINE method for diet assessment. Fam Pract 1994 Dec;11(4):375-81.

(42) Scheier MF, Carver CS, Bridges MW. Distinguishing optimism from neuroticism (and trait anxiety, self-mastery, and self-esteem): a reevaluation of the Life Orientation Test. J Pers Soc Psychol 1994 Dec;67(6):1063-78.

(43) Foster GD, Wadden TA, Vogt RA, Brewer G. What is a reasonable weight loss? Patients' expectations and evaluations of obesity treatment outcomes. J Consult Clin Psychol 1997 Feb;65(1):79-85.

(44) Health Protection Scotland. The epidemiology of orthopaedic surgical site infection occuring up to one year after surgery: a feasibility study of telephone screening and direct observation by trained healthcare workers. Health Protection Scotland; 2010.

(45) Horan TC, Andrus M, Dudeck MA. CDC/NHSN surveillance definition of health care-associated infection and criteria for specific types of infections in the acute care setting. Am J Infect Control 2008 Jun;36(5):309-32.

(46) Ertlet TW, Marino JM, Mitchell JE. Psychosocial aspects of body contourning surgery after bariatric surgery. In: Shiffman MA, Di Giuseppe A, editors. Body Contouring.Berlin: Springer-Verlag; 2010. p. 633-9.

(47) Keating CL, Moodie ML, Bulfone L, Swinburn BA, Stevenson CE, Peeters A. Healthcare Utilization and Costs in Severely Obese Subjects Before Bariatric Surgery. Obesity (Silver Spring) 2012 May 4.

(48) (DIRUM) Database of Instruments for Resource Use Measurement. 2012. Bangor, Bangor University.

Ref Type: Online Source

(49) Petrou S. WoLFF (Wound dressings in Lower Limb Fracture) Questionnaire. 2012. 1-8-2012.

(50) EPIC Norfolk Study. Food Frequency Questionnaire. Cambridge: University of Cambridge; 2012. Report No.: (CAMB/PQ/6/1205).

For peer review only - http://bmjopen.bmj.com/site/about/guidelines.xhtml

17 Appendix E: Summary of Patient Reported Outcome Questions

Table 17-1Table 17-1 shows a summary table of each set of questions which will be asked of the patient, when they will be asked and if they are validated or not validated.

Questions are classed as not validated in the form they are being asked if they have not been used in a published study and are identified by either a 0 or P in the validated column. 0 means that the set of questions are not validated P means that the questions are partially validated. Partially validated question sets are where a previously published question set has had additional questions added in order to fulfil the SCOTS objectives.

TABLE 17-1: SUMMARY OF SCOTS PATIENT REPORTED OUTCOME MEASURES.

For peer review only

For peer review only - http://bmjopen.bmj.com/site/about/guidelines.xhtml

Glasgow Clinical Trials Unit

PROMS	Total	Total Male	Total Femal	Validated	baseline	2 year	3 year	Total
About you								
Demographics – Essential	1	1	1	P	1	1	1	3
Marital Status	1	1	1	1	1	1	1	3
Ethnicity	1	1	1	1	1	0	0	1
Education	1	1	1	1	1	0	0	1
Occupation	1	1	1	P	1	1	1	3
Health History								
Your Medical History	1	1	1	1	1	0	0	1
Your Weight History	1	1	1	1	1	0	0	1
Family History	1	1	1	P	1	0	0	1
Family History of Obesity	1	1	1	0	1	0	0	1
Quality of Life								
SF-12 v2 –licence required	1	1	1	1	1	1	1	3
EQ5D 5L	1	1	1	1	1	1	1	3
Patient Health Questionnaire (PHQ-9) (No permissions Free)	1	1	1	1	1	1	1	3
Generalized Anxiety Disorder GAD-7 (No permissions Free)	1	1	1	1	1	1	1	3
Impact of Weight								
Impact of Weight on Quality of Life (IWQOL-Lite) – Licence required	1	1	1	1	1	1	1	3
Reproductive Health – Females only	1	0	1	1	1	1	1	3
Massachusetts Male Aging Study: Single Question Assessment Males only	1	1	0	1	1	1	1	3
International Prostate Symptom Score (IPSS) Males only	1	1	0	1	1	1	1	3
Continence	1	1	1	1	1	1	1	3
Reflux Questionnaire	1	1	1	1	1	1	1	3
Lifestyle								
Smoking	1	1	1	1	1	1	1	3
The Alcohol Use Disorders Identification Test: Self-Report Version	1	1	1	1	1	1	1	3
Physical Activity IPAC short form self administered	1	1	1	1	1	1	1	3
Nutrition	1	1	1	1	1	1	1	3
Personal Outlook								
Life Orientation Test (REVISED)	1	1	1	1	1	1	0	2
Expectations of Surgery	1	1	1	P	1	0	0	1
Surgical Recovery								
Post operative pain (1 month acute, 6 month-1 year chronic)	1	1	1	0	0	0	0	0
Surgical site infection	1	1	1	1	0	0	0	0
Skin Excess	1	1	1	0	0	0	0	0
Plastic surgery	1	1	1	P	0	0	0	0
Healthcare and Support								
Outpatient care	1	1	1	1	1	1	1	3
Contacts with other health and social care professionals	1	1	1	1	1	1	1	3
Devices and specialist equipment	1	1	1	1	1	1	1	3
Benefits	1	1	1	1	1	1	1	3
Multivitamins and Supplements use	1	1	1	1	1	1	1	3
	34	33	32	**26**	**30**	**23**	**22**	**75**

Surgical Obesity Treatment Study

For peer review only - http://bmjopen.bmj.com/site/about/guidelines.xhtml

For peer review only

This page is intentionally blank

For peer review only

For peer review only - http://bmjopen.bmj.com/site/about/guidelines.xhtml

Medical history questions included in baseline questionnaires/patient reported outcome measures (PROMs).

Why we are asking you these questions?

It is important to know what illness patients have before their surgery so we can look at how much overall health has changed after their weight loss surgery.

Medical History

1. Do you have a history of any of the following? – tick all that apply

- ☐ Deep vein thrombosis (Deep vein thrombosis (DVT) is a blood clot in one of the deep veins in the leg.)
- ☐ Pulmonary embolism (A pulmonary embolism is a blood clot in the pulmonary artery which is the blood vessel that transports blood from the heart to the lungs.)
- ☐ High blood pressure
- ☐ Diabetes
- ☐ Angina/heart attack
- ☐ Heart Failure
- ☐ Stroke/mini-stroke
- ☐ Arthritis
- ☐ Back problems
- ☐ Chronic Bronchitis
- ☐ Eczema/psoriasis
- ☐ Asthma
- ☐ Thyroid problems
- ☐ Migraine
- ☐ Anxiety/depression
- ☐ Kidney disease
- ☐ Liver disease
- ☐ Cancer
- ☐ Irritable bowel syndrome
- ☐ Doctor diagnosed sleep apnoea (Obstructive sleep apnoea (OSA) is a condition that causes interrupted breathing during sleep and requires a machine to keep airways open while you sleep. Only check this box if your doctor has diagnosed sleep apnoea.)
- ☐ Other (please specify _______________)

For peer review only

SCOTS

Patient Questionnaire

Draft 01

Lifestyle

This section asks about your smoking, drinking, eating and exercising habits.

Why are we asking you these questions

Smoking habits may alter after weight loss surgery.

Smoking

1. Smoking history

Current ☐ 1

Former ☐ 2

Never ☐ 3

If Current,

1a. How many per day? |__|__|__|

If former,

1a. Year stopped? |__|__|__|__|
Y Y Y Y

For peer review only

SCOTS

Work

Patient Questionnaire

Draft 02

Why are we asking you these questions

The ability to work or go about usual activity is an important part of a patient's overall health and quality of life. We are interested to see if there is a change in your occupation before and after your weight loss surgery.

<u>Occupation</u>

1. Please select a category that best describes your current employment status

Working full time (30hrs or more per week)	☐ 1	please go to section A
Working part time (less than 30hrs or more per week)	☐ 2	please go to section A
Unable to work because of illness or disability	☐ 3	please go to section B
Student	☐ 4	please go to section A
Unemployed and looking for work	☐ 5	please go to section B
Carer for children or relative	☐ 6	please go to section B
At home and not looking for paid employment	☐ 6	please go to section B
Other	☐ 6	please go to section B

For peer review only

Section A

1. What is your current occupation? Since many people have more than one job at a given time, we would like to know about the job that is your primary source of income

____

2. Have you had any days off sick in the last month? Yes ☐ 1 No ☐ 2 Not applicable ☐ 3

   a. If Yes, how many days in total (please only include the number of days you were not able to work)? |_|_|_|

3. Are you receiving benefits? Yes ☐ 1 No ☐ 2

Section B

1. Are you receiving benefits? Yes ☐ 1 No ☐ 2

Produced by Robertson Centre for Biostatistics, University of Glasgow

Social security (benefits) questions included in baseline questionnaires/patient reported outcome measures (PROMs).

Why we are asking you these questions?

Weight loss surgery can be expensive, but often the improvements in patients' health are great. This is to be used in health economic analysis which balances the cost of weight loss surgery with the improvements in patients' health and wellbeing and overall cost of bariatric surgery.

Due to the personal nature of these questions, you may choose not to complete them.

I am happy to complete these questions

- ☐ Yes
- ☐ No

If No, please go to the next section.

<u>Benefits</u>

1. In the past three months, you may have received some benefits from the government to support you. In the table below, please tick all benefits you have received in the past 3 months.

- ☐ Attendance Allowance
- ☐ Carer's Allowance
- ☐ Child Tax Credit
- ☐ Council Tax Benefit
- ☐ Disability Living Allowance—caring
- ☐ Disability Living Allowance—mobility
- ☐ Employment and Support Allowance
- ☐ Housing Benefit
- ☐ Income Support
- ☐ Jobseeker's Allowance
- ☐ Pension Credit
- ☐ Statutory Sick Pay
- ☐ State Pension
- ☐ Other (please specify _______________)
- ☐ None

For peer review only

Healthcare utilisation (devices and specialist equipment) questions included in baseline questionnaires/patient reported outcome measures (PROMs).

Why we are asking you these questions?

Some people may require specialist equipment or devices as a result of their weight and changes in requirements for equipment will be considered in the overall cost of weight loss surgery.

<u>**Devices and Specialist Equipment**</u>

1	Do you currently use any aids or specialist equipment?	☐ Yes<br>☐ No<br>If No, move to next section	
1a	Stair lift	☐ Yes<br>☐ No	If Yes, funded by:<br>☐ provided by Social Services<br>☐ provided by hospital<br>☐ bought by you
1b.	Grab rail	☐ Yes<br>☐ No	If Yes, funded by:<br>☐ provided by Social Services<br>☐ provided by hospital<br>☐ bought by you
1c.	Bariatric bed	☐ Yes<br>☐ No	If Yes, funded by:<br>☐ provided by Social Services<br>☐ provided by hospital<br>☐ bought by you
1d.	Bariatric shower stool	☐ Yes<br>☐ No	If Yes, funded by:<br>☐ provided by Social Services<br>☐ provided by hospital<br>☐ bought by you
1e.	Bariatric chair	☐ Yes<br>☐ No	If Yes, funded by:<br>☐ provided by Social Services<br>☐ provided by hospital<br>☐ bought by you
1f.	Wheel chair (bariatric)	☐ Yes<br>☐ No	If Yes, funded by:<br>☐ provided by Social Services<br>☐ provided by hospital<br>☐ bought by you
1g.	Mobility scooter	☐ Yes<br>☐ No	If Yes, funded by:<br>☐ provided by Social Services<br>☐ provided by hospital<br>☐ bought by you
1h.	Dressing aids	☐ Yes<br>☐ No	If Yes, funded by:<br>☐ provided by Social Services<br>☐ provided by hospital<br>☐ bought by you
1i.	Other	☐ Yes<br>☐ No	If Yes, please describe any other devices or specialist equipment you use.<br>_______________<br>_______________<br>Funded by:<br>☐ provided by Social Services<br>☐ provided by hospital<br>☐ bought by you

For peer review only

Supplementary Table 1. STROBE Statement completed checklist (from von Elm *et al.* [1]).

SECTION/TOPIC	ITEM No.	STROBE Recommendation	OUR PAPER
**Title and abstract**	1	(a) Indicate the study's design with a commonly used term in the title or the abstract	(a) See title
		(b) Provide in the abstract an informative and balanced summary of what was done and what was found	(b) See abstract
**Introduction**			
Background / rationale	2	Explain the scientific background and rationale for the investigation being reported	Paragraphs 1-3 provide background and paragraph 4 explains rationale
Objectives	3	State specific objectives, including any prespecified hypotheses	Paragraph 4
**Methods**			
Study design	4	Present key elements of study design early in the paper	Paragraph 3/'Study Design' section
Setting	5	Describe the setting, locations, and relevant dates, including periods of recruitment, exposure, follow-up, and data collection	Paragraphs 4-6/ 'Participant and Centre Eligibility', 'Recruitment Procedures and Consent' and 'Data Collection' sections
Participants	6	(a) Give the eligibility criteria, and the sources and methods of selection of participants. Describe methods of follow-up	(a) Paragraphs 4-6/ 'Participant and Centre Eligibility', 'Recruitment Procedures and Consent' and 'Data Collection' sections
		(b) For matched studies, give matching criteria and number of exposed and unexposed	(b) Not applicable
Variables	7	Clearly define all outcomes, exposures, predictors, potential confounders, and effect modifiers. Give diagnostic criteria, if applicable	Paragraphs 7-9/'Data Collection' section
Data sources / measurement	8	For each variable of interest, give sources of data and details of methods of assessment (measurement). Describe comparability of assessment methods if there is more than one group	Paragraphs 7 and 8/'Data Collection' section
Bias	9	Describe any efforts to address potential sources of bias	Not applicable
Study size	10	Explain how the study size was arrived at	Paragraph 4/'Patient and Centre Eligibility' section, paragraph 1 of 'Results' section

For peer review only

Quantitative variables	11	Explain how quantitative variables were handled in the analyses. If applicable, describe which groupings were chosen and why	'Statistical Analyses' section
Statistical methods	12	(a) Describe all statistical methods, including those used to control for confounding	(a) 'Statistical Analyses' section
		(b) Describe any methods used to examine subgroups and interactions	(b) Not applicable
		(c) Explain how missing data were addressed	(c) 'Statistical Analyses' section
		(d) If applicable, explain how loss to follow-up was addressed	(d) Not applicable
		(e) Describe any sensitivity analyses	(e) Not applicable
**Results**			
Participants	13	(a) Report numbers of individuals at each stage of study— e.g. numbers potentially eligible, examined for eligibility, confirmed eligible, included in the study, completing follow-up, and analysed	(a) Paragraph 1 and Figure 1
		(b) Give reasons for non-participation at each stage	(b) Paragraph 1 and Figure 1
		(c) Consider use of flow diagram	(c) Figure 1
Descriptive data	14	(a) Give characteristics of study participants (e.g. demographic, clinical, social) and information on exposures and potential confounders	(a) Paragraphs 2-5/'Characteristics of recruited and analysed sample', 'Comorbidities', "Health and obesity-related quality of life' and physical activity' sections, Table 1 and Table 2
		(b) Indicate number of participants with missing data for each variable of interest	(b) Paragraphs 2-7, Table 1, Table 2, Supplementary Table 2 and Supplementary Table 3
		(c) Summarise follow-up time (e.g. average and total amount)	(c) Not applicable
Outcome data	15	Report numbers of outcome events or summary measures over time	Table 2, Supplementary Table 2 and Supplementary Table 3
Main results	16	(a) Give unadjusted estimates and, if applicable, confounder-adjusted estimates and their precision (e.g. 95% confidence interval). Make clear which confounders were adjusted for and why they were included	(a) Table 3 and Supplementary Table 4
		(b) Report category boundaries when continuous variables were categorised	(b) Supplementary Table 4

For peer review only

		(c) If relevant, consider translating estimates of relative risk into absolute risk for a meaningful time period	(c) Not applicable
Other analyses	17	Report other analyses done e.g. analyses of subgroups and interactions, and	Not applicable
**Discussion**			
Key results	18	Summarise key results with reference to study objectives	Paragraph 1
Limitations	19	Discuss limitations of the study, taking into account sources of potential bias or imprecision. Discuss both direction and magnitude of any potential bias	Paragraph 5
Interpretation	20	Give a cautious overall interpretation of results considering objectives, limitations, multiplicity of analyses, results from similar studies, and other relevant evidence	'Conclusion'
Generalisability	21	Discuss the generalisability (external validity) of the study results	Paragraph 5
**Other**			
Funding	22	Give the source of funding and the role of the funders for the present study and, if applicable, for the original study on which the present article is based	'Funding' section

Reference

1) von EE, Altman DG, Egger M, Pocock SJ, Gotzsche PC, Vandenbroucke JP. The Strengthening the Reporting of Observational Studies in Epidemiology (STROBE) statement: guidelines for reporting observational studies. *Lancet* 2007; **370**(9596): 1453-1457

For peer review only

Supplementary Table 2. Surgical Obesity Treatment Study (SCOTS) baseline data by body mass index (BMI) group.

			BMI (kg/m²)				
			Group 1	Group 2	Group 3	Group 4	Group 5
		Total Number of Participants Completing Question (N missing)	< 40 N=24	40-44 N=64	45-49 N=64	50-54 N=44	55+ N=53
**Demographics**	Sex: % Male	249 (0)	33.3%	23.4%	31.3%	38.6%	22.6%
	SIMD: % Q1 (Most Deprived)	247 (2)	20.8%	20.3%	25.0%	30.2%	44.2%
**Medical history**	% DVT	249 (0)	8.3%	1.6%	3.1%	4.5%	1.9%
	% Hypertension	249 (0)	45.8%	32.8%	51.6%	45.5%	41.5%
	% Type 2 Diabetes	249 (0)	45.8%	39.1%	60.9%	61.4%	41.5%
	% CVD	249 (0)	16.7%	9.4%	6.3%	6.8%	5.7%
	% Arthritis	249 (0)	16.7%	23.4%	35.9%	22.7%	39.6%
	% Back Problems	249 (0)	41.7%	45.3%	50.0%	38.6%	50.9%
	% Asthma	249 (0)	0.0%	25.0%	29.7%	27.3%	43.4%
	% Migraine	249 (0)	20.8%	10.9%	17.2%	25.0%	28.3%
	% Irritable Bowel Syndrome	249 (0)	16.7%	18.8%	20.3%	18.2%	13.2%
	% Sleep Apnoea	249 (0)	16.7%	20.3%	28.1%	27.3%	35.8%
	% PCOS	167 (10)	46.7%	8.3%	17.1%	20.0%	13.2%
	% Gastroesophageal Reflux	240 (9)	26.1%	38.1%	52.5%	39.5%	36.0%
**Incontinence**	% ICIQ-UI SF Score ≥6	239 (10)	26.1%	42.2%	46.7%	40.5%	54.0%
	% ICIQ-UI SF Score ≥6 (females only)	168 (9)	40.0%	42.9%	50.0%	56.0%	56.4%
	% ICIQ-UI SF Score ≥6 (males only)	71 (1)	0.0%	40.0%	40.0%	17.6%	45.5%
	Median (LQ, UQ) ICIQ-UI SF Score	239 (10)	0.0 (0.0; 6.0)	4.0 (0.0; 9.5)	5.0 (0.0; 8.0)	3.0 (0.0; 12.0)	9.0 (3.0; 13.0)
**Depression**	Mean (SD) PHQ-9 Score	244 (5)	7.4 (6.6)	9.5 (6.4)	9.0 (6.1)	10.9 (6.5)	10.5 (5.9)
	% PHQ-9 Score ≥10	244 (5)	26.1%	46.9%	38.1%	48.8%	51.0%
**Anxiety**	Median (LQ, UQ) GAD-7 Score	243 (6)	4.0 (1.0; 6.0)	5.0 (2.0; 10.5)	5.5 (2.0; 9.0)	4.0 (2.0; 12.0)	7.0 (3.0; 10.0)
	% GAD-7 Score ≥6	243 (6)	30.4%	42.2%	50.0%	39.5%	62.7%
**Smoking Status**	% Current	240 (9)	17.4%	4.8%	4.8%	2.3%	4.0%
	% Former		30.4%	45.2%	46.8%	39.5%	48.0%
	% Never		52.2%	50.0%	48.4%	58.1%	48.0%
**Quality of Life**							
SF-12	Mean (SD) SF-12 PCS	236 (13)	43.5 (11.3)	40.3 (11.0)	37.6 (10.6)	35.3 (11.6)	31.1 (10.3)
	Mean (SD) SF-12 MCS	236 (13)	48.7 (7.7)	44.8 (9.1)	45.7 (11.9)	43.8 (10.8)	46.1 (10.2)
EQ-5D-5L	Median (LQ, UQ) EQ-5D-5L Score	237 (12)	0.8 (0.7; 0.9)	0.7 (0.3; 0.8)	0.6 (0.4; 0.8)	0.6 (0.4; 0.7)	0.5 (0.1; 0.6)
	Mean (SD) EQ-5D-5L VAS	237 (12)	64.9 (22.6)	58.8 (22.3)	56.5 (22.5)	51.7 (18.9)	47.9 (21.8)
IWQOL-Lite (Standardised Scoring)	Mean (SD) Physical Function	243 (6)	35 (24.9)	50.5 (23.3)	54.2 (24.5)	61.1 (21.6)	74.8 (20.6)
	Median (LQ, UQ) Self Esteem	242 (7)	60.7 (42.9; 85.7)	71.4 (53.6; 89.3)	71.4 (39.3; 92.9)	89.3 (71.4; 96.4)	85.7 (71.4; 96.4)
	Mean (SD) Sexual Life	231 (18)	40.8 (33.4)	58.8 (30.0)	54.2 (31.2)	60.4 (34.7)	63.7 (28.8)
	Mean (SD) Public Distress	243 (6)	28.9 (25.7)	48.1 (22.4)	55.9 (25.7)	70.1 (24.1)	76.1 (19.8)
	Mean (SD) Work	236 (13)	31 (30.1)	38.9 (29.3)	40 (29.2)	47.5 (25.1)	55.5 (28.6)
	Mean (SD) Total Score	242 (7)	40.5 (22.5)	53.7 (18.9)	55.3 (22.0)	64.1 (19.0)	71.8 (17.5)

For peer review only

**Healthcare Utilisations**	% Use of Any Aids or Specialist Equipment	232 (17)	9.1%	21.3%	27.9%	25.6%	51.0%
**Social Security**	% Unable to Work Due to Illness or Disability	248 (1)	25.0%	23.4%	23.4%	23.3%	34.0%
	% Disability Living Allowance - Caring	236 (13)	17.4%	17.7%	21.3%	17.5%	18.0%
	% Disability Living Allowance - Mobility	236 (13)	17.4%	19.4%	23.0%	17.5%	20.0%

ICIQ-UI SF score: ≥6 = 'moderate incontinence' [1]
PHQ-9 scores: 0-4 = 'minimal depression'; 5-9 = 'mild depression'; 10-14 = 'moderate depression'; 15-19 = 'moderately severe depression'; 20-27 = 'severe depression' [2]
GAD-7 scores: 0-5 = 'mild anxiety'; 6-10 = 'moderate anxiety'; 11-15 = 'moderately severe anxiety'; 15-21 = 'severe anxiety' [3]

References

1. Avery K, Donovan J, Peters TJ, Shaw C, Gotoh M, Abrams P. ICIQ: A brief and robust measure for evaluating the symptoms and impact of urinary incontinence. *Neurourol Urodyn* 2004; **23**(4)**:** 322–330

2. Spitzer RL, Kroenke K, Williams JBW. Validation and utility of a self-report version of PRIME-MD: The PHQ Primary Care Study. *J Am Med Assoc* 1999; **282**(18): 1737-1744

3. Spitzer RL, Kroenke K, Williams JBW, Löwe B. A brief measure for assessing generalized anxiety disorder: The GAD-7. *Arch Intern Med* 2006; **166**(10): 1092-1097

For peer review only

Supplementary Table 3. Surgical Obesity Treatment Study (SCOTS) baseline data by age group.

			Age (years)				
			Group 1	Group 2	Group 3	Group 4	Group 5
		Total Number of Participants Completing Question (N missing)	< 35 N=36	35-44 N=63	45-49 N=63	50-54 N=43	55+ N=44
**Demographics**	Sex: % Male	249 (0)	22.2%	22.2%	28.6%	30.2%	43.2%
	SIMD: % Q1 (Most Deprived)	247 (2)	40.0%	28.6%	14.5%	39.5%	27.3%
**Medical history**	% DVT	249 (0)	2.8%	3.2%	3.2%	0.0%	6.8%
	% Hypertension	249 (0)	11.1%	36.5%	52.4%	44.2%	63.6%
	% Type 2 Diabetes	249 (0)	30.6%	50.8%	54.0%	44.2%	63.6%
	% CVD	249 (0)	0.0%	6.3%	6.3%	9.3%	18.2%
	% Arthritis	249 (0)	5.6%	15.9%	36.5%	27.9%	59.1%
	% Back Problems	249 (0)	47.2%	50.8%	31.7%	39.5%	65.9%
	% Asthma	249 (0)	30.6%	22.2%	31.7%	20.9%	36.4%
	% Migraine	249 (0)	27.8%	17.5%	20.6%	18.6%	15.9%
	% Irritable Bowel Syndrome	249 (0)	8.3%	27.0%	23.8%	4.7%	15.9%
	% Sleep Apnoea	249 (0)	5.6%	20.6%	28.6%	25.6%	50.0%
	% PCOS	167 (10)	26.9%	26.1%	9.3%	7.1%	12.5%
	% Gastroesophageal Reflux	240 (9)	50.0%	50.0%	36.1%	28.6%	37.2%
**Incontinence**	% ICIQ-UI Score ≥6	239 (10)	18.2%	44.3%	50.8%	50.0%	47.6%
	% ICIQ-UI Score ≥6 (females only)	168 (9)	20.0%	51.1%	55.8%	62.1%	50.0%
	% ICIQ-UI Score ≥6 (males only)	71 (1)	12.5%	21.4%	38.9%	23.1%	44.4%
	Median (LQ, UQ) ICIQ-UI Score	239 (10)	0.0 (0.0; 4.0)	5.0 (0.0; 11.0)	6.0 (0.0; 11.0)	5.5 (3.0; 11.0)	4.5 (0.0; 11.0)
**Depression**	Mean (SD) PHQ-9 Score	244 (5)	9.0 (6.2)	10.3 (6.3)	9.2 (5.9)	9.1 (7.0)	10.2 (6.2)
	% PHQ-9 Score ≥10	244 (5)	42.9%	50.0%	37.7%	37.2%	51.2%
**Anxiety**	Median (LQ, UQ) GAD-7 Score	243 (6)	5.0 (2.0; 10.0)	6.0 (3.0; 12.0)	5.0 (3.0; 8.0)	5.0 (1.0; 11.0)	3.0 (1.0; 8.0)
	% GAD-7 Score ≥6	243 (6)	48.6%	54.8%	42.6%	47.6%	39.5%
**Smoking Status**	% Current	240 (9)	0.0%	6.5%	5.0%	0.0%	14.0%
	% Former		34.4%	45.2%	41.7%	48.8%	46.5%
	% Never		65.6%	48.4%	53.3%	51.2%	39.5%
**Quality of Life**							
SF-12	Mean (SD) SF-12 PCS	236 (13)	41.2 (11.4)	38 (10.9)	36.3 (11.8)	37.7 (11.9)	32.5 (10.1)
	Mean (SD) SF-12 MCS	236 (13)	44.9 (10.9)	44.5 (10.1)	46.2 (9.2)	44.7 (10.5)	46.8 (11.5)
EQ-5D-5L	Median (LQ, UQ) EQ-5D-5L Score	237 (12)	0.7 (0.6; 0.8)	0.6 (0.5; 0.8)	0.6 (0.5; 0.8)	0.7 (0.2; 0.8)	0.4 (0.2; 0.7)
	Mean (SD) EQ-5D-5L VAS	237 (12)	58.8 (20.7)	55.4 (21.7)	57.1 (21.7)	55.4 (25.8)	50.0 (20.4)
IWQOL-Lite (Standardised Scoring)	Mean (SD) Physical Function	243 (6)	49.5 (20.9)	54.0 (26.1)	57.2 (26.4)	57.7 (29.3)	66.0 (20.0)
	Median (LQ, UQ) Self Esteem	242 (7)	85.7 (67.9; 92.9)	85.7 (60.7; 96.4)	75.0 (53.6; 92.9)	71.4 (50.0; 92.9)	58.9 (39.3; 82.1)
	Mean (SD) Sexual Life	231 (18)	54.1 (26.5)	56.7 (32.4)	53.2 (33.7)	59.0 (31.9)	65.0 (31.2)
	Mean (SD) Public Distress	243 (6)	62.7 (25.8)	61.5 (26.0)	58 (26.8)	55.7 (31.7)	52.0 (25.6)
	Mean (SD) Work	236 (13)	44.3 (29.2)	42.7 (28.2)	38.8 (26.0)	47.3 (34.5)	47.1 (29.7)
	Mean (SD) Total Score	242 (7)	57.4 (18.8)	59.4 (21.6)	57.0 (22.7)	58.1 (26.0)	60.3 (18.2)

For peer review only

**Healthcare Utilisations**	% Use of Any Aids or Specialist Equipment	232 (17)	16.1%	14.5%	24.1%	42.5%	53.7%
**Social Security**	% Unable to Work Due to Illness or Disability	248 (1)	11.1%	17.5%	25.4%	28.6%	47.7%
	% Disability Living Allowance - Caring	236 (13)	9.4%	19.4%	17.2%	14.3%	31.0%
	% Disability Living Allowance - Mobility	236 (13)	12.5%	17.7%	17.2%	16.7%	35.7%

ICIQ-UI SF score: ≥6 = 'moderate incontinence' [1]
PHQ-9 scores: 0-4 = 'minimal depression'; 5-9 = 'mild depression'; 10-14 = 'moderate depression'; 15-19 = 'moderately severe depression'; 20-27 = 'severe depression' [2]
GAD-7 scores: 0-5 = 'mild anxiety'; 6-10 = 'moderate anxiety'; 11-15 = 'moderately severe anxiety'; 15-21 = 'severe anxiety' [3]

References

1. Avery K, Donovan J, Peters TJ, Shaw C, Gotoh M, Abrams P. ICIQ: A brief and robust measure for evaluating the symptoms and impact of urinary incontinence. *Neurourol Urodyn* 2004; **23**(4)**:** 322–330
2. Spitzer RL, Kroenke K, Williams JBW. Validation and utility of a self-report version of PRIME-MD: The PHQ Primary Care Study. *J Am Med Assoc* 1999; **282**(18): 1737-1744
3. Spitzer RL, Kroenke K, Williams JBW, Löwe B. A brief measure for assessing generalized anxiety disorder: The GAD-7. *Arch Intern Med* 2006; **166**(10): 1092-1097

For peer review only

Supplementary Table 4. Association of age and body mass index (BMI) with self-reported comorbidities and mental health indicators.

Comorbidities and Mental Health Indicators	Variable	BMI (Model 1[1]) and Age (Model 2[2])		BMI and Age (Model 3[3])	
		Odds Ratio (95% CI)	p-value	Odds Ratio (95% CI)	p-value
**Hypertension (N = 107)**	BMI	0.97 (0.72, 1.31)	0.84	0.89 (0.63, 1.24)	0.49
	Age	2.03 (1.47, 2.79)	<0.0001	2.02 (1.44, 2.85)	<0.0001
**Type 2 Diabetes (N = 124)**	BMI	0.94 (0.69, 1.26)	0.66	0.93 (0.67, 1.30)	0.68
	Age	1.41 (1.06, 1.87)	0.02	1.36 (1.00, 1.85)	0.05
**CVD (N = 20)**	BMI	0.61 (0.31, 1.18)	0.14	0.71 (0.36, 1.42)	0.34
	Age	2.98 (1.52, 5.85)	<0.01	2.27 (1.10, 4.67)	0.03
**Arthritis (N = 73)**	BMI	1.45 (1.05, 2.00)	0.02	1.62 (1.09, 2.40)	0.02
	Age	3.01 (2.00, 4.54)	< 0.0001	3.64 (2.26, 5.88)	< 0.0001
**Back Problems (N = 115)**	BMI	1.09 (0.81, 1.47)	0.57	1.12 (0.81, 1.54)	0.49
	Age	1.21 (0.91, 1.59)	0.19	1.12 (0.83, 1.51)	0.46
**Asthma (N = 70)**	BMI	1.85 (1.32, 2.59)	<0.001	1.81 (1.27, 2.58)	<0.01
	Age	1.12 (0.82, 1.52)	0.49	1.11 (0.79, 1.57)	0.54
**Migraine (N = 49)**	BMI	1.55 (1.09, 2.20)	0.02	1.63 (1.11, 2.38)	0.01
	Age	0.85 (0.60, 1.18)	0.33	0.91 (0.62, 1.33)	0.63
**Irritable Bowel Syndrome (N = 44)**	BMI	0.97 (0.66, 1.44)	0.9	1.00 (0.65, 1.54)	1.00
	Age	0.98 (0.69, 1.41)	0.93	1.07 (0.71, 1.60)	0.76
**Sleep Apnoea (N = 66)**	BMI	1.69 (1.21, 2.36)	<0.01	1.88 (1.27, 2.77)	<0.01
	Age	2.28 (1.55, 3.34)	< 0.0001	2.05 (1.35, 3.11)	<0.001
**PCOS (N = 28)**	BMI	0.72 (0.42, 1.23)	0.23	0.74 (0.42, 1.30)	0.29
	Age	0.56 (0.36, 0.88)	0.01	0.56 (0.35, 0.90)	0.02
**Gastroesophageal Reflux (N = 97)**	BMI	1.02 (0.75, 1.39)	0.88	1.06 (0.76, 1.47)	0.73
	Age	0.73 (0.55, 0.98)	0.04	0.68 (0.50, 0.93)	0.01
**Male Incontinence (ICIQ-UI score ≥ 6) (N = 22)**	BMI	1.58 (0.81, 3.09)	0.18	1.99 (0.92, 4.30)	0.08
	Age	2.05 (1.05, 4.01)	0.04	2.19 (1.06, 4.53)	0.03
**Female Incontinence (ICIQ-UI score ≥ 6) (N = 83)**	BMI	1.45 (1.01, 2.09)	0.05	1.40 (0.94, 2.08)	0.10
	Age	1.59 (1.10, 2.28)	0.01	1.49 (1.02, 2.19)	0.04

For peer review only

**Depression (PHQ-9 score ≥ 10) (N = 107)**	BMI	1.25 (0.92, 1.70)	0.15	1.33 (0.96, 1.86)	0.09
	Age	1.04 (0.79, 1.37)	0.79	0.99 (0.73, 1.34)	0.94
**Anxiety (GAD-7 score ≥ 6) (N = 114)**	BMI	1.42 (1.04, 1.95)	0.03	1.47 (1.05, 2.05)	0.03
	Age	0.91 (0.69, 1.20)	0.49	0.91 (0.67, 1.24)	0.56

[1] Model 1 – Unadjusted model including only the effect of BMI (per 10 kg/m^2) on comorbidity.

[2] Model 2 – Unadjusted model including only the effect of age (per 10 years) on comorbidity.

[3] Model 3 – Model including the effects of age (per 10 years) and BMI (per 10 kg/m^2) on comorbidity, after adjusting additionally for sex, Scottish Index of Multiple Deprivation (SIMD) and smoking status.

Regressions analyses were performed for conditions affecting 10 or more participants only.

For peer review only

BMJ Open

Surgical Obesity Treatment Study (SCOTS), a prospective, observational cohort study on health and socio-economic burden in treatment-seeking individuals with severe obesity in Scotland, U.K.

Journal:	*BMJ Open*
Manuscript ID	bmjopen-2020-046441.R2
Article Type:	Original research
Date Submitted by the Author:	28-Jun-2021
Complete List of Authors:	Mackenzie, Ruth; University of Glasgow Institute of Cardiovascular and Medical Sciences, BHF Glasgow Cardiovascular Research Centre<br>Greenlaw, Nicola; University of Glasgow Robertson Centre for Biostatistics<br>Ali, Abdulmajid; University Hospital Ayr<br>Bruce, Duff; NHS Grampian<br>Bruce, Julie; University of Warwick Warwick Clinical Trials Unit<br>Grieve, Eleanor; University of Glasgow, Institute of Health and Wellbeing<br>Lean, Mike; University of Glasgow School of Medicine Dentistry and Nursing<br>Lindsay, Robert; University of Glasgow Institute of Cardiovascular and Medical Sciences, BHF Glasgow Cardiovascular Research Centre<br>Sattar, Naveed; University of Glasgow, Institute of Cardiovascular and Medical Sciences<br>Stewart, Sally; Glasgow Caledonian University, School of Health and Life Sciences<br>Ford, Ian; University of Glasgow Robertson Centre for Biostatistics<br>Logue, Jennifer; Lancaster University, Lancaster Medical School
**Primary Subject Heading**:	Epidemiology
Secondary Subject Heading:	Surgery
Keywords:	EPIDEMIOLOGY, Adult surgery < SURGERY, Quality in health care < HEALTH SERVICES ADMINISTRATION & MANAGEMENT

SCHOLARONE™ Manuscripts

I, the Submitting Author has the right to grant and does grant on behalf of all authors of the Work (as defined in the below author licence), an exclusive licence and/or a non-exclusive licence for contributions from authors who are: i) UK Crown employees; ii) where BMJ has agreed a CC-BY licence shall apply, and/or iii) in accordance with the terms applicable for US Federal Government officers or employees acting as part of their official duties; on a worldwide, perpetual, irrevocable, royalty-free basis to BMJ Publishing Group Ltd ("BMJ") its licensees and where the relevant Journal is co-owned by BMJ to the co-owners of the Journal, to publish the Work in this journal and any other BMJ products and to exploit all rights, as set out in our licence.

The Submitting Author accepts and understands that any supply made under these terms is made by BMJ to the Submitting Author unless you are acting as an employee on behalf of your employer or a postgraduate student of an affiliated institution which is paying any applicable article publishing charge ("APC") for Open Access articles. Where the Submitting Author wishes to make the Work available on an Open Access basis (and intends to pay the relevant APC), the terms of reuse of such Open Access shall be governed by a Creative Commons licence – details of these licences and which Creative Commons licence will apply to this Work are set out in our licence referred to above.

Other than as permitted in any relevant BMJ Author's Self Archiving Policies, I confirm this Work has not been accepted for publication elsewhere, is not being considered for publication elsewhere and does not duplicate material already published. I confirm all authors consent to publication of this Work and authorise the granting of this licence.

For peer review only

TITLE:

Surgical Obesity Treatment Study (SCOTS), a prospective, observational cohort study on health and socio-economic burden in treatment-seeking individuals with severe obesity in Scotland, U.K.

AUTHORS:

Ruth M. Mackenzie[1], Nicola Greenlaw[2], Abdulmajid Ali[3], Duff Bruce[4], Julie Bruce[5], Eleanor Grieve[6], Mike Lean[7], Robert S. Lindsay[1], Naveed Sattar[1], Sally Stewart[8], Ian Ford[2], Jennifer Logue[9], on behalf of the SCOTS investigators

AFFILIATIONS:

[1]Institute of Cardiovascular and Medical Sciences, University of Glasgow, Glasgow, UK.

[2]Robertson Centre for Biostatistics, University of Glasgow, Glasgow, UK.

[3]University Hospital Ayr, NHS Ayrshire and Arran, Ayr, UK.

[4]NHS Grampian, Aberdeen, UK.

[5]Clinical Trials Unit, University of Warwick, Warwick, UK.

[6]Institute of Health and Wellbeing, University of Glasgow, Glasgow, UK.

[7]School of Medicine, Dentistry and Nursing, University of Glasgow, Glasgow, UK

[8]School of Health and Life Sciences, Glasgow Caledonian University, Glasgow, UK.

[9]Lancaster Medical School, Lancaster University, Lancaster, UK.

For peer review only

CORRESPONDENCE TO:

Jennifer Logue

Lancaster Medical School

Faculty of Health and Medicine

Lancaster University

LA1 4YG

Telephone: 01524 595133

j.logue1@lancaster.ac.uk

WORD COUNT:

4,685 (including strengths and limitations of this study, funding, data availability, competing interests, author contribution and acknowledgements statements)

4,229 (excluding strengths and limitations of this study, funding, data availability, competing interests, author contribution and acknowledgements statements)

For peer review only

ABSTRACT

Objectives: There is a lack of evidence to inform the delivery and follow-up of bariatric surgery for people with severe obesity. The SurgiCal Obesity Treatment Study (SCOTS) is a national longitudinal cohort of people undergoing bariatric surgery. Here we describe characteristics of the recruited SCOTS cohort, and the relationship between health and socio-economic status with body mass index (BMI) and age.

Participants/Methods: 445 participants scheduled for bariatric surgery at any of 14 centres in Scotland, UK were recruited between 2013 and 2016 for this longitudinal cohort study (1 withdrawal); 249 completed health-related preoperative patient reported outcome measures. Regression models were used to estimate the effect of a 10-unit increase in age or BMI, adjusting for sex, smoking and socio-economic status.

Results: Mean age was 46 years and median BMI was 47 kg/m^2. For each 10 kg/m^2 higher BMI there was a change of -5.2 (95% CI -6.9, -3.5; p<0.0001) in Rand 12-item Short Form Survey Physical Component Summary (SF-12 PCS), -0.1 (95% CI -0.2, -0.1; p<0.0001) in EuroQoL 5-level EQ-5D version index score, 14.2 (95% CI 10.7, 17.7; p<0.0001) in Impact of Weight on Quality of Life-Lite Physical Function score. We observed a 3.1 times higher use of specialist aids and equipment at home (OR 3.1, 95% CI 1.9, 5.0; p < 0.0001). Broadly similar results were seen for each 10-year higher age, including a change of -2.1 (95% CI -3.7, -0.5; p<0.01) in SF-12 PCS.

Conclusions: Higher BMI combined with older age is associated with poor physical functioning and quality of life in people seeking bariatric surgery treatment. Policy makers must consider the health and care needs of these individuals and invest to provide increased access to effective weight management.

For peer review only

Trial registration: This study was registered prospectively at the International Standard Randomised Controlled Trials Number registry: ISRCTN47072588

STRENGTHS AND LIMITATIONS OF THIS STUDY

- A limitation is that selection for bariatric surgery is often based on the presence of comorbidity so these results, while applicable to a treatment-seeking population, may not be directly applicable to the population with severe obesity in general.
- The fact that almost every bariatric surgery patient in Scotland was recruited is a major strength of this study and renders it more representative than many studies undertaken in the field.
- A further strength is that the majority of questionnaires used were externally validated and wide-ranging, containing a number of unique questions covering medical, social, psychological and physical domains.

INTRODUCTION

Obesity (body mass index [BMI] ≥ 30kg/m^2) and, in particular, severe obesity (BMI ≥ 40kg/m^2) are associated with a variety of negative health outcomes, including increased risk of most major chronic diseases(1). In recent years, severe obesity has emerged as a major public health concern with rates increasing rapidly in a number of countries across the world, including the United States (US) where the prevalence of BMI > 40 kg/m^2 rose by 70% between 2000 and 2010(2), and around 7.7% of adults are now considered to have severe obesity(3). Similarly, levels of severe obesity have risen in the United Kingdom (UK) with 2.9% of all adults in England(4) and 4% of all adults in Scotland now estimated to have a BMI ≥ 40 kg/m^2(5).

For peer review only - http://bmjopen.bmj.com/site/about/guidelines.xhtml

As the prevalence of severe obesity rises, effective treatment is a priority. The efficacy of bariatric surgery for significant long-term weight loss is well established(6,7). However, for the UK National Health Service (NHS), like many other health systems, the commissioning of bariatric surgery is low priority. Indeed, despite obesity prevalence being amongst the highest in the European Union, the UK performs only nine bariatric surgery procedures per 100 000 people(8,9) while Sweden, a country with a similar health service but lower obesity prevalence, performs 70-80 procedures per 100 000 people(10). In North America, the rate of surgery is around 40-50 per 100 000 people, with the majority of these operations performed in the US(8).

The low prioritisation of bariatric surgery within the UK and the strict criteria for access to surgery, including complex pre-surgical pathways and pre-surgical weight-loss requirements(5), results in low numbers of individuals with severe obesity actually receiving surgery. Those receiving surgery generally do so after many years of alternative conservative interventions, at a point when their mean BMI is extremely high, at around 45 kg/m^2, and they are at a median age of 47 years(11). To date, it is unclear how this delay in treatment impacts on health, physical functioning and quality of life.

The SurgiCal Obesity Treatment Study (SCOTS) is the first national epidemiological study established to investigate long-term outcomes following bariatric surgery. This cohort study collected clinical and patient-reported health outcomes from treatment-seeking individuals from across Scotland with severe obesity before they underwent bariatric surgery(12). The aim of the present study is to describe the health-related characteristics of the recruited SCOTS cohort and to examine relationships between age, preoperative BMI and other health-related factors in our recruited sample of adults with severe obesity from across Scotland.

For peer review only

PARTICIPANTS AND METHODS

For peer review only - http://bmjopen.bmj.com/site/about/guidelines.xhtml

This study was registered prospectively at the International Standard Randomised Controlled Trials Number (ISRCTN) registry: ISRCTN47072588. In reporting our findings, we have adhered to the STROBE (STrengthening the Reporting of OBservational studies in Epidemiology) Statement(13) (Supplementary Table 1).

Ethics

A favourable ethical opinion for this study was obtained from the West of Scotland Research Ethics Committee 4 on 07/02/2013 (13/WS/0005).

Patient Involvement

Patients identified via bariatric surgery peer support groups in Scotland were involved in the design and conduct of this research. During the protocol development stage, patients provided input with regard to data collection and defining research questions. In addition, methods of recruitment were informed by discussions with patients during two focus group sessions. Patients were subsequently involved in reviewing paperwork for recruitment and appeared in the recruitment video. There is also a patient member of the independent study steering committee and patients were invited to a meeting to discuss plans for dissemination of study results.

Study Design

SCOTS is a national, prospective, observational cohort study of adults aged over 16 years who were eligible for primary bariatric surgery in Scotland. Participants were recruited over a four year period from December 2013 to February 2017. Recruitment to the cohort has now closed although follow-up will continue until October 2020. A detailed protocol for the SCOTS study was published previously(12) and is included as Supplementary File 1, but was amended in 2016 to reflect a smaller cohort size and shorter follow-up. This was in response to changes in national service commissioning contributing to lower numbers of bariatric surgery which, in turn, affected recruitment potential and planned sample size. Any changes from the original protocol are outlined below.

For peer review only

Participant and Centre Eligibility

As outlined in the protocol(12) (Supplementary File 1), patients scheduled to undergo a primary bariatric surgery procedure at any of the ten NHS-funded or four private hospitals in Scotland providing this surgery were eligible for invitation to the study. Inclusion criteria were that patients were aged 16 years or over, and undergoing their first bariatric surgery procedure. Patients were required to have capacity to consent, provide written informed consent and be resident in Scotland. Patients who did not meet these criteria or who did not have English language skills to complete written questionnaires were ineligible(12) (Supplementary File 1).

Recruitment Procedures and Consent

Patients were approached about the study at least four weeks prior to their primary bariatric surgical procedure by the clinical bariatric surgery team or by a research nurse in preoperative assessment clinics. Written signed consent for access to medical records, linkage of electronic health records, and for postal questionnaires was obtained on a subsequent clinical visit.

Data Collection

Recruited participants were asked to complete questionnaires preoperatively and at two and three years postoperatively. Prior to receiving questionnaires, participants were made aware (via patient information leaflets) that the estimated time for completion of each questionnaire was one hour. The approximate time it would take to complete the questionnaire (one hour) was also stated clearly on the first page of each questionnaire received by participants. Completion could be either by post or electronically via a secure link sent by email. Two reminders were sent by the participant's chosen method and a third reminder, if required, was sent by post to all participants. No further strategy was used after three reminders.

Baseline preoperative questionnaires collected health-related information, including weight, medical history, smoking status, alcohol use, gastrointestinal symptoms, urological health, depression,

For peer review only

anxiety, health-related quality of life (HRQoL) and obesity-specific quality of life (O-QoL), life optimism, physical activity, healthcare utilisation, employment, and social security. Comorbidity was assessed by self-report using a questionnaire designed specifically for this study (Supplementary File 2). Gastrointestinal symptoms were evaluated using a questionnaire developed for the REFLUX trial(14). Urological health was assessed using the International Prostate Symptom Score (IPSS)(15) and the International Consultation on Incontinence Questionnaire-Urinary Incontinence Short Form (ICIQ-UI SF) wherein a score ≥ 6 indicates moderate incontinence(16). Female reproductive health data was obtained using a modified version of the questionnaire developed for the Longitudinal Assessment of Bariatric Surgery (LABS-2) study(17). Information on male erectile dysfunction was obtained using a modified version of the questionnaire developed for the Massachusetts Male Ageing Study(18). Anxiety and depression were assessed using the Generalised Anxiety Disorder Assessment (GAD-7)(19) and Patient Health Questionnaire (PHQ)-9 (PHQ-9)(20) instruments respectively. A PHQ-9 score ≥ 10 is indicative of moderate to severe depression while a GAD-7 score ≥ 6 is reflective of moderate to severe anxiety(19,20). Smoking status was ascertained using a questionnaire specifically developed for this study (Supplementary File 2). Alcohol use was determined using a modified version of the Alcohol Use Disorders Identification Test (AUDIT)(21). HRQoL was assessed using the Rand 12-item Short Form Survey (SF-12)(22) and EuroQoL 5-level EQ-5D version (EQ-5D-5L)(23,24) instruments while O-QoL was assessed using the Impact of Weight on Quality of Life-Lite (IWQOL-Lite) questionnaire(25). Standardised scoring was used when interpreting IWQOL-Lite questionnaires(26). Life optimism was determined using a modified version of the Life Orientation Test (LOT) wherein a score range of 0-13 reflects low optimism (high pessimism)(27). Data on physical activity was obtained using the International Physical Activity Questionnaire (IPAQ) – Short Form(28). Information on participants’ employment, social security status, and healthcare utilisation was obtained using questionnaires specifically developed for this study (Supplementary File 2).

For peer review only - http://bmjopen.bmj.com/site/about/guidelines.xhtml

Each participant's quintile of the Scottish Index of Multiple Deprivation (SIMD), an area-based measure of socioeconomic status(29), was derived from their postcode. Combining a number of indicators of socioeconomic status across seven domains, the SIMD provides a relative measure of deprivation which can be used to compare data zones by ranking them from most to least deprived. The seven domains include income, employment, health, education, skills and training, housing, geographic access, and crime(29).

Height and weight data were collected by clinical staff at recruitment allowing BMI to be calculated.

We herein report baseline data recorded during the recruitment visit for the whole recruited cohort before bariatric surgery, and patient-reported outcomes for the sub-set completing baseline questionnaires.

Statistical Analyses

Continuous data are reported as means and standard deviations (SD) or medians and lower (Q1) and upper (Q3) quartiles depending on data distribution, and counts and percentages are reported for categorical data. Age and BMI data were categorised *a priori*; BMI (kg/m^2) is reported as 5-unit bands (< 40, 40-44, 45-49, 50-54, ≥ 55) and age was categorised using 10-year age bands (< 35 years, 35-44, 45-49, 50-54 and ≥ 55 years). Other baseline demographics are summarised by group.

Linear regression models were used to examine continuous quality of life measures and logistic regression for the binary 'need for specialist aids' and 'equipment in the home to assist with daily living'. Models were used to estimate the effect of a 10 unit increase in age or BMI in 1) an unadjusted model and 2) in a model adjusted for sex, SIMD, smoking status and BMI or age respectively as these factors may be associated with obesity and related comorbidity. Regression model effect estimates, or odds ratios (ORs), and corresponding 95% confidence intervals (CIs) and associated p-values are provided. Data was analysed as available, without any imputation for missing data. All analyses were performed using SAS (version 9.3).

For peer review only - http://bmjopen.bmj.com/site/about/guidelines.xhtml

RESULTS

Over the three year recruitment period, a total of 548 patients were approached and screened for eligibility to participate. Of these, 103/548 (19%) were excluded or declined to participate (Figure 1). We recruited 445/548 (81%) participants but one participant withdrew consent leaving a recruited sample of 444 (81%). Of the recruited sample, 413/444 (93%) consented to data linkage and questionnaire follow-up whilst 31/444 (7%) consented to data linkage only. Of these 413 participants, a total of 164/413 (40%) were not included in the subsequent analysis: 129 did not return a baseline questionnaire and 35 had bariatric surgery before their baseline patient reported outcome measures (PROMs) questionnaires were completed. Of the 129 who did not return baseline questionnaires, 84/129 (65%) progressed to surgery, 43/129 (33%) did not progress to surgery and the status of 2/129 (2%) was unknown. Completed preoperative baseline PROMs data for 249/413 participants (60% of those consented) were available for analysis (Figure 1).

Characteristics of recruited and analysed sample

Demographic data are summarised in Table 1. Participant characteristics were similar between the total recruited sample (n= 444) and the analysed sub-set (n = 249) with completed PROMS before bariatric surgery (Table 1). Mean age was 46 years (SD 9.1 years) with a higher proportion of women than men (71% vs 29%). Half of recruited participants were aged 35 to 49 years, with one third being over 50 years. The median BMI was 47 kg/m^2 (Q1 43; Q3 54) with more than 21% having a BMI of ≥ 55+ kg/m^2. Over half of the participants (55%) lived in areas of high socio-economic deprivation (SIMD quintiles 1 and 2). There were no statistically significant differences between the analysed sub-set (n = 249) and the non-analysed sub-set (n = 195, data not shown).

For peer review only - http://bmjopen.bmj.com/site/about/guidelines.xhtml

Table 1. Baseline sociodemographic characteristics of recruited (n = 444) and analysed (n = 249) samples of SCOTS participants.

Characteristic		Recruited Sample N = 444	Analysed Sample* N = 249
Sex, N (%)	Male	123 (27.7)	72 (28.9)
	Female	321 (72.3)	177 (71.1)
	Missing	0	0
Age (years)	Mean (SD)	46.2 (9.1)	45.9 (9.1)
	Missing	0	0
Age Group, N (%)	< 35 years	61 (13.7)	36 (14.5)
	35-44 years	116 (26.1)	63 (25.3)
	45-49 years	109 (24.5)	63 (25.3)
	50-54 years	79 (17.8)	43 (17.3)
	55+ years	79 (17.8)	44 (17.7)
	Missing	0	0
BMI (kg/m^2)	Median (Q1, Q3)	47.2 (42.7 ; 53.6)	47.6 (42.8 ; 53.8)
	Missing	1	0
BMI Group, N (%)	BMI < 40	52 (11.7)	24 (9.6)
	BMI 40-44	115 (26.0)	64 (25.7)
	BMI 45-49	116 (26.2)	64 (25.7)
	BMI 50-54	71 (16.0)	44 (17.7)
	BMI 55+	89 (20.1)	53 (21.3)
	Missing	1	0
SIMD Quintile, N (%)	Quintile 1 (most deprived)	135 (30.5)	70 (28.3)
	Quintile 2	108 (24.4)	65 (26.3)
	Quintile 3	84 (19.0)	51 (20.6)
	Quintile 4	68 (15.4)	34 (13.8)
	Quintile 5 (least deprived)	47 (10.6)	27 (10.9)
	Missing	2	2
Marital Status, N (%)	Married/civil partnership/co-habiting	Not collected	155 (63)
	Single/separated/divorced/ widowed		91 (37)
	Missing		3
Ethnic Group, N (%)	White	Not collected	243 (97.6)
	Mixed		4 (1.6)
	Asian/Asian Scottish/Asian British		1 (0.4)
	African Caribbean/Black		1 (0.4)
	Other		0 (0.0)
	Missing		0
Education, N (%)	School only	Not collected	58 (23.5)
	Formal qualifications through training at work		54 (21.9)
	Qualification (other than a degree from college or university)		64 (25.9)
			71 (28.7)

For peer review only

	Degree from college or university<br>Missing		 2
Current Employment Status, N (%)	Working full time<br>Working part time<br>Unable to work because of illness or disability<br>Student/unemployed and seeking employment/ unemployed and not seeking employment/ carer/other<br>Missing	Not collected	124 (50.0)<br>24 (9.7)<br>64 (25.8)<br>36 (14.5)<br>1

Working full time = ≥ 30 hours per week; working part-time = < 30 hours per week. Percentages may not total 100% in all cases due to rounding. *Participants who returned baseline questionnaires prior to their bariatric surgery are included in the analysed sample.

For peer review only

Comorbidities

For the analysed sample (n = 249), self-reported medical comorbidities, and physical, mental and functional measures are presented in Table 2. Over 40% reported having at least one of hypertension, type 2 diabetes (T2D), back problems, anxiety/depression and gastroesophageal reflux. Over 60% of the sample reported more than three comorbidities. Over 40% of male participants reported erectile dysfunction while one third of males described urinary incontinence. Half of female participants reported urinary incontinence. Mean depression scores reflected mild depression, although 44% of participants had scores indicating moderate to severe depression. Anxiety scores for all participants were indicative of mild anxiety (median 5.0); with half of participants having scores indicative of moderate to severe anxiety. The mean life optimism score for participants was reflective of low optimism (high pessimism). Very few participants smoked (5%) and, on average, alcohol consumption was moderate.

For peer review only

For peer review only - http://bmjopen.bmj.com/site/about/guidelines.xhtml

Table 2. Preoperative health-related characteristics of SCOTS participants undergoing bariatric surgery (n = 249).

		N = 249<br>N (%)	Missing<br>N (%)
**Comorbidity, Self-report**	DVT	8 (3.2)	0
	Pulmonary embolism	4 (1.6)	0
	Hypertension	107 (43.0)	0
	Type 2 diabetes	124 (49.8)	0
	Angina/heart attack	17 (6.8)	0
	Heart failure	2 (0.8)	0
	Stroke/mini stroke	6 (2.4)	0
	Arthritis	73 (29.3)	0
	Back problems	115 (46.2)	0
	Chronic bronchitis	4 (1.6)	0
	Eczema/psoriasis	33 (13.3)	0
	Asthma	70 (28.1)	0
	Thyroid problems	32 (12.9)	0
	Migraine	49 (19.7)	0
	Anxiety/depression	114 (45.8)	0
	Kidney disease	7 (2.8)	0
	Liver disease	2 (0.8)	0
	Cancer	4 (1.6)	0
	Irritable bowel syndrome	44 (17.7)	0
	Sleep apnoea	66 (26.5)	0
	CVD	20 (8.0)	0
**N (%) Self-reported Comorbidities**	None	9 (3.6)	0
	1-2	80 (32.1)	0
	≥ 3	160 (64.3)	0
**Gastrooesophageal Reflux**	Yes	97 (40.4)	9 (3.6)
**Female Reproductive Health N = 75***	Mean (SD) age years, last natural menstrual period	39.4 (10.9)	4 (5.3)
**Female Reproductive Health N = 177**	PCOS, N (%)	28 (16.8)	10 (5.6)
**Male Reproductive Health N = 72**	Impotence, N (%)	28 (41.2)	4 (5.6)
	IPSS score ≥8, N (%)	34 (47.9)	1 (1.4)
**Incontinence**	Median (Q1, Q3) ICIQ-UI SF score	4 (0.0; 10.0)	10 (4.0)
	ICIQ-UI SF score ≥6	105 (43.9)	10 (4.0)
Incontinence, Females N = 177	ICIQ-UI SF score ≥6, N (%)	83 (49.4)	9 (5.1)
Incontenence, Males N = 72	ICIQ-UI SF score ≥6, N (%)	22 (31.0)	1 (1.4)
**Depression**	Mean (SD) PHQ-9 score	9.6 (6.3)	5 (2.0)
	N (%) PHQ-9 score ≥10	107 (43.9)	5 (2.0)
**Anxiety**	Median (Q1, Q3) GAD-7	5 (2.0; 9.0)	6 (2.4)
	N (%) GAD-7 score ≥6	114 (46.9)	6 (2.4)

For peer review only

**Smoking Status**	Current	13 (5.4)	9 (3.6)
	Former	105 (43.8)	
	Never	122 (50.8)	
**Alcohol Use**	Median (Q1, Q3) AUDIT	3 (1.0; 6.0)	20 (8.0)
**Quality of Life**			
SF-12	Mean (SD) PCS	37 (11.4)	13 (5.2)
	Mean (SD) MCS	45.5 (10.3)	13 (5.2)
EQ-5D-5L	Median (Q1, Q3)	0.6 (0.3; 0.8)	12 (4.8)
	Mean (SD) VAS	55.3 (22.1)	12 (4.8)
IWQOL-Lite (Standardised Scoring)	Mean (SD) Physical Function	56.9 (25.4)	6 (2.4)
	Mean (SD) Self Esteem	70.7 (27.1)	7 (2.8)
	Mean (SD) Sexual Life	57.1 (31.7)	18 (7.2)
	Mean (SD) Public Distress	58.1 (27.2)	6 (2.4)
	Mean (SD) Work	43.6 (29.2)	13 (5.2)
	Mean (SD) Total score	58.5 (21.7)	7 (2.8)
**Life Optimism**	Mean (SD) LOT score	13 (4.9)	14 (5.6)
**Physical Activity**	≥1 walking, moderate or vigorous activity in last 7 days	201 (83.4)	8 (3.2)
	Median (Q1, Q3) IPAQ score**	720 (40.0; 1800.0)	6 (3.0)
**Healthcare Utilisations**	Using any aids or specialist equipment	67 (28.9)	17 (6.8)
	Median (Q1, Q3) GP visits in last 3 months	2 (1.0; 3.0)	79 (31.7)
	Median (Q1, Q3) visits to other health/social care providers in last 3 months	3 (1.0; 5.0)	73 (29.3)
**Social Security**	Unable to work due to illness or disability	64 (25.8)	1 (0.4)
	Receiving Disability Living Allowance (caring)	44 (18.6)	13 (5.2)
	Receiving Disability Living Allowance (mobility)	47 (19.9)	13 (5.2)

DVT, deep vein thrombosis; CVD, cardiovascular disease; PCOS, polycystic ovary syndrome; GP, general practitioner. IPSS Scores: 0-7 = 'Mild symptoms'; 8-19 = 'Moderate symptoms'; 20-35 = 'Severe symptoms' [15]. ICIQ-UI SF score: ≥6 = 'moderate incontinence' [16]. PHQ-9 scores: 0-4 = 'minimal depression'; 5-9 = 'mild depression'; 10-14 = 'moderate depression'; 15-19 = 'moderately severe depression'; 20-27 = 'severe depression' [20]. GAD-7 scores: 0-5 = 'mild anxiety'; 6-10 = 'moderate anxiety'; 11-15 = 'moderately severe anxiety'; 15-21 = 'severe anxiety'[19]. * 75/177 (42%) female participants reported not menstruating in the last 12 months. ** MET minutes/week.

For peer review only - http://bmjopen.bmj.com/site/about/guidelines.xhtml

256 **Health and obesity-related quality of life**

257 Mean SF-12 Physical Component Summary (PCS) and Mental Component Summary (MCS) scores
258 were low; PCS 37.0 (11.4), MCS 45.5 (10.3). The median EQ-5D-5L score of sample participants was
259 0.6 (0.3; 0.8) while the mean EQ-5D-5L visual analogue scale (VAS) was 55.3 (22.1). Participants had
260 a mean IWQOL-Lite Physical Function score of 56.9 (25.4) and a mean total score of 58.5 (21.7)
261 (Table 2) where an increase in IWQOL-Lite score indicates a worsening in quality of life.

262 **Physical activity**

263 Over 80% of SCOTS participants reported undertaking at least 10 minutes of either walking,
264 moderate or vigorous activity in the last seven days and the median IPAQ score for the sample was
265 720.0 MET minutes/week. Almost one third (29%) of participants reported using aids or specialist
266 equipment to assist with their daily activities in the home (Table 2).

267 **Comorbidity by BMI and age**

268 Comorbidity data is presented by BMI group and age group in Supplementary Tables 2 and 3
269 respectively. In order to further investigate the associations between BMI and age on physical,
270 mental and functional measures, and healthcare utilisation within the SCOTS population, regression
271 analyses were performed (Table 3). There was no significant correlation between BMI and age
272 (correlation = 0.0075, p = 0.9061). Higher BMI values and higher ages were negatively associated
273 with physical, but not mental, HRQoL scores (Table 3). For each 10 kg/m^2 higher BMI there was a
274 change of -5.2 (95% CI -6.9, -3.5; p<0.0001) in SF-12 PCS, -0.1 (95% CI -0.2, -0.1; p<0.0001) in EQ-5D-
275 5L score and 14.2 (95% CI 10.7, 17.7; p<0.0001) in IWQOL-Lite Physical Function score (where an
276 increase in score indicates a worsening). We observed a 3.1 times higher use of specialist aids and
277 equipment in the home (OR 3.1, 95% CI 1.9, 5.0; p < 0.0001), adjusting for age, sex, smoking and
278 socio-economic deprivation. For each 10-year higher age, there was a change of -2.1 (95% CI -3.7, -
279 0.5; p<0.01) in SF-12 PCS score, -0.1 (95% CI -0.1, 0.0; p<0.01) in EQ-5D-5L score and 5.01 (95% 1.8,

For peer review only

For peer review only - http://bmjopen.bmj.com/site/about/guidelines.xhtml

8.3; p<0.01) in IWQOL-Lite Physical Function score, and a 3.1 (OR 3.1, 95% CI 1.9, 5.0; p<0.0001) times higher use of specialist aids or equipment in the home, adjusting for BMI, sex, smoking and socio-economic status.

Interactions were explored between smoking and both age and BMI (with smoking as a two-level variable [smoked or never smoked] due to small numbers in the current smoker group) and a borderline significant interaction between age and smoking status was observed (Supplementary Table 4). Further exploration in the sub-populations of smokers (current or former) and those who had never smoked, revealed a significant effect of age on the use of specialist aids or equipment in the home in both sub-populations, but the odds ratio suggests a trend towards a slightly larger odds in those participants who had never smoked (Supplementary Table 4). No significant effect of age or BMI on moderate to severe depression (PHQ-9) was observed in either the unadjusted or adjusted models. However, on extending that model to include the interactions between smoking and each of age and BMI, we observe a significant interaction between BMI and smoking status. Considering the sub-populations of smokers and those who had never smoked, there was no significant effect of BMI on smokers, but there was a significant effect of BMI in those who never smoked, with increasing BMI having increased odds of moderate to severe depression (Supplementary Table 4).

With regard to medical comorbidities, as shown in Supplementary Table 5, higher BMI had a significant association with higher prevalence of asthma in the SCOTS population, while older age was associated with higher prevalence of hypertension, arthritis and sleep apnoea (Supplementary Table 5).

For peer review only - http://bmjopen.bmj.com/site/about/guidelines.xhtml

Table 3. Association of age and body mass index (BMI) with quality of life (QoL), use of specialist equipment in the home and social security.

QoL Indicators and Functional Measures	Variable	Unadjusted Models[1]	Adjusted Model[2]
		Regression Coefficient (95% CI)[3]	Regression Coefficient (95% CI)[3]
**SF-12 PCS**	BMI	-4.91 (-6.55, -3.28)	-5.21 (-6.90, -3.52)
	Age	-2.44 (-4.03, -0.85)	-2.14 (-3.73, -0.54)
**SF-12 MCS**	BMI	-0.49 (-2.07, 1.09)	-0.40 (-2.06, 1.26)
	Age	0.42 (-1.04, 1.87)	0.68 (-0.89, 2.25)
**EQ-5D-5L Score**	BMI	-0.11 (-0.15, -0.06)	-0.11 (-0.16, -0.06)
	Age	-0.08 (-0.12, -0.03)	-0.07 (-0.11, -0.02)
**EQ-5D-5L VAS**	BMI	-7.08 (-10.38, -3.79)	-7.73 (-11.10, -4.36)
	Age	-2.19 (-5.30, 0.92)	-0.65 (-3.77, 2.46)
**IWQOL-Lite Physical Function**	BMI	13.72 (10.28, 17.17)	14.20 (10.69, 17.70)
	Age	5.77 (2.28, 9.26)	5.01 (1.75, 8.27)
**IWQOL-Lite Self Esteem**	BMI	5.09 (1.02, 9.16)	5.99 (1.86, 10.12)
	Age	-4.74 (-8.51, -0.97)	-5.11 (-8.96, -1.25)
**IWQOL-Lite Sexual Life**	BMI	5.56 (0.71, 10.41)	5.74 (0.82, 10.67)
	Age	3.86 (-0.75, 8.47)	3.01 (-1.71, 7.73)
**IWQOL-Lite Public Distress**	BMI	15.36 (11.72, 19.00)	16.07 (12.34, 19.80)
	Age	-2.78 (-6.57, 1.02)	-3.04 (-6.51, 0.44)
**IWQOL-Lite Work**	BMI	9.54 (5.25, 13.84)	9.59 (5.16, 14.02)
	Age	1.95 (-2.25, 6.16)	1.04 (-3.15, 5.24)
**IWQOL Total Score**	BMI	10.31 (7.29, 13.33)	10.88 (7.79, 13.97)
	Age	1.20 (-1.84, 4.24)	0.55 (-2.33, 3.43)
**Use of Aids or Specialist Equipment**	BMI	2.34 (1.62, 3.39)*	3.10 (1.94, 4.95)*
	Age	2.65 (1.76, 4.00)*	3.10 (1.94, 4.95)*
**Disability Living Allowance (Caring)**	BMI	1.19 (0.82, 1.73)*	1.07 (0.72, 1.59)*
	Age	1.54 (1.03, 2.31)*	1.62 (1.08, 2.46)*
**Disability Living Allowance (Mobility)**	BMI	1.18 (0.82, 1.70)*	1.13 (0.77, 1.65)*
	Age	1.64 (1.10, 2.45)*	1.62 (1.08, 2.44)*

[1] Unadjusted models including only the effect of BMI (per 10 kg/m^2) or age (per 10 years) on QoL indicators and functional measures. [2] Adjusted model including the effects of BMI (per 10 kg/m^2) and age (per 10 years) on QoL indicators and functional measures, after adjusting additionally for sex, Scottish Index of Multiple Deprivation (SIMD) and smoking status. [3] Regression coefficient (95% CI) is Estimate (95% CI) for results from the linear regression and Odds Ratio (95% CI) for results from the logistic regression. Odds Ratio results are indicated with a *. N ≥ 10 for all indicators and measures for which regression analyses were performed.

For peer review only

For peer review only - http://bmjopen.bmj.com/site/about/guidelines.xhtml

DISCUSSION

Despite escalating levels of severe obesity in Western society and the concomitant increase in bariatric surgical procedures being performed in some countries(30), there is a dearth of information on the health status of people living with severe obesity. This is the first report from the national Scottish cohort study of people seeking surgical treatment for severe obesity. We recruited 444 adults from 14 centres across Scotland over a three year period, including all NHS centres and major private hospitals undertaking bariatric surgery. We found that higher BMI and older age were associated with decreased physical quality of life, increased use of specialist aids and equipment in the home, and a high prevalence of comorbidities.

There has been a significant increase in the prevalence of a BMI ≥ 40kg/m^2 in recent decades; in Scotland, obesity prevalence has trebled in women age 16-64 since 1995. However, it is hard to assess the global increase due to lack of reporting of BMI ≥ 40kg/m^2 in national health survey data(31). While it is known that health care resource use increases in people with a BMI ≥ 30kg/m^2 , with service use estimated to be over 25% higher than for those with a BMI in the normal weight range(32), few data exist for those with BMI 40kg/m^2 and above. In 2016, the Global BMI Mortality Collaboration(33) conducted an individual-participant-data meta-analysis of 239 prospective studies and found a 2.8 times increased risk of all cause mortality for people with a BMI of 40-60kg/m^2. Greive *et al.*(34) conducted a systematic review which focussed on the economic cost of severe obesity (BMI ≥ 40kg/m^2) and found limited literature describing increased prescribing, outpatient utilisation and intensive care admission, and hospital length of stays during critical illness. However, in both studies there was no dissagregation of BMI beyond > 40kg/m^2 meaning that the health consequences of severe obesity are not yet fully described.

There has been extensive research on the relationship between health-related quality of life and obesity(35). Ul-Haq *et al.*(36) performed a meta-analysis of eight studies (43 086 particpants) and found physical quality of life, measured by the Rand 36-item Short Form Health Survey (SF-36), was

For peer review only - http://bmjopen.bmj.com/site/about/guidelines.xhtml

reduced by 9.7 points in those with BMI 40kg/m^2 compared to those with a BMI in the normal range, although, again, there was no disaggregation above BMI 40kg/m^2. Van Nunen *et al.*(37) perfomed a meta-analysis to compare the general, non-treatment seeking population to patients within weight management programmes and those seeking bariatric surgery. They found that those seeking surgical treatment reported the most severely reduced HRQoL, perhaps reflecting their reasons for seeking definitive surgical treatment. Our cohort of treatment-seeking individuals who completed a rich battery of patient reported measures, provides data to show that health-related and obesity-related quality of life of those with the highest body mass is extremely poor and this is compounded by increasing age. Quality of life scores of SCOTS participants in both the upper BMI (≥ 55 kg/m^2) and older age (≥ 55 years) groups included physical scores comparable with those reported by cancer patients receiving palliative care(38), patients with chronic heart failure expressing end of life preferences(39), and patients with end stage kidney disease(40). Furthermore, patients with severe chronic obstructive pulmonary disease (COPD) report higher quality of life scores, indicating a better quality of life, than our cohort of treatment-seeking obese participants(41). As far as we are aware, this is the first study to investigate physical and mental health in patients with severe obesity awaiting bariatric surgery with finer level consideration of BMI up to ≥ 55kg/m^2.

UK guidelines(42) currently indicate that bariatric surgery is a treatment option for those with a BMI ≥ 40 kg/m^2, or between 35 kg/m^2 and 40 kg/m^2 in the presence of other significant diseases (T2D or hypertension, for example) which could be improved if they lost weight. Non-surgical weight management must have been attempted but not resulted in clinically beneficial weight loss before surgery is indicated. However, baseline SCOTS data appear to suggest that the low prioritisation of bariatric surgery and a lengthy preoperative pathway in the UK is associated with surgical treatment being reserved for individuals at an older age with very high BMI. Indeed, in 2018, the Global Registry initiative of the International Federation for the Surgery of Obesity and Metabolic Disorders (IFSO) reported a global median pre-bariatric surgery BMI of 41.7 kg/m^2(43), as compared to 47.6 kg/m^2 in the SCOTS cohort. Similarly, IFSO reported a median patient age of 42 years at the time of

For peer review only - http://bmjopen.bmj.com/site/about/guidelines.xhtml

bariatric surgery(43), as compared to a median age of 47 years for SCOTS participants. This combination of higher BMI and older age means that, at the time of surgery, Scottish patients have high levels of comorbidity and poor physical functioning. Bariatric surgery is considered a highly cost-effective intervention(44). However, the health economic models rely on data primarily from US and Scandinavian studies(45–49), where BMI and age at the time of surgery are lower than in the UK. Higher BMI and older age are risk factors for post-operative complications(50,51) and also associated with lower total weight loss(52–54). Type 2 diabetes remission rates are negatively correlated with age(55). As such, focussing bariatric surgery provision on those with older age and higher BMI may result in higher costs of surgery with increased length of hospital stay, higher rates of post-operative complications, lower overall weight loss and lower rates of disease remission. Consequently, the impressive health benefits and resultant cost-savings of bariatric surgery observed in clinical trials and observational cohorts from other countries may not be fully realised for the UK/Scottish population.

The SCOTS dataset represents a unique and rich resource. A major strength of the study is its representativeness. Indeed, every clinical team providing publicly-funded bariatric surgery in Scotland approached their patients for recruitment to the study, rendering it highly representative of the population in comparison to other studies undertaken in the field. However, the number of participants with valid baseline questionnaires was lower than anticipated. In many cases this could be attributed to the participant undergoing surgery before completing the questionnaire, or the participant leaving the bariatric surgery pathway before surgery. The overall length of the questionnaire may have also played a role. Participants living in the most deprived areas were well represented in our cohort and the mean quality of life findings were broadly similar to those of bariatric surgery cohorts from across the world(56–58). A further strength of the study is that questionnaires were externally validated and wide-ranging, containing a number of unique questions covering medical, social, psychological and physical functioning domains. This wide range of self-reported health measures will allow us to account for a range of potentially mediating and

For peer review only - http://bmjopen.bmj.com/site/about/guidelines.xhtml

confounding factors in future analyses. In addition, we have revealed the extent of comorbidities, including musculoskeletal, urinary and mental health problems affecting people with severe obesity. Low numbers of some comorbidities meant that this could not be a focus of this analysis.

A limitation of this study is that selection for bariatric surgery is often based on the presence of comorbidity so these results, while applicable to a treatment-seeking population, may not be directly applicable to the whole population with severe obesity in the wider society. While we will have access to medical records via electronic health record data linkage in follow-up, the current analyses are based on self-report of selected comorbidities. It is well known that self-reported weights are underreported, particularly by people with very high BMI(59). However, we are confident of the accuracy of weight and height as these data were collected in clinic during the recruitment visit. With regard to future analyses, follow-up of the SCOTS cohort is ongoing which will allow us to report the longitudinal health trajectory after bariatric surgery. The electronic health record infrastructure in Scotland(60) will also permit us to study health outcomes of SCOTS participants across a range of clinical events and, ultimately, determine the effect of bariatric surgery and other factors associated with health outcomes.

Obesity is a multisystem disease which affects every facet of a person’s life. Our data have shown that higher body mass index combined with older age is associated with very poor physical functioning, and health-related and obesity-related quality of life. Indeed, quality of life scores for those living with severe obesity in Scotland are akin to those seen in the end stage of diseases such as cancer and heart failure. The health consequences of severe obesity and the extent to which treatments such as bariatric surgery can improve these are not yet known. Researchers should ensure that they include people with severe obesity in population cohorts and treatment studies, and study the impact of severe obesity in more detail; there are substantial differences in the health

For peer review only

For peer review only - http://bmjopen.bmj.com/site/about/guidelines.xhtml

status of those with a BMI above 50kg/m^2 and those whose BMI is around 40kg/m^2. Policy makers should consider the health and care needs of the growing numbers of inidviduals living with obesity. There will be considerable future demand for health care and services must be designed to accomodate the physical needs of the individuals. While primary prevention of obesity is clearly paramount to avoid more people developing such a debilitating, chronic condition, investment is urgently needed, both in the UK and globally, to provide increased access to bariatric surgery and other forms of effective weight management, directly targeting patient groups who will benefit from surgical intervention as early in the disease course as possible.

FUNDING

This work was supported by the National Institute for Health Research (NIHR) Health Technology Assessment Programme (project number 10/42/02). The views expressed are those of the authors and not necessarily those of the NIHR or the Department of Health and Social Care. Professor J. Bruce is supported from NIHR Research Capability Funding via University Hospitals Coventry and Warwickshire.

DATA AVAILABILITY

Deidentified participant data will be available upon reasonable request by bona fide researchers after completion of the study and our current funded analyses (estimated early 2022). Data requests should be submitted in the first instance to j.logue1@lancaster.ac.uk and will be reviewed by the study's publication committee.

COMPETING INTERESTS

For peer review only

For peer review only - http://bmjopen.bmj.com/site/about/guidelines.xhtml

Professor Lean has received advisory board and speaker fees from Eli Lilly, Novo Nordisk, Merck and Sanofi. Dr Lindsay has received advisory board and speaker fees from Eli Lilly, Novo Nordisk and Servier. Professor Sattar has consulted for Eli Lilly, Novo Nordisk, Pfizer and Sanofi and received compensation. All other authors declare no potential competing interests.

AUTHOR CONTRIBUTION

RMM, NG, AA, DB, JB, EG, ML, RSL, NS, SS, IF and JL were involved in the conception, planning and design of the study, revising it critically and approving the final version for submission. NG conducted the analyses. RMM and JL wrote the first draft. All authors agree to be accountable for all aspects of the work and to ensure that questions relating to the accuracy or integrity of any part of the work are appropriately investigated and resolved.

ACKNOWLEDGEMENTS

These data have been made available for analysis by members of the SCOTS Investigators group, Mr Afshin Alijani, Dr Ewan Bell, Professor Andrew Collier, Ms Carol Craig, Dr Jennifer Darrion, Mr David Galloway, Mr Hasan Kasem, Ms Joeleen McKean, Mr Stuart Oglesby, Mr Chris Shearer, Mr Rob Stuart and Mr Bruce Tulloh, due to their hard work in collecting data, and the patients who have participated in the study. The authors also wish to thank Mr Richard Welbourn, Ms Catherine Quinn, Professor David Beard, Dr John Mooney, Ms Elizabeth Thompson, Mrs Joanne O'Donnell, Mrs Jane Munro, Dr Iona Donnelly and Dr Samantha Alvarez Madrazo.

REFERENCES

1 Kitahara CM, Flint AJ, Berrington de Gonzalez A, Bernstein L, et al. Association between Class

For peer review only

III Obesity (BMI of 40-59 kg/m2) and Mortality: A Pooled Analysis of 20 Prospective Studies. *PLoS Med* 2014;11(7):e1001673

2 Sturm R, Hattori A. Morbid obesity rates continue to rise rapidly in the United States. *Int J Obes* 2013;37(6):889-891

3 Flegal KM, Kruszon-Moran D, Carroll MD, et al. Trends in obesity among adults in the United States, 2005 to 2014. *JAMA* 2016;315(21):2284-2291

4 Baker C. House of Commons Library | Obesity Statistics. *Obes Stat (Briefing Pap Number 3336)* 2019;3336:1-18

5 Grieve E, Mackenzie RM, Munro J, et al. Variations in bariatric surgical care pathways: A national costing study on the variability of services and impact on costs. *BMC Obes* 2018;5(1):43

6 Colquitt JL, Pickett K, Loveman E, et al. Surgery for weight loss in adults. *Cochrane Database Syst Rev* 2014;issue 8:CD003641

7 O'Brien PE, McPhail T, Chaston TB, et al. Systematic review of medium-term weight loss after bariatric operations. *Obes Surg* 2006;16:1032-1040

8 Angrisani L, Santonicola A, Iovino P, et al. Bariatric Surgery Worldwide 2013. *Obes Surg* 2015;25(10):1822-1832

9 Welbourn R, Le Roux CW, Owen-Smith A, et al. Why the NHS should do more bariatric surgery; how much should we do? *BMJ (Online)* 2016;353:i1472

10 Hedenbro JL, Näslund E, Boman L, et al. Formation of the Scandinavian Obesity Surgery Registry, SOReg. *Obes Surg* 2015;25(10):1893-1900

11 Booth HP, Khan O, Fildes A, et al. Changing Epidemiology of Bariatric Surgery in the UK: Cohort Study Using Primary Care Electronic Health Records. *Obes Surg* 2016;26(8):1900-1905

For peer review only

For peer review only - http://bmjopen.bmj.com/site/about/guidelines.xhtml

12 Logue J, Stewart S, Munro J, et al. SurgiCal obesity treatment study (SCOTS): Protocol for a national prospective cohort study of patients undergoing bariatric surgery in Scotland. *BMJ Open* 2015;5(5):e008106

13 von Elm E, Altman DG, Egger M, et al. The Strengthening the Reporting of Observational Studies in Epidemiology (STROBE) statement: guidelines for reporting observational studies. *Lancet* 2007;370(9596):1453-1457

14 Macran S, Wileman S, Barton G, et al. The development of a new measure of quality of life in the management of gastro-oesophageal reflux disease: The Reflux questionnaire. *Qual Life Res* 2007;16(2):331-343

15 Barry MJ, Fowler FJ, O'leary MP, et al. The American Urological Association Symptom Index for Benign Prostatic Hyperplasia. *J Urol* 2017;197(2):S189-S197

16 Avery K, Donovan J, Peters TJ, et al. ICIQ: A brief and robust measure for evaluating the symptoms and impact of urinary incontinence. *Neurourol Urodyn* 2004;23(4):322–330

17 Gosman GG, King WC, Schrope B, et al. Reproductive health of women electing bariatric surgery. *Fertil Steril* 2010;94(4):1426-1431

18 Feldman HA, Goldstein I, Hatzichristou DG, et al. Impotence and its medical and psychosocial correlates: Results of the Massachusetts Male Aging Study. *J Urol* 1994;151(1):54-61

19 Spitzer RL, Kroenke K, Williams JBW, et al. A brief measure for assessing generalized anxiety disorder: The GAD-7. *Arch Intern Med* 2006;166(10):1092-1097

20 Spitzer RL, Kroenke K, Williams JBW. Validation and utility of a self-report version of PRIME-MD: The PHQ Primary Care Study. *J Am Med Assoc* 1999;282(18):1737-1744

21 Reinert DF, Allen JP. The alcohol use disorders identification test (AUDIT): A review of recent research. *Alcohol Clin Exp Res* 2002;26(2):272–279

For peer review only

For peer review only - http://bmjopen.bmj.com/site/about/guidelines.xhtml

22 Ware JE, Kosinski M, Keller SD. A 12-Item Short-Form Health Survey: Construction of Scales and Preliminary Tests of Reliability and Validity. *Med Care* 1996;34(3):220-233

23 Herdman M, Gudex C, Lloyd A, et al. Development and preliminary testing of the new five-level version of EQ-5D (EQ-5D-5L). *Qual Life Res* 2011;20(10):1727-1736

24 Fermont JM, Blazeby JM, Rogers CA, et al. The EQ-5D-5L is a valid approach to measure health related quality of life in patients undergoing bariatric surgery. *PLoS One* 2017;12(12):e0189190

25 Kolotkin RL, Crosby RD. Psychometric evaluation of the impact of weight on quality of life-lite questionnaire (IWQOL-Lite) in a community sample. *Qual Life Res* 2002;11(2):157-171

26 Kolotkin RL, Crosby RD. The Impact of Weight on Quality of Life-Lite (IWQOL-Lite): User's Manual. Durham, NC: Obesity and Quality of Life Consulting 2002

27 Scheier MF, Carver CS, Bridges MW. Distinguishing Optimism From Neuroticism (and Trait Anxiety, Self-Mastery, and Self-Esteem): A Reevaluation of the Life Orientation Test. *J Pers Soc Psychol* 1994;67(6):1063-1078

28 Craig CL, Marshall AL, Sjöström M, et al. International physical activity questionnaire: 12-Country reliability and validity. *Med Sci Sports Exerc* 2003;35(8):1381-1395

29 Scottish Index of Multiple Deprivation 2016: introductory booklet. 2016. https://www.gov.scot/publications/scottish-index-multiple-deprivation-2016/ (accessed January 2020)

30 Arterburn DE, Courcoulas AP. Bariatric surgery for obesity and metabolic conditions in adults. *BMJ (Online)* 2014;349:g3961

31 Williamson K, Nimegeer A, Lean M. Rising prevalence of BMI ≥40 kg/m^2: A high-demand epidemic needing better documentation. *Obes Rev* 2020;21(4):e12986

For peer review only

For peer review only - http://bmjopen.bmj.com/site/about/guidelines.xhtml

32 le Roux CW, Chubb B, Nørtoft E, et al. Obesity and healthcare resource utilization: results from Clinical Practice Research Database (CPRD). *Obes Sci Pract* 2018;4(5):409-416

33 Di Angelantonio E, Bhupathiraju SN, Wormser D, et al. Body-mass index and all-cause mortality: individual-participant-data meta-analysis of 239 prospective studies in four continents. *Lancet* 2016;388(10046):776-786

34 Grieve E, Fenwick E, Yang HC, et al. The disproportionate economic burden associated with severe and complicated obesity: A systematic review. *Obes Rev* 2013;14(11):883-894

35 Kolotkin RL, Andersen JR. A systematic review of reviews: exploring the relationship between obesity, weight loss and health-related quality of life. *Clin Obes* 2017;7(5):273-289

36 Ul-Haq Z, Mackay DF, Fenwick E, et al. Meta-analysis of the association between body mass index and health-related quality of life among adults, assessed by the SF-36. *Obesity* 2013;21(3):E322-E327

37 Van Nunen AMA, Wouters EJM, Vingerhoets AJJM, et al. The health-related quality of life of obese persons seeking or not seeking surgical or non-surgical treatment: A meta-analysis. *Obes Surg* 2007;17(10):1357-1366

38 Radbruch L, Sabatowski R, Loick G, et al. Cognitive impairment and its influence on pain and symptom assessment in a palliative care unit: Development of a Minimal Documentation System. *Palliat Med* 2000;14(4):266-276

39 Brunner-La Rocca HP, Rickenbacher P, Muzzarelli S, et al. End-of-life preferences of elderly patients with chronic heart failure. *Eur Heart J* 2012;33(6):752-759

40 Iyasere O, Brown EA, Johansson L, et al. Quality of life with conservative care compared with assisted peritoneal dialysis and haemodialysis. *Clin Kidney J* 2018;12(2):262-268

41 Voll-Aanerud M, Eagan TML, Wentzel-Larsen T, et al. Respiratory symptoms, COPD severity,

For peer review only

For peer review only - http://bmjopen.bmj.com/site/about/guidelines.xhtml

and health related quality of life in a general population sample. *Respir Med* 2008;102(3):399-406

42 National Institute for Health and Clinical Excellence (NICE). *Weight management: lifestyle services for overweight or obese adults.* 2014. https://www.nice.org.uk/guidance/ph53 (accessed January 2020)

43 Welbourn R, Hollyman M, Kinsman R, et al. Bariatric Surgery Worldwide: Baseline Demographic Description and One-Year Outcomes from the Fourth IFSO Global Registry Report 2018. *Obes Surg* 2019;29(3):782-795

44 Avenell A, Robertson C, Skea Z, et al. Bariatric surgery, lifestyle interventions and orlistat for severe obesity: The rebalance mixed-methods systematic review and economic evaluation. *Health Technol Assess* 2018;22(68):1-246

45 Alsumali A, Eguale T, Bairdain S, et al. Cost-Effectiveness Analysis of Bariatric Surgery for Morbid Obesity. *Obes Surg* 2018;28(8):2203-2214

46 Maciejewski ML, Arterburn DE. Cost-effectiveness of Bariatric Surgery. *JAMA* 2013;310(7):742-743

47 Keating C, Neovius M, Sjöholm K, et al. Health-care costs over 15 years after bariatric surgery for patients with different baseline glucose status: Results from the Swedish Obese Subjects study. *Lancet Diabetes Endocrinol* 2015;3(11):855-865

48 Hoerger TJ, Zhang P, Segel JE, et al. Cost-effectiveness of bariatric surgery for severely obese adults with diabetes. *Diabetes Care* 2010;33(9):1933-1939

49 Borisenko O, Mann O, Duprée A. Cost-utility analysis of bariatric surgery compared with conventional medical management in Germany: A decision analytic modeling. *BMC Surg* 2017;17(1):87

For peer review only - http://bmjopen.bmj.com/site/about/guidelines.xhtml

For peer review only

50 Khan MA, Grinberg R, Johnson S, et al. Perioperative risk factors for 30-day mortality after bariatric surgery: Is functional status important? *Surg Endosc* 2013;27(5):1772-1777

51 Quilliot D, Sirveaux MA, Nomine-Criqui C, et al. Evaluation of risk factors for complications after bariatric surgery. *J Visc Surg* 2018;155(3):201-210

52 Livhits M, Mercado C, Yermilov I, et al. Preoperative predictors of weight loss following bariatric surgery: Systematic review. *Obes Surg* 2012;22:70-89

53 Nickel F, De La Garza JR, Werthmann FS, et al. Predictors of Risk and Success of Obesity Surgery. *Obes Facts* 2019;12(4):427-439

54 Parri A, Benaiges D, Schröder H, et al. Preoperative predictors of weight loss at 4 years following bariatric surgery. *Nutr Clin Pract* 2015;30(3):420-424

55 Jans A, Näslund I, Ottosson J, et al. Duration of type 2 diabetes and remission rates after bariatric surgery in Sweden 2007-2015: A registry-based cohort study. *PLoS Med* 2019;16(11):e1002985

56 Malone M, Alger-Mayer S, Polimeni JM. Health Related Quality of Life After Gastric Bypass Surgery. *Appl Res Qual Life* 2012;7:155-161

57 Pristed SG, Omar HK, Kroustrup JP. Association Between Fulfilment of Expectations and Health-related Quality of Life after Gastric Bypass. *Appl Res Qual Life* 2013;8(1):101-111

58 Julia C, Ciangura C, Capuron L, et al. Quality of life after Roux-en-Y gastric bypass and changes in body mass index and obesity-related comorbidities. *Diabetes Metab* 2013;39(2):148-154

59 Spencer EA, Appleby PN, Davey GK, et al. Validity of self-reported height and weight in 4808 EPIC–Oxford participants. *Public Health Nutr* 2002;5(4):561-565

60 Public Health Scotland Data and Intelligence. https://www.isdscotland.org (accessed January 2020)

For peer review only

596

597 **FIGURE LEGEND**

598 **Figure 1. Screening, consent and follow-up.** 548 patients were screened for inclusion in the SurgiCal
599 Obesity Treatment Study. Of these 548, 103 were non-eligible (62 patients had undergone previous
600 bariatric surgery and 1 patient lacked the necessary English language skills) and 40 were eligible but
601 did not consent. 31 patients consented to only part 1 of the study (data linkage) while 413
602 consented to both part 1 and part 2 (completion of baseline PROMs). 249 baseline PROMs were
603 available for analysis. PROMs, patient reported outcome measures.

For peer review only

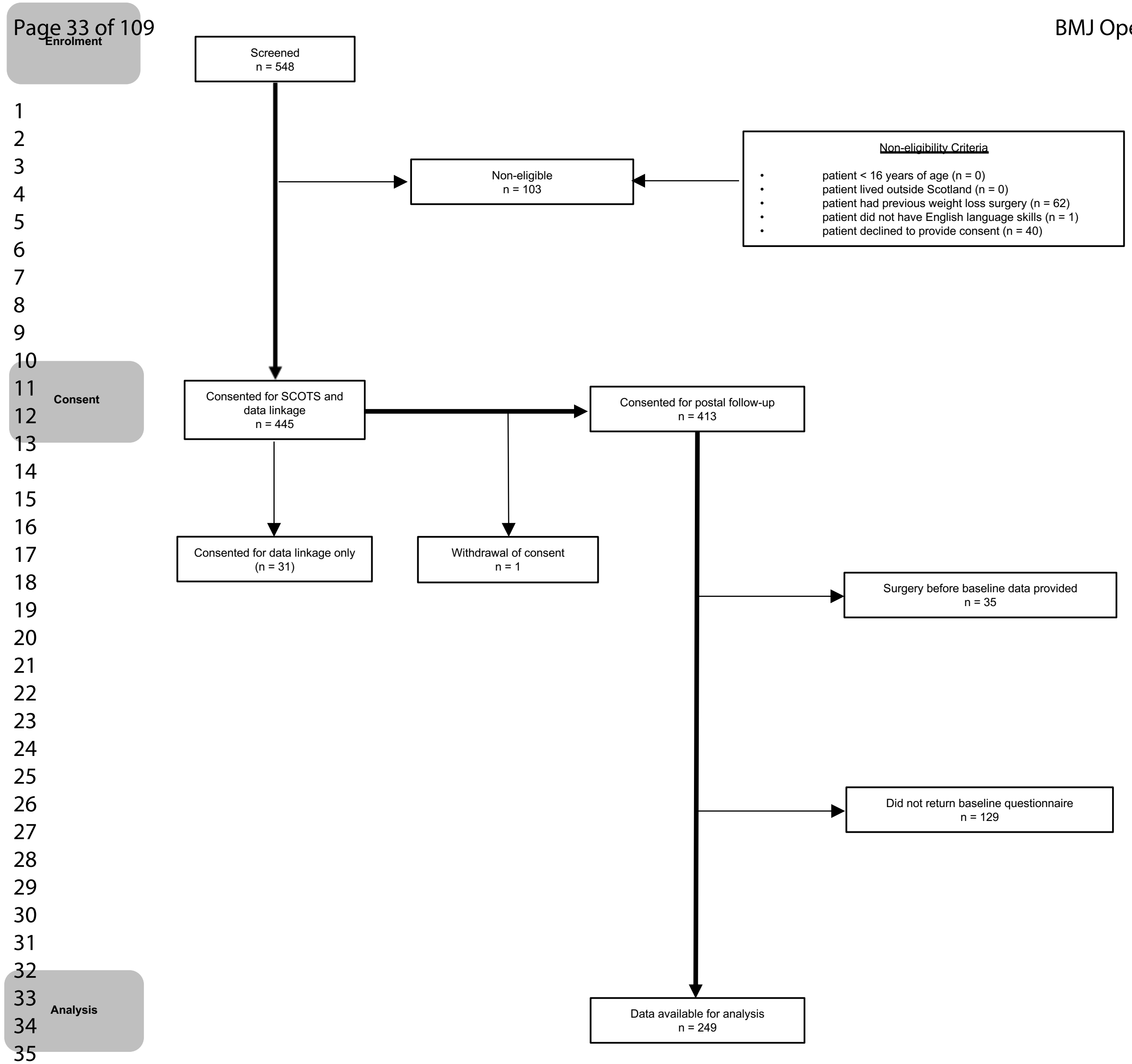
Enrolment
Screened
n = 548
Non-eligible
n = 103
Non-eligibility Criteria
• patient < 16 years of age (n = 0)
• patient lived outside Scotland (n = 0)
• patient had previous weight loss surgery (n = 62)
• patient did not have English language skills (n = 1)
• patient declined to provide consent (n = 40)
Consent
Consented for SCOTS and data linkage
n = 445
Consented for postal follow-up
n = 413
Consented for data linkage only
(n = 31)
Withdrawal of consent
n = 1
Surgery before baseline data provided
n = 35
Did not return baseline questionnaire
n = 129
Analysis
Data available for analysis
n = 249

Supplementary Table 1. STROBE Statement completed checklist (from von Elm *et al.* [1]).

SECTION/TOPIC	ITEM No.	STROBE Recommendation	OUR PAPER
**Title and abstract**	1	(a) Indicate the study's design with a commonly used term in the title or the abstract	(a) See title
		(b) Provide in the abstract an informative and balanced summary of what was done and what was found	(b) See abstract
**Introduction**			
Background / rationale	2	Explain the scientific background and rationale for the investigation being reported	Paragraphs 1-3 provide background and paragraph 4 explains rationale
Objectives	3	State specific objectives, including any prespecified hypotheses	Paragraph 4
**Methods**			
Study design	4	Present key elements of study design early in the paper	Paragraph 3/'Study Design' section
Setting	5	Describe the setting, locations, and relevant dates, including periods of recruitment, exposure, follow-up, and data collection	Paragraphs 4-6/'Participant and Centre Eligibility', 'Recruitment Procedures and Consent' and 'Data Collection' sections
Participants	6	(a) Give the eligibility criteria, and the sources and methods of selection of participants. Describe methods of follow-up	(a) Paragraphs 4-6/'Participant and Centre Eligibility', 'Recruitment Procedures and Consent' and 'Data Collection' sections
		(b) For matched studies, give matching criteria and number of exposed and unexposed	(b) Not applicable
Variables	7	Clearly define all outcomes, exposures, predictors, potential confounders, and effect modifiers. Give diagnostic criteria, if applicable	Paragraphs 7-9/'Data Collection' section
Data sources / measurement	8	For each variable of interest, give sources of data and details of methods of assessment (measurement). Describe comparability of assessment methods if there is more than one group	Paragraphs 7 and 8/'Data Collection' section
Bias	9	Describe any efforts to address potential sources of bias	Not applicable
Study size	10	Explain how the study size was arrived at	Paragraph 4/'Patient and Centre Eligibility' section, paragraph 1 of 'Results' section

For peer review only

Quantitative variables	11	Explain how quantitative variables were handled in the analyses. If applicable, describe which groupings were chosen and why	'Statistical Analyses' section
Statistical methods	12	(a) Describe all statistical methods, including those used to control for confounding	(a) 'Statistical Analyses' section
		(b) Describe any methods used to examine subgroups and interactions	(b) Not applicable
		(c) Explain how missing data were addressed	(c) 'Statistical Analyses' section
		(d) If applicable, explain how loss to follow-up was addressed	(d) Not applicable
		(e) Describe any sensitivity analyses	(e) Not applicable
**Results**			
Participants	13	(a) Report numbers of individuals at each stage of study— e.g. numbers potentially eligible, examined for eligibility, confirmed eligible, included in the study, completing follow-up, and analysed	(a) Paragraph 1 and Figure 1
		(b) Give reasons for non-participation at each stage	(b) Paragraph 1 and Figure 1
		(c) Consider use of flow diagram	(c) Figure 1
Descriptive data	14	(a) Give characteristics of study participants (e.g. demographic, clinical, social) and information on exposures and potential confounders	(a) Paragraphs 2-5/'Characteristics of recruited and analysed sample', 'Comorbidities', "Health and obesity-related quality of life' and physical activity' sections, Table 1 and Table 2
		(b) Indicate number of participants with missing data for each variable of interest	(b) Paragraphs 2-7, Table 1, Table 2, Supplementary Table 2 and Supplementary Table 3
		(c) Summarise follow-up time (e.g. average and total amount)	(c) Not applicable
Outcome data	15	Report numbers of outcome events or summary measures over time	Table 2, Supplementary Table 2 and Supplementary Table 3
Main results	16	(a) Give unadjusted estimates and, if applicable, confounder-adjusted estimates and their precision (e.g. 95% confidence interval). Make clear which confounders were adjusted for and why they were included	(a) Table 3 and Supplementary Table 4
		(b) Report category boundaries when continuous variables were categorised	(b) Supplementary Table 4

For peer review only

		(c) If relevant, consider translating estimates of relative risk into absolute risk for a meaningful time period	(c) Not applicable
Other analyses	17	Report other analyses done e.g. analyses of subgroups and interactions, and	Not applicable
**Discussion**			
Key results	18	Summarise key results with reference to study objectives	Paragraph 1
Limitations	19	Discuss limitations of the study, taking into account sources of potential bias or imprecision. Discuss both direction and magnitude of any potential bias	Paragraph 5
Interpretation	20	Give a cautious overall interpretation of results considering objectives, limitations, multiplicity of analyses, results from similar studies, and other relevant evidence	'Conclusion'
Generalisability	21	Discuss the generalisability (external validity) of the study results	Paragraph 5
**Other**			
Funding	22	Give the source of funding and the role of the funders for the present study and, if applicable, for the original study on which the present article is based	'Funding' section

Reference

1) von EE, Altman DG, Egger M, Pocock SJ, Gotzsche PC, Vandenbroucke JP. The Strengthening the Reporting of Observational Studies in Epidemiology (STROBE) statement: guidelines for reporting observational studies. *Lancet* 2007; **370**(9596): 1453-1457

For peer review only

Supplementary Table 2. Surgical Obesity Treatment Study (SCOTS) baseline data by body mass index (BMI) group.

			BMI (kg/m^2)				
			Group 1	Group 2	Group 3	Group 4	Group 5
		Total Number of Participants Completing Question (N missing)	< 40 N=24	40-44 N=64	45-49 N=64	50-54 N=44	55+ N=53
**Demographics**	Sex: % Male	249 (0)	33.3%	23.4%	31.3%	38.6%	22.6%
	SIMD: % Q1 (Most Deprived)	247 (2)	20.8%	20.3%	25.0%	30.2%	44.2%
**Medical history**	% DVT	249 (0)	8.3%	1.6%	3.1%	4.5%	1.9%
	% Hypertension	249 (0)	45.8%	32.8%	51.6%	45.5%	41.5%
	% Type 2 Diabetes	249 (0)	45.8%	39.1%	60.9%	61.4%	41.5%
	% CVD	249 (0)	16.7%	9.4%	6.3%	6.8%	5.7%
	% Arthritis	249 (0)	16.7%	23.4%	35.9%	22.7%	39.6%
	% Back Problems	249 (0)	41.7%	45.3%	50.0%	38.6%	50.9%
	% Asthma	249 (0)	0.0%	25.0%	29.7%	27.3%	43.4%
	% Migraine	249 (0)	20.8%	10.9%	17.2%	25.0%	28.3%
	% Irritable Bowel Syndrome	249 (0)	16.7%	18.8%	20.3%	18.2%	13.2%
	% Sleep Apnoea	249 (0)	16.7%	20.3%	28.1%	27.3%	35.8%
	% PCOS	167 (10)	46.7%	8.3%	17.1%	20.0%	13.2%
	% Gastroesophageal Reflux	240 (9)	26.1%	38.1%	52.5%	39.5%	36.0%
**Incontinence**	% ICIQ-UI SF Score ≥6	239 (10)	26.1%	42.2%	46.7%	40.5%	54.0%
	% ICIQ-UI SF Score ≥6 (females only)	168 (9)	40.0%	42.9%	50.0%	56.0%	56.4%
	% ICIQ-UI SF Score ≥6 (males only)	71 (1)	0.0%	40.0%	40.0%	17.6%	45.5%
	Median (LQ, UQ) ICIQ-UI SF Score	239 (10)	0.0 (0.0; 6.0)	4.0 (0.0; 9.5)	5.0 (0.0; 8.0)	3.0 (0.0; 12.0)	9.0 (3.0; 13.0)
**Depression**	Mean (SD) PHQ-9 Score	244 (5)	7.4 (6.6)	9.5 (6.4)	9.0 (6.1)	10.9 (6.5)	10.5 (5.9)
	% PHQ-9 Score ≥10	244 (5)	26.1%	46.9%	38.1%	48.8%	51.0%
**Anxiety**	Median (LQ, UQ) GAD-7 Score	243 (6)	4.0 (1.0; 6.0)	5.0 (2.0; 10.5)	5.5 (2.0; 9.0)	4.0 (2.0; 12.0)	7.0 (3.0; 10.0)
	% GAD-7 Score ≥6	243 (6)	30.4%	42.2%	50.0%	39.5%	62.7%
**Smoking Status**	% Current	240 (9)	17.4%	4.8%	4.8%	2.3%	4.0%
	% Former		30.4%	45.2%	46.8%	39.5%	48.0%
	% Never		52.2%	50.0%	48.4%	58.1%	48.0%
**Quality of Life**							
SF-12	Mean (SD) SF-12 PCS	236 (13)	43.5 (11.3)	40.3 (11.0)	37.6 (10.6)	35.3 (11.6)	31.1 (10.3)
	Mean (SD) SF-12 MCS	236 (13)	48.7 (7.7)	44.8 (9.1)	45.7 (11.9)	43.8 (10.8)	46.1 (10.2)
EQ-5D-5L	Median (LQ, UQ) EQ-5D-5L Score	237 (12)	0.8 (0.7; 0.9)	0.7 (0.3; 0.8)	0.6 (0.4; 0.8)	0.6 (0.4; 0.7)	0.5 (0.1; 0.6)
	Mean (SD) EQ-5D-5L VAS	237 (12)	64.9 (22.6)	58.8 (22.3)	56.5 (22.5)	51.7 (18.9)	47.9 (21.8)
IWQOL-Lite (Standardised Scoring)	Mean (SD) Physical Function	243 (6)	35 (24.9)	50.5 (23.3)	54.2 (24.5)	61.1 (21.6)	74.8 (20.6)
	Median (LQ, UQ) Self Esteem	242 (7)	60.7 (42.9; 85.7)	71.4 (53.6; 89.3)	71.4 (39.3; 92.9)	89.3 (71.4; 96.4)	85.7 (71.4; 96.4)
	Mean (SD) Sexual Life	231 (18)	40.8 (33.4)	58.8 (30.0)	54.2 (31.2)	60.4 (34.7)	63.7 (28.8)
	Mean (SD) Public Distress	243 (6)	28.9 (25.7)	48.1 (22.4)	55.9 (25.7)	70.1 (24.1)	76.1 (19.8)
	Mean (SD) Work	236 (13)	31 (30.1)	38.9 (29.3)	40 (29.2)	47.5 (25.1)	55.5 (28.6)
	Mean (SD) Total Score	242 (7)	40.5 (22.5)	53.7 (18.9)	55.3 (22.0)	64.1 (19.0)	71.8 (17.5)

For peer review only

**Healthcare Utilisations**	% Use of Any Aids or Specialist Equipment	232 (17)	9.1%	21.3%	27.9%	25.6%	51.0%
**Social Security**	% Unable to Work Due to Illness or Disability	248 (1)	25.0%	23.4%	23.4%	23.3%	34.0%
	% Disability Living Allowance - Caring	236 (13)	17.4%	17.7%	21.3%	17.5%	18.0%
	% Disability Living Allowance - Mobility	236 (13)	17.4%	19.4%	23.0%	17.5%	20.0%

ICIQ-UI SF score: ≥6 = 'moderate incontinence' [1]
PHQ-9 scores: 0-4 = 'minimal depression'; 5-9 = 'mild depression'; 10-14 = 'moderate depression'; 15-19 = 'moderately severe depression'; 20-27 = 'severe depression' [2]
GAD-7 scores: 0-5 = 'mild anxiety'; 6-10 = 'moderate anxiety'; 11-15 = 'moderately severe anxiety'; 15-21 = 'severe anxiety' [3]

References

1. Avery K, Donovan J, Peters TJ, Shaw C, Gotoh M, Abrams P. ICIQ: A brief and robust measure for evaluating the symptoms and impact of urinary incontinence. *Neurourol Urodyn* 2004; **23**(4)**:** 322–330

2. Spitzer RL, Kroenke K, Williams JBW. Validation and utility of a self-report version of PRIME-MD: The PHQ Primary Care Study. *J Am Med Assoc* 1999; **282**(18): 1737-1744

3. Spitzer RL, Kroenke K, Williams JBW, Löwe B. A brief measure for assessing generalized anxiety disorder: The GAD-7. *Arch Intern Med* 2006; **166**(10): 1092-1097

For peer review only

Supplementary Table 3. Surgical Obesity Treatment Study (SCOTS) baseline data by age group.

			Age (years)				
			Group 1	Group 2	Group 3	Group 4	Group 5
		Total Number of Participants Completing Question (N missing)	< 35 N=36	35-44 N=63	45-49 N=63	50-54 N=43	55+ N=44
**Demographics**	Sex: % Male	249 (0)	22.2%	22.2%	28.6%	30.2%	43.2%
	SIMD: % Q1 (Most Deprived)	247 (2)	40.0%	28.6%	14.5%	39.5%	27.3%
**Medical history**	% DVT	249 (0)	2.8%	3.2%	3.2%	0.0%	6.8%
	% Hypertension	249 (0)	11.1%	36.5%	52.4%	44.2%	63.6%
	% Type 2 Diabetes	249 (0)	30.6%	50.8%	54.0%	44.2%	63.6%
	% CVD	249 (0)	0.0%	6.3%	6.3%	9.3%	18.2%
	% Arthritis	249 (0)	5.6%	15.9%	36.5%	27.9%	59.1%
	% Back Problems	249 (0)	47.2%	50.8%	31.7%	39.5%	65.9%
	% Asthma	249 (0)	30.6%	22.2%	31.7%	20.9%	36.4%
	% Migraine	249 (0)	27.8%	17.5%	20.6%	18.6%	15.9%
	% Irritable Bowel Syndrome	249 (0)	8.3%	27.0%	23.8%	4.7%	15.9%
	% Sleep Apnoea	249 (0)	5.6%	20.6%	28.6%	25.6%	50.0%
	% PCOS	167 (10)	26.9%	26.1%	9.3%	7.1%	12.5%
	% Gastroesophageal Reflux	240 (9)	50.0%	50.0%	36.1%	28.6%	37.2%
**Incontinence**	% ICIQ-UI Score ≥6	239 (10)	18.2%	44.3%	50.8%	50.0%	47.6%
	% ICIQ-UI Score ≥6 (females only)	168 (9)	20.0%	51.1%	55.8%	62.1%	50.0%
	% ICIQ-UI Score ≥6 (males only)	71 (1)	12.5%	21.4%	38.9%	23.1%	44.4%
	Median (LQ, UQ) ICIQ-UI Score	239 (10)	0.0 (0.0; 4.0)	5.0 (0.0; 11.0)	6.0 (0.0; 11.0)	5.5 (3.0; 11.0)	4.5 (0.0; 11.0)
**Depression**	Mean (SD) PHQ-9 Score	244 (5)	9.0 (6.2)	10.3 (6.3)	9.2 (5.9)	9.1 (7.0)	10.2 (6.2)
	% PHQ-9 Score ≥10	244 (5)	42.9%	50.0%	37.7%	37.2%	51.2%
**Anxiety**	Median (LQ, UQ) GAD-7 Score	243 (6)	5.0 (2.0; 10.0)	6.0 (3.0; 12.0)	5.0 (3.0; 8.0)	5.0 (1.0; 11.0)	3.0 (1.0; 8.0)
	% GAD-7 Score ≥6	243 (6)	48.6%	54.8%	42.6%	47.6%	39.5%
**Smoking Status**	% Current	240 (9)	0.0%	6.5%	5.0%	0.0%	14.0%
	% Former		34.4%	45.2%	41.7%	48.8%	46.5%
	% Never		65.6%	48.4%	53.3%	51.2%	39.5%
**Quality of Life**							
SF-12	Mean (SD) SF-12 PCS	236 (13)	41.2 (11.4)	38 (10.9)	36.3 (11.8)	37.7 (11.9)	32.5 (10.1)
	Mean (SD) SF-12 MCS	236 (13)	44.9 (10.9)	44.5 (10.1)	46.2 (9.2)	44.7 (10.5)	46.8 (11.5)
EQ-5D-5L	Median (LQ, UQ) EQ-5D-5L Score	237 (12)	0.7 (0.6; 0.8)	0.6 (0.5; 0.8)	0.6 (0.5; 0.8)	0.7 (0.2; 0.8)	0.4 (0.2; 0.7)
	Mean (SD) EQ-5D-5L VAS	237 (12)	58.8 (20.7)	55.4 (21.7)	57.1 (21.7)	55.4 (25.8)	50.0 (20.4)
IWQOL-Lite (Standardised Scoring)	Mean (SD) Physical Function	243 (6)	49.5 (20.9)	54.0 (26.1)	57.2 (26.4)	57.7 (29.3)	66.0 (20.0)
	Median (LQ, UQ) Self Esteem	242 (7)	85.7 (67.9; 92.9)	85.7 (60.7; 96.4)	75.0 (53.6; 92.9)	71.4 (50.0; 92.9)	58.9 (39.3; 82.1)
	Mean (SD) Sexual Life	231 (18)	54.1 (26.5)	56.7 (32.4)	53.2 (33.7)	59.0 (31.9)	65.0 (31.2)
	Mean (SD) Public Distress	243 (6)	62.7 (25.8)	61.5 (26.0)	58 (26.8)	55.7 (31.7)	52.0 (25.6)
	Mean (SD) Work	236 (13)	44.3 (29.2)	42.7 (28.2)	38.8 (26.0)	47.3 (34.5)	47.1 (29.7)
	Mean (SD) Total Score	242 (7)	57.4 (18.8)	59.4 (21.6)	57.0 (22.7)	58.1 (26.0)	60.3 (18.2)

For peer review only

**Healthcare Utilisations**	% Use of Any Aids or Specialist Equipment	232 (17)	16.1%	14.5%	24.1%	42.5%	53.7%
**Social Security**	% Unable to Work Due to Illness or Disability	248 (1)	11.1%	17.5%	25.4%	28.6%	47.7%
	% Disability Living Allowance - Caring	236 (13)	9.4%	19.4%	17.2%	14.3%	31.0%
	% Disability Living Allowance - Mobility	236 (13)	12.5%	17.7%	17.2%	16.7%	35.7%

ICIQ-UI SF score: ≥6 = 'moderate incontinence' [1]
PHQ-9 scores: 0-4 = 'minimal depression'; 5-9 = 'mild depression'; 10-14 = 'moderate depression'; 15-19 = 'moderately severe depression'; 20-27 = 'severe depression' [2]
GAD-7 scores: 0-5 = 'mild anxiety'; 6-10 = 'moderate anxiety'; 11-15 = 'moderately severe anxiety'; 15-21 = 'severe anxiety' [3]

References

1. Avery K, Donovan J, Peters TJ, Shaw C, Gotoh M, Abrams P. ICIQ: A brief and robust measure for evaluating the symptoms and impact of urinary incontinence. *Neurourol Urodyn* 2004; **23**(4)**:** 322–330
2. Spitzer RL, Kroenke K, Williams JBW. Validation and utility of a self-report version of PRIME-MD: The PHQ Primary Care Study. *J Am Med Assoc* 1999; **282**(18): 1737-1744
3. Spitzer RL, Kroenke K, Williams JBW, Löwe B. A brief measure for assessing generalized anxiety disorder: The GAD-7. *Arch Intern Med* 2006; **166**(10): 1092-1097

For peer review only

Supplementary Table 4. Significant interactions with smoking as a two-level variable

Outcome	Model	Sub-population	Effect	OR (95% CI)	p-value
Use of aids or specialist equipment	Adjusted model as previously provided (smoke as 3-level)		Age	3.10 (1.94, 4.95)	<0.0001
	Adjusted model (smoke as 2-level)		Age	3.21 (2.01, 5.12)	<0.0001
	Adjusted model extended to include age*smoke and BMI*smoke		Age*smoke		0.044
			BMI*smoke		0.87
	Adjusted model, for the effect of Age in each of the smoking sub-populations	ANY Smoker	Age	2.35 (1.27, 4.36)	0.0065
		NEVER Smoker	Age	6.49 (2.63, 16.00)	<0.0001
PHQ-9 (assume included in the supplementary tables)	Adjusted model as previously provided (smoke as 3-level)		BMI	1.33 (0.96, 1.86)	0.0865
	Adjusted model (smoke as 2-level)		BMI	1.31 (0.94, 1.81)	0.1093
	Adjusted model extended to include age*smoke and BMI*smoke		Age*smoke		0.8800
			BMI*smoke		0.0156
	Adjusted model, for the effect of Age	ANY Smoker	BMI	0.88 (0.56, 1.37)	0.5679

For peer review only

	in each of the smoking sub-populations				
		NEVER Smoker	BMI	2.28 (1.31, 3.96)	0.0037

Unless otherwise noted, smoking status refers to a two-level variable [any smoking (current or former) and never smoked] as opposed to the three-level variable (current, former and never) used in previous analyses.

The sub-population model of never smokers for the use of aids and specialist equipment in the home outcome should be interpreted with caution due to small numbers of events.

For peer review only

Supplementary Table 5. Association of age and body mass index (BMI) with self-reported comorbidities and mental health indicators.

Comorbidities and Mental Health Indicators	Variable	BMI (Model 1[1]) and Age (Model 2[2])		BMI and Age (Model 3[3])	
		Odds Ratio (95% CI)	p-value	Odds Ratio (95% CI)	p-value
**Hypertension (N = 107)**	BMI	0.97 (0.72, 1.31)	0.84	0.89 (0.63, 1.24)	0.49
	Age	2.03 (1.47, 2.79)	<0.0001	2.02 (1.44, 2.85)	<0.0001
**Type 2 Diabetes (N = 124)**	BMI	0.94 (0.69, 1.26)	0.66	0.93 (0.67, 1.30)	0.68
	Age	1.41 (1.06, 1.87)	0.02	1.36 (1.00, 1.85)	0.05
**CVD (N = 20)**	BMI	0.61 (0.31, 1.18)	0.14	0.71 (0.36, 1.42)	0.34
	Age	2.98 (1.52, 5.85)	<0.01	2.27 (1.10, 4.67)	0.03
**Arthritis (N = 73)**	BMI	1.45 (1.05, 2.00)	0.02	1.62 (1.09, 2.40)	0.02
	Age	3.01 (2.00, 4.54)	< 0.0001	3.64 (2.26, 5.88)	< 0.0001
**Back Problems (N = 115)**	BMI	1.09 (0.81, 1.47)	0.57	1.12 (0.81, 1.54)	0.49
	Age	1.21 (0.91, 1.59)	0.19	1.12 (0.83, 1.51)	0.46
**Asthma (N = 70)**	BMI	1.85 (1.32, 2.59)	<0.001	1.81 (1.27, 2.58)	<0.01
	Age	1.12 (0.82, 1.52)	0.49	1.11 (0.79, 1.57)	0.54
**Migraine (N = 49)**	BMI	1.55 (1.09, 2.20)	0.02	1.63 (1.11, 2.38)	0.01
	Age	0.85 (0.60, 1.18)	0.33	0.91 (0.62, 1.33)	0.63
**Irritable Bowel Syndrome (N = 44)**	BMI	0.97 (0.66, 1.44)	0.90	1.00 (0.65, 1.54)	1.00
	Age	0.98 (0.69, 1.41)	0.93	1.07 (0.71, 1.60)	0.76
**Sleep Apnoea (N = 66)**	BMI	1.69 (1.21, 2.36)	<0.01	1.88 (1.27, 2.77)	<0.01
	Age	2.28 (1.55, 3.34)	< 0.0001	2.05 (1.35, 3.11)	<0.001
**PCOS (N = 28)**	BMI	0.72 (0.42, 1.23)	0.23	0.74 (0.42, 1.30)	0.29
	Age	0.56 (0.36, 0.88)	0.01	0.56 (0.35, 0.90)	0.02
**Gastroesophageal Reflux (N = 97)**	BMI	1.02 (0.75, 1.39)	0.88	1.06 (0.76, 1.47)	0.73
	Age	0.73 (0.55, 0.98)	0.04	0.68 (0.50, 0.93)	0.01
**Male Incontinence (ICIQ-UI score ≥ 6) (N = 22)**	BMI	1.58 (0.81, 3.09)	0.18	1.99 (0.92, 4.30)	0.08
	Age	2.05 (1.05, 4.01)	0.04	2.19 (1.06, 4.53)	0.03

For peer review only

**Female Incontinence (ICIQ-UI score ≥ 6) (N = 83)**	BMI	1.45 (1.01, 2.09)	0.05	1.40 (0.94, 2.08)	0.10
	Age	1.59 (1.10, 2.28)	0.01	1.49 (1.02, 2.19)	0.04
**Depression (PHQ-9 score ≥ 10) (N = 107)**	BMI	1.25 (0.92, 1.70)	0.15	1.33 (0.96, 1.86)	0.09
	Age	1.04 (0.79, 1.37)	0.79	0.99 (0.73, 1.34)	0.94
**Anxiety (GAD-7 score ≥ 6) (N = 114)**	BMI	1.42 (1.04, 1.95)	0.03	1.47 (1.05, 2.05)	0.03
	Age	0.91 (0.69, 1.20)	0.49	0.91 (0.67, 1.24)	0.56

[1] Model 1 – Unadjusted model including only the effect of BMI (per 10 kg/m^2) on comorbidity.

[2] Model 2 – Unadjusted model including only the effect of age (per 10 years) on comorbidity.

[3] Model 3 – Model including the effects of age (per 10 years) and BMI (per 10 kg/m^2) on comorbidity, after adjusting additionally for sex, Scottish Index of Multiple Deprivation (SIMD) and smoking status.

Regressions analyses were performed for conditions affecting 10 or more participants only.

For peer review only

Surgical Obesity Treatment Study

SCOTS

**Running title:**	**Surgical Obesity Treatment Study**
**Protocol Version:**	**1.6**
**Date:**	22/11/2017
**REC Reference Number:**	**13/WS/0005**
**ISRCTN/Clinical trial.gov:**	**47072588**
**Sponsor's Protocol Number:**	**GN21CA196**
**Sponsor:**	**NHS Greater Glasgow & Clyde**
**Funder:**	**National Institute for Health Research Health Technology Assessment Programme Ref 10/42/02**

Date	Protocol version	Amendment number & description
14/12/2012	1.0	Original
15/04/2013	1.1	Amendment 1: Minor changes to the format and presentation of the protocol
11/07/2013	1.2	Amendment 2: Correction of typographical errors in the protocol
30/06/2014	1.3	Amendment 3: Change to recruitment process for private hospitals involved in the study. Private hospitals will now become participant identification sites (and any NHS sites which in future are unable to support local recruitment), with participants being recruited and

For peer review only - http://bmjopen.bmj.com/site/about/guidelines.xhtml

		consented by a research team member (usually a research nurse).
03/06/2015	1.4	Amendment 4: Introduction of follow-up letter for patients recruited over the telephone by research nurses at the clinical research facility if completed consent form is not received within two weeks.
07/02/2017	1.5	Amendment 5; Revised study plan (including timetable & outputs). Patient recruitment stopping at a lower number and earlier than originally planned due to unforeseen recruitment issues. Amendment to clinical objectives and outcomes to reflect smaller sample size and shorter follow-up period. Reduction of post-operative questionnaire frequency to 2 and 3 years post-operatively only. Introduction of modest voucher incentives for PROMS completion.
22/11/2017	1.6	Amendment 6; Removal of annual blood test reminder. This procedure is completed as part of the routine clinical care by the patient's GP and therefore, it is not necessary for a reminder to be sent in addition for the SCOTS study.

This study will be performed according to the Research Governance Framework for Health and Community Care (Second edition, 2006) and WORLD MEDICAL ASSOCIATION DECLARATION OF HELSINKI Ethical Principles for Medical Research Involving Human Subjects 1964 (as amended).

For peer review only - http://bmjopen.bmj.com/site/about/guidelines.xhtml

Glasgow Clinical Trials Unit

CONTACTS

Chief Investigator

Dr Jennifer Logue MBChB MRCP MD FRCPath
CSO Clinician Scientist
Clinical Senior Lecturer and Honorary Consultant in Metabolic Medicine
Institute of Cardiovascular and Medical Sciences University of Glasgow
BHF Glasgow Cardiovascular Research Centre University of Glasgow
126 University Place, Room 208
University of Glasgow
Glasgow
G12 8TA

Tel: 0141 330 2569
Fax: 0141 330 6955
E-mail: Jennifer.Logue@Glasgow.ac.uk

Co-investigator

Professor Naveed Sattar
Professor of Metabolic Medicine
Cardiovascular and Medical Sciences
BHF Glasgow Cardiovascular Research Centre
RC214 Level C2
126 University Place, Room 208
University of Glasgow
Glasgow
G12 8TA
Tel: 0141 330 3419
Fax: 0141 330 6972
E-mail: Naveed.Sattar@glasgow.ac.uk

Dr Julie Bruce
Principal Research Fellow
Division of Health Sciences,
Warwick Medical School,
The University of Warwick,
Coventry,
CV4 7AL
Tel: 024 7615 1128
E-mail: julie.bruce@warwick.ac.uk

Mr Duff Bruce
Bariatric Surgeon
Ward 33
Aberdeen Royal Infirmary
Foresterhill
Aberdeen
AB25 2ZN
Tel: 01224 551275
Fax: 01224 443212
E-mail: duff.bruce@nhs.net

For peer review only

For peer review only - http://bmjopen.bmj.com/site/about/guidelines.xhtml

Glasgow Clinical Trials Unit

Dr Robert Lindsay
Reader Diabetes and Endocrinology
Cardiovascular and Medical Sciences
RC337 Level C3
BHF Glasgow Cardiovascular Research Centre
126 University Place, Room 208
University of Glasgow
Glasgow
G12 8TA
Tel: 0141 211 2502
Fax: 0141 330 6997
E-mail: Robert.Lindsay@glasgow.ac.uk

Professor Ian Ford
Professor of Biostatistics
R1122B Level 11
Robertson Centre for Biostatistics
Boyd Orr Building
Glasgow
G12 8QQ
Tel: 0141 330 4048
Fax: 0141 330 5094
E-mail: Ian.Ford@glasgow.ac.uk

Professor Andrew Briggs
Chair in Health Economics
(Health Economics and Health Technology Assessment) HEHTA
Institute of Health & Wellbeing
1 Lilybank Gardens
Glasgow
G12 8RZ
Tel: 0141 330 5017
E-mail: Andrew.Briggs@glasgow.ac.uk

Professor Mike Lean
Professor in Human Nutrition
R408 Level 4
Human Nutrition
Glasgow Royal Infirmary
84 Castle Street Glasgow
G40SF
Tel: 0141 211 4686
Fax: 0141 211 4844
E-mail: Mike.Lean@glasgow.ac.uk

Jane Munro
Research Lead
Cardiovascular and Medical Sciences
BHF Glasgow Cardiovascular Research Centre University of Glasgow
126 University Place, Room 208
University of Glasgow
Glasgow

For peer review only - http://bmjopen.bmj.com/site/about/guidelines.xhtml

Glasgow Clinical Trials Unit

G12 8TA
Tel: 0141 330 1670
E-mail: Jane.Munro@glasgow.ac.uk

Project Research Assistant

Mrs. Joanne O'Donnell
SCOTS Research Assistant
Cardiovascular and Medical Sciences
BHF Glasgow Cardiovascular Research Centre University of Glasgow
126 University Place, Room 208
University of Glasgow
Glasgow
G12 8TA
Tel: 0141 330 1670
Fax: 0141 330 5094
E-mail: Joanne.O'Donnell@glasgow.ac.uk

Study Statistician

Professor Ian Ford
Professor of Biostatistics
R1122B Level 11
Robertson Centre for Biostatistics
Boyd Orr Building
Glasgow
G12 8QQ
Tel: 0141 330 4048
Fax: 0141 330 5094
E-mail: Ian.Ford@glasgow.ac.uk

Data Centre

Alex McConnachie
Assistant Director of Biostatistics (Robertson Centre)
R1122E Level 11
Robertson Centre for Biostatistics
Boyd Orr Building
Glasgow
G12 8QQ
Tel: 01413304744
Fax: 0141 330 5094
E-mail: Alex.McConnachie@glasgow.ac.uk

Chair of the Study Steering Committee

Mr Richard Welbourn
Bariatric Surgeon
Musgrove Park Hospital
Taunton
TA1 5DA
Tel: 01823 343 565
Fax: 01823 353560
E-mail: richardwelbourn@gmail.com

For peer review only - http://bmjopen.bmj.com/site/about/guidelines.xhtml

Sponsor - NHS Greater Glasgow and Clyde R&D

Sponsor's representative

Dr Maureen Travers
Research Co-ordinator
NHS Greater Glasgow & Clyde
Research and Development Management Office
Tennent Institute
38 Church Street
Western Infirmary
Glasgow G11 6NT
Tel: 0141 211 6389
E-mail: maureen.travers@ggc.scot.nhs.uk

Funding Body

National Institute for Health Research (NIHR)

Funding Body Representative

Mrs Lesley Dodd
Programme Manager
NIHR Co-ordinating Centre for Health Technology Assessment (NETSCC, HTA)
University of Southampton
Alpha House
Enterprise Road
Southampton Science Park
Chilworth
Southampton
SO16 7NS
Tel: 02380 597558
Fax: 02380 595639
E-mail: L.Dodd@southampton.ac.uk

For peer review only

For peer review only - http://bmjopen.bmj.com/site/about/guidelines.xhtml

Glasgow Clinical Trials Unit

PROTOCOL APPROVAL

Surgical Obesity Treatment Study (SCOTS)

Chief Investigator — **Dr Jennifer Logue**
MBChB MRCP MD FRCPath CSO Clinician Scientist
Clinical Senior Lecturer and Honorary Consultant in Metabolic Medicine
Institute of Cardiovascular and Medical Sciences BHF Glasgow Cardiovascular Research Centre
University of Glasgow
126 University Place, Room 208
University of Glasgow
Glasgow
G12 8QQ

Signature:

Date: Tuesday, 03 August 2021

Sponsor's representative — **Dr Maureen Travers**
Research Co-ordinator
NHS Greater Glasgow & Clyde Research and Development Management Office
Tennent Institute
38 Church Street
Western Infirmary
Glasgow
G11 6NT

Signature:

Date:

For peer review only

For peer review only - http://bmjopen.bmj.com/site/about/guidelines.xhtml

TABLE OF CONTENTS

CONTACTS **3**

PROTOCOL APPROVAL **7**

TABLE OF CONTENTS **8**

ABBREVIATIONS **11**

STUDY SYNOPSIS **14**

GLOSSARY OF TERMS **16**

STUDY FLOW CHART **19**

SCHEDULE OF ASSESSMENTS **20**

1 INTRODUCTION **23**

1.1 Background 23

1.2 Rationale 23

1.2.1 Definition of bariatric surgery 24

1.2.2 Clinical Outcomes of weight loss surgery 24

1.3 Study hypothesis 25

2 STUDY OBJECTIVES **25**

2.1 Primary Objective 25

2.2 Secondary Objectives 25

2.3 Primary Outcome 26

2.4 Secondary Outcomes 26

3 STUDY DESIGN **27**

3.1 Overview of study design 27

3.1.1 Part 1 Record Linkage 29

3.1.2 Part 2 Patient Reported Outcomes (PROMS) Questionnaire 29

3.1.3 Part 3: Registration for a database of bariatric patients for future research 29

3.1.4 Service user involvement 30

3.2 Study Population 30

For peer review only - http://bmjopen.bmj.com/site/about/guidelines.xhtml

3.3 Reference populations 30

3.4 Inclusion criteria 31

3.5 Exclusion criteria 31

3.6 Identification of participants and consent 31

3.7 Withdrawal of subjects 32

4 STUDY PROCEDURES 33

4.1 Duration of participation 36

4.2 Study schedule 37

4.2.1 Start of Study - Surgical team registration 37

4.2.2 Visit 1 Routine care invitation visit 38

4.2.3 Visit 2 Recruitment visit 38

4.2.4 SCOTS study team contact patient 42

4.2.5 Visit 3 Weight loss operation 42

4.2.6 Visit 4 -Post operative follow up 42

4.2.7 Visit 5 - Re-operation (If applicable) 43

5 STATISTICS AND DATA ANALYSIS 44

5.1 Statistical Analysis Plan 44

5.2 Software for statistical analysis 44

5.3 Sample size 44

5.4 Management and delivery 46

6 STUDY CLOSURE 47

7 Data Handling 47

7.1.1 Surgeon registration data 47

7.1.2 Patient recruitment data 47

7.1.3 Surgical data collection 48

7.1.4 Patient Reported Outcomes 48

7.1.5 Routine clinical information 48

For peer review only - http://bmjopen.bmj.com/site/about/guidelines.xhtml

7.1.6 Re-operation 48

7.1.7 Record Retention 49

7.1.8 Access to data 49

8 SCOTS STUDY MANAGEMENT.. 50

8.1 Study Sponsor.. 50

8.2 SCOTS Study Team 50

8.3 SCOTS Principal investigators.. 50

8.4 Routine management of study: SCOTS Management Group (SMG) 50

8.5 SCOTS Steering Committee (SSC).. 51

9 STUDY MONITORING/AUDITING 52

10 PROTOCOL AMENDMENTS.. 52

11 ETHICAL CONSIDERATIONS.. 52

11.1 Ethical conduct of the study.. 52

11.2 Informed consent 52

12 INSURANCE AND INDEMNITY 53

13 FUNDING 53

14 ANNUAL REPORTS.. 53

15 DISSEMINATION OF FINDINGS.. 54

16 REFERENCES.. 55

17 Appendix E: Summary of Patient Reported Outcome Questions.. 59

For peer review only - http://bmjopen.bmj.com/site/about/guidelines.xhtml

ABBREVIATIONS

BHF GCRC	*British Heart Foundation, Glasgow Cardiovascular Research Centre*
BMI	*Body Mass Index*
BP	*Blood Pressure*
BPD-DS	*BilioPancreatic Diversion with Duodenal Switch*
CHD	*Coronary Heart Disease*
CHI	*Community Health Index*
CHF	*Congestive Heart Failure*
CI	*Chief Investigator*
CKD	*Chronic Kidney Disease*
CNORIS	*Clinical Negligence and Other Risks Indemnity Scheme*
CRF	*Case Report Form*
CSO	*Chief Scientist Office*
CTU	*Clinical Trials Unit*
CVD	*Cardiovascular Disease*
DM	*Diabetes Mellitus*
DM(E)C	*Data Monitoring (Ethics) Committee*
DOH	*Department of Health*
DS	*Duodenal Switch*
e-CRF	*Electronic Case Report Form*
EQ-5D	*EuroQol -5D*
GAD-7	*Generalised Anxiety Disorder – 7*
GCP	*Good Clinical Practice*
GCP	*Good Clinical Practice*
GCTU	*Glasgow Clinical Trials Unit*
GP	*General Practitioner*

For peer review only - http://bmjopen.bmj.com/site/about/guidelines.xhtml

GRDS	*Gastric Reduction Duodenal Switch*
GROS	*General Register Office for Scotland*
HbA1c	*Glycated Haemoglobin A1c*
HDL	*High Density Lipoprotein*
HR-QL	*Health Related- Quality of Life*
HTA	*Health Technology Assessment*
ICMS	*Institute of Cardiovascular and Medicinal Sciences*
IPAQ	*International Physical Activity Questionnaire*
IPSS	*International Prostate Symptom Score*
IRAS	*Integrated Research Application System*
ISD	*Information and Services Division (NHS)*
ISRCTN	*International Standard Randomised Controlled Trial Number*
IWQOL-Lite	*Impact of Weight on Quality of Life Lite Questionnaire*
LOT	*Life orientation Test*
MG	*Management Group*
MGM	*Management Group Meeting*
MI	*Myocardial Infarction*
NBSR	*National Bariatric Surgery Register*
NHS	*National Health Service*
NICE	*National Institute for Health and Clinical Excellence*
NIHR	*National Institute for Health Research*
NIHR CSP	*National Institute for Health Research Coordinated System for Gaining NHS Permission*
PHQ-9	*Patient Health Questionnaire - 9*
PI	*Principal Investigator*
PROMS	*Patient Reported Outcome Measures*
QALY	*Quality Adjusted Life Year*

For peer review only

For peer review only - http://bmjopen.bmj.com/site/about/guidelines.xhtml

QOL	*Quality Of Life*
R&D	*Research and Development*
RCB	*Robertson Centre for Biostatistics*
REC	*Research Ethics Committee*
SCOTS	*SurgiCal Obesity Treatment Study*
SCR	*Scottish Cancer Registry*
SCI-DC	*Scottish Care Information – Diabetes Collaboration*
SDRN	*Scottish Diabetes Research Network*
SDRN EG	*Scottish Diabetes Research Network Epidemiology Group*
SF-12	*Short Form -12*
SIGN	*Scottish Intercollegiate Guidelines Network*
SMG	*SCOTS Management Group*
SMR	*Scottish Morbidity Record*
SOP	*Standard Operating Procedure*
SSC	*SCOTS Steering Committee*
SSI	*Surgical Site Infection*

For peer review only

For peer review only - http://bmjopen.bmj.com/site/about/guidelines.xhtml

Glasgow Clinical Trials Unit

STUDY SYNOPSIS

Title of Study:	**Surgical Obesity Treatment Study**; SCOTS
Study Centre:	University of Glasgow
Duration of Study:	03/01/2012 – 30/06/2026
Primary Objective:	The SurgiCal Obesity Treatment Study (SCOTS) will establish the clinical outcomes and adverse events of different bariatric surgical procedures (taking into consideration the different care pathways), their impact on quality of life and nutritional status, and the effect on co-morbidities in a cohort of 450 patients.
Secondary Objectives:	1. The physical and mental health and social burden of severe obesity.<br>2. Incidence of acute and chronic postoperative complications. Acute complications, defined as up to three months post-surgery, will include surgical site infection; chronic complications will include revisional surgery, plastic surgery and chronic pain, for different bariatric surgical procedures<br>3. The effect of surgical experience and the pre and post-operative care pathway on complication rates and weight loss, for different bariatric surgical procedures.<br>4. The effect of the pre-surgical pathway and criteria on bariatric surgery patient selection.<br>5. Change in health related quality of life, anxiety and depression over time pre- and post-operatively for a mean of 3 years from date of bariatric surgery.<br>6. The weight status pre and post-operatively for 3 years after bariatric surgery.<br>7. The glycaemic control, lipids, blood pressure, medication prescription, and rate of diabetes complications (microalbuminuria and renal disease, and retinopathy) in those that have pre-existing diabetes or develop diabetes for 3 years follow up since bariatric surgery.<br>8. Changes in socio-economic factors (employment, benefit receipt, sick leave, and health care use) for 3 years since bariatric surgery.
Primary Endpoint:	Change in weight from pre-bariatric surgery weight

For peer review only - http://bmjopen.bmj.com/site/about/guidelines.xhtml

Glasgow Clinical Trials Unit

Rationale:	The efficacy of bariatric surgery for large scale, long term weight loss is well established. However, many questions remain over the medium term benefits of that weight loss for health, particularly when accounting for the potential complications of surgery.
Methodology:	Observational cohort study/ registry
Sample Size:	450
Screening:	All new bariatric surgery patients
Registration/Randomisation:	Not applicable
Main Inclusion Criteria:	• Patients 16 years or over undergoing their first bariatric surgery in NHS hospitals or private practice in Scotland.<br>• Capacity to consent<br>• Resident in Scotland<br>• Written informed consent
Main Exclusion Criteria:	• Does not meet inclusion criteria<br>• Patients who have had previous weight loss surgery (at time of potential recruitment are undergoing a repeat procedure)<br>• No written informed consent<br>• Patients under 16 years<br>• Patients who do not have capacity to consent for themselves<br>• Weight loss surgery performed at a site outside Scotland<br>• Patients who live outside Scotland<br>• Patients who do not have strong enough English language skills to understand the patient information leaflet without assistance will be excluded from Part 2 of the study.
Product, Dose, Modes of Administration:	Web based or paper questionnaire to be completed<br>1 month pre-surgery<br>2 years post surgery<br>3 years post surgery
Duration of Treatment:	Observational study so protocol will be applied until a participant has been in study for 3 years or until December 2020 whichever comes first. If funding available record linkage will continue throughout patient's life.
Statistical Analysis:	Detailed statistical analysis plans will be developed by the Robertson Centre for Biostatistics to address each of the objectives described above.

For peer review only - http://bmjopen.bmj.com/site/about/guidelines.xhtml

GLOSSARY OF TERMS

***Bariatric Surgical Procedures**: A bariatric surgical procedure is defined as a surgical intervention which has the primary purpose of large scale weight loss in a patient who is obese. Bariatric Surgical procedures eligible for inclusion in SCOTS are: **duodenal switch**, **gastric banding, gastric bypass** and **sleeve gastrectomy**. The inclusion of emerging techniques in this definition will be decided by the steering committee.*

***Biliopancreatic diversion with duodenal switch (BPD-DS):** see **Duodenal Switch**:*

***Body mass index (BMI)** is a simple index of weight-for-height that is commonly used to classify **overweight** and **obesity** in adults. It is defined as a person's weight in kilograms divided by the square of his height in meters (kg/m^2). BMI provides the most useful population-level measure of overweight and obesity as it is the same for both sexes and for all ages of adults. However, it should be considered a rough guide because it may not correspond to the same degree of fatness in different individuals.*

***Co-morbidity**: is either the presence of one or more disorders (or diseases) in addition to a primary disease or disorder, or the effect of such additional disorders or diseases.*

***Duodenal Switch**: (DS) is a weight loss surgery procedure that is composed of a restrictive and a malbsorbative aspect. The restrictive portion of the surgery involves removing approximately 70% of the stomach along the greater curvature. The malabsorptive portion of the surgery reroutes a lengthy portion of the small intestine, creating two separate pathways and one common channel. Duodenal switch (DS) is also known as biliopancreatic diversion with duodenal switch (BPD-DS) or gastric reduction duodenal switch (GRDS).*

***Endoluminal barrier:** is a liner that fits inside a section of the intestine, creating a barrier between the intestinal wall and the food you eat. It helps patients to lose weight by preventing food from coming into contact with the intestinal wall thus delaying digestion, which will alter the activation of hormonal signals that originate in the intestine.*

***Gastric balloon**: An inflatable device implanted in the stomach as an adjunct to therapy of morbid obesity. For the purposes of the SCOTS study a balloon treatment is not considered to be bariatric surgery. Use of a balloon before or after surgery will be recorded as a pre-operative treatment but a balloon alone would not meet the criteria for inclusion in SCOTS.*

***Gastric band:** adjustable gastric banding surgery, is a restrictive weight loss surgery that works by decreasing the amount of food that is able to be consumed before feeling full. During gastric banding surgery, a firm silicone band with an inner inflatable ring is placed around the stomach to slow the passage of food from the upper portion of the stomach to the lower portion*

***Gastric bypass**: a surgical bypass operation that typically involves reducing the size of the stomach and reconnecting the smaller stomach to bypass the first portion of the small*

For peer review only - http://bmjopen.bmj.com/site/about/guidelines.xhtml

intestine so as to restrict food intake and reduce caloric absorption in cases of severe obesity. This includes Gastric bypass, Roux en-Y (proximal), Gastric bypass, Roux en-Y (distal) and Mini-gastric bypass" (MGB)

Gastric reduction duodenal switch (GRDS): see duodenal switch

Glycaemic control*: is a medical term referring to the typical levels of blood sugar (glucose) in a person with diabetes mellitus. Much evidence suggests that many of the long-term complications of diabetes, especially the microvascular complications, result from many years of hyperglycemia (elevated levels of glucose in the blood).*

Hemoglobin A1c (HbA1c): *Blood sugar levels fluctuate throughout the day and glucose records are imperfect indicators of these changes, the percentage of haemoglobin which is glycosylated is used as a proxy measure of long-term glycemic control in research trials and clinical care of people with diabetes. This test, the hemoglobin A1c or glycosylated hemoglobin reflects average glucoses over the preceding 2–3 months. From 1 October 2011 the way in which HbA1c results are expressed in the UK has changed. Results are now reported in the IFCC reference method of mmol/L, rather than the DCCT units as a percentage. The equivalent of the current HbA1c targets of 6.5% and 7.5% are therefore 48mmol/mol and 58mmol/mol in the new units for diabetic patients, with the non-diabetic reference range of 4.0% to 6.0% being 20mmol/L to 42mmol/L.*

HDL cholesterol: *High density lipoprotein cholesterol. Lipoproteins, which are combinations of fats (lipids) and proteins, are the form in which lipids are transported in the blood. HDLs transport cholesterol from the tissues of the body to the liver, so the cholesterol can be eliminated in the bile. HDL cholesterol is therefore considered the 'good' cholesterol: The higher the HDL cholesterol level, the lower the risk of coronary artery disease. The average man has an HDL cholesterol level of 40 to 50 mg/dL. In the average woman, HDL levels range from 50 to 60 mg/dL.*

Health Technology Assessment*: is a multidisciplinary process that summarises information about the medical, social, economic and ethical issues related to the use of a health technology in a systematic, transparent, unbiased, robust manner. Its aim is to inform the formulation of safe, effective, health policies that are patient focused and seek to achieve best value.*

Hypertriglycerideamia*: denotes high (''hyper-'') blood levels (''-aemia'') of triglycerides, the most abundant fatty molecule in most organisms.*

Laparoscopic gastric banding*: Lap Band surgery, or adjustable gastric banding surgery, is a restrictive weight loss surgery that works by decreasing the amount of food that is able to be consumed before feeling full. During gastric banding surgery, a firm silicone band with an inner inflatable ring is placed around the stomach to slow the passage of food from the upper portion of the stomach to the lower portion. Most surgeries are performed laparoscopically, which allows the surgeon to work using long instruments placed in the body via incisions a few centimetres long.*

For peer review only - http://bmjopen.bmj.com/site/about/guidelines.xhtml

Laparoscopy*: is a type of surgical procedure that allows a surgeon to access the inside of the abdomen and pelvis without having to make large incisions in the skin. It is also known as keyhole surgery. Laparoscopy is minimally invasive. This is made possible with an instrument called a laparoscope.*

Morbidity*: Morbidity is an incidence of ill health.*

Mortality*: is incidence of death in a population.*

NHS patient*: A patient who receives free healthcare through the National Health Service*

Nutritional Status*: The state of a person's health in terms of the nutrients stored in his or her body.*

Obesity*: Obesity is a term used to describe somebody who is very overweight with a high degree of body fat. There are a number of ways a person's weight can be assessed. The most widely used method is the body mass index.*

Overweight*: a BMI of between 25 and 29, would be considered overweight*

Obese*: a BMI of 30 or above would be considered obese*

Private sector*: healthcare and medicine provided by entities other than the government and paid for by the patient or a private insurance company.*

Prospective cohort study*: a study designed to determine the relationship between a condition and a characteristic shared by some members of a group. The population selected is healthy at the beginning of the study. Members share a particular characteristic. The study follows the population groups over a long period, noting the rate at which a condition occurs in the population.*

Revisional surgery*: a bariatric surgical procedure on a patient who has already had bariatric surgery*

Roux-en-Y gastric bypass: *a small part of the stomach is used to create a new stomach pouch, roughly the size of an egg. The smaller stomach is connected directly to the middle portion of the small intestine (jejunum), bypassing the rest of the stomach and the upper portion of the small intestine (duodenum).*

Sleeve gastrectomy*: is a surgical weight-loss procedure in which the stomach is reduced to about 25% of its original size, by surgical removal of a large portion of the stomach, following the major curve. The open edges are then attached together (often with surgical staples) to form a sleeve or tube with a banana shape. The procedure permanently reduces the size of the stomach. The procedure is performed laparoscopically and is not reversible.*

For peer review only - http://bmjopen.bmj.com/site/about/guidelines.xhtml

Glasgow Clinical Trials Unit

STUDY FLOW CHART

FIGURE 0-1 THIS STUDY FLOW CHART SUMMARISES THE PATIENT JOURNEY TRHOUGHOUT THE STUDY

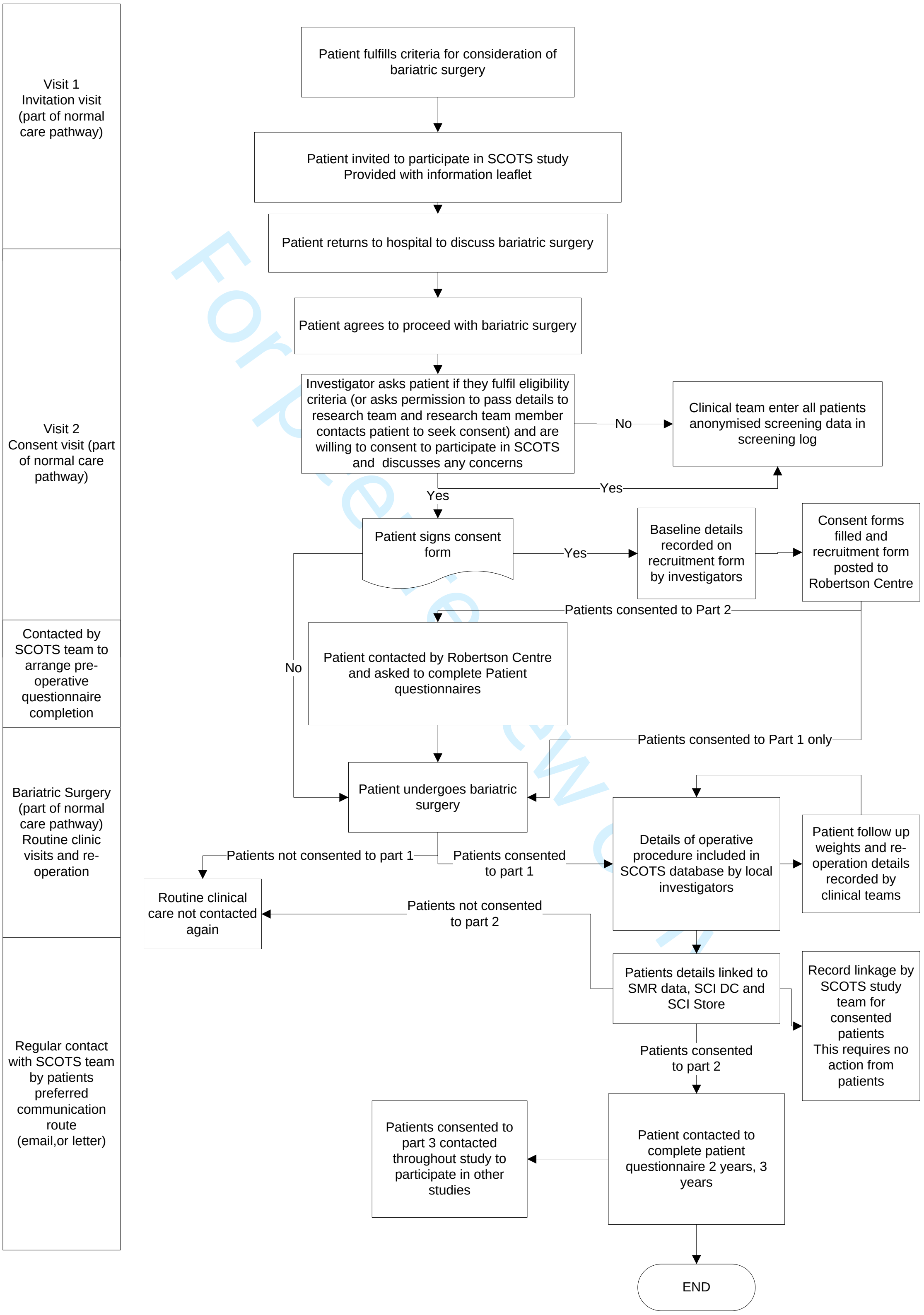

For peer review only - http://bmjopen.bmj.com/site/about/guidelines.xhtml

SCHEDULE OF ASSESSMENTS

TABLE 0-1: THIS SCHEDULE SHOWS EACH EVENT THAT INVOLVES THE PATIENT AND STUDY TEAM.

	Pre-surgery					Post surgery SCOTS Questionnaires	
Study Procedure	Pre-surgery Visit 1 1 year to six weeks before surgery	Pre-surgery Visit 2 1 year to six weeks before surgery	Pre-surgery Contact1	Pre-surgery Contact2	Surgery T0	2yr	3yr
Patients agree to proceed to bariatric surgery with clinical team	√						
Review Inclusion/Exclusion Criteria	√[a]						
Patients provided with patient information sheet	√						

For peer review only - http://bmjopen.bmj.com/site/about/guidelines.xhtml

Glasgow Clinical Trials Unit

Patient data entered onto screening log for SCOTS		√						
Patient return for pre-surgery clinic		√						
Clinical team ask permission from patients to pass details to research team who will contact patient (if not recruiting in local site)		(√)						
Obtain Informed Consent to participate in SCOTS study		√						
Patient baseline and contact details entered on registration form and sent to Robertson Centre for Biostatistics		√						
Consent form passed filed in Site File, copy given to patient and copy sent to RCB		√						
Contacted by SCOTS team to ask to complete pre-surgery questionnaire			√					

For peer review only

For peer review only - http://bmjopen.bmj.com/site/about/guidelines.xhtml

Patient completes pre-surgery questionnaire				√			
Patient undergoes bariatric surgery					√		
Clinical teams record operative details for patient post surgery					√		
Clinical teams record weight and re-operation details						√	√
Patients contacted to remind them to complete post surgery questionnaire						√	√
Part 3 consented patients may be invited to participate in other studies	Ongoing throughout study						
Record linkage to SCI-Store, SCI – DC, SMR (ISD), GRO throughout study	Ongoing throughout study, no interaction with patient unless to clarify if admission was a complication of bariatric surgery.						

For peer review only

1 INTRODUCTION

1.1 Background

SCOTS is a prospective cohort study commissioned by the Department of Health (DoH) National Institute of Health Research (NIHR) which will follow bariatric surgery patients for 3 years after their surgery. The NIHR commissioning brief for this study stated: "Obesity is a growing problem in the United Kingdom, with a growing number of people among the morbidly obese. Concomitantly, there is an increase in requests for bariatric surgery but insufficient evidence of long term effectiveness and safety of these procedures. There are existing registries of bariatric surgery, but these suffer from problems of collected data, completeness of follow- up, or data availability for secondary analysis. There is a need for a long term study of bariatric surgery, so that the outcomes and complications of different procedures, their impact on quality of life and nutritional status, and the effect on co-morbidities can be monitored in both the short and the long term." A recent Health Technology Assessment (HTA) (1) concluded that "Bariatric surgery is a more clinically effective intervention for treating obesity compared to non-surgical options" however they qualified this with "Uncertainties remain over the long-term outcomes of bariatric surgical interventions, particularly their impact on weight-related health problems and patient quality of life. Further studies will be required". This study was awarded NIHR HTA funding to address some of the uncertainties around the clinical effectiveness of bariatric surgery in the longer term. The original plan included a 10 year follow up period, however, due to unforeseen recruitment issues (reductions in both NHS & private surgical numbers) the study & statistical plans were reviewed. Following discussions with the NIHR it was agreed that many objectives could still be met from the number of patients currently recruited over a shorter period of follow up (3 years). The protocol has been updated to reflect these changes. Clearly some patients will have completed the 1 month, 6 month and 1 year post-operative questionnaire already. Where appropriate, these data will be used to supplement the year 2 and 3 questionnaires and the health record data in order to deliver the study objectives.

1.2 Rationale

The efficacy of bariatric surgery for large scale, long term weight loss is well established (2). However, many questions remain over the medium term benefits of that weight loss for health, particularly when accounting for the potential complications of surgery. Given that the proportions of the population who are obese is increasingly rapidly, further information is required as to who benefits most from bariatric surgery.

For peer review only - http://bmjopen.bmj.com/site/about/guidelines.xhtml

1.2.1 Definition of bariatric surgery

A bariatric surgical procedure is defined as a surgical intervention which has the primary purpose of large scale weight loss in a patient who is obese. Currently that includes gastric bypass, sleeve gastrectomy and laparoscopic gastric banding. The inclusion of emerging techniques in this definition will be decided by the SCOTS Steering Committee (SCC).

1.2.2 Clinical Outcomes of weight loss surgery

There are some pre-existing data on clinical outcomes with 10 year mortality rates being the best known. Overall mortality was found to be 29-40% lower in patients receiving bariatric surgery compared to Body Mass Index (BMI)-matched subjects not receiving surgery (3;4). Although impressive, these figures are from studies which initially recruited in the 1980s and early 1990s and further work is required to update the mortality rate in the current population, now that primary prevention measures for cardiovascular disease, such as statins and ACE-inhibitors, have lowered the risk of premature mortality in at risk subjects, including those who are obese or have diabetes.

Diabetes improvement post bariatric surgery has also been of interest. Dixon et al conducted a randomised controlled trial of laparoscopic gastric banding in patients with newly diagnosed type 2 diabetes resulted in a 73% remission rate after 2 years (5). Similar results have been shown for gastric bypass using meta-analysis of heterogeneous, mainly retrospective, cohort studies (6). Whether remission is maintained beyond the first few years post surgery is unknown. However, the main outcomes of interest are the longer term effects on the development of diabetes complications such as retinopathy, nephropathy and cardiovascular disease and those are not established.

The effect of bariatric surgery on cardiovascular outcomes has not been ascertained. It is known that HDL cholesterol is less likely to be low and hypertriglycerideamia is less common after surgery (7), as expected by a procedure which will reduce intra-abdominal adiposity and thus insulin resistance, but a reduction in cardiovascular events has not been shown. Hypertension, which would be presumed to decrease, in fact shows no difference 8 years post surgery, once the initial antihypertensive effects of acute weight loss in the first few years post-surgery are accounted for (8). Many have used cardiovascular risk factors measured during the acute weight loss phase, applied a population based cardiovascular risk score and stated a reduction in cardiovascular risk; this is of course a flawed use of such a score based on temporarily reduced cardiovascular risk factors. So, while the general population and the majority of physicians may assume that weight loss with bariatric surgery reduces the risk of a cardiovascular event, this is currently unknown.

What is established is that health related quality of life (HR-QoL) is increased compared to pre-operative levels (9). When patients who decide to proceed to surgery are compared to those who opt for non-surgical treatment only, the baseline HR-QoL is far lower in those

For peer review only - http://bmjopen.bmj.com/site/about/guidelines.xhtml

who select surgery, showing at least a perception of reduced quality of life in these individuals (10). To what extent the improvements seen in HR-QoL correlate with weight loss, complications and clinical outcomes is not known.

The complication rates of bariatric surgery are poorly reported in the literature, leading to polarised views on their true incidence. Mortality rates in the first year post surgery have been reported as being similar to those of BMI-matched controls (11); however, the same results may not be found today due to the decreased mortality rates as a result of primary preventative. There is little report of specific wound complications and re-operation rates, with a general belief that the low rates reported by the few studies releasing this data would not be replicated in routine practice.

1.3 Study hypothesis

This study is a registry of bariatric surgery and is therefore the study design is based on objectives but not hypotheses.

2 STUDY OBJECTIVES

SCOTS is a prospective observational cohort study of 450 patients undergoing bariatric surgery in Scotland which will be used to assess medium term outcomes and complications of bariatric surgery. This is in order to identify risks and complications of bariatric surgery and develop an evidence base to support clinical teams in building a patient centred, safe, effective and efficient bariatric surgery service.

2.1 Primary Objective

The SurgiCal Obesity Treatment Study (SCOTS) will establish the clinical outcomes and adverse events of different bariatric surgical procedures (taking in to consideration different care pathways), their impact on quality of life and nutritional status, and the effect on co-morbidities in a cohort of 450 patients.

2.2 Secondary Objectives

To establish in a cohort of obese patients who are undergoing bariatric surgery:

1. The physical and mental health and social burden of severe obesity.
2. Incidence of acute and chronic postoperative complications. Acute complications, defined as up to three months post-surgery, will include surgical site infection; chronic complications will include revisional surgery, plastic surgery and chronic pain, for different bariatric surgical procedures.

For peer review only - http://bmjopen.bmj.com/site/about/guidelines.xhtml

3. The effect of surgical experience and the pre and post-operative care pathway on complication rates and weight loss, for different bariatric surgical procedures.
4. The effect of the pre-surgical pathway and criteria on bariatric surgery patient selection. Change in health related quality of life, anxiety and depression over time pre- and post-operatively for a mean of 3 years from date of bariatric surgery.
5. The weight status pre and post-operatively for 3 years after bariatric surgery.
6. The glycaemic control, lipids, blood pressure, medication prescription, and rate of diabetes complications (microalbuminuria and renal disease, and retinopathy) in those that have pre-existing diabetes or develop diabetes for 3 years follow up since bariatric surgery.
7. Changes in socio-economic factors (employment, benefit receipt, sick leave, and health care use) for 3 years since bariatric surgery.

All the above objectives are due to be achieved by December 2020 (Start Jan 2012, 2 years development, 3 years recruitment finishing March 2017, mean 3 years post-operative follow-up finishing December 2020).

2.3 Primary Outcome

Change in weight from pre-bariatric surgery weight.

2.4 Secondary Outcomes

1. Incidence of physical and psychological comorbidity in a population seeking bariatric surgery

In the 3 years following bariatric surgery:

2. Change in weight/BMI from pre-bariatric surgery weight
3. Rate of incident type 2 diabetes
4. Change in incidence of depression and anxiety compared to baseline level pre-operatively
5. Incidence of complications immediately post-operatively and the need for readmission for revisional procedures
6. Change in health-related quality of life compared to baseline level pre-operatively
7. Change in glycaemic control, cardiovascular risk factors, CKD, retinopathy and medications prescribed in patients with diabetes compared to equally obese patient with diabetes who did not have bariatric surgery
8. Cost of the procedure and follow-up

Outcomes, including their source and frequency of measurement, are summarised in table 1.

For peer review only - http://bmjopen.bmj.com/site/about/guidelines.xhtml

3 STUDY DESIGN

3.1 Overview of study design

This 3 year prospective cohort study will collect data on the medium term outcomes of bariatric surgery in a minimum of 400 individuals. The study will be performed according to the Research Governance Framework for Health and Community Care (Second edition, 2006) (12). The information required to meet the objectives will be collected by a combination of sources including: bariatric surgery specific information entered by clinical teams; data linkage to routine clinical data and patient reported outcome measure questionnaires (PROMS). The outcomes and the data source are described in detail in Table 3-1.

TABLE 3-1: PRIMARY AND SECONDARY OBJECTIVES OF SCOTS DESCRIBING SOURCES OF OUTCOME DATA

	Outcome	Data source	Frequency
1	Incidence of physical and psychological comorbidity in a population seeking bariatric surgery.	SCI-DC Scottish National dataset, eDRIS at National Service Scotland, Patient reported questionnaires.	Continuous throughout study
2.1	Change in weight/BMI from pre-bariatric surgery	Clinical teams	At recruitment, annual check up
2.2	Rate of incidence of type 2 diabetes	SCI-DC Scottish National dataset	Continuous throughout study
2.3	Change in incidence of depression and anxiety compared to baseline levels pre-operatively	eDRIS at National Service Scotland, Patient reported questionnaires	Pre-operatively, ,2,3 years post operatively
2.4	Incidence of complications immediately post-operatively and the need for revisional procedures	Patient reported questionnaires, surgical teams	Pre-operatively, 2, 3 years post operatively, at time of re-operation.
2.5	Change in health related quality of life compared to baseline	Patient reported questionnaires	Pre-operatively, 2,3years post operatively
2.6	Change in glycaemic control, cardiovascular risk factors, chronic kidney disease (CKD), retinopathy, medications in patients with	SCI-DC Scottish National dataset	Pre-operatively, 2, 3 years post operatively, at time of re-operation.

For peer review only - http://bmjopen.bmj.com/site/about/guidelines.xhtml

	diabetes compared with non-obese cohort		
2.7	Cost of the procedure and follow-up (to inform cost effectiveness analysis)	Clinical teams, e-prescribing record linkage, patient reported outcomes	At time of patients operation, continuous linkage throughout the study

This protocol describes the objectives of the study, the sources of data collection for each objective, and the input required from patients and clinical teams throughout the study. A data collection portal will be developed for the study with secure logins for clinical teams and participating patients. A detailed user manual will be developed for the operation of this system will be produced by the Robertson Centre for Biostatistics - University of Glasgow.

A SCOTS web based portal will be developed which will present information on the study to the public and other researchers. The portal will contain secure logins to the Robertson Centre databases which will allow clinical teams to enter data. Clinical teams will be asked to register for a user ID and password. These will be issued once the team have undergone the necessary training for their delegated role. Everyone who has a user ID and login will be a member of the SCOTS investigators group.

The SCOTS portal will include an area which will allow patients who have agreed to complete the patient questionnaires to login securely and complete the patient reported outcome questionnaires. If patients prefer to complete the questionnaires on paper the data will be entered by staff at the Robertson Centre into the database. Patients will be kept up to date with progress of the study via updates on the study website. Patients will be sent reminders through their preferred route of communication to let them know when they are due to complete a questionnaire.

Patient identifiable information will be stored separately from the clinical outcome data. The information will be linked using a database code. Within the Robertson Centre individuals who can access patient information to send reminder letters will not have access to any clinical information. Any clinical information to be analysed will have all patient contact details remove. Production of any reports will follow the NHS Information and Statistics division (ISD) Statistical Disclosure Control protocol (13) to ensure that patient confidentially is maintained, (this is a requirement of the record linkage arrangement with ISD).

SCOTS has three distinct parts. All adult patients who are having weight loss surgery in Scotland will be invited to participate in three parts of the study. These parts are described in detail below.

For peer review only - http://bmjopen.bmj.com/site/about/guidelines.xhtml

3.1.1 Part 1 Record Linkage

The study will collect data by record linkage to patients' clinical outcomes. Information will be recorded by the clinical team on the operation they have had. They will be followed using their medical records until at least December 2020 and if funding is available for the rest of their life. This will include all hospital admissions, blood test results, diagnostic test results, diabetes complications, medications they are prescribed, pregnancy, psychiatry and mortality. This part of the study will observe patients care. The study will not alter their planned care in any way. No additional tests or treatments will be given to patients who consent to be part of the study. (This will fulfil the primary objective and objectives 1, 2, 3 and 4). If patients do not consent to participate in this part of the study they will not be asked to participate in Part 2 or Part 3.

Each record linkage requires approvals to access data including a favourable ethical opinion. These will be sought by the SCOTS Project team. A separate procedure for each data linkage will be developed by the Robertson Centre for Biostatistics. Patient identifiable information is required to identify study participants. The information required is patient CHI number, family name, given name, patient date of birth, gender, postcode, consent data and unique study identifier.

3.1.2 Part 2 Patient Reported Outcomes (PROMS) Questionnaire

We will ask patients if they are willing to complete a questionnaire which will include questions on quality of life, overall health and fitness, diet, and anxiety and depression, healthcare utilisation and ability to work. They will also be asked about problems they may have experienced since their surgery such as pain and surgical site infection post discharge.). Where patients are not completing PROMS and there is no reliable clinical weight record, we will write after 3 years requesting completion of a weight questionnaire (simply asking their current weight). The patients will be offered a voucher based incentive of £30 for completing both the year 2 and 3 questionnaires (high street voucher), and £10 for the year 3 weight follow-up questionnaire (for those no longer in clinical follow-up/not completing PROMS)

3.1.3 Part 3: Registration for a database of bariatric patients for future research

Patients who have agreed to Parts 1 and 2 will be asked if they are willing to be contacted about possibly taking part in other studies. This will not be an obligation to take part, just an expression of interest in being invited to take part. This will create a database of patients who have had bariatric surgery and are willing to consider participation in future research. Ethical opinion will be sought for the development of these study protocols.

For peer review only - http://bmjopen.bmj.com/site/about/guidelines.xhtml

3.1.4 Service user involvement

SCOTS will uphold the principles embodied by NIHR INVOLVE in relation to user involvement in research, to provide training and support, negotiated roles, time and equitable access for service users involved in this study. SCOTS recruited a patient focus group made of service users who have undergone different procedures both in NHS and private healthcare.

The SCOTS patient focus group have commented on the design of the study, in particular by providing advice on the acceptability of the patient reported outcome questionnaires, the burden to patients and on material and resources that will be given to potential participants. Future sessions are planned to discuss patient recruitment and retention strategies. SCOTS have included a service user on the SCOTS Steering Committee.

3.2 Study Population

All patients due to have a bariatric surgical procedure in Scotland will be invited to participate - both NHS and private sector.

A bariatric surgical procedure is defined as a surgical intervention which has the primary purpose of large scale weight loss in a patient who is obese. Currently those procedures include: duodenal switch; gastric banding; gastric bypass and sleeve gastrectomy. The inclusion of emerging techniques in this definition will be decided by the steering committee.

3.3 Reference populations

Within this protocol the objectives will be met by comparing before and after surgery outcomes for the SCOTS cohort. Additional funding shall be sought to develop protocols to allow comparisons with the following groups for a final Health Technology Assessment report.

1. SCOTS will compare outcomes with the IT based non-surgical weight management database in Greater Glasgow and Clyde (treatment seeking population with current data on 10,000 patients and 3,000 new referrals per year) which has prospective consent for research allowing data linkage for morbidity and mortality along with adjustment for smoking, deprivation, BMI, age and sex.

2. Standardised mortality ratios are available for a BMI matched cohort from the Scottish Health Survey.

For peer review only - http://bmjopen.bmj.com/site/about/guidelines.xhtml

3. The Scottish diabetes database allows an anonymous control group matched for key demographics to be created with prospective follow up of diabetes care and complications, compared to those with diabetes who had bariatric surgery.

3.4 Inclusion criteria

- Patients aged 16 years or over undergoing their first bariatric surgery in NHS hospitals or private practice in Scotland.
- Capacity to consent
- Resident in Scotland
- Written informed consent

3.5 Exclusion criteria

- Does not meet inclusion criteria
- Patients who have had previous weight loss surgery (at time of potential recruitment are undergoing a repeat procedure)
- No written informed consent
- Patients under 16 years
- Patients who do not have capacity to consent for themselves
- Weight loss surgery performed at a site outside Scotland
- Patients who live outside Scotland
- Patients who do not have strong enough English language skills to understand the patient information leaflet without assistance will be excluded from Part 2 of the study, the patient questionnaires. If they require a translator to understand the information they may be able to participate in Part 1 of the study which requires no further interaction from the patient. This decision will be made by the clinical teams caring for patients.

3.6 Identification of participants and consent

Patients will be prospectively identified by clinical teams as they are referred for surgery. Patients will be recruited from both NHS and private bariatric surgical practice in Scotland. They will either be recruited by a member of the surgical team prior to undergoing a bariatric surgical procedure, including any significant pre-operative weight loss (such as a very low energy diet for liver shrinkage or use of an intra-gastric balloon) which is considered part of the surgical pathway, or the surgical team will ask the patient's permission to pass over their name and contact details to the SCOTS research team who will then contact the patient directly to give further information and obtain fully informed consent.

For peer review only - http://bmjopen.bmj.com/site/about/guidelines.xhtml

Depending on local patient pathways patient information sheets will be provided to patients at a clinic visits or group session. This will be provided at least 24 hours before the patient will be asked to consent. In the case of those patients who are to be recruited by the research team (identified by surgical team), a patient information sheet will be provided when the permission is sought to hand over contact details to the research team. Patients who meet the eligibility criteria will be identified by their local clinical care teams. All patients undergoing bariatric surgery will be recorded on the screening log at their next appointment. This will allow patients to consider their participation and ask any additional questions they may have about the study face to face. An independent contact is provided on the patient information leaflet who patients can contact to discuss participation in research studies.

They will be asked to give fully informed consent for clinical data linkage (Part 1) and to be contacted by Glasgow University by post, email or phone about to completing the Patient questionnaires (Part 2) and for permission to contact them about future studies (Part 3). Patients who require a translator will only be asked to participate in Part 1. Only patients who agree to Part 1 will be eligible for Part 2 or Part 3.

3.7 Withdrawal of subjects

Participants have the right to withdraw from the study at any point for any reason. The SCOTS web site and patient information sheets will provide contact details for patients if they wish to withdraw from the study. If patients have asked to complete the forms by paper, contact details will be included within each paper questionnaire. The SCOTS team will provide patients with a form to complete, if they are contacted with a request to withdraw from the study.

If the SCOTS team are contacted by a patient who no longer wishes to participate in the study, they will be contact by the SCOTS team at University of Glasgow and asked to specify which following options they would like to apply to their data:

A. (For those within Part 1) I no longer wish to continue the study but I am content to allow my existing data to remain within the study for analysis.

B. (For those consented to Part 1 and 2). I am content for my records to be linked but I no longer wish to complete the patient questionnaires.

C. (For those consented to Part 1, 2 and 3). I am content for my records to be linked but I no longer wish to complete the patient questionnaires or be contacted regarding future studies.

D. For any patient I would like to completely remove my all data from the study and wish not to be contacted again by the study.

For peer review only - http://bmjopen.bmj.com/site/about/guidelines.xhtml

When a request is received from a patient to withdraw from the SCOTS study, a standardised form will be sent to the patient by their preferred communication route. The form will be completed by the patient including the patients name date of birth and address, and the on receipt data manager will flag the patient as having withdrawn from the study. The patient will be asked if they would like to be informed of the findings of the study even though they will no longer be participating in the study.

4 STUDY PROCEDURES

The study design involves local clinical teams identifying and potentially recruiting participants (some participants will be recruited by the SCOTS research team after identification by the local clinical team). Patients who consent to participate will have recruitment data collected by their clinical care teams or the SCOTS research team. Following their operation patients will have their weight loss surgery details entered into the database. The clinical teams then use the system to include follow up weights and re-operations. For the remainder of the study, patients who have consented to part 2 of the study will be asked to complete questionnaires and their health outcomes will be recorded by record linkage to routine data sets. A detailed breakdown of patient contacts with the study is described in Table 4-1 below.

TABLE 4-1: SCOTS PATIENT VISIT SCHEDULE: DESCRIBES EACH INTERATION WITH PATIENTS IF THEY CONSENT TO PART 1 AND 2 OF THE STUDY.

Patient contact	Time	Routine care	SCOTS	Part 1	Part 2	Part 3
1	1 year to 6 weeks before surgery	Agree bariatric surgery	Patients given/sent Invitation to participate letter and patient information sheet (and asked if they are to be contacted by SCOTS research team for further information and informed consent if no local recruitment)	1	1	
2	1 year to 6 weeks before surgery	Patient pre-surgical clinic visit, dietician, psychologist if local recruitment; phone call from SCOTS research team member if research team recruitment	Patients asked if they are willing to consent to participate in SCOTS, Patient signs consent forms, clinical team and patient complete contact details and baseline height and weight.	1	1	
3	At least one month before surgery		Patient contacted by SCOTS team to complete pre-operative questionnaire	1	1	
4	Date of surgery	Patient has weight loss operation	Clinical team enters details of weight loss operation to SCOTS portal	1	1	

For peer review only - http://bmjopen.bmj.com/site/about/guidelines.xhtml

5	Patient admitted to hospital	Identified by record linkage	If patient hospital admission is possibly related to bariatric surgery, patient will be contacted by SCOTS Team.	1	1	
7	At routine follow up visits	Patients attend routine clinical visits	Clinical teams enter weight and re-operation details		1	
8	Two year post surgery	Patients have routine annual blood test and results are made available via SCI-store.	Patients complete two year post surgical questionnaire		1	
9	Three years post surgery	Patients have routine annual blood test and results are made available via SCI-store.	Patients complete three year post surgical questionnaire		1	
10	Any point after recruitment		Possibly contacted to participate in future studies			1
11	Three years onward	Patient continues to have routine annual blood test every year	Patient contacted to thank them for completing the PROMS. Record linkage continues. Patients will be informed about future SCOTS publications.		1	
Total				5	9	1

Figure **4-1** shows what happens at each point within the study. This includes background record linkage and patient contacts.

FIGURE 4-1: BASELINE – PRIOR TO OPERATIVE WEIGHT LOSS (INCLUDING PRE LIVER-PREP DIET OR BALLOON PHASE)

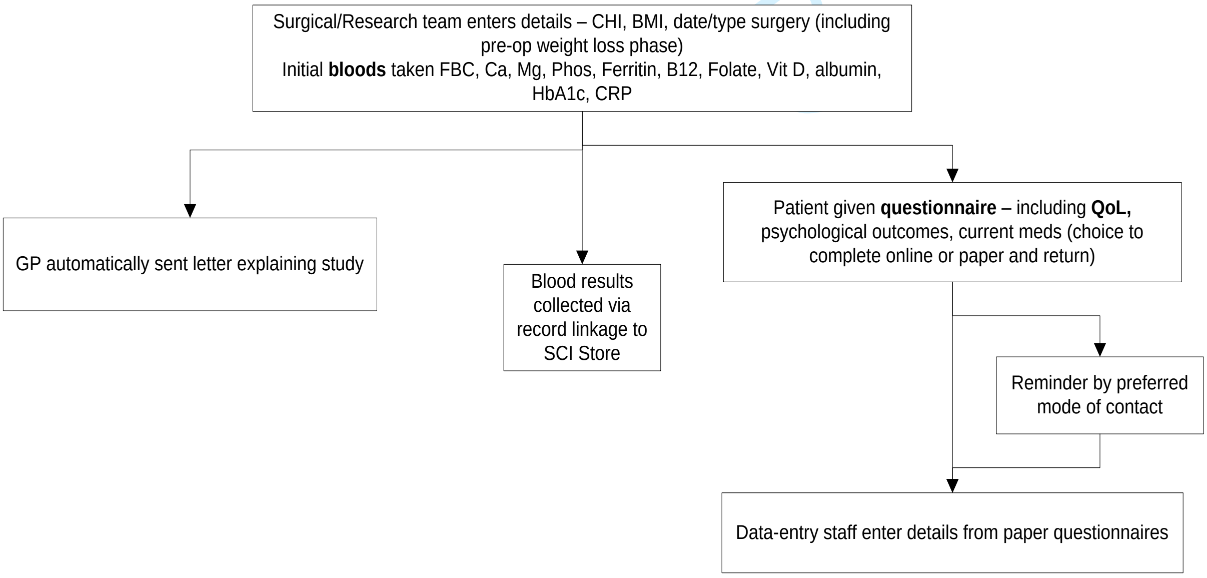

For peer review only - http://bmjopen.bmj.com/site/about/guidelines.xhtml

For peer review o

FIGURE 4-2: 2 AND 3 YEARS POST DEFINITIVE SURGERY

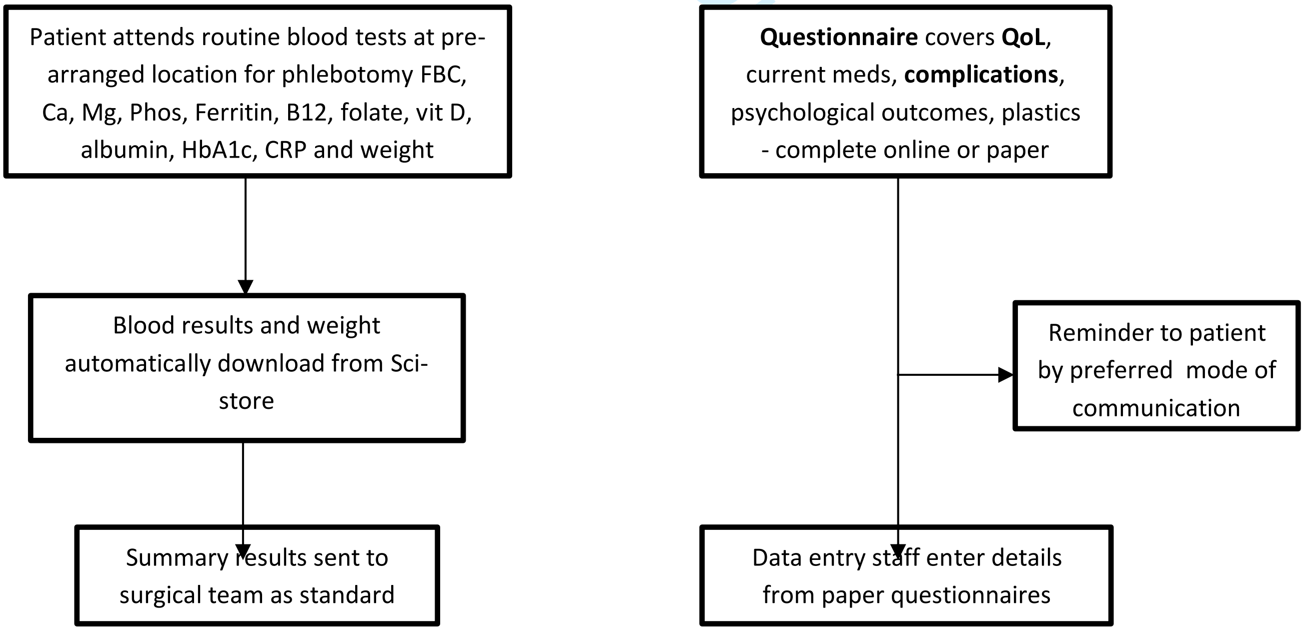

For peer review only - http://bmjopen.bmj.com/site/about/guidelines.xhtml

FIGURE 4-3: PART 3 IF NEW BARIATRIC SURGERY STUDY BEGINS

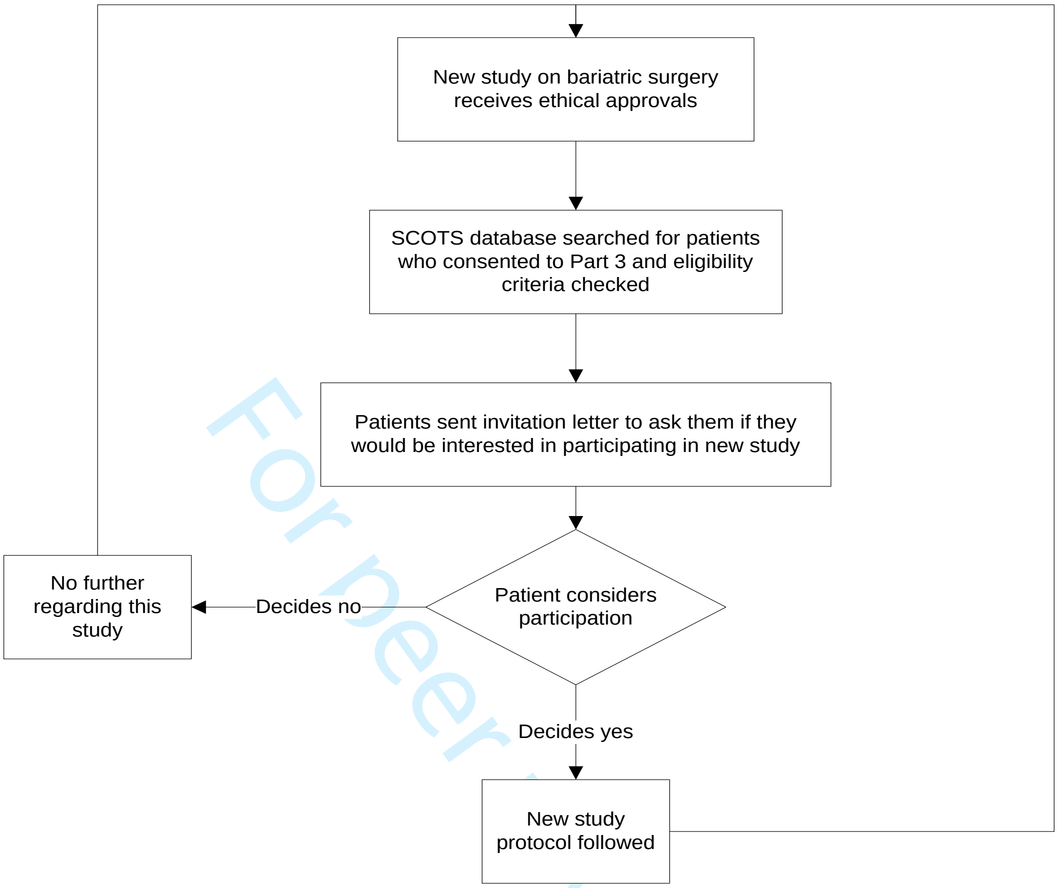

4.1 Duration of participation

Part 1: if funding is available, record linkage, follow up of patients will continue throughout their life. The end of a patient's life will be shown by linkage to the GRO data set. This will be used to flag deceased patients.

Part 2: patient reported outcome measures (PROMS) questionnaires will be collected for a maximum of 3 years. Collection of PROMS will stop after December 2020.

Part 3: Patient contact details will be kept by the study team until the end of the patient's life. The end of a patient's life will be shown by linkage to the GRO data set. This will be used to flag deceased patients and they will be removed from the database in Part 3 to ensure patients families are not contacted after their death. Patient will only be contacted for future studies if record linkage is available to show that they are still living.

Patients will be able to withdraw from the study at any point. Data collected will be retained unless explicitly requested to be removed from the study by the patient.

For peer review only - http://bmjopen.bmj.com/site/about/guidelines.xhtml

4.2 Study schedule

This section provides a brief overview of the procedures for investigators at each point within the study. SCOTS will integrate into routine care pathways for bariatric surgery as much as possible. No additional clinic visits will be required. There are five points within the patient journey that the clinical/research team are required to undertake any tasks. These points are described in detail in the following sections.

4.2.1 Start of Study - Surgical team registration

Each site will be defined before the start of the study and login and passwords will be created for each principal investigator and their clinical teams.

A training plan will be developed for clinical teams. Clinical teams will be provided with an overview of the study provided with a training package which will be developed electronically, to be completed at their convenience, electronic guidance will be integrated into the data collection tool to assist them. A user manual will be created which will explain the data which is required for each field and define each data field.

An electronic form will be used to collect surgeon specific data the first time they log onto the bariatric data collections system. This form will be developed by the study Data Centre at the Robertson Centre for Biostatistics, University of Glasgow and access to the form will be restricted, with only authorised site-specific personnel able to make entries or amendments to their data. A manual will be developed to assist investigators with use of the electronic data collection system. It is the investigator's responsibility to ensure completion and to review and approve all data captured. Surgeons will be asked to outline which locations they work in, which procedures they use, and the details of these procedures. They will also be asked information on their surgical experience including date when they qualified, number of procedures the previously year, total number of procedures undertaken in the last five years.

The aim of this task is to ensure that surgeons' data is entered at the start of the study and patient records can be pre-populated to ensure that data can be collected as efficiently as possible.

Checklist:	
Surgeon registers on SCOTS system	
Surgeon enters names and email addresses of clinical team who will be part of SCOTS investigators team	
Investigators have the opportunity to view SCOTS training materials	
SCOTS resource pack is available at the site	

For peer review only - http://bmjopen.bmj.com/site/about/guidelines.xhtml

4.2.2 Visit 1 Routine care invitation visit

Patient pathways vary throughout Scotland, invitation and introduction should take place at either an individual's clinic visit or at a group session discussing weight loss surgery. The aim of this visit is to ensure that patients who are planning weight loss surgery are aware of the SCOTS study, why it is being undertaken and what would be required from them.

4.2.2.1 Invitation visit protocol (in sites with local team recruitment)

1. Patient attends routine clinic to discuss bariatric surgery with clinical team.
2. Patient provided with an overview of SCOTS
3. Patient provided with opportunity to ask initial questions
4. Patient provided with invitation to participate in SCOTS and patient information leaflet (pre-prepared pack).

Checklist:	
Patients provided with overview of SCOTS	
Patient provided with SCOTS information pack	

4.2.2.2 Invitation visit protocol (in sites with SCOTS research team recruitment)

5. Patient attends routine clinic to discuss bariatric surgery with clinical team.
6. Patient provided with a summary overview of SCOTS
7. Patient provided with invitation to participate in SCOTS and patient information leaflet (pre-prepared pack).
8. Patient asked permission for surgical team to pass on contact details to SCOTS research team
9. Participant Identification form completed and returned to research team

Checklist:	
Patients provided with a summary overview of SCOTS	
Patient provided with SCOTS information pack	
Permission sought and Participant Identification Form completed and returned	

4.2.3 Visit 2 Recruitment visit

At the next follow up clinic appointment patients will be asked if they wish to take part in SCOTS. Patients must have been given more than 24 hours to consider their participation.

For peer review only - http://bmjopen.bmj.com/site/about/guidelines.xhtml

For patient's in sites where recruitment is carried out by the SCOTS research team the following will occur after the research team receive the participant information form from the local site:

1. SCOTS research team contact patient via telephone
2. Patient provided with an overview of SCOTS
3. Patient provided with opportunity to ask initial questions

Details of all patients who plan to have bariatric surgery will be recorded on a screening log. These logs will recorded on paper. Every patient who plans bariatric surgery will be logged anonymously with details of the individual's age category on date of invitation, gender, calendar quarter surgery planned, if they meet the SCOTS eligibility criteria, if they consent and type of surgery planned (for participants identified via local sites but recruited by the SCOTS team only those giving permission for their details to be forwarded to local teams will have a screening log completed as the research team shall do this). The purpose of this log is to demonstrate if the SCOTS data set is representative of all patients who are having bariatric surgery in Scotland. It will also provide a record of number of patients who consent. A copy of the screening logs will be sent to the Robertson Centre on an annual basis.

This is an anonymised screening record and patients who do not wish to participate will have no further information collected.

4.2.3.1 Informed consent protocol (in sites with local team recruitment)

1. Ask the patient if they have any questions relating to the study documentation. Ensure that the patient fully comprehends the information given.
2. Explain that participation is voluntary and should they decide to proceed with participation in the study, they are free to withdraw at any time without giving an explanation.
3. If a patient chooses to decline, thank them for their time and allow them to leave.
4. If they are unsure, allow further time to consider and arrange a convenient date to meet again reiterating that their participation is voluntary. Offer the option to speak to another member of the study team or an independent advisor.
5. All the check boxes within the consent form must be initialled by the patient to confirm they understand each part of the study. Ask the patient to initial each part of the consent form and sign the bottom section adding their name in block capitals.
6. The person obtaining informed consent signs and dates the consent forms adding their name in block capitals.
7. If a witness is required they can also sign and date the consent form (this may be in the case where an interpreter is required).
8. One copy of the form should be provided to the patient, one filed in the patient's notes and one copy stored in the site folder, and a copy sent to the Robertson Centre for the study files. A copy of the version of the patient information leaflet which was given to the patient should be filed within the site study file.

For peer review only - http://bmjopen.bmj.com/site/about/guidelines.xhtml

9. If the patient consents to the study the patient recruitment form should be completed in the presence of the patient. Record patient name and CHI number (for NHS patients), sex, height and weight.
10. Patients should be asked to complete the contact details part of the form including their e-mail and mobile phone contact details and ensure that they identify their preferred route of communication.
11. The Patient recruitment form will be send to the Robertson Centre and a copy filed within the site study file; When they are sent to the Robertson Centre a form will be faxed to show what documentation has been sent in the post, this will be recorded in a postage log.
12. Paper copies of the screening log are returned to the Robertson Centre annually, in self addressed envelopes provided by the SCOTs project team. These will also be recorded in a postage log.

Checklist:	
Check patient name and date of birth correct on patient recruitment form	
Consent from signed and dated at each point	
Patient retains copy of consent form	
Copy of consent form filed in notes	
Copy of consent form in study file	
Copy of consent form in study file sent to Robertson Centre	
Copy of patient recruitment form sent to Robertson Centre	
Copy of patient recruitment form filed in study file	
Consent form and patient recruitment form entered on postage log	

4.2.3.2 Informed consent protocol (in sites with SCOTS research team recruitment)

1. When the Participant Identification Form is received from the surgical team start to complete the SCOTS Recruitment Log and contact the patient via telephone.
2. Ask the patient if they have any questions relating to the study documentation. Ensure that the patient fully comprehends the information given.
3. Explain that participation is voluntary and should they decide to proceed with participation in the study, they are free to withdraw at any time without giving an explanation.
4. Complete the screening log (do not add participant ID as form is to be anonymous)
5. If a patient chooses to decline, thank them for their time and allow them to leave.
6. If they are unsure, allow further time to consider and arrange a convenient date to phone again reiterating that their participation is voluntary. Offer the option to speak to another member of the study team or an independent advisor.
7. All the check boxes within the consent form must be initialled by the patient to confirm they understand each part of the study. Ask the patient to initial each part of the consent form and sign the bottom section adding their name in block capitals.
8. For participants should then be asked post the completed consent form back to the research team in the stamped addressed envelope provided.

For peer review only - http://bmjopen.bmj.com/site/about/guidelines.xhtml

If the research team does not receive the completed consent form from the patient within two weeks and cannot reach the patient by telephone, a follow up letter should then be sent.

9. On receipt of the signed form, the person that obtained informed consent signs and dates the consent forms adding their name in block capitals.
10. If a witness is required they can also sign and date the consent form (this may be in the case where an interpreter is required).
11. One copy of the form should be provided to the patient (posted back in case of SCOTS team recruitment), one filed in the patient's notes and one copy stored in the site folder, and a copy sent to the Robertson Centre for the study files. A copy of the version of the patient information leaflet which was given to the patient should be filed within the site study file.
12. If the patient consents to the study the patient recruitment form should be completed with the patient via the telephone. Record patient name and CHI number (for NHS patients), sex, height and weight.
13. Patients should be asked for their contact details including their e-mail and mobile phone contact details and ensure that they identify their preferred route of communication.
14. The Patient recruitment form will be send to the Robertson Centre and a copy filed within the site study file; When they are sent to the Robertson Centre a form will be faxed to show what documentation has been sent in the post, this will be recorded in a postage log.
15. The Letter to Inform Surgeon of Participation should be completed and sent to the surgeon along with the relevant operation details recording form.
16. Paper copies of the screening log are returned to the Robertson Centre annually, in self addressed envelopes provided by the SCOTS project team. These will also be recorded in a postage log.

Checklist:	
Check patient name and date of birth correct on patient recruitment form	
Consent from signed and dated at each point	
Patient sent copy of signed consent form	
Copy of consent form filed in notes	
Copy of consent form in study file	
Copy of consent form in study file sent to Robertson Centre	
Copy of patient recruitment form sent to Robertson Centre	
Copy of patient recruitment form filed in study file	
Consent form and patient recruitment form entered on postage log	

For peer review only - http://bmjopen.bmj.com/site/about/guidelines.xhtml

4.2.4 SCOTS study team contact patient

If the patient has consented to Part 2 of the study, the patient will be contacted by staff from the Robertson Centre by their preferred route of communication to invite them to complete a patient reported outcomes questionnaire. They will be provided with a username and initial password which will need to be changed the first time they login to their account. The preadmission questionnaires will be available for the patient to complete. Patients will be able to complete the questionnaire in one or more sittings. If the patients have not logged into the system within two weeks of receiving their information they will be sent a reminder message by their preferred mode of contact. Given the smaller numbers recruited, the patients are now being offered a voucher based incentive to ensure a good return of the two and three year questionnaires. They will be offered an incentive of £30 for completing both the year 2 and 3 questionnaires, and £10 for the year 3 weight follow-up questionnaire (for those no longer in clinical follow-up/not completing PROMS).

Patients will be contacted two and three years after their weight loss operation. Patients will be contacted as part of the study closure phase to thank them for their participation in the study and inform them that they are no longer required to complete the questionnaires.

4.2.5 Visit 3 Weight loss operation

After the patient has their weight loss operation, the clinical teams will enter data onto the SCOTS data collection tool. This will involve the team member who has been delegated to record surgical information on the system. They will log in and be asked for the location of the surgery, which surgeon operated on the patient and their usual operation details will be pre-populated. If the operation is in any way different from the surgeon's usual technique, an edit function will allow the exact details of the operation to be recorded.

If the operation is a re-operation on an existing SCOTS consented patient, the SCOTS database will be searched to allow the patient's re-operation to be recorded.

4.2.6 Visit 4 -Post operative follow up

When patients attend follow up visits to see their surgeon, clinical teams will record the patient's weight and any complications experienced by the patient. If the patient has a gastric band, clinical teams will be able to record band adjustments in the data collection system. Essential fields for the follow-up visits are date of appointment and weight. Within the SCOTS portal it is possible for clinical teams to produce individual reports for patients on their weight loss for inclusion in their medical records, in the letters to GPs or for the patients themselves.

For peer review only - http://bmjopen.bmj.com/site/about/guidelines.xhtml

4.2.7 Visit 5 - Re-operation (If applicable)

If a patient requires a re-operation, surgeons will be able to record details of the latest procedure and the reasons for the re-operation. This can happen at any time throughout the study. Details of how the surgeons will record re-operations will be included within the user manual.

For peer review only

For peer review only - http://bmjopen.bmj.com/site/about/guidelines.xhtml

5 STATISTICS AND DATA ANALYSIS

5.1 Statistical Analysis Plan

The study will have separate detailed Statistical Analysis Plans to cover each analyses required for reporting to study committees, for publications and for the final report to the study funder, which will be authored by the study statistician and agreed by the Study Steering Committee before analysis takes place.

An important goal within the funded period is to build various predictive models to use accumulating information to predict good and bad outcomes, so that clinicians can identify treatment opportunities and make better informed intervention management decisions. Generically, these prediction models will relate potentially predictive information – both on entry into the SCOTS database on and around diagnosis, and from subsequent visits and at surgical and other treatment interventions – to outcomes of interest. The mainstay will be Cox proportional hazards time-to-event models, incorporating demographic, clinical, laboratory and life event data (e.g. hospitalisations, strokes, cancers, non-weight loss surgery, and so on) as both fixed and time varying covariates. The influence of missing data on the robustness of predictions in these models will be assessed. Importantly, a hierarchy of models will be built, with ease of use for the clinician in mind, from very simple models built from easy to obtain information, through to more complex models with very many and possibly some difficult and/or expensive to measure covariates.

5.2 Software for statistical analysis

The statistical software to be used for each analysis will be specified in the relevant Statistical Analysis Plan

5.3 Sample size

At the time of grant proposal writing, 230 operations were funded in NHS Scotland each year (of which approximately 60 are bypass). Bariatric surgeons performed an additional 270 private procedures per year and they were willing to commit to entering data (approximately 80 bypass). Therefore 500 procedures per year were expected to be entered into the database with a belief that as numbers of people with severe obesity (BMI >40) are rising rapidly, this number will increase despite financial constraints.

Previous recommendations from the NHS Scotland national planning forum (15)recommended that each health board should aim to provide 9 operations/100,000 population. Scotland's estimated population on the 30th June 2011 was 5,254,800 (16) (http://www.gro-scotland.gov.uk/files2/stats/population-estimates/mid-2011/j22829703.htm). This would mean around 470 operations per year within the NHS alone. This figure was expected to be reached over a number of years. Within private

For peer review only - http://bmjopen.bmj.com/site/about/guidelines.xhtml

healthcare bariatric surgery was estimated to be around 270 per year in Scotland (reported by personal communications from bariatric surgeons).

Given the potential for a surgical learning curve (17), new surgeon's data will be recorded but not analysed for the primary outcome until they have performed 50 operations. Collection of this early data will provide important information on the effects of the learning curve on patient outcomes.

From previous studies (3;4) we expected a 10-year mortality of around 5% (100 deaths). This sample size would allow the mortality rate to be estimated with 95% confidence to within +/- 1% (i.e. for a 5% 10-year death rate, the 95% confidence interval will be between 4% and 6%). We planned to compare this mortality rate with an age-sex matched healthy population from the Registrar General of Scotland's life tables (assumed known with no sampling error). 100 deaths was sufficient to allow us to build a predictive model for death post surgery (conventionally one requires around 10 events per prognostic covariate considered). An initial sample size of 2000 was proposed as it would easily provide adequate power for the original outcomes under investigation.

However, there have been a number of unforeseen recruitment issues that have impacted on the numbers stated above. In order to explore whether the current sample is likely to show meaningful results, the available statistical power for detecting 3 year difference in HbA1c and quality of life (physical and mental components) was calculated using the numbers of participants available as of 29th of July 2016. The majority of data to inform sample size were taken from papers where bariatric procedure was laparoscopic adjustable gastric banding (LAGB) as this is recognised as the least effective of the bariatric procedures so will give a conservative estimate of measure of effect. This shows that there is >99% power to show differences in these outcomes at three years with the current sample size.

In order to explore the likely event rate for cardiovascular events and deaths, we performed health record linkage for currently recruited participants, linking with inpatient care and death records. Follow-up is from the date of surgery, so for those patients that went on to have surery details entered (n=180), there are 272 separate admissions. Of those 272, there are 72 'emergency' admission, and 4 of these, in 3 patients, are 'circulatory disease' using the main condition only. The codes included for these hospital admissions are;

· Angina pectoris (x1)
· Acute MI (x1)
· Pulmonary embolism without mention of acute cor pulmonale (x1)
· Orthostatic hypotension (x1)

Using date of operation as the starting point, we have a total (crude) follow-up time of 203.52 years (the mean is 1.13 years and the median is 1.04 years). As the number of cardiovascular events is so low, it is impossible to extrapolate this to a future event rate at this time. It should be noted that the participants have been cleared as healthy for elective surgery meaning that it is unlikely that there would be many cardiovascular events in early follow-up.

For peer review only - http://bmjopen.bmj.com/site/about/guidelines.xhtml

Following discussions with the NIHR it was agreed that recruitment will stop at approximately 400 patients and these numbers will be sufficient to answer the majority of objectives initially set.

5.4 Management and delivery

The Robertson Centre for Biostatistics, part of the Glasgow Clinical Trials Unit, a fully registered UK CRN Clinical Trials Unit, will manage and analyse trial data.

For peer review only

For peer review only - http://bmjopen.bmj.com/site/about/guidelines.xhtml

6 STUDY CLOSURE

Part 1 Record linkage: under the current funding record linkage will continue until December 2020. If additional funding is available Part 1 record linkage will continue until the end of the patient's life. The end of a patient's life will be shown by linkage to the GRO data set. This will be used to flag deceased patient and they will be removed from the database in Part 3 to ensure patients families are not contacted after their death.

Part 2 Patient reported Questionnaires: The study will end when the steering committee agrees that one or more of the following situations applies:

- Observation has been undertaken until December 2020
- Sufficient patients have completed the 3 year post surgery questionnaire

Patients will be contacted to thank them for their participation in the patient reported outcomes part of the study.

Part 3 Bariatric Surgery Research Studies. Patients contact details will be maintained until the end of the patient's life.

7 Data Handling

Data for the SCOTS study will be collected from a number of sources. These are summarised in Table 3-1. A detailed data management plan will be developed by the Robertson Centre team, which will include verification of paper data entry and validation checks. A separate protocol will be developed for each data linkage.

7.1.1 Surgeon registration data

An electronic data collection form will be developed by the study Data Centre at the Robertson Centre for Biostatistics, University of Glasgow. These data will include the surgeons' usual procedures and registration of the clinical teams. These data will be used to complete the operation details for each patient. It will be the investigators responsibility to ensure that these data are entered correctly and are kept up to date.

7.1.2 Patient recruitment data

A paper case report form (CRF) will be used to collect patient contact details and height and weight at time of recruitment will be completed by the clinical teams when the patient has consented to participate in SCOTS. Patients will be asked to specify their preferred form of communication throughout the study. They will be told that they may be contacted via another route if they do not respond. This CRF will be developed by the study Data Centre at the Robertson Centre for Biostatistics, University of Glasgow. Competed forms will be checked by the clinical teams and sent to the Robertson Centre for initial data entry. These forms will be sent along with signed consent forms. It is the investigator's responsibility to ensure completion and to review and approve all data captured in the CRF. A standard

For peer review only - http://bmjopen.bmj.com/site/about/guidelines.xhtml

operating procedure will be used by the Roberson Centre to ensure that information is entered into the SCOTS database in an accurate and timely manner.

7.1.3 Surgical data collection

An electronic case report form (e-CRF) will be used to collect data on weight loss surgery procedures. On or shortly after the patient undergoes weight loss surgery a member of the clinical team will record specific information on the patient's weight loss operation. Each surgeon will have been asked to register the locations they operate in, which procedures they perform and details of their usual specifications. This will then populate the fields for a particular type of surgery and clinical teams will be asked to confirm that the usual techniques have been applied for each procedure. Access to the e-CRF will be restricted, with only authorised site-specific personnel able to make entries or amendments to their patients' data. It is the investigator's responsibility to ensure completion and to review and approve all data captured in the e-CRF, however, a member from the SCOTS study team, with the appropriate NHS Research Passport permissions, will visit the site staff at least once per year to ensure surgical and follow-up data are complete. Data will be validated at the point of entry into the e-CRF and at regular intervals during the study. Data discrepancies will be flagged to the study site and any data changes will be recorded in order to maintain a complete audit trail (reason for change, date change made, who made change).

7.1.4 Patient Reported Outcomes

An electronic case report form (e-CRF) or a paper case report form (depending on patient preference) will be used to collect patient reported study data. The e-CRF and CRF will be developed by the study data centre at the Robertson Centre for Biostatistics, University of Glasgow and access to the e-CRF will be restricted, with only authorised site-specific personnel able to make entries or amendments to their patients' data. All data handling procedures will be detailed in a study specific Data Management Plan.

7.1.5 Routine clinical information

A number of features have been built into the SCOTS system which will be available for surgeons to use during routine follow up visits. Each time a patient attends, their weight will be recorded. Notes can be included and exported for inclusion within patient notes, provided for patients or within letters to GPs.

7.1.6 Re-operation

An electronic case report form (e-CRF) will be used to collect data on re-operations. On or shortly after the patient undergoes weight loss surgery a member of the clinical team will record specific information on the patients repeat weight loss operation. Not only will the details of the operation be recorded but the reason for operation will be included within the e-CRF.

For peer review only - http://bmjopen.bmj.com/site/about/guidelines.xhtml

7.1.7 Record Retention

To enable evaluations and/or audits from regulatory authorities, the investigator agrees to keep records, including the identity of all participating subjects (sufficient information to link records), all original signed informed consent forms, source documents, and detailed records of treatment disposition in accordance with ICH GCP, local regulations, or as specified in the Clinical Study Agreement, whichever is longer. Data will be retained at the Robertson Centre once the recruitment phase of the study is complete. During recruitment a copy of the patient consent form and the patient registration will be retained within the site file and within the patient notes.

7.1.8 Access to data

Clinical teams will be able to access their own data. All patients who have been entered within the SCOTS system will be viewed by the clinical teams who entered data. Data which is routinely collected for the National Bariatric Surgery Registry (NBSR) can be entered within the system for surgeons who wish to contribute to this process. Surgeons fully anonymised data (no patient identifiable information) can be exported to a file which surgeons can share with the NBSR if they surgeons wish to contribute. The management and approvals for this will be responsibility of the investigating surgeon and data will not be shared by the SCOTS team.

For peer review only

For peer review only - http://bmjopen.bmj.com/site/about/guidelines.xhtml

8 SCOTS STUDY MANAGEMENT

8.1 Study Sponsor

NHS Greater Glasgow and Clyde Research and Development Office as study sponsors are responsible for the management and conduct (according to GCP). The SCOTS steering committee (SSC) oversee the management and conduct of the study and report to and advise the sponsor on the study. All minutes from both the SCOTS management group (SMG) and SCOTS steering committee (SSC) are copied to NHS Greater Glasgow and Clyde Research and Development Office

8.2 SCOTS Study Team

A study team comprising of the chief investigator, a research lead and a project manager, in collaboration with the data management team based at the Robertson Centre will run the project on a day to day basis. Issues arising from patients or investigators will be managed by this team and those that cannot be resolved by that team will be discussed with the chief Investigator and escalated to the study management group for discussion.

8.3 SCOTS Principal investigators

Each site will have an identified principal investigator who will oversee the application of this protocol locally. Each member of the clinical team involved in the study will be required to register for a login and password to access the e-CRF through the SCOTS data collection portal. Any member of the local clinical teams who has registered username and password and entered data to the SCOTS data collection portal will be a member of the SCOTS Investigators Group. The investigators will be required to enter some initial registration data to link themselves with the surgeons they work with. Planned publications outlined in Table 15-1 will be written on behalf of the SCOTS Investigators Group.

8.4 Routine management of study: SCOTS Management Group (SMG)

The trial will be coordinated from the University of Glasgow by the SCOTS management group (SMG). The SMG will consist of the principal investigator, other co-applicants, project manager and representatives from the Clinical Trials Unit. The role of the group is to monitor all aspects of the conduct and progress of the trial, ensure that the protocol is adhered to and take appropriate action to safeguard participants and the quality of the trial itself. It will be responsible for the day to day running of the trial and budget. It will meet at least 6 monthly, with more meetings initially and as required, and provide information to the steering committee as to the progress of the study.

For peer review only - http://bmjopen.bmj.com/site/about/guidelines.xhtml

8.5 SCOTS Steering Committee (SSC)

SCOTS Steering Committee (SSC) will be established to provide overall supervision of the study and ensure adherence to GCP. It will have an independent chair, a patient representative, and at least two other independent academics along with the principal investigator. Observers from the HTA programme will be invited to SSC meetings, and all SSC papers will be supplied to the HTA programme. The SSC will meet at the start of the study, and annually thereafter.

The role of the SSC is to provide overall supervision of the trial and ensure that it is being conducted in accordance with the principles of GCP and the relevant regulations. The SSC will:

- agree the trial protocol and any protocol amendments
- provide advice to the investigators on all aspects of the trial
- include members who are independent of the investigators, in particular an independent chairperson.

Decisions about continuation or termination of the trial or substantial amendments to the protocol are usually the responsibility of the SSC who advise the sponsor.

For peer review only

For peer review only - http://bmjopen.bmj.com/site/about/guidelines.xhtml

9 STUDY MONITORING/AUDITING

This study is a cohort study. It will therefore be audited on random sampling basis by GG&C auditors on behalf of the GG&C sponsor who audit 10% of sponsored studies per annum.

10 PROTOCOL AMENDMENTS

Any change in the study protocol will require an amendment. Any proposed protocol amendments will be initiated by the CI following discussion with the SSC and any required amendment forms will be submitted to the regulatory authority, ethics committee and sponsor. The CI and the SSC will liaise with study sponsor to determine whether an amendment is non-substantial or substantial. All amended versions of the protocol will be signed by the CI and sponsor representative. Before the amended protocol can be implemented favourable opinion/approval must be sought from the original reviewing Research and Ethics Committee (REC) and Research and Development (R&D) office(s).

11 ETHICAL CONSIDERATIONS

11.1 Ethical conduct of the study

The study will be carried out in accordance with the World Medical Association Declaration of Helsinki (1964) and its revisions (Tokyo [1975], Venice [1983], Hong Kong [1989], South Africa [1996] and Edinburgh [2000]).

Favourable ethical opinion will be sought from an appropriate REC before patients are entered into this study. Patients will only be allowed to enter the study once they have provided written informed consent.

The CI will be responsible for updating the REC of any new information related to the study.

11.2 Informed consent

Patients will be informed of the SCOTS study as they discuss weight loss surgery with their clinical teams. They will be provided with an information sheet on the study by a trained member of the clinical teams during a routine clinic visit or during a group session depending on the local patient pathway. The patient will be given time to consider their participation in the study, review the SCOTS information sheet and ask about any concerns they may have. Patients will then be asked to sign the SCOTS consent form.

For peer review only - http://bmjopen.bmj.com/site/about/guidelines.xhtml

12 INSURANCE AND INDEMNITY

SCOTS is sponsored by NHS Greater Glasgow & Clyde and NHS indemnity is provided under the Clinical Negligence and Other Risks Indemnity Scheme (CNORIS). In addition, as the protocol authors are employees of the University of Glasgow negligent harm caused by the design of the trial will be covered by the Clinical Trials Insurance policy held by the University of Glasgow.

Investigators working in private healthcare will recruit to this NHS funded study as part of their NHS role and will therefore be covered as detailed above.

13 FUNDING

Funding source: National Institute for Health Research (NIHR) Health Technology Assessment Programme

Grant Award number: 10/42/02

Original Cost: £2,124,030

Amended Cost: £1,516,255.60

URL of this project on the NIHR Website: http://www.hta.ac.uk/project/2608.asp

Start Date: January 2012

End date: June 2021.

Estimated date of publication in HTA journal series: Late 2021. This date takes account of time for report preparation and printing based on current average times for these activities.

International Standard Randomised Controlled Trial Number: ISRCTN 47072588

URL of this project on the Controlled Trials Website: http://www.controlled-trials.com/ISRCTN47072588

14 ANNUAL REPORTS

A bi-annual progress report will be submitted to NIHR (the funder), the first being submitted 6 months from the date that all funding started (January 2012).

Annual reports will be submitted to the ethics committee and sponsor with the first submitted one year after the date that all study related approvals are in place. These reports will follow the template on the IRAS web site.

For peer review only - http://bmjopen.bmj.com/site/about/guidelines.xhtml

15 DISSEMINATION OF FINDINGS

Study results whether, negative or positive, will be disseminated. Details of the plan for dissemination are detailed below. Some of the outcomes described may be reported in a number of peer reviewed journal articles. The SCOTS Publication and Presentation SOP (18) describes how this will be managed throughout the study and will ensure that NIHR guidance for publications is adhered to.

TABLE 15-1: SCOTS PUBLICATION PLANS

	Date	Description of publication
1	**Jan 2015**	Methods paper: Outlining the methods used in the study including the IT based nutritional blood monitoring and the dataset being collected. Published – Logue et al BMJ Open 2015;5:e008106
2	**Jul 2017**	Health and socio-economic burden of severe obesity in a population undergoing bariatric surgery.
3	**Jul 2019**	The effect of the pre and post-surgery pathway and surgical experience on weight and complications outcomes after bariatric surgery.
4	**Jun 2021**	Change in health and socioeconomic factors 3 years after bariatric surgery.<br>Full HTA report within the NIHR HTA Journal Series.

For peer review only - http://bmjopen.bmj.com/site/about/guidelines.xhtml

16 REFERENCES

(1) Picot J, Jones J, Colquitt JL, Gospodarevskaya E, Loveman E, Baxter L, et al. The clinical effectiveness and cost-effectiveness of bariatric (weight loss) surgery for obesity: a systematic review and economic evaluation. Health Technol Assess 2009 Sep;13(41):1-357, iii.

(2) O'Brien PE, Sawyer SM, Laurie C, Brown WA, Skinner S, Veit F, et al. Laparoscopic adjustable gastric banding in severely obese adolescents: a randomized trial. JAMA 2010 Feb 10;303(6):519-26.

(3) Adams TD, Gress RE, Smith SC, Halverson RC, Simper SC, Rosamond WD, et al. Long-term mortality after gastric bypass surgery. N Engl J Med 2007 Aug 23;357(8):753-61.

(4) Sjostrom L, Narbro K, Sjostrom CD, Karason K, Larsson B, Wedel H, et al. Effects of bariatric surgery on mortality in Swedish obese subjects. N Engl J Med 2007 Aug 23;357(8):741-52.

(5) Dixon JB, O'Brien PE, Playfair J, Chapman L, Schachter LM, Skinner S, et al. Adjustable gastric banding and conventional therapy for type 2 diabetes: a randomized controlled trial. JAMA 2008 Jan 23;299(3):316-23.

(6) Buchwald H, Estok R, Fahrbach K, Banel D, Jensen MD, Pories WJ, et al. Weight and type 2 diabetes after bariatric surgery: systematic review and meta-analysis. Am J Med 2009 Mar;122(3):248-56.

(7) Sjostrom L, Lindroos AK, Peltonen M, Torgerson J, Bouchard C, Carlsson B, et al. Lifestyle, diabetes, and cardiovascular risk factors 10 years after bariatric surgery. N Engl J Med 2004 Dec 23;351(26):2683-93.

(8) Sjostrom L, Lindroos AK, Peltonen M, Torgerson J, Bouchard C, Carlsson B, et al. Lifestyle, diabetes, and cardiovascular risk factors 10 years after bariatric surgery. N Engl J Med 2004 Dec 23;351(26):2683-93.

(9) Mathus-Vliegen EM, de Wit LT. Health-related quality of life after gastric banding. Br J Surg 2007 Apr;94(4):457-65.

(10) Karlsson J, Taft C, Ryden A, Sjostrom L, Sullivan M. Ten-year trends in health-related quality of life after surgical and conventional treatment for severe obesity: the SOS intervention study. Int J Obes (Lond) 2007 Aug;31(8):1248-61.

(11) Ford I, Murray H, Packard CJ, Shepherd J, Macfarlane PW, Cobbe SM. Long-term follow-up of the West of Scotland Coronary Prevention Study. N Engl J Med 2007 Oct 11;357(15):1477-86.

(12) Research Governance Framework for Health and Community Care (Second edition). Scottish Executive Health Department; 2006.

For peer review only - http://bmjopen.bmj.com/site/about/guidelines.xhtml

(13) NHS National Services Scotland. ISD Statistical Disclosure Control Protocol. Edinburgh: ISD Scotland Publications; 2010.

(14) Scottish Intercollegiate Guideline Network. Management of Obesity 115 - A national clincal guideline. Edinburgh: Scottish Intercollegiate Guidelines Network; 2010 Feb 1. Report No.: 115.

(15) Knox H, Davies S. Obestity Treatment Best Practice Guidance. Edinburgh: SGHD Quality Unit, Planning and Quality Division; 12 A.D. Jul 1.

(16) Mid-2011 Population Estimates Scotland Population estimates by sex, age and administrative area.

National Statistics publication for Scotland; 2012 May 31.

(17) Chevallier JM, Paita M, Rodde-Dunet MH, Marty M, Nogues F, Slim K, et al. Predictive factors of outcome after gastric banding: a nationwide survey on the role of center activity and patients' behavior. Ann Surg 2007 Dec;246(6):1034-9.

(18) SCOTS Management Team. Surgical Obesity Treatment Study (SCOTS) Publication and Presentation Group Operating Procedures. 2012.

(19) Scottish Government. Scottish Health Survey 2010 - Volume 2: Technical Report. 2008.

(20) General Register Office. Scottish Census 2011 Household Questionnaire. General Register Office, editor. GRO . 2011.

Ref Type: Online Source

(21) Bruce J, Thornton AJ, Scott NW, Marfizo S, Powell R, Johnston M, et al. Chronic preoperative pain and psychological robustness predict acute postoperative pain outcomes after surgery for breast cancer. Br J Cancer 2012 Sep 4;107(6):937-46.

(22) Mercer SW, Watt GC. The inverse care law: clinical primary care encounters in deprived and affluent areas of Scotland. Ann Fam Med 2007 Nov;5(6):503-10.

(23) Wadden TA, Foster GD. Weight and Lifestyle Inventory (WALI). Obesity (Silver Spring) 2006 Mar;14 Suppl 2:99S-118S.

(24) Diabetes UK. Diabetes Risk Score. 2012. Diabetes UK.

Ref Type: Online Source

(25) Ware J, Jr., Kosinski M, Keller SD. A 12-Item Short-Form Health Survey: construction of scales and preliminary tests of reliability and validity. Med Care 1996 Mar;34(3):220-33.

(26) The EuroQol Group. EuroQol-a new facility for the measurement of health-related quality of life. Health Policy 1990;16(3):199-208.

For peer review only - http://bmjopen.bmj.com/site/about/guidelines.xhtml

(27) Spitzer RL, Kroenke K, Williams JB. Validation and utility of a self-report version of PRIME-MD: the PHQ primary care study. Primary Care Evaluation of Mental Disorders. Patient Health Questionnaire. JAMA 1999 Nov 10;282(18):1737-44.

(28) Cameron IM, Crawford JR, Lawton K, Reid IC. Psychometric comparison of PHQ-9 and HADS for measuring depression severity in primary care. Br J Gen Pract 2008 Jan;58(546):32-6.

(29) Spitzer RL, Kroenke K, Williams JB, Lowe B. A brief measure for assessing generalized anxiety disorder: the GAD-7. Arch Intern Med 2006 May 22;166(10):1092-7.

(30) Kolotkin RL, Crosby RD. Psychometric evaluation of the impact of weight on quality of life-lite questionnaire (IWQOL-lite) in a community sample. Qual Life Res 2002 Mar;11(2):157-71.

(31) Kolotkin RL, Crosby RD, Pendleton R, Strong M, Gress RE, Adams T. Health-related quality of life in patients seeking gastric bypass surgery vs non-treatment-seeking controls. Obes Surg 2003 Jun;13(3):371-7.

(32) Gosman GG, King WC, Schrope B, Steffen KJ, Strain GW, Courcoulas AP, et al. Reproductive health of women electing bariatric surgery. Fertil Steril 2010 Sep;94(4):1426-31.

(33) Derby CA, Araujo AB, Johannes CB, Feldman HA, McKinlay JB. Measurement of erectile dysfunction in population-based studies: the use of a single question self-assessment in the Massachusetts Male Aging Study. Int J Impot Res 2000 Aug;12(4):197-204.

(34) Barry MJ, Fowler FJ, Jr., O'Leary MP, Bruskewitz RC, Holtgrewe HL, Mebust WK, et al. The American Urological Association symptom index for benign prostatic hyperplasia. The Measurement Committee of the American Urological Association. J Urol 1992 Nov;148(5):1549-57.

(35) Abrams P, Avery K, Gardener N, Donovan J. The International Consultation on Incontinence Modular Questionnaire: www.iciq.net. J Urol 2006 Mar;175(3 Pt 1):1063-6.

(36) Macran S, Wileman S, Barton G, Russell I. The development of a new measure of quality of life in the management of gastro-oesophageal reflux disease: the Reflux questionnaire. Qual Life Res 2007 Mar;16(2):331-43.

(37) Grant A, Wileman S, Ramsay C, Bojke L, Epstein D, Sculpher M, et al. The effectiveness and cost-effectiveness of minimal access surgery amongst people with gastro-oesophageal reflux disease - a UK collaborative study. The REFLUX trial. Health Technol Assess 2008 Sep;12(31):1-iv.

(38) Helander A, Lunner K, Leifman A, Borg S. [Alcohol-related problems in connection with overweight surgery]. Lakartidningen 2011 May 4;108(18):964-5.

For peer review only - http://bmjopen.bmj.com/site/about/guidelines.xhtml

(39) Babor TF, Higgins-Biddle JC, Saunders JB, Monteiro MG. The Alcohol Use Disorders Identification Test. World Health Organisation Department of Mental Health and Substance Dependence; 2001 Jan 1. Report No.: WHO/MSD/MSB/01.6a.

(40) Craig CL, Marshall AL, Sjostrom M, Bauman AE, Booth ML, Ainsworth BE, et al. International physical activity questionnaire: 12-country reliability and validity. Med Sci Sports Exerc 2003 Aug;35(8):1381-95.

(41) Roe L, Strong C, Whiteside C, Neil A, Mant D. Dietary intervention in primary care: validity of the DINE method for diet assessment. Fam Pract 1994 Dec;11(4):375-81.

(42) Scheier MF, Carver CS, Bridges MW. Distinguishing optimism from neuroticism (and trait anxiety, self-mastery, and self-esteem): a reevaluation of the Life Orientation Test. J Pers Soc Psychol 1994 Dec;67(6):1063-78.

(43) Foster GD, Wadden TA, Vogt RA, Brewer G. What is a reasonable weight loss? Patients' expectations and evaluations of obesity treatment outcomes. J Consult Clin Psychol 1997 Feb;65(1):79-85.

(44) Health Protection Scotland. The epidemiology of orthopaedic surgical site infection occuring up to one year after surgery: a feasibility study of telephone screening and direct observation by trained healthcare workers. Health Protection Scotland; 2010.

(45) Horan TC, Andrus M, Dudeck MA. CDC/NHSN surveillance definition of health care-associated infection and criteria for specific types of infections in the acute care setting. Am J Infect Control 2008 Jun;36(5):309-32.

(46) Ertlet TW, Marino JM, Mitchell JE. Psychosocial aspects of body contourning surgery after bariatric surgery. In: Shiffman MA, Di Giuseppe A, editors. Body Contouring.Berlin: Springer-Verlag; 2010. p. 633-9.

(47) Keating CL, Moodie ML, Bulfone L, Swinburn BA, Stevenson CE, Peeters A. Healthcare Utilization and Costs in Severely Obese Subjects Before Bariatric Surgery. Obesity (Silver Spring) 2012 May 4.

(48) (DIRUM) Database of Instruments for Resource Use Measurement. 2012. Bangor, Bangor University.

Ref Type: Online Source

(49) Petrou S. WoLFF (Wound dressings in Lower Limb Fracture) Questionnaire. 2012. 1-8-2012.

(50) EPIC Norfolk Study. Food Frequency Questionnaire. Cambridge: University of Cambridge; 2012. Report No.: (CAMB/PQ/6/1205).

For peer review only - http://bmjopen.bmj.com/site/about/guidelines.xhtml

17 Appendix E: Summary of Patient Reported Outcome Questions

Table 17-1 shows a summary table of each set of questions which will be asked of the patient, when they will be asked and if they are validated or not validated.

Questions are classed as not validated in the form they are being asked if they have not been used in a published study and are identified by either a 0 or P in the validated column. 0 means that the set of questions are not validated P means that the questions are partially validated. Partially validated question sets are where a previously published question set has had additional questions added in order to fulfil the SCOTS objectives.

TABLE 17-1: SUMMARY OF SCOTS PATIENT REPORTED OUTCOME MEASURES.

For peer review only - http://bmjopen.bmj.com/site/about/guidelines.xhtml

PROMS	Total	Total Male	Total Femal	Validated	baseline	2 year	3 year	Total
About you								
Demographics – Essential	1	1	1	P	1	1	1	3
Marital Status	1	1	1	1	1	1	1	3
Ethnicity	1	1	1	1	1	0	0	1
Education	1	1	1	1	1	0	0	1
Occupation	1	1	1	P	1	1	1	3
Health History								
Your Medical History	1	1	1	1	1	0	0	1
Your Weight History	1	1	1	1	1	0	0	1
Family History	1	1	1	P	1	0	0	1
Family History of Obesity	1	1	1	0	1	0	0	1
Quality of Life								
SF-12 v2 –licence required	1	1	1	1	1	1	1	3
EQ5D 5L	1	1	1	1	1	1	1	3
Patient Health Questionnaire (PHQ-9) (No permissions Free)	1	1	1	1	1	1	1	3
Generalized Anxiety Disorder GAD-7 (No permissions Free)	1	1	1	1	1	1	1	3
Impact of Weight								
Impact of Weight on Quality of Life (IWQOL-Lite) – Licence required	1	1	1	1	1	1	1	3
Reproductive Health – Females only	1	0	1	1	1	1	1	3
Massachusetts Male Aging Study: Single Question Assessment Males only	1	1	0	1	1	1	1	3
International Prostate Symptom Score (IPSS) Males only	1	1	0	1	1	1	1	3
Continence	1	1	1	1	1	1	1	3
Reflux Questionnaire	1	1	1	1	1	1	1	3
Lifestyle								
Smoking	1	1	1	1	1	1	1	3
The Alcohol Use Disorders Identification Test: Self-Report Version	1	1	1	1	1	1	1	3
Physical Activity IPAC short form self administered	1	1	1	1	1	1	1	3
Nutrition	1	1	1	1	1	1	1	3
Personal Outlook								
Life Orientation Test (REVISED)	1	1	1	1	1	1	0	2
Expectations of Surgery	1	1	1	P	1	0	0	1
Surgical Recovery								
Post operative pain (1 month acute, 6 month-1 year chronic)	1	1	1	0	0	0	0	0
Surgical site infection	1	1	1	1	0	0	0	0
Skin Excess	1	1	1	0	0	0	0	0
Plastic surgery	1	1	1	P	0	0	0	0
Healthcare and Support								
Outpatient care	1	1	1	1	1	1	1	3
Contacts with other health and social care professionals	1	1	1	1	1	1	1	3
Devices and specialist equipment	1	1	1	1	1	1	1	3
Benefits	1	1	1	1	1	1	1	3
Multivitamins and Supplements use	1	1	1	1	1	1	1	3
	34	33	32	**26**	**30**	**23**	**22**	**75**

For peer review only

For peer review only - http://bmjopen.bmj.com/site/about/guidelines.xhtml

This page is intentionally blank

For peer review only

For peer review only - http://bmjopen.bmj.com/site/about/guidelines.xhtml

Medical history questions included in baseline questionnaires/patient reported outcome measures (PROMs).

Why we are asking you these questions?

It is important to know what illness patients have before their surgery so we can look at how much overall health has changed after their weight loss surgery.

Medical History

1. Do you have a history of any of the following? – tick all that apply

☐ Deep vein thrombosis	(Deep vein thrombosis (DVT) is a blood clot in one of the deep veins in the leg.)
☐ Pulmonary embolism	(A pulmonary embolism is a blood clot in the pulmonary artery which is the blood vessel that transports blood from the heart to the lungs.)
☐ High blood pressure	
☐ Diabetes	
☐ Angina/heart attack	
☐ Heart Failure	
☐ Stroke/mini-stroke	
☐ Arthritis	
☐ Back problems	
☐ Chronic Bronchitis	
☐ Eczema/psoriasis	
☐ Asthma	
☐ Thyroid problems	
☐ Migraine	
☐ Anxiety/depression	
☐ Kidney disease	
☐ Liver disease	
☐ Cancer	
☐ Irritable bowel syndrome	
☐ Doctor diagnosed sleep apnoea	(Obstructive sleep apnoea (OSA) is a condition that causes interrupted breathing during sleep and requires a machine to keep airways open while you sleep. Only check this box if your doctor has diagnosed sleep apnoea.)
☐ Other (please specify _______________)	

For peer review only

SCOTS

Smoking

Patient Questionnaire

Draft 01

Lifestyle

This section asks about your smoking, drinking, eating and exercising habits.

Why are we asking you these questions

Smoking habits may alter after weight loss surgery.

<u>Smoking</u>

1. Smoking history

Current ☐ 1

Former ☐ 2

Never ☐ 3

If Current,

1a. How many per day? |__|__|__|

If former,

1a. Year stopped? |__|__|__|__| Y Y Y Y

For peer review only

SCOTS

Work

Patient Questionnaire

Draft 02

Why are we asking you these questions

The ability to work or go about usual activity is an important part of a patient's overall health and quality of life. We are interested to see if there is a change in your occupation before and after your weight loss surgery.

Occupation

1. Please select a category that best describes your current employment status

Working full time (30hrs or more per week)	☐ 1	please go to section A
Working part time (less than 30hrs or more per week)	☐ 2	please go to section A
Unable to work because of illness or disability	☐ 3	please go to section B
Student	☐ 4	please go to section A
Unemployed and looking for work	☐ 5	please go to section B
Carer for children or relative	☐ 6	please go to section B
At home and not looking for paid employment	☐ 6	please go to section B
Other	☐ 6	please go to section B

Section A

1. What is your current occupation? Since many people have more than one job at a given time, we would like to know about the job that is your primary source of income

____

2. Have you had any days off sick in the last month? Yes ☐ 1 No ☐ 2 Not applicable ☐ 3

   a. If Yes, how many days in total (please only include the number of days you were not able to work)? |_|_|_|

3. Are you receiving benefits? Yes ☐ 1 No ☐ 2

Section B

1. Are you receiving benefits? Yes ☐ 1 No ☐ 2

For peer review only

Social security (benefits) questions included in baseline questionnaires/patient reported outcome measures (PROMs).

Why we are asking you these questions?

Weight loss surgery can be expensive, but often the improvements in patients' health are great. This is to be used in health economic analysis which balances the cost of weight loss surgery with the improvements in patients' health and wellbeing and overall cost of bariatric surgery.

Due to the personal nature of these questions, you may choose not to complete them.

I am happy to complete these questions

- ☐ Yes
- ☐ No

If No, please go to the next section.

Benefits

1. In the past three months, you may have received some benefits from the government to support you. In the table below, please tick all benefits you have received in the past 3 months.

- ☐ Attendance Allowance
- ☐ Carer's Allowance
- ☐ Child Tax Credit
- ☐ Council Tax Benefit
- ☐ Disability Living Allowance—caring
- ☐ Disability Living Allowance—mobility
- ☐ Employment and Support Allowance
- ☐ Housing Benefit
- ☐ Income Support
- ☐ Jobseeker's Allowance
- ☐ Pension Credit
- ☐ Statutory Sick Pay
- ☐ State Pension
- ☐ Other (please specify _______________)
- ☐ None

For peer review only

Healthcare utilisation (devices and specialist equipment) questions included in baseline questionnaires/patient reported outcome measures (PROMs).

Why we are asking you these questions?

Some people may require specialist equipment or devices as a result of their weight and changes in requirements for equipment will be considered in the overall cost of weight loss surgery.

Devices and Specialist Equipment

1	Do you currently use any aids or specialist equipment?	☐ Yes<br>☐ No<br>If No, move to next section	
1a	Stair lift	☐ Yes<br>☐ No	If Yes, funded by:<br>☐ provided by Social Services<br>☐ provided by hospital<br>☐ bought by you
1b.	Grab rail	☐ Yes<br>☐ No	If Yes, funded by:<br>☐ provided by Social Services<br>☐ provided by hospital<br>☐ bought by you
1c.	Bariatric bed	☐ Yes<br>☐ No	If Yes, funded by:<br>☐ provided by Social Services<br>☐ provided by hospital<br>☐ bought by you
1d.	Bariatric shower stool	☐ Yes<br>☐ No	If Yes, funded by:<br>☐ provided by Social Services<br>☐ provided by hospital<br>☐ bought by you
1e.	Bariatric chair	☐ Yes<br>☐ No	If Yes, funded by:<br>☐ provided by Social Services<br>☐ provided by hospital<br>☐ bought by you
1f.	Wheel chair (bariatric)	☐ Yes<br>☐ No	If Yes, funded by:<br>☐ provided by Social Services<br>☐ provided by hospital<br>☐ bought by you
1g.	Mobility scooter	☐ Yes<br>☐ No	If Yes, funded by:<br>☐ provided by Social Services<br>☐ provided by hospital<br>☐ bought by you
1h.	Dressing aids	☐ Yes<br>☐ No	If Yes, funded by:<br>☐ provided by Social Services<br>☐ provided by hospital<br>☐ bought by you
1i.	Other	☐ Yes<br>☐ No	If Yes, please describe any other devices or specialist equipment you use.<br>_______________<br>_______________<br>Funded by:<br>☐ provided by Social Services<br>☐ provided by hospital<br>☐ bought by you

For peer review only
